# Drug–gene Interaction Screens Coupled to Tumor Data Analyses Identify the Most Clinically Relevant Cancer Vulnerabilities Driving Sensitivity to PARP Inhibition

**DOI:** 10.1158/2767-9764.CRC-22-0119

**Published:** 2022-10-21

**Authors:** Kunzah Jamal, Alessandro Galbiati, Joshua Armenia, Giuditta Illuzzi, James Hall, Sabrina Bentouati, Daniel Barrell, Miika Ahdesmäki, for the Functional Genomics Centre Group, Mark J. O'Connor, Elisabetta Leo, Josep V. Forment

**Affiliations:** 1 DDR Biology, Bioscience, AstraZeneca, Cambridge, United Kingdom.; 2 Early Computational Oncology, Oncology R&D, AstraZeneca, Cambridge, United Kingdom.; 3 AstraZeneca-Cancer Research Horizons Functional Genomics Centre, Jeffrey Cheah Biomedical Centre, University of Cambridge, Cambridge, United Kingdom.

## Abstract

PARP inhibitors (PARPi) are currently indicated for the treatment of ovarian, breast, pancreatic, and prostate cancers harboring mutations in the tumor suppressor genes *BRCA1* or *BRCA2*. In the case of ovarian and prostate cancers, their classification as homologous recombination repair (HRR) deficient (HRD) or mutated also makes PARPi an available treatment option beyond *BRCA1* or *BRCA2* mutational status. However, identification of the most relevant genetic alterations driving the HRD phenotype has proven difficult and recent data have shown that other genetic alterations not affecting HRR are also capable of driving PARPi responses. To gain insight into the genetics driving PARPi sensitivity, we performed CRISPR-Cas9 loss-of-function screens in six PARPi-insensitive cell lines and combined the output with published PARPi datasets from eight additional cell lines. Ensuing exploration of the data identified 110 genes whose inactivation is strongly linked to sensitivity to PARPi. Parallel cell line generation of isogenic gene knockouts in ovarian and prostate cancer cell lines identified that inactivation of core HRR factors is required for driving *in vitro* PARPi responses comparable with the ones observed for *BRCA1* or *BRCA2* mutations. Moreover, pan-cancer genetic, transcriptomic, and epigenetic data analyses of these 110 genes highlight the ones most frequently inactivated in tumors, making this study a valuable resource for prospective identification of potential PARPi-responsive patient populations. Importantly, our investigations uncover *XRCC3* gene silencing as a potential new prognostic biomarker of PARPi sensitivity in prostate cancer.

**Significance:**

This study identifies tumor genetic backgrounds where to expand the use of PARPis beyond mutations in *BRCA1* or *BRCA2*. This is achieved by combining the output of unbiased genome-wide loss-of-function CRISPR-Cas9 genetic screens with bioinformatics analysis of biallelic losses of the identified genes in public tumor datasets, unveiling loss of the DNA repair gene *XRCC3* as a potential biomarker of PARPi sensitivity in prostate cancer.

## Introduction

The breast cancer susceptibility genes *BRCA1* and *BRCA2* (*BRCA* genes) are well-known tumor suppressors whose inactivation increases the probability of developing cancer, particularly of breast and ovarian origin (1), but also in the pancreas and prostate (2, 3). BRCA proteins are key components of the homologous recombination repair (HRR) pathway of DNA repair and their deficiency fosters genome instability, one of the hallmarks of cancer (reviewed in ref. 4). PARP inhibitors (PARPi) are current treatment options for patients with ovarian, breast, pancreatic, and prostate cancer harboring mutations in *BRCA1* or *BRCA2* (BRCAm) but they have also been approved in broader patient populations in ovarian and prostate cancer (5–7). These broader approvals have involved the identification of tumors with HRR deficiency (HRD) beyond BRCAm, either by the use of genomic detection of genome instability patterns (“genomic scars”) linked to HRD or by genetic identification of mutations in non-BRCA genes linked to HRR (HRRm; refs. 8, 9).

Different HRRm gene panels have been used in clinical trials to identify the best biomarkers of response to PARPi. Most of these efforts have almost invariably found that HRRm beyond *BRCA* genes are rare, making it difficult to assess the validity of some of these biomarkers (7, 10, 11). However, there seems to be a significant HRD tumor population identified through detection of genomic scars for which current HRRm gene panels fail to explain their genetic origin (5, 6). Moreover, recent preclinical work has highlighted that mutations in genes not involved in HRR could be valid biomarkers to predict PARPi sensitivity (12–14), probably suggesting the need of a more holistic gene selection strategy in these panels.

In this work, and in an effort to identify cancer-relevant genetic biomarkers of PARPi responses in an unbiased way, we carried out CRISPR-Cas9 genome-wide loss-of-function (LoF) screens (reviewed in ref. 15) to uncover genetic determinants of sensitivity to PARPi in a variety of cell lines. We coupled the output with a bespoke analysis pipeline of tumor genetic and epigenetic data to uncover the most clinically relevant vulnerabilities to PARPi, identifying potential new biomarkers of PARPi sensitivity. In addition, we provide for the first time head-to-head comparison of PARPi responses between defects in *BRCA* genes and other genes identified by our analyses through generation of isogenic cell line pairs in clinically relevant tissue types, namely ovarian and prostate cancer cells.

## Materials and Methods

### Cell Lines and Compounds

LNCAP (RRID:CVCL_0395), DU145 (NCI-DTP catalog no. DU-145, RRID:CVCL_0105), and SKOV3 (NCI-DTP catalog no. SKOV-3, RRID:CVCL_0532) cells were obtained from ATCC. The DLD1 wild-type (RRID:CVCL_0248) and *BRCA2*^−/−^ (RRID:CVCL_HD57) cell lines were purchased from Horizon Discovery. Cell line identification (short tandem repeat typing) was validated using the CellCheck assay (IDEXX Bioanalytics). All cell lines were validated free of virus and *Mycoplasma* contamination using the MycoSEQ assay (Thermo Fisher Scientific) or STAT-Myco assay (IDEXX Bioanalytics). All cell lines were grown according to supplier instructions. LNCAP, DU145, and DLD1 were grown in RPMI1640 growth media (Corning 17-105-CV) supplemented with 10% FBS and 2 mmol/L glutamine. SKOV3 were grown in McCoy's 5A (Modified) Medium (Thermo Fisher Scientific 16600082). Olaparib and AZD0156 (ATM inhibitor, ATMi) were made by AstraZeneca, carboplatin and cisplatin were bought from Tocris Bioscience (catalog no. 2626 and 15663-27-1). Olaparib, ATMi, and carboplatin were all solubilized in DMSO at 10 mmol/L stock concentration. Cisplatin was solubilized in an aqueous solution at 1.67 mmol/L.

### Gene Expression Analysis by qRT-PCR

Total RNA was isolated from cells in 96-well plates using the Qiagen FastLane Cell Probe Kit (QIAGEN, 216413), according to the manufacturer's instructions to a final volume of 40 μL per well, at the indicated timepoint after treatment. Gene expression was evaluated by qPCR using the ONE-step QuantiTect Probe RT-PCR Kit (Qiagen, catalog no./ID: 204445). For each reaction, 2 μL of RNA were used. Real-time qPCR reactions were performed on a Roche Lightcycler 480 II Sequence Detection System.

The following Taqman probes were obtained from Thermo Fisher Scientific: BRCA2 (Hs00609073_m1), IPO8 (Hs00914057_m1), RAD51B (Hs01568768_m1), RAD54L (Hs00941668.m1), Actin (Hs01060665.g1).

For data analysis, Δ*C*_t_ was calculated by subtracting average *C*_t_ of housekeeping genes from each *C*_t_. An average Δ*C*_t_ for the control group was calculated and subtracted from ΔC_t_ to calculate negative ΔΔ*C*_t_. 2^−ΔΔ*C*_t_^ was used to calculate fold change.

### Immunoblotting

Cells were lysed in RIPA buffer (Sigma-Aldrich) supplemented with protease inhibitors (Roche), phosphatase inhibitors (Sigma-Aldrich), and benzonase (Merck, catalog no. 103773). After 30 minutes of incubation on ice, the lysates were cleared through centrifugation at 15,000 rpm 4°C for 20 minutes and supernatant were kept for sample loading. NuPAGE LDS Sample Buffer (Thermo Fisher Scientific, catalog no. NP0008) and NuPAGE Sample Reducing Agent (Thermo Fisher Scientific, catalog no. NP0004) were added to the samples. Equal amounts of whole cell lysates were separated on 4%–12% Bis-Tris NuPAGE gels and analyzed by standard immunoblotting. Immunoblots are representative of experiments that were performed at least twice. For ATM signaling pathway analysis, cells were pretreated with the ATMi for 1 hour prior to ionizing radiation induction by a high-voltage X-ray-generator tube (Faxitron X-Ray Corporation).

The following antibodies were used: actin (Sigma-Aldrich, catalog no. A2228, RRID:AB_476697, 1:2,000 dilution), ATM (Abcam, catalog no. ab78, RRID:AB_306089, 1:2,000), pATM (S1981; Abcam, catalog no. ab81292, RRID:AB_1640207, 1:1,000), BRCA1 (Millipore, catalog no. OP92, RRID:AB_2750876, 1:1,000), Cas9 (Abcam, catalog no. ab204448, RRID:AB_2893352, 1:1,000), GAPDH (Cell Signaling Technology, catalog no. 2118, RRID:AB_561053, 1:1,000), KAP1 (Abcam, catalog no. ab10483, RRID:AB_297222, 1:1,000), pKAP1 (S824; Abcam, catalog no. ab70369, RRID:AB_1209417, 1:1,000), pKAP1 (S473; BioLegend, catalog no. 654102, RRID:AB_2561782, 1:1,000), CHEK2 (Cell Signaling Technology, catalog no. 2661, RRID:AB_331479, 1:1,000), PALB2 (Bethyl, catalog no. A301-246A, RRID:AB_890607, 1:1,000), RAD51C (Novus, catalog no. NB100-177, RRID:AB_10001856, 1:1,000).

### Immunofluorescence—RAD51 Foci Assay

Cells were plated in 96-well plates (Perkin Elmer CellCarrier-96 Ultra Microplates, catalog no. 6055302) to reach 70% confluency the next day and ionizing radiation was induced by a high-voltage X-ray-generator tube (Faxitron X-Ray Corporation). For EdU staining, cells were incubated with 10 μmol/L EdU for 1 hour prior to fixation. At the indicated timepoints after treatment, cells were fixed in 4% paraformaldehyde for 15 minutes at room temperature and then permeabilized in PBS+0.1% Triton X-100 for 10 minutes at room temperature. Blocking was performed using 0.5% BSA + 0.2% gelatin from cold water fish skin (Sigma, catalog no. G7765) in PBS for 1 hour at room temperature. Cells were washed with PBS and incubated with EdU click-It reaction [HEPES pH 7.5 125 mmol/L, CuSO_4_·5H_2_O 20 mmol/L, Ascorbate 100 mmol/L, Alexa Azide 647 (Sigma, A10277) 5 mmol/L], for 20 minutes at room temperature in the dark. The Click-it reaction was removed and cells were washed three times with PBG. Primary antibodies were incubated overnight at 4°C, followed by Alexa-Fluor secondary antibodies and DAPI (Sigma, 1 μg/mL) for 1 hour at room temperature. The following antibodies were used: Alexa Fluor 594 secondary (Thermo Fisher Scientific, catalog no. A-11037, RRID:AB_2534095, 1:2,000 dilution), Alexa Fluor 488 secondary (Thermo Fisher Scientific, catalog no. A-11029, RRID:AB_2534088, 1:2,000), γH2AX (Millipore, catalog no. 05-636, RRID:AB_309864, 1:2,000), RAD51 (Bioacademia 70-001, 1:7,000).

### Colony Formation Assay

To evaluate the efficacy of olaparib and platinum, a colony formation assay (CFA) was employed. Here, cells were seeded at low density in a 24-well plate and exposed to olaparib for a time corresponding to more than five replication cell cycles: all except BRCA2, PALB2, and RAD51C knockout (KO) cells were plated at 500 cells per well for 9–14 days whereas BRCA2, PALB2, and RAD51C KO cells were plated at 800 cells per well for 10–14 days. After cell attachment, compounds were dispensed with automated digital D300 HP dispenser (Tecan). Drugs were added from compound stocks dissolved in DMSO, in seven titration dilutions, where each concentration was tested in triplicate in each plate. DMSO served as a vehicle control. The concentration ranges tested were chosen to obtain dose–response and to cover minimal to maximal activity of a given compound. Plates were incubated at 37°C, 5% CO_2_ for indicated times. Colony formation and absence of contamination was regularly checked on the microscope. Next, colonies were fixed and stained using Blue-G-250 brilliant blue [#B8522-1EA, Sigma, reconstituted in 25% (v/v) methanol and 5% (v/v) acetic acid] for 15 minutes. Prior to imaging, plates were thoroughly washed with deionised water (dH_2_O). Plates with stained colonies were scanned with GelCount (Oxford OPTRONIX) at 600 dpi resolution. Colony formation was scored by quantifying the total optical density measured with ImageJ software (RRID:SCR_003070), using a 24-well plate region of interest mask. Data analysis was performed by normalization on the vehicle treated of the respective plate set as = 100. Data were normalized and plotted to respective vehicle control (set as = 100) and IC_50_ were calculated using Prism GraphPad software.

### Cell Proliferation Assay—CellTiter-glo

To evaluate the efficacy of olaparib and platinum, a CellTiter-glo assay was employed. Here, cells were seeded at low density (400–500 cells per well) in a 96-well plate and exposed to olaparib for 7–8 days allowing the cells to go through at least five replication cycles. After cell attachment, compounds were dispensed with automated digital D300 HP dispenser (Tecan). Drugs were added from compound stocks dissolved in DMSO, in seven titration dilutions, where each concentration was tested in a triplicate in each plate. DMSO served as a vehicle control. The concentration ranges tested were chosen to obtain dose–response and to cover minimal to maximal activity of a given compound. Plates were incubated at 37°C, 5% CO_2_ for indicated times. Cell growth was stopped by adding CellTiter-Glo as per manufacturer's instructions (Promega; G7570). Data analysis was performed by normalization on the vehicle treated of the respective plate set as = 100. Data were normalized and plotted to respective vehicle control (set as = 100) using Prism GraphPad software.

### Lentiviral Production and Transduction

HEK293T cells seeded at the concentration of 400,000 cells per well in 6-well plates were transfected with the Cas9-expressing plasmid or the single-guide RNA (sgRNA) targeting plasmid and viral packaging, psPax2 (RRID:Addgene_12260) and pMD2.G (RRID:Addgene_12259) at the following mixing ratio: 0.9 μg lentiviral vector (Cas9 or sgRNA cloned into vector), 0.9 μg psPax2 and 0.2 μg pMD2.G, 2 μL PLUS reagent, 6 μL Lipofectamine LTX (Thermo Fisher Scientific, catalog no. 15338100) in 500 μL per well. The reaction mix was incubated for 30 minutes at room temperature and then added to the cells in 1.5 mL Opti-MEM. A total of 6 hours after transfection, OPTIMEM was replaced with fresh medium and 72 hours after transfection the virus-containing supernatant was collected and filtered with 0.45 μmol/L filters.

### Generation of Cas9-expressing Cells

To produce SKOV3 cells stably expressing Cas9, these were transduced with the pKLV2-EF1a-BsdCas9-W (RRID:Addgene_67978) expressing vector. To establish the LNCAP cell line expressing a doxycycline-inducible Cas9, cells were transduced with the pBSK-TOIC-Cas9-T2A-TagBFP-Obl-r26-AAVS-Invneo vector (16). Following transduction, cells were selected with blasticidine at 15 μg/mL for three passages and analyzed by Western blot analysis.

### sgRNA Cloning Into Lentiviral Vector

For the SKOV3, DLD1 and LNCAP KO generation, the sgRNA targeting the sequence of interest was cloned into the Bbsl region of the vector pKLV-U6gRNA(BbsI)-PGKpuro2ABFP (RRID:Addgene_50946) or pKLV2-hU6gRNA5(BbsI)-EF1a-mClover3-T2A-HygR-W, as previously described in ref. 17. Correct cloning was verified via Sanger sequencing.

The following sgRNA sequences (5′ to 3′) were used for cell line generation: ATM (GTTGGTTACATACTTGGACT, AZ generated; AGTTGACAGCCAAAGTCTTG, by Horizon), BRCA2 (TCTACCTGACCAATCGATG and GCCCATTGATGGCTAAAAC, AZ generated), PALB2 (CGATTCACTTACCTGAAGG, AZ generated), RAD51B (GCTTGTGGATCCCTCACAG, AZ generated; AAACAAGTTCTTGGCAAGAG, by Horizon), RAD51C (CACAAGAAGTGTACAGCAC, AZ generated), RAD54L (ATCCTTAGATCCTCCATCGA, by Horizon), XRCC3 (CAAACUGAAAUCGGUAAAGG, by Synthego).

### Generation of CRISPR KO Cell Lines

To generate the CRISPR/Cas9 KO cell lines, cells were seeded at 100,000 cells per well in a 6-well plate. For the LNCAP cells, Cas9 was induced with 100 ng/mL doxycyclin (Sigma) to allow Cas9 expression and after 24 hours the cell medium was refreshed without doxycyclin. The next day, cells were transduced with the lentivirus containing the sgRNA targeting the gene of interest or a nontargeting sgRNA (control, CTRL, cell line) as described above. Viral supernatant was mixed in 2 mL cell culture medium supplemented with 8 μg/mL polybrene (Millipore) and further incubated overnight at 37°C. The medium was refreshed on the following day and the transduced cells were cultured further. A total of 48 hours following transduction, transduced cells were moved into antibiotic selection (hygromycin, 100 μg/mL or puromycin, 0.5 μg/mL) for 5 days. Cell lines were expanded and the KO of the gene of interest was validated by immunoblot, TIDE or ICE sequencing (18, 19) or an RT-PCR to assess the KO efficiency. Single-cell clones were derived from the KO pools from serial dilution plating in 96-well plates and KO efficiency was validated with Western blot analysis, TIDE, or RT-PCR.

### Statistical Analyses

Results are shown as mean ± SEM or percentages ± 95% confidence interval as indicated. *P* value was calculated by Student two-tailed *t* test or *χ*^2^ test, respectively, using Prism software. Mutual exclusivity analysis was performed using Fisher exact test.

### CRISPR Screens and Data Analysis

CAMA1 (RRID:CVCL_1115), U2OS (RRID:CVCL_0042), DLD1 (RRID:CVCL_0248), T47D (RRID:CVCL_0I95), HCC1806 (KCB catalog no.# KCB 2014032YJ, RRID:CVCL_1258), and BT549 (NCI-DTP catalog no. BT-549, RRID:CVCL_1092) cancer cells were obtained from ATCC and infected with lentiviral particles containing the whole-genome sgRNA library, subjected to puromycin selection, and passaged to ensure loss of affected protein products. Puromycin-resistant cells were exposed to 1 μmol/L olaparib for 21 days, and resistant pools were isolated. Genomic DNA was extracted from these and from parallel cell cultures treated in the absence of olaparib, and DNA libraries were prepared and sequenced. Genomic DNA was extracted and gRNAs sequenced as described previously (17). Single-end Illumina sequencing reads of 19 nucleotides were counted for each gRNA using in-house written software.

For all the screens each replicate of plasmid, DMSO control and PARPi-treated (or *PARP1* KO) cell line sequenced samples were first counted for exact matches of the Yusa library (20). The counts were then analyzed for negative selection that is, depletion in the treatment versus control replicates (two each) by the MAGeCK algorithm (21). Genes whose FDR was less than or equal to 0.1 were included as sensitizing hits. In addition, each DMSO control sample was further assessed against the plasmid sample using BAGEL (22) to ensure the expected depletion of essential genes as a quality control measure.

### Biallelic Inactivation Analysis in The Cancer Genome Atlas Data

Biallelic inactivation prevalence was estimated from The Cancer Genome Atlas (TCGA) pan-cancer data by evaluating prevalence of somatic/germline alterations using internal variant calls (23, 24), homozygous deletion and promoter methylation data using level 3 data from TCGA. Biallelic inactivation was defined as (i) a germline pathogenic mutation with loss of heterozygosity (LOH) of the wild-type allele, (ii) a germline pathogenic mutation and a somatic pathogenic mutation, (iii) a somatic pathogenic mutation with LOH of the wild-type allele, (iv) two different somatic pathogenic mutations, (v) homozygous deletion, or (vi) promoter hypermethylation.

### Data Availability Statement

The data generated in this study are available within the article and its Supplementary Data. Raw data from the CRISPR screens performed in this study can be found at the European Nucleotide Archive at EMBL-EBI under accession number PRJEB54620.

## Results

### CRISPR Screens to Identify Genes Involved in the Response to PARPi

**FIGURE 1:**
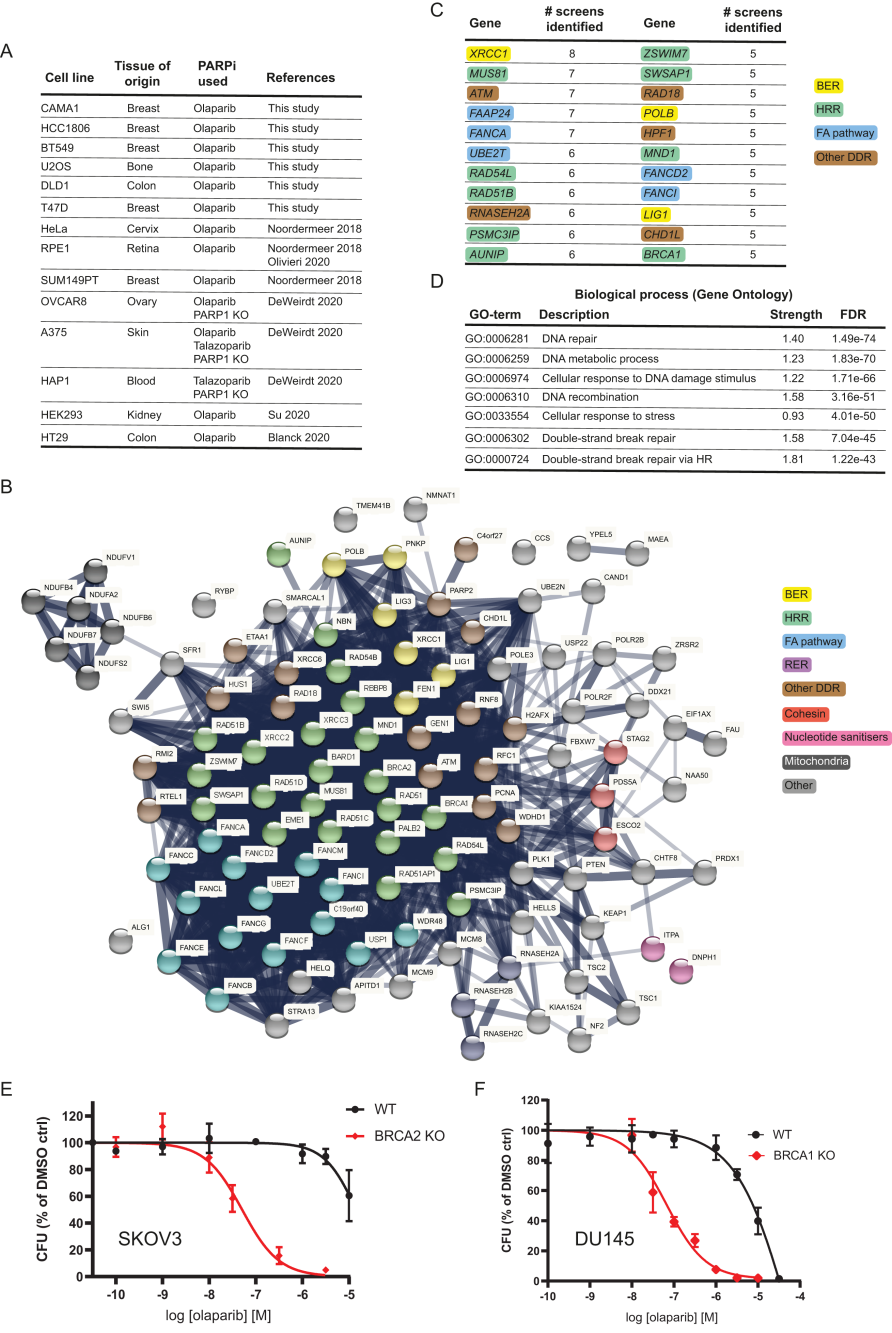
Identification of biomarkers of PARPi sensitivity through CRISPR-Cas9 LoF screens. **A,** Summary of cell lines and CRISPR screens performed or analyzed in this study. Further details can be found in the Materials and Methods section. **B,** STRING pathway analysis of the 110 genes identified in at least two CRISPR screens whose loss sensitizes cells to PARPi treatment. BER: base excision repair; HRR: homologous recombination repair; FA: Fanconi anaemia pathway; RER: ribonucleotide excision repair; DDR: DNA damage response. **C,** Ranking of the top 22 genes identified in our analyses. Genes are color coded for the different DNA repair pathways they are primarily linked to. Acronyms are as in **B**. **D,** Top seven biological processes enriched in the pathway analysis. Dose–response curve for SKOV3 BRCA2 KO (clone 13; **E**) and DU145 BRCA1 KO (clone A1; **F**) isogenic pairs treated with olaparib for 10–14 days in clonogenic survival assays. Results are shown as mean of *n* = 4 biological replicates ± SD for the dose–response curves.

As a way to uncover potential biomarkers of response to PARPi in an unbiased way, we performed genome-wide CRISPR-Cas9 LoF screens in six tumor cell lines from different tissue of origin (breast, colon, and bone), looking for genes whose inactivation would render cells sensitive to PARPi. The six cell lines were chosen by them carrying no genetic or epigenetic alterations in *BRCA* or other HRR genes and presenting a IC_50_ for olaparib at least 10-fold higher than the values reported for *BRCAm* cell lines in colony-forming assays (in the 10–100 nmol/L range; ref. 25) to maximize the chances of identifying sensitization events. The output was combined with results from published CRISPR screens in eight other cell lines from different tissue of origin (cervix, retina, breast, ovary, skin, blood, kidney, and colon) that, except one (SUM149PT, a *BRCA1 m* breast cancer cell line), do not carry genetic or epigenetic alterations in *BRCA* or other HRR genes (refs. 12, 26–29; Fig. 1A). To standardize the results, all raw data (where available) were run through the same analysis pipeline (see Materials and Methods), which identified 1,147 genes whose loss would confer sensitivity to PARPi (or compromised fitness in a PARP1-deficient background; Supplementary Table S1). As a way to generate a list of high confidence genes, we selected genes that were identified in more than one independent screen, which reduced the number of genes in the list to 110 (Fig. 1B; Supplementary Table S2). Pathway analysis of these 110 genes revealed a strong enrichment in DNA repair genes, especially in genes involved in HRR, as expected by the ranking of genes that were identified more often in all screens analyzed (Fig. 1C and D).

Our analyses identified *bona fide* clinical biomarkers of sensitivity to PARPi such as *BRCA1* and *BRCA2*. In addition, we also identified others such as the RNASEH2 complex genes (*RNASEH2A, RNASEH2B, RNASEH2C*); *CHD1L*, which encodes the chromatin remodeler ALC1; the gene encoding the nucleotide sanitizer DNPH1 or genes involved in DNA base excision repair (*XRCC1, POLB, LIG1*), which have already been shown to play roles in PARPi responses that are not dependent on HRR defects (refs. 12–14, 30–32; Fig. 1B and C).

Lack of relevant cellular models has prevented the direct comparison between the *in vitro* responses to PARPi in cell lines deficient in validated clinical biomarkers such as *BRCA1* or *BRCA2* versus other HRR genes. This is particularly the case in cell lines of ovarian and prostate origin. As such, we decided to generate CRISPR-mediated *BRCA1* or *BRCA2* gene KOs in the ovarian adenocarcinoma cell line SKOV3 and the prostate carcinoma DU145 cell line (Supplementary Fig. S1A and Materials and Methods). These cell lines were chosen based on their innate resistance to olaparib treatment, which would make acquisition of sensitivity caused by any given gene KO to be easier to observe, and by them being amenable to CFAs, the gold standard to measure PARPi sensitivity *in vitro* (see Materials and Methods).

We generated several *BRCA2* KO clones in SKOV3 cells and *BRCA1* KO clones in DU145 cells and validated them functionally to confer sensitivity to olaparib using the CFA (Fig. 1E and F; Supplementary Fig. S1B and S1D). The IC_50_ values for olaparib in the *BRCA1* or *BRCA2* KO cell lines we generated (0.067 μmol/L and 0.051 μmol/L, respectively) are in line with previously published data (25), further validating our approach.

### Defects in Core HRR Factors Phenocopy BRCA Deficiency

**FIGURE 2:**
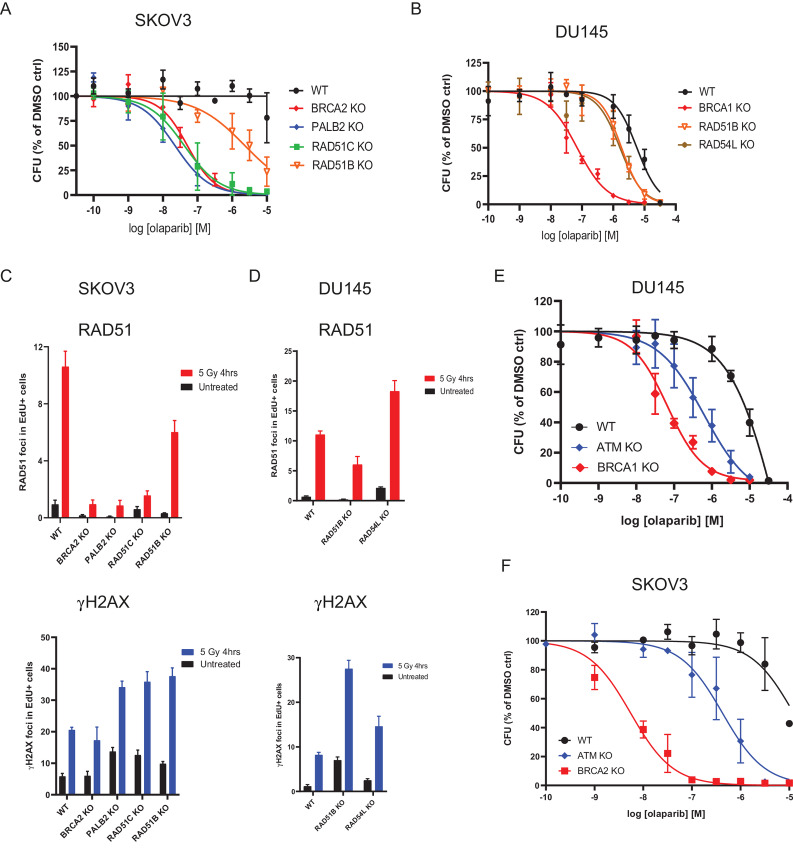
CRISPR/Cas9 KO of *PALB2, RAD51B, RAD51C*, *RAD54L,* or *ATM* sensitises cell models to olaparib treatment. Dose–response curves for SKOV3 (**A**) and DU145 (**B**) isogenic pairs treated with olaparib for 10–14 days in clonogenic survival assays. Results are shown as mean of *n* = 4 biological replicates ± SD. Quantification of RAD51 and γH2AX foci by immunofluorescence in SKOV3 (**C**) and DU145 (**D**) isogenic pairs 4 hours after treatment with 5 Gy of ionizing radiation or in untreated cells. Results are shown as mean number of foci per nucleus in replicating (EdU-positive) cells (*n* = 3 biological replicates ± SD). Dose–response curves for DU145 (**E**) and SKOV3 (**F**) isogenic pairs treated with olaparib for 10–14 days in clonogenic survival assays. Results are shown as mean of *n* = 4 biological replicates ± SD for the dose–response curves. Curves for WT, *BRCA1* KO DU145, and *BRCA2* KO SKOV3 are the same as the ones shown in Fig. 1.

PALB2 and the RAD51 paralogs are key proteins in the HRR pathway and we identified their loss as a cause of sensitivity to PARPi in our CRISPR screen analyses (Fig. 1B; Supplementary Table S2). Mammalian cells with mutations in these factors display increased spontaneous chromosomal abnormalities and sensitivity to DNA-damaging agents (33, 34). Mutations in *PALB2, RAD51B,* or *RAD51C* have been explored in clinical trials as biomarkers of sensitivity to PARPi (7, 10, 11). As such, we generated *PALB2, RAD51B,* or *RAD51C* KO clones in the SKOV3 ovarian cell line (Supplementary Fig. S2A–F) and compared their sensitivity to olaparib against the *BRCA2* KO SKOV3 cell line. Importantly, *PALB2* or *RAD51C* deficiency phenocopied *BRCA2* loss, while the level of sensitization provided by *RAD51B* inactivation was more modest (Fig. 2A). We generated an additional *RAD51B* KO in the DU145 prostate cell line (Supplementary Fig. S2G) and compared its sensitivity to olaparib against the *BRCA1* KO DU145 cell line. Similar to what we observed in SKOV3, RAD51B inactivation caused a more modest sensitization to olaparib than that observed upon BRCA1 loss (Fig. 2B). Interestingly, a similar level of sensitization to olaparib in *RAD51B* KO cells was observed upon inactivation in DU145 of the ATPase RAD54L (Fig. 2B; Supplementary Fig. S2H), whose loss also conferred sensitivity to PARPi in CRISPR screens (Supplementary Table S2).

To better understand the different responses observed with the several gene KO produced in the canonical HRR pathway, we measured accumulation of the RAD51 recombinase at ionizing radiation (IR)-induced nuclear foci as a surrogate for HRR proficiency (35). Consistent with literature reports, we observed a stark decrease in RAD51 foci formation in the SKOV3 cell lines defective for BRCA2, PALB2, or RAD51C, indicative of inefficient HRR, despite the induction of DNA damage as measured by detection of the phosphorylated form of histone variant H2AX (known as γH2AX; Fig. 2C). Importantly, and in agreement with previous reports (33), RAD51B inactivation resulted in a more modest reduction in RAD51 foci formation both in SKOV3 and DU145 cells despite efficient DNA damage induction (Fig. 2C and D), consistent with the reduced sensitivity of *RAD51B* KO cells to olaparib treatment. Also as reported previously, RAD54L deficiency led to an increase in RAD51 foci formation (Fig. 2D), which has been linked to the impaired removal of RAD51 molecules upon resolution of HRR and/or the increased number of RAD51 presynaptic complexes required to find homology on the donor DNA strand (36, 37).

Collectively, these data provide evidence confirming a similar level of response to olaparib in BRCA2-, PALB2-, and RAD51C-deficient models, a somewhat more modest sensitivity caused by RAD51B or RAD54L deficiencies, and a link between drug response effects to HRR defects in the form of RAD51 foci formation abnormalities.

### *ATM* Loss as Biomarker for Olaparib Sensitization

Our analyses of CRISPR screen data identified *ATM* loss as one of the most recurrent events driving sensitivity to PARPi (Fig. 1C; Supplementary Table S2). Although ATM is not considered a core HRR factor, its deficiency has been linked to sensitivity to PARPi (38, 39) and *ATM* mutations have been explored as patient selection biomarkers in clinical trials assessing the efficacy of PARPi (7, 10, 11). We generated and functionally validated ATM KO cells in DU145 (Supplementary Fig. S3A and S3B) and SKOV3 (Supplementary Fig. S3C and S3D) and compared them with their BRCA-mutant counterparts for their response to olaparib. ATM inactivation caused sensitivity to olaparib in both cell lines; however, the sensitization effect did not reach the levels observed for BRCA mutations (Fig. 2E and F). To confirm these findings, we generated an additional ATM KO in the colorectal carcinoma cell line DLD1 (Supplementary Fig. S3E), for which a BRCA2 KO was already available (40). Importantly, DLD1 ATM KO cells were also sensitive to olaparib but less so than their BRCA2 KO counterpart (Supplementary Fig. S3F). Given the relatively high prevalence of *ATM* mutations in prostate cancer (7), we decided to generate an additional ATM KO cell line in another prostate cancer cell line, LNCAP (Supplementary Fig. S3G), which also resulted in increased sensitivity to olaparib treatment (Supplementary Fig. S3H). Taken together, these results confirm that ATM loss confers sensitivity to PARPi in cell lines from a range of different tumor types.

### Clinical Prevalence of Mutations Identifies *XRCC3* Loss in Prostate Cancer

**FIGURE 3:**
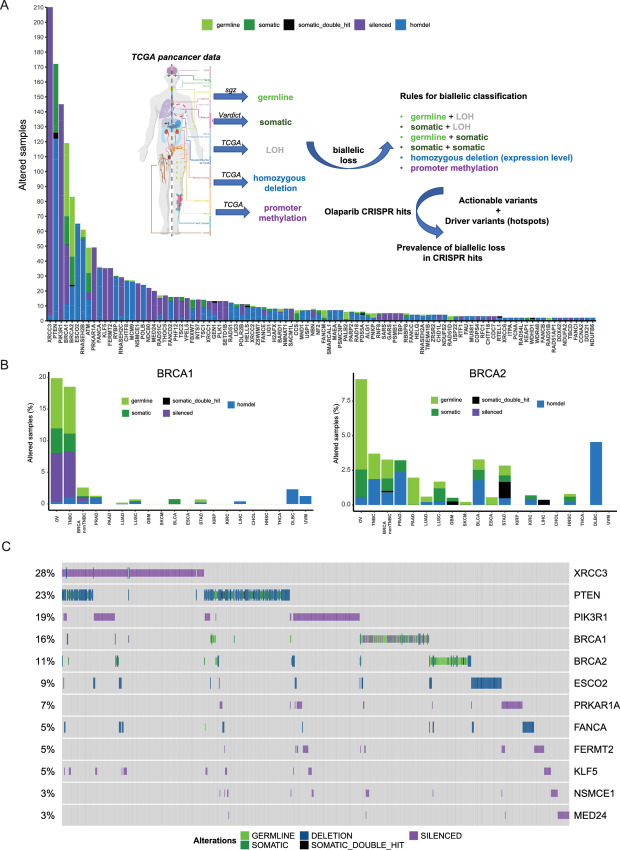
Analyses of genes identified through CRISPR/Cas9 screening in tumor datasets. **A,** Analysis of the total number of samples showing biallelic loss of the 110 confidence genes identified through CRISPR screens in tumor types where PARPi are currently approved (ovarian, breast, pancreas, prostate). The inset shows the pipeline design (see also Materials and Methods). **B,** Frequency of biallelic inactivation events for *BRCA1* (left) and *BRCA2* (right) across the different tumor types analyzed. Ovarian (OV), triple-negative breast cancer (TNBC), prostate adenocarcinoma (PRAD), pancreatic adenocarcinoma (PAAD), lung adenocarcinoma (LUAD), lung squamous cell carcinoma (LUSC), glioblastoma (GBM), bladder urothelial carcinoma (BLCA), esophageal carcinoma (ESCA), stomach adenocarcinoma (STAD), kidney renal papillary cell carcinoma (KIRP), kidney renal clear cell carcinoma (KIRC), liver hepatocellular carcinoma (LIHC), cholangiocarcinoma (CHOL), head and neck squamous cell carcinoma (HNSC), thyroid carcinoma (THCA), diffuse large B-cell lymphoma (DLBCL), uveolar melanoma (UVM). **C,** Oncoprint of the top 12 genes showing biallelic LoF in the subset of tumor types where PARPi are a treatment option (ovary, breast, pancreas, prostate). Each bar represents an individual tumor. Percentages are for the number of altered samples in the whole dataset.

CRISPR LoF screens explore, by definition, responses in a setting where the function of any given gene is completely or almost completely abrogated. As such, we created an analysis pipeline of tumor pan-cancer patient data from TCGA to explore the prevalence of biallelic LoF events (including deleterious somatic and germline mutations, homozygous deletions and promoter hypermethylation) in each of the 110 confidence genes in our dataset in the subset of tumor types where PARPi are already available treatment options (ovary, breast, prostate, and pancreas; Fig. 3A; Supplementary Table S3 and Materials and Methods). Importantly, our analyses identified biallelic LoF of *BRCA1* or *BRCA2* in 20% and 9% of ovarian cancer samples, respectively, in line with what has been reported previously (Fig. 3B; refs. 41, 42).

**FIGURE 4:**
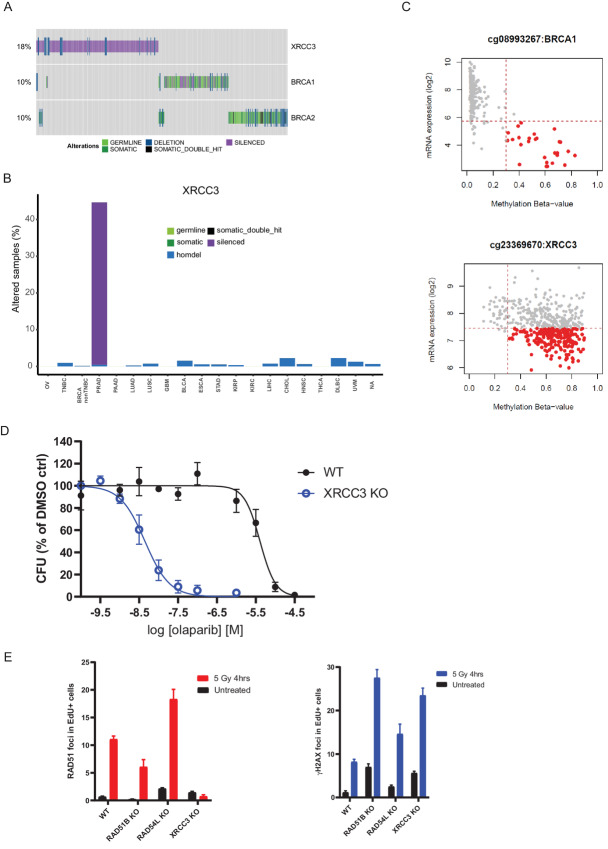
*XRCC3* gene silencing as potential biomarker of olaparib sensitivity in prostate cancer. **A,** Oncoprint depicting mutual exclusivity between LoF of *XRCC3, BRCA1,* and *BRCA2* in the pan-cancer analysis. Each bar represents an individual tumor. Percentages indicate the number of altered samples in the whole dataset. **B,***XRCC3* biallelic inactivation is primarily driven by gene silencing in prostate cancer. Tumor types are the same as listed in Fig. 3. **C,** Correlation between *BRCA1* (top) and *XRCC3* (bottom) mRNA expression and promoter methylation scores. Highlighted in red are samples with high methylation scores and low mRNA expression values. **D,** Dose–response curves for DU145 isogenic *XRCC3* KO cells treated with olaparib for 10–15 days in clonogenic assays. Results are shown as mean of *n* = 3 biological replicates ± SD. **E,** Quantification of RAD51 and γH2AX foci by immunofluorescence in DU145 isogenic pairs 4 hours after treatment with 5 Gy of ionizing radiation or in untreated cells. Results are shown as mean number of foci per nucleus in replicating (EdU-positive) cells (*n* = 3 biological replicates ± SD). Data for WT, *RAD51B*, and *RAD54L* KO are the same as shown in Fig. 2C.

Interestingly, from the list of 110 confidence genes, *XRCC3* was the most frequently altered gene in our analyses specifically in PARPi-approved settings (Fig. 3A and C). XRCC3 is one of the five RAD51 paralogs encoded in the human genome (the others being RAD51B, RAD51C, RAD51D, and XRCC2) and its function in HRR is well described (33). Accordingly, statistical analyses of the most altered genes in our dataset showed that LoF of *XRCC3* is mutually exclusive with either *BRCA1* (Fisher exact *P* value 0.002) or *BRCA2* (Fisher exact *P* value 0.01) LoF when considering the pan-cancer dataset (Supplementary Fig. S4A and S4B), an effect that becomes even more significant when limiting the analysis to ovarian, breast, pancreatic, and prostate tumors (*BRCA1 P* value 4.1E-07; *BRCA2 P* value 6.4E-04; Fig. 4A).

Although there was some level of homozygous deletions adding to the *XRCC3* LoF events in our dataset, it was promoter methylation in prostate cancer samples what drove their prevalence (Fig. 4B and C; Supplementary Table S3). As a way to mimic this *XRCC3* LoF state, we generated *XRCC3* KO in the DU145 prostate cancer cell line (Supplementary Fig. S4C). Strikingly, XRCC3 loss conferred a high level of sensitivity to olaparib in DU145 cells (Fig. 4D), which correlated with a stark reduction in the ability of these cells to form RAD51 foci upon IR treatment with no impact on γH2AX foci formation (Fig. 4E). Taken together, our data confirm XRCC3 loss as a bona fide marker of PARPi sensitivity with potential clinical relevance, especially in the prostate cancer setting.

## Discussion

Although a number of functional genomics screens have been performed to explore the genetic determinants of PARPi sensitivity in different cellular models, this study is the first one to try to put the identified biomarkers of sensitivity in a context of prevalence based on available tumor genetic data. We aimed to achieve this goal in two ways. First, by generating head-to-head olaparib sensitivity data on a set of genes identified in the screens, mutations of which have been explored as patient selection biomarkers in PARPi clinical trials (5, 7, 10, 11) and that are involved in different aspects of DNA repair biology, especially HRR. This comparison has been importantly done for the first time in tissue-type relevant cell models (ovary and prostate) with the appropriate clinically approved benchmark controls for PARPi sensitivity (BRCA1 or BRCA2 deficiency). Together with BRCA1 and BRCA2, deficiency in PALB2 or RAD51C caused the most significant increases in sensitivity to olaparib in these *in vitro* models (Supplementary Fig. S5), highlighting the key role these proteins perform in HRR and suggesting that inactivating mutations in *PALB2* or *RAD51C* could be considered as equivalent to *BRCA* mutations with regards to their response to PARPi treatment.

Inactivation of a second group of genes, consisting of *ATM, RAD51B,* and *RAD54L*, generated intermediate sensitivity profiles to olaparib treatment when compared with *BRCA1* or *BRCA2* loss in the relevant cell models (Supplementary Fig. S5). Our data with *RAD51B* are consistent with published literature (33) and our results with *ATM* are in agreement with the differential responses observed in patients with prostate cancer harboring tumors with mutations in *ATM* treated with olaparib when compared with those carrying *BRCA2* mutations (7). Given that we observed a reduced impact on HRR proficiency (as measured by RAD51 foci formation) in RAD51B- or RAD54L-deficient cells compared with those lacking BRCA2, PALB2, or RAD51C (Fig. 3C and D) and that ATM does not play a central role in HRR (39, 42), these data suggest that significant impairment of HRR is required to elicit a BRCA-like response to olaparib. Notwithstanding, our data support the inclusion of mutations in these genes as prospective patient selection biomarkers for PARPi treatment, as highlighted in recent prostate cancer clinical trials (7, 10, 11). For the specific case of ATM, and given the synthetic lethal interaction described between ATM loss and inactivation of the other DNA-damage response apical kinases ATR or DNA-PK (43), it is possible that combination of PARPi with ATR or DNA-PK inhibitors could result in better responses than any single-agent approach (44, 45).

Our second attempt to provide a more relevant clinical context to the genes identified as potential biomarkers of PARPi response through CRISPR screening was to develop an analysis pipeline assessing the prevalence of biallelic LoF events in a wide range of tumor types. It was surprising that our analysis identified LoF of *XRCC3* as the most prevalent in PARPi-treated tumor types among the 110 confidence genes, mostly driven by gene silencing caused by promoter methylation in prostate cancer. As expected given the known role of XRCC3 in HRR, its inactivation is mutually exclusive with loss of BRCA1 or BRCA2. In that regard, it is important to highlight that no mutual exclusivity was identified between LoF of bona fide HRR genes such as *BRCA1* or *BRCA2* and genes like *RNASEH2B* or *POLB*. Interestingly, however, a degree of co-occurrence was detected between LoF of the cohesin factor gene *ESCO2* and the BER gene *POLB*, and between *BRCA2* and *RNASEH2B* (Supplementary Fig. S3B). It will be interesting to explore whether combinations of these biomarkers could drive better PARPi responses in the relevant tumor types.

XRCC3 forms a functional complex (the CX3 complex) with another RAD51 paralog, RAD51C, whose LoF events are also mainly caused by gene silencing, in this case in breast cancer samples (Supplementary Table S3). RAD51C forms another protein complex (the BCDX2 complex) with other RAD51 paralogs (RAD51B, RAD51D, and XRCC2) that does not include XRCC3 (33). Given that XRCC3 deficiency causes a high level of sensitivity to olaparib in the DU145 prostate cancer cell line, and that RAD51C loss phenocopies PARPi sensitivity caused by BRCA2 loss in the ovarian cell line SKOV3, while the same is not true for the other BCDX2 complex component RAD51B, it is tempting to speculate that there is a more important function of the CX3 RAD51 paralog complex in mediating PARPi responses. Whatever the case, the identification of *XRCC3* silencing as an important LoF event in prostate cancer opens the possibility to explore its clinical relevance in a tumor type where olaparib is already a treatment option. Retrospective analysis of tumor material linked to patient outcome in ongoing prostate cancer clinical trials (46, 47), once available, could definitely shed light into the importance of this newly identified biomarker.

## Supplementary Material

Supplementary Figures S1-S5 Supplementary Figure S1. A, Workflow to generate isogenic HRR KO cell models (see also Methods). B, Dose-response curve for SKOV3 BRCA2 KO isogenic pairs treated with olaparib for 10-14 days in clonogenic survival assays. Results are shown as mean of n=4 biological replicates ± SD for the dose-response curves. Clone 13 was selected for further experimentation. C, Fold-change mRNA expression in WT and KO cells to assess BRCA2 loss in SKOV3 cells. D, Dose-response curve for DU145 BRCA1 KO isogenic pairs treated with olaparib for 10-14 days in clonogenic survival assays. Results are shown as mean of n=2 biological replicates ± SD for the dose-response curves. Clone A1 was selected for further experimentation. E, Western blot data to assess BRCA1 loss in DU145 cells (clone A1). Supplementary Figure S2. A, Dose-response curve for SKOV3 PALB2 KO isogenic pairs treated with olaparib for 10-14 days in clonogenic survival assays. Results are shown as mean of n=4 biological replicates ± SD for the dose-response curves. Clone 19 was selected for further experimentation. B, Western blot data to assess PALB2 loss in SKOV3 cells (clone 19). C, Dose-response curve for SKOV3 RAD51C KO clone 3 isogenic pairs treated with olaparib for 10-14 days in clonogenic survival assays. Results are shown as mean of n=3 biological replicates ± SD for the dose-response curves. D, Western blot data to assess RAD51C loss in SKOV3 cells (clone C7 was selected for further experimentation and its dose-response curve is shown in Figure 2A). E, Dose-response curve for SKOV3 RAD51B KO isogenic pairs treated with olaparib for 10-14 days in clonogenic survival assays. Results are shown as mean of n=4 biological replicates ± SD for the dose-response curves. Clone 4 was selected for further experimentation. F-H, Fold-change mRNA expression in WT and KO cells to assess RAD51B loss in SKOV3 cells (clone 4) (F) and DU145 cells (G), and loss of RAD54L in DU145 cells (H). Supplementary Figure S3. A-E and G, Western blots to assess ATM loss (A, C) and ATM signalling (B, D, E, G) 4 h after treating cells with 5 Gy of ionizing radiation (IR) in the presence or absence of ATM inhibitor (AZD0156, 100 nM) added 2 h before treatment in DU145 (B), SKOV3 (D), DLD1 (E) and LNCAP (G) ATM KO isogenic cell lines. F and H, Dose-response curves for ATM KO DLD1 clones (F) and LNCAP (H) isogenic pairs treated with olaparib for 10-14 days in clonogenic survival assays. Results are shown as mean of n=4 biological replicates ± SD for the dose-response curves. Clonogenic survival data on (H) was generated with LNCAP ATM KO2 cells (see panel G). Clone 5 of the DLD1 ATM KO cells was selected for further experimentation. Supplementary Figure S4. A, Pan-cancer analysis of the total number of samples showing biallelic loss of the 110 confidence genes identified through CRISPR screens. B, Oncoprint of the top 10 genes showing biallelic LoF in the pan-cancer analysis. Each bar represents an individual tumour. Percentages are for the number of altered samples in the whole dataset. C, Inference of CRISPR Edits (ICE) analyses of the edited region of the XRCC3 gene in WT (bottom) or XRCC3 KO (top) DU145 cells. ICE quantification reported an overall editing efficiency of 98% in the KO cells, with a 1 bp deletion accounting for 22% of the events and a 1 bp insertion accounting for the remaining 77%. Supplementary Figure S5. Half-maximal inhibitory concentration (IC50) for olaparib in the different isogenic cell lines used in this study as measured in clonogenic survival assays. Values were calculated from the dose-response curves in Figures 1, 2 and 4 and Supplementary Figure S3.

Supplementary Table S1 CRISPR-Cas9 loss-of-function screens analysed in this study.

Supplementary Table S2 List of 110 confidence genes from CRISPR-Cas9 loss-of-function screens.

Supplementary Table S3 Breakdown of biallelic losses (as percentages of total number of samples analysed) detected for each of the 110 confidence genes identified through CRISPR screens in all the tumour types analysed.
