# Supplementary material for: Drug–gene Interaction Screens Coupled to Tumor Data Analyses Identify the Most Clinically Relevant Cancer Vulnerabilities Driving Sensitivity to PARP Inhibition
Source: Cancer Res Commun. 2022 Oct 21;2(10):1244–54. doi: 10.1158/2767-9764.CRC-22-0119 (PMC10035383; doi:10.1158/2767-9764.CRC-22-0119)
Supplement: Supplementary Figures S1-S5 — Supplementary Figure S1. A, Workflow to generate isogenic HRR KO cell models (see also Methods). B, Dose-response curve for SKOV3 BRCA2 KO isogenic pairs treated with olaparib for 10-14 days in clonogenic survival assays. Results are shown as mean of n=4 biological replicates ± SD for the dose-response curves. Clone 13 was selected for further experimentation. C, Fold-change mRNA expression in WT and KO cells to assess BRCA2 loss in SKOV3 cells. D, Dose-response curve for DU145 BRCA1 KO isogenic pairs treated with olaparib for 10-14 days in clonogenic survival assays. Results are shown as mean of n=2 biological replicates ± SD for the dose-response curves. Clone A1 was selected for further experimentation. E, Western blot data to assess BRCA1 loss in DU145 cells (clone A1). Supplementary Figure S2. A, Dose-response curve for SKOV3 PALB2 KO isogenic pairs treated with olaparib for 10-14 days in clonogenic survival assays. Results are shown as mean of n=4 biological replicates ± SD for the dose-response curves. Clone 19 was selected for further experimentation. B, Western blot data to assess PALB2 loss in SKOV3 cells (clone 19). C, Dose-response curve for SKOV3 RAD51C KO clone 3 isogenic pairs treated with olaparib for 10-14 days in clonogenic survival assays. Results are shown as mean of n=3 biological replicates ± SD for the dose-response curves. D, Western blot data to assess RAD51C loss in SKOV3 cells (clone C7 was selected for further experimentation and its dose-response curve is shown in Figure 2A). E, Dose-response curve for SKOV3 RAD51B KO isogenic pairs treated with olaparib for 10-14 days in clonogenic survival assays. Results are shown as mean of n=4 biological replicates ± SD for the dose-response curves. Clone 4 was selected for further experimentation. F-H, Fold-change mRNA expression in WT and KO cells to assess RAD51B loss in SKOV3 cells (clone 4) (F) and DU145 cells (G), and loss of RAD54L in DU145 cells (H). Supplementary Figure S3. A-E and G, Weste [file crc-22-0119-s01.pdf]

Supplementary Figure 1

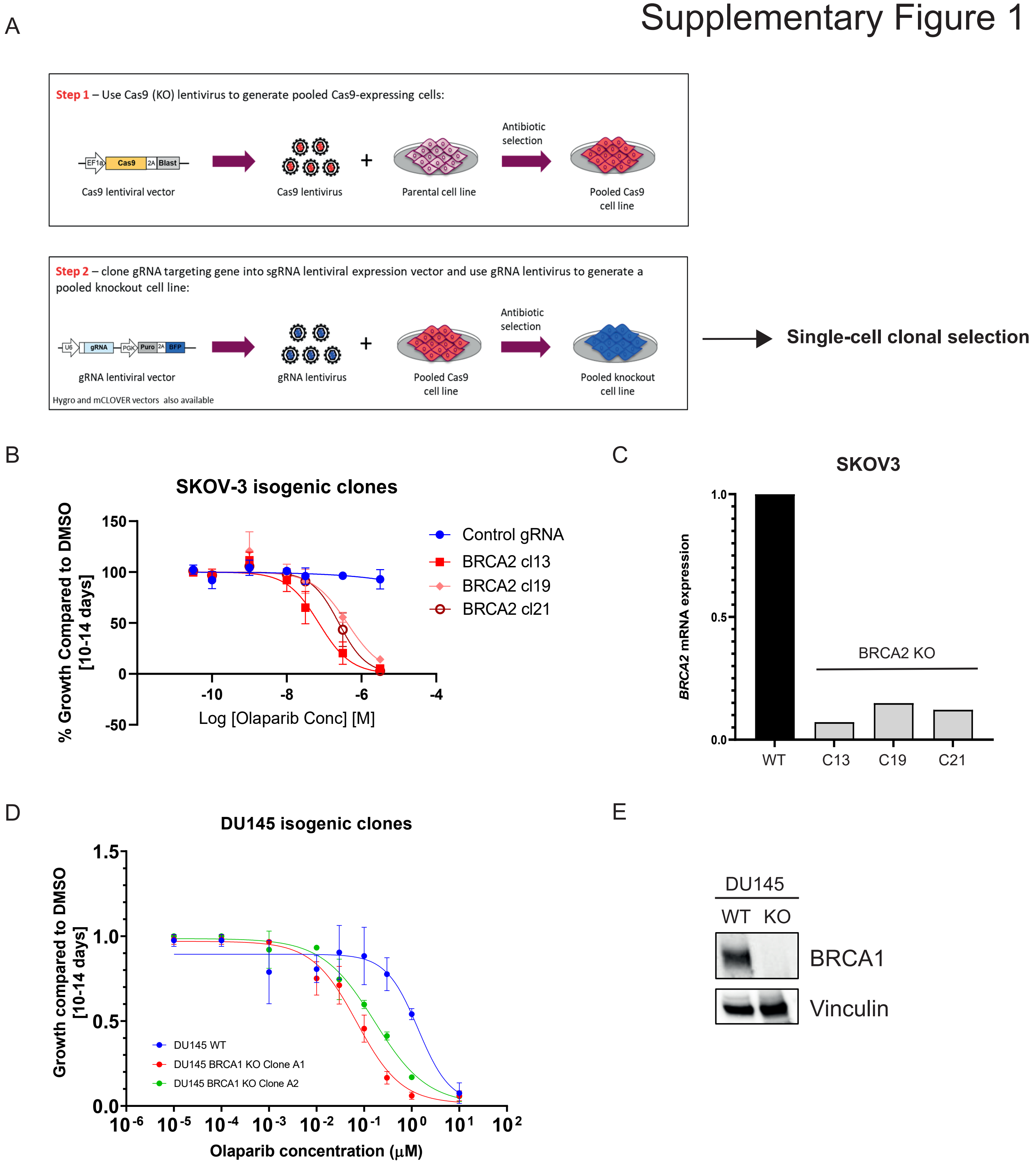

Supplementary Figure S1. A, Workflow to generate isogenic HRR KO cell models (see also Methods). **B,** Dose-response curve for SKOV3 BRCA2 KO isogenic pairs treated with olaparib for 10-14 days in clonogenic survival assays. Results are shown as mean of n=4 biological replicates ± SD for the dose-response curves. Clone 13 was selected for further experimentation. **C,** Fold-change mRNA expression in WT and KO cells to assess BRCA2 loss in SKOV3 cells. **D,** Dose-response curve for DU145 BRCA1 KO isogenic pairs treated with olaparib for 10-14 days in clonogenic survival assays. Results are shown as mean of n=2 biological replicates ± SD for the dose-response curves. Clone A1 was selected for further experimentation. **E,** Western blot data to assess BRCA1 loss in DU145 cells (clone A1).

Supplementary Figure 2

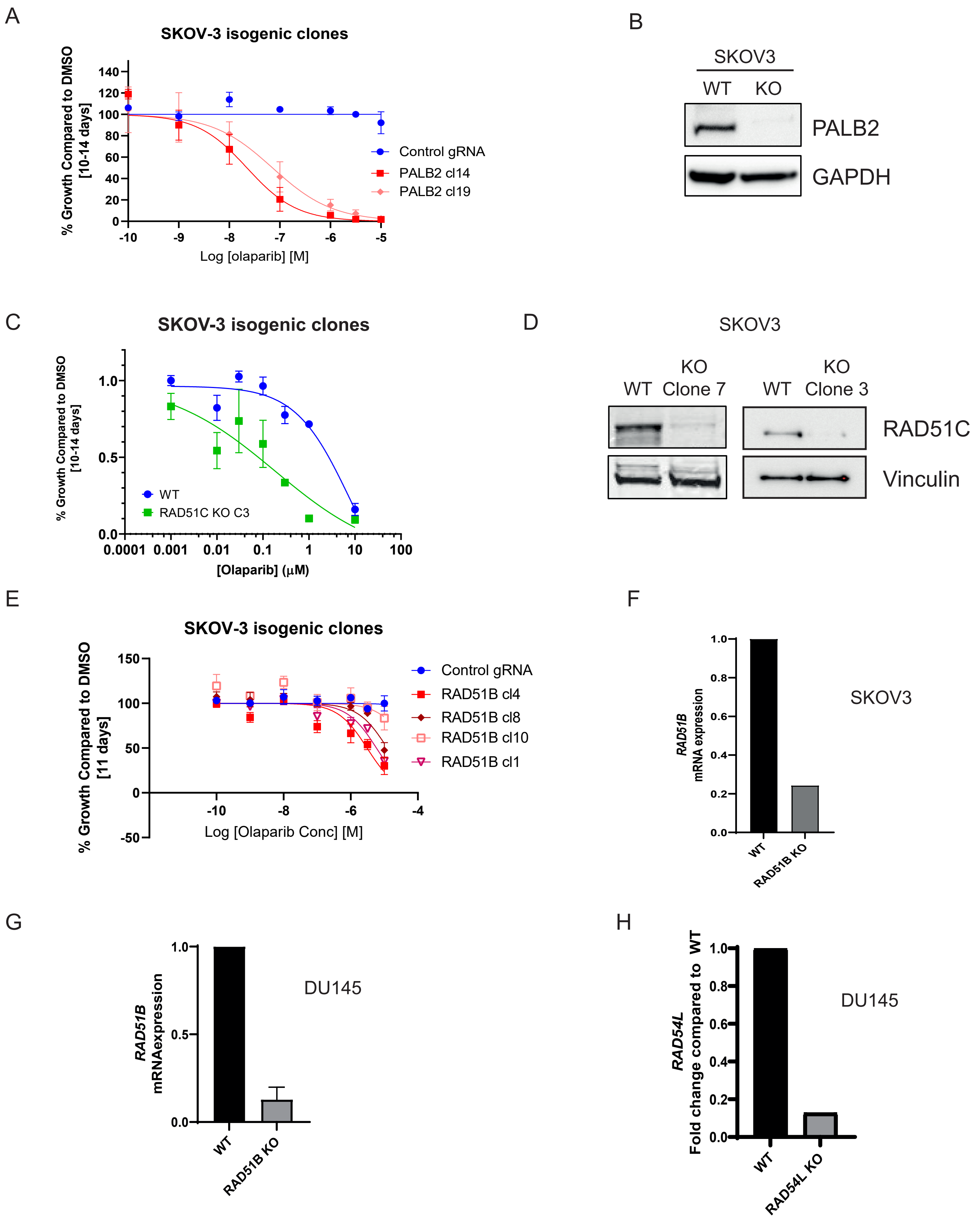

Supplementary Figure S2. A, Dose-response curve for SKOV3 PALB2 KO isogenic pairs treated with olaparib for 10-14 days in clonogenic survival assays. Results are shown as mean of n=4 biological replicates ± SD for the dose-response curves. Clone 19 was selected for further experimentation. **B,** Western blot data to assess PALB2 loss in SKOV3 cells (clone 19). **C,** Dose-response curve for SKOV3 RAD51C KO clone 3 isogenic pairs treated with olaparib for 10-14 days in clonogenic survival assays. Results are shown as mean of n=3 biological replicates ± SD for the dose-response curves. **D,** Western blot data to assess RAD51C loss in SKOV3 cells (clone C7 was selected for further experimentation and its dose-response curve is shown in Figure 2A). **E,** Dose-response curve for SKOV3 RAD51B KO isogenic pairs treated with olaparib for 10-14 days in clonogenic survival assays. Results are shown as mean of n=4 biological replicates ± SD for the dose-response curves. Clone 4 was selected for further experimentation. **F-H,** Fold-change mRNA expression in WT and KO cells to assess RAD51B loss in SKOV3 cells (clone 4) (F) and DU145 cells (G), and loss of RAD54L in DU145 cells (H).

Supplementary Figure 3

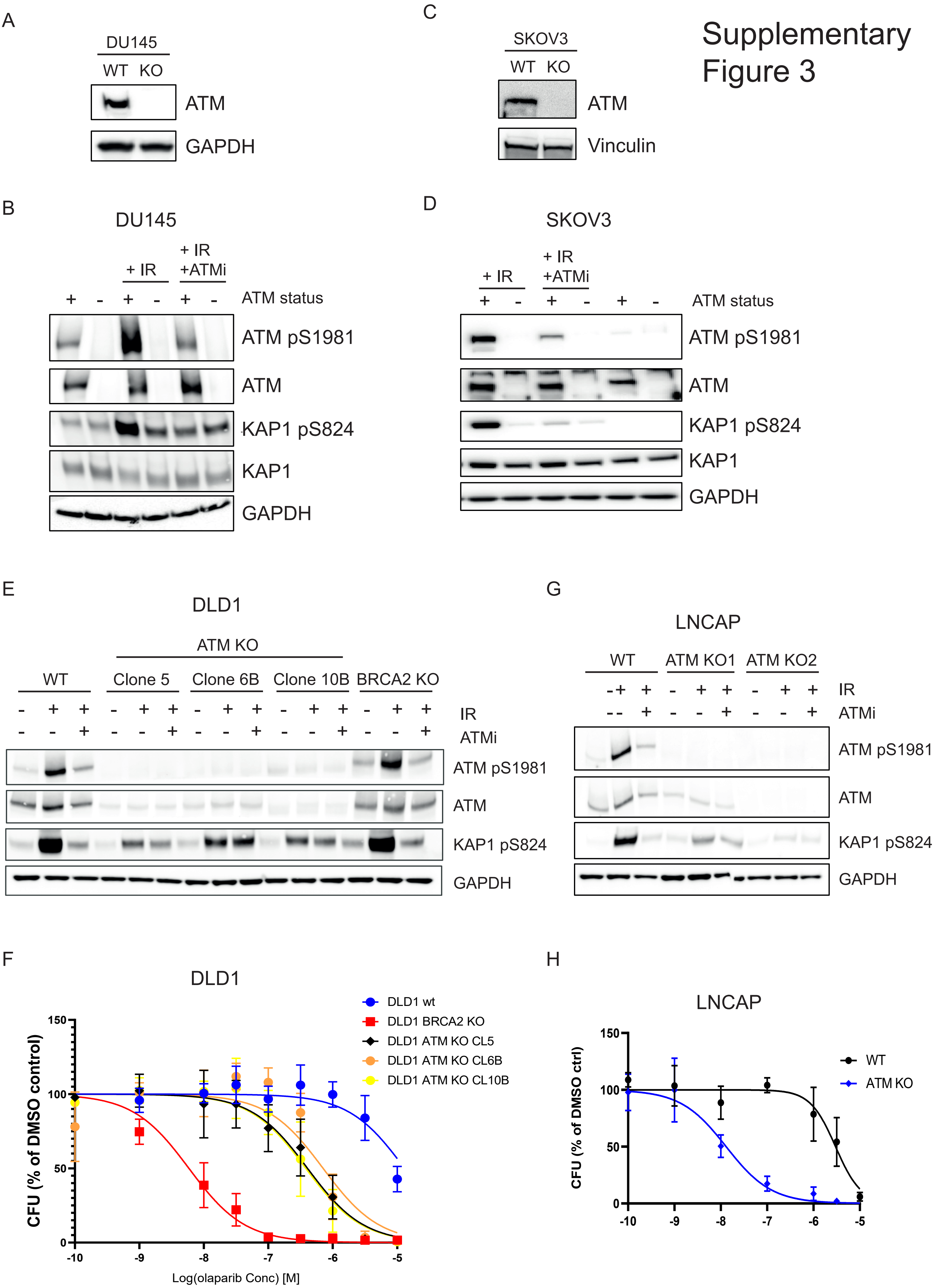

Supplementary Figure S3. A-E and G, Western blots to assess ATM loss (A, C) and ATM signalling (B, D, E, G) 4 h after treating cells with 5 Gy of ionizing radiation (IR) in the presence or absence of ATM inhibitor (AZD0156, 100 nM) added 2 h before treatment in DU145 (B), SKOV3 (D), DLD1 (E) and LNCAP (G) *ATM* KO isogenic cell lines. **F and H**, Dose-response curves for ATM KO DLD1 clones (F) and LNCAP (H) isogenic pairs treated with olaparib for 10-14 days in clonogenic survival assays. Results are shown as mean of n=4 biological replicates ± SD for the dose-response curves. Clonogenic survival data on (H) was generated with LNCAP ATM KO2 cells (see panel G). Clone 5 of the DLD1 ATM KO cells was selected for further experimentation.

Supplementary Figure 4

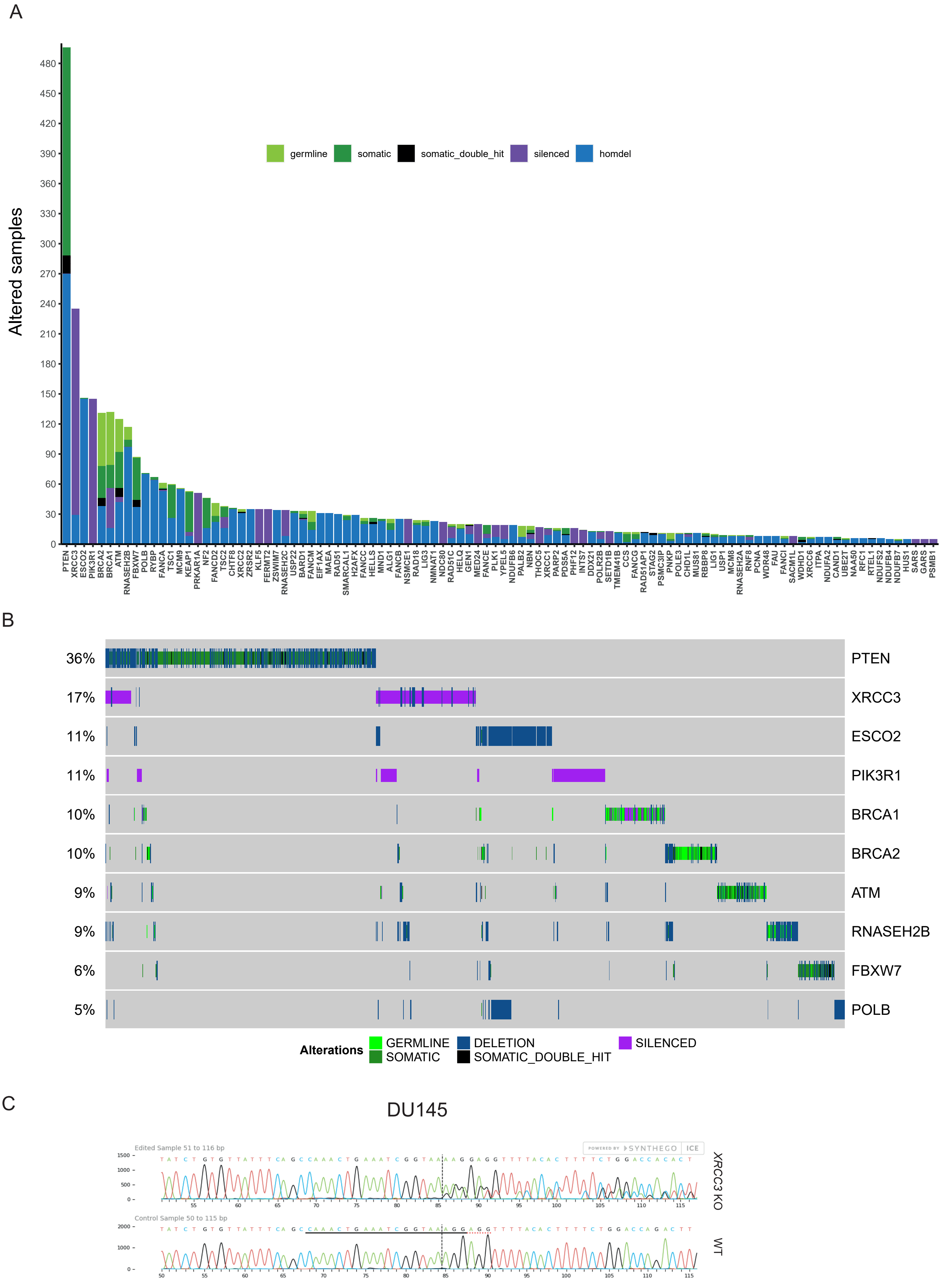

Supplementary Figure S4. A, Pan-cancer analysis of the total number of samples showing biallelic loss of the 110 confidence genes identified through CRISPR screens. **B,** Oncoprint of the top 10 genes showing biallelic LoF in the pan-cancer analysis. Each bar represents an individual tumour. Percentages are for the number of altered samples in the whole dataset. **C,** Inference of CRISPR Edits (ICE) analyses of the edited region of the *XRCC3* gene in WT (bottom) or *XRCC3* KO (top) DU145 cells. ICE quantification reported an overall editing efficiency of 98% in the KO cells, with a 1 bp deletion accounting for 22% of the events and a 1 bp insertion accounting for the remaining 77%.

Supplementary Figure 5

	**Cell line olaparib IC50 (μM)**		
**Genotype**	SKOV3	DU145	DLD1
Parental	> 10	8	> 10
ATM KO	1	0.45	0.381
BRCA1 KO		0.067	
BRCA2 KO	0.051		0.006
PALB2 KO	0.0223		
RAD51B KO	3	1.8	
RAD51C KO	0.04		
RAD54L KO		1.6	
XRCC3 KO		0.007	

Supplementary Figure S5. Half-maximal inhibitory concentration (IC50) for olaparib in the different isogenic cell lines used in this study as measured in clonogenic survival assays. Values were calculated from the dose-response curves in Figures 1, 2 and 4 and Supplementary Figure S3.
