# Supplementary material for: Drug–gene Interaction Screens Coupled to Tumor Data Analyses Identify the Most Clinically Relevant Cancer Vulnerabilities Driving Sensitivity to PARP Inhibition
Source: Cancer Res Commun. 2022 Oct 21;2(10):1244–54. doi: 10.1158/2767-9764.CRC-22-0119 (PMC10035383; doi:10.1158/2767-9764.CRC-22-0119)
Supplement: Supplementary Table S3 — Breakdown of biallelic losses (as percentages of total number of samples analysed) detected for each of the 110 confidence genes identified through CRISPR screens in all the tumour types analysed. [file crc-22-0119-s04.pdf]

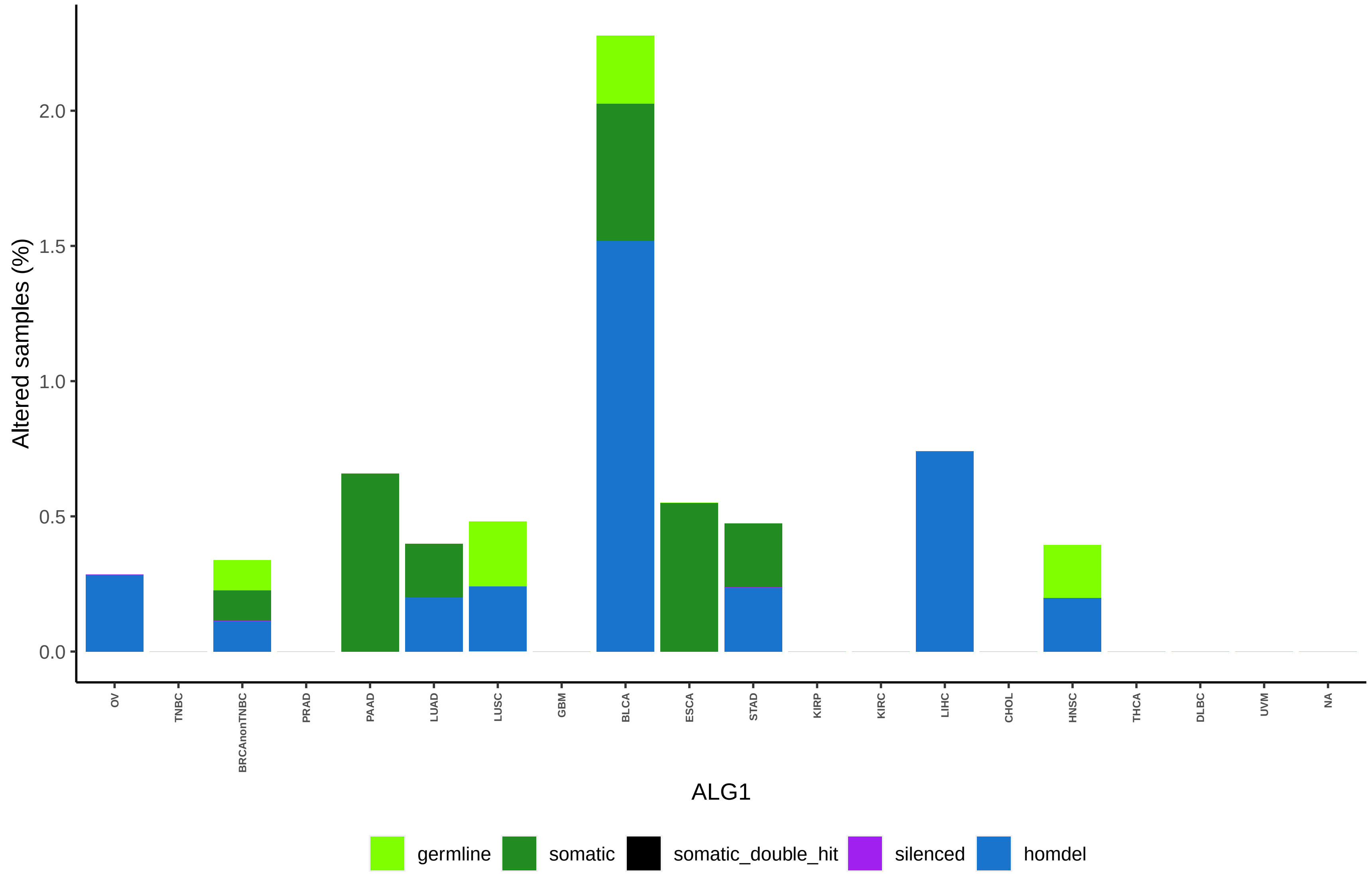
Altered samples (%)
0.0
0.5
1.0
1.5
2.0
OV TNBC BRCAnonTNBC PRAD PAAD LUAD LUSC GBM BLCA ESCA STAD KIRP KIRC LIHC CHOL HNSC THCA DLBC UVM NA
ALG1
germline somatic somatic_double_hit silenced homdel

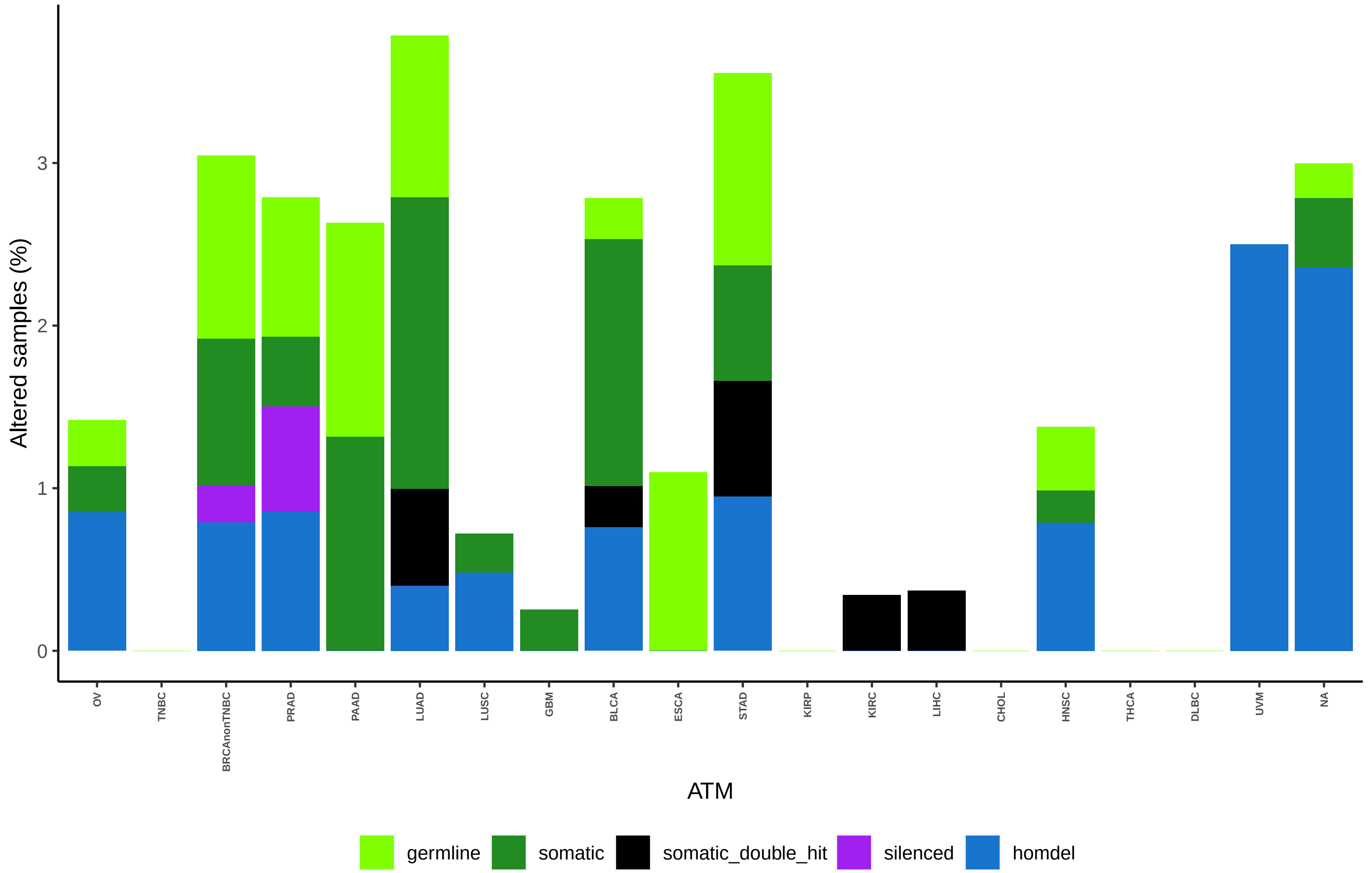
Altered samples (%)
0
1
2
3
OV TNBC BRCAnonTNBC PRAD PAAD LUAD LUSC GBM BLCA ESCA STAD KIRP KIRC LIHC CHOL HNSC THCA DLBC UVM NA
ATM
germline somatic somatic_double_hit silenced homdel

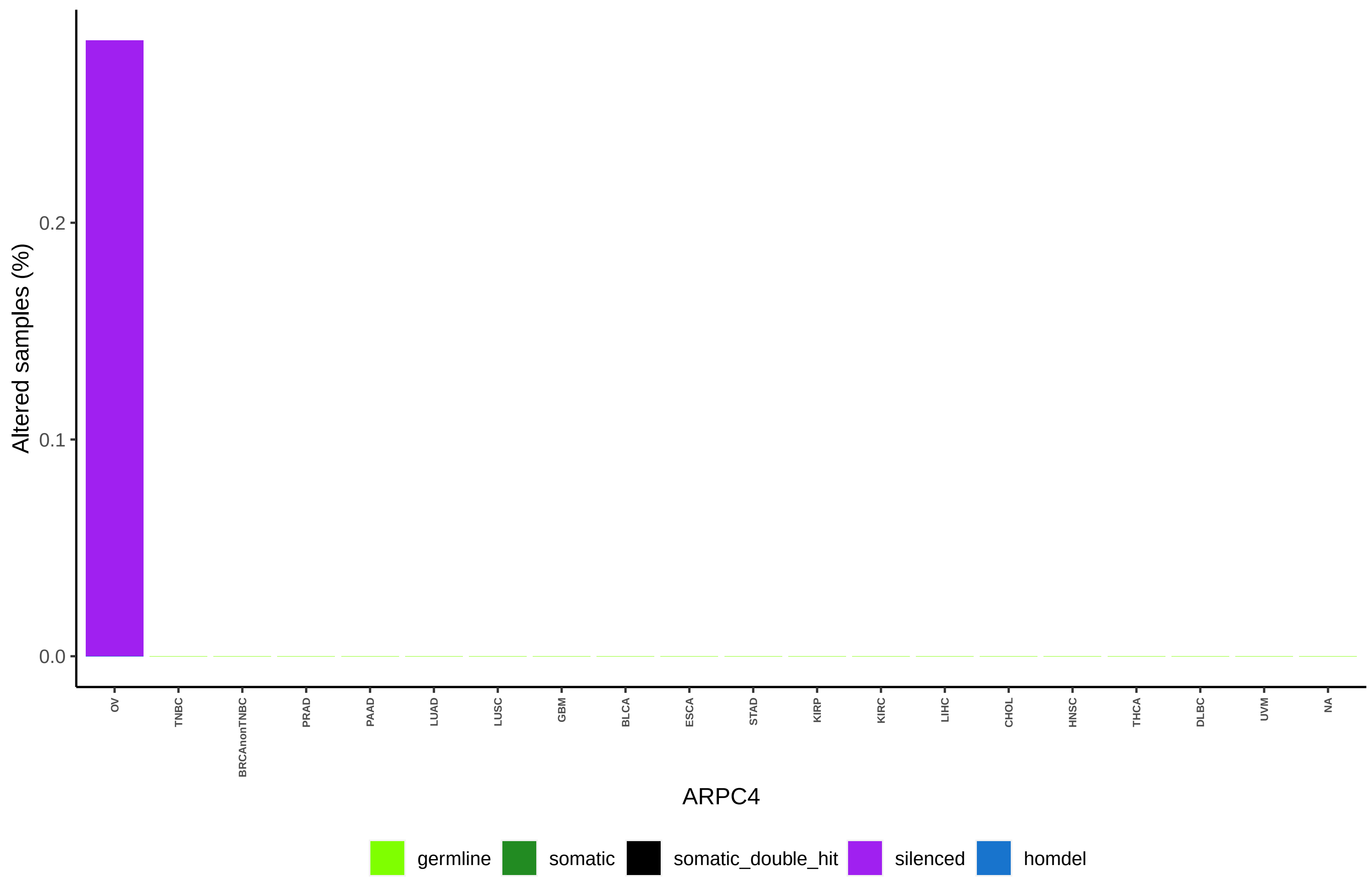
Altered samples (%)
0.0
0.1
0.2
OV TNBC BRCAnonTNBC PRAD PAAD LUAD LUSC GBM BLCA ESCA STAD KIRP KIRC LIHC CHOL HNSC THCA DLBC UVM NA
ARPC4
germline somatic somatic_double_hit silenced homdel

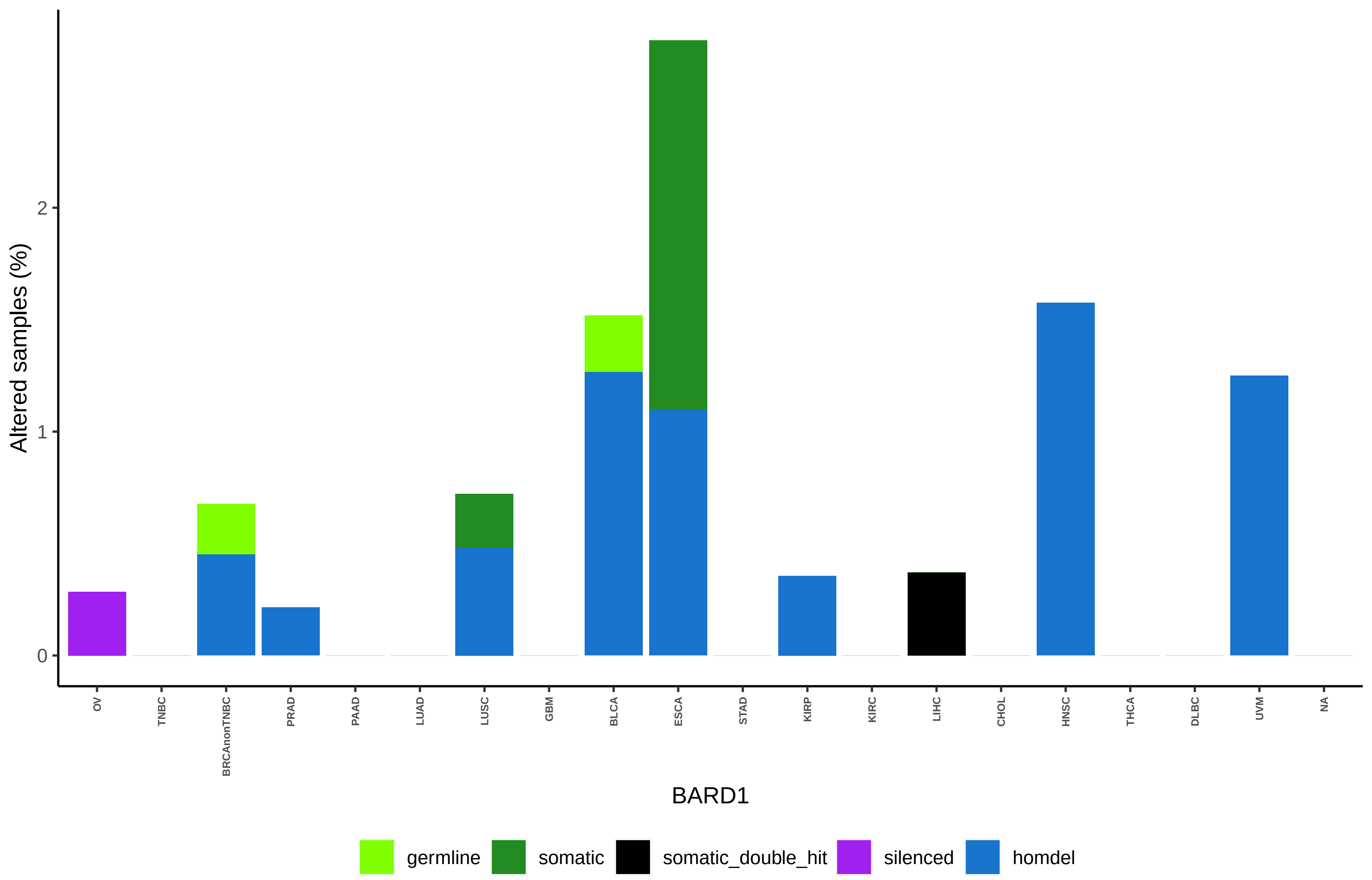
Altered samples (%)
0
1
2
OV TNBC BRCAnonTNBC PRAD PAAD LUAD LUSC GBM BLCA ESCA STAD KIRP KIRC LIHC CHOL HNSC THCA DLBC UVM NA
BARD1
germline somatic somatic_double_hit silenced homdel

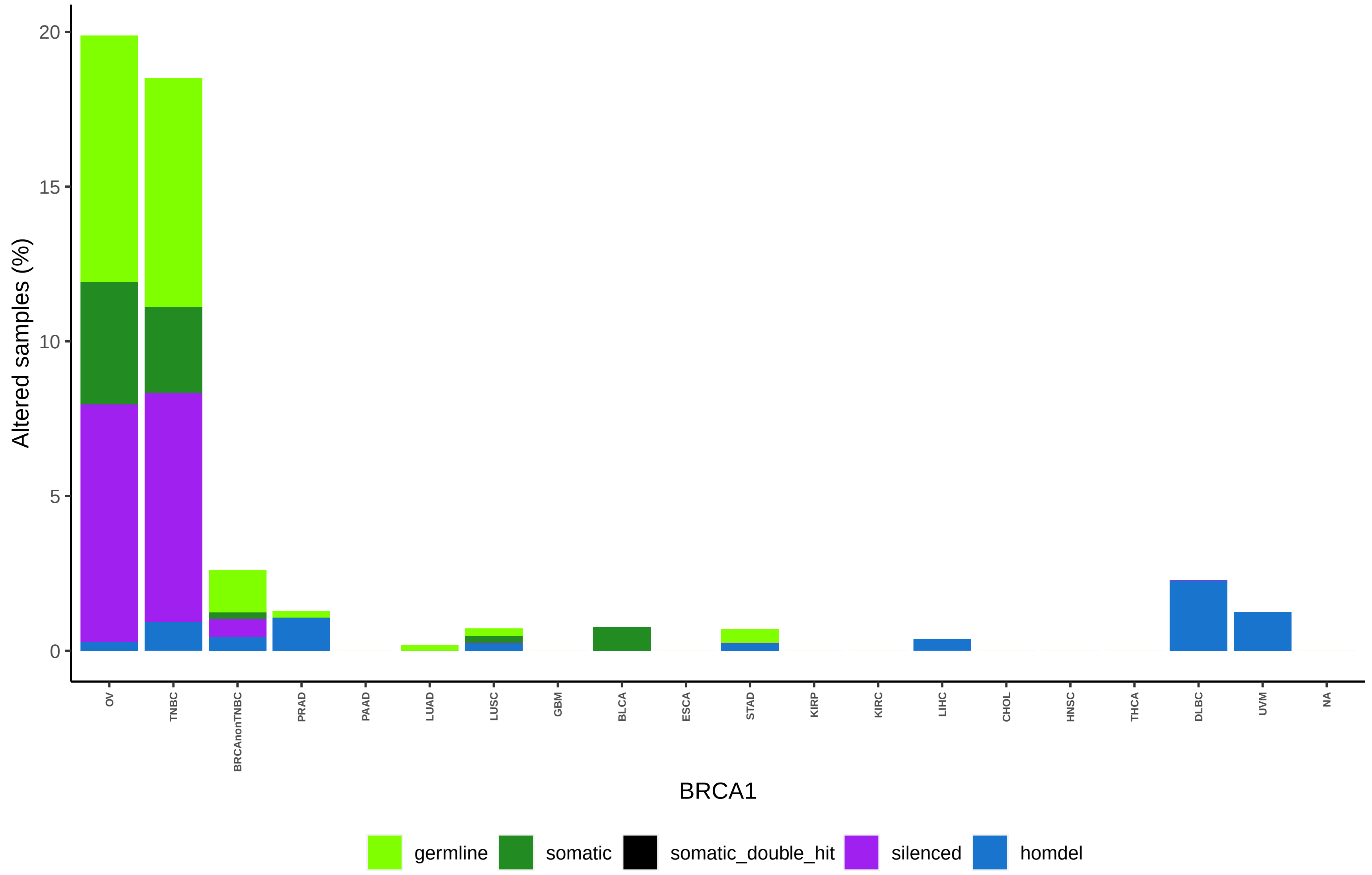
Altered samples (%)
0
5
10
15
20
OV
TNBC
BRCAnonTNBC
PRAD
PAAD
LUAD
LUSC
GBM
BLCA
ESCA
STAD
KIRP
KIRC
LIHC
CHOL
HNSC
THCA
DLBC
UVM
NA
BRCA1
germline
somatic
somatic_double_hit
silenced
homdel

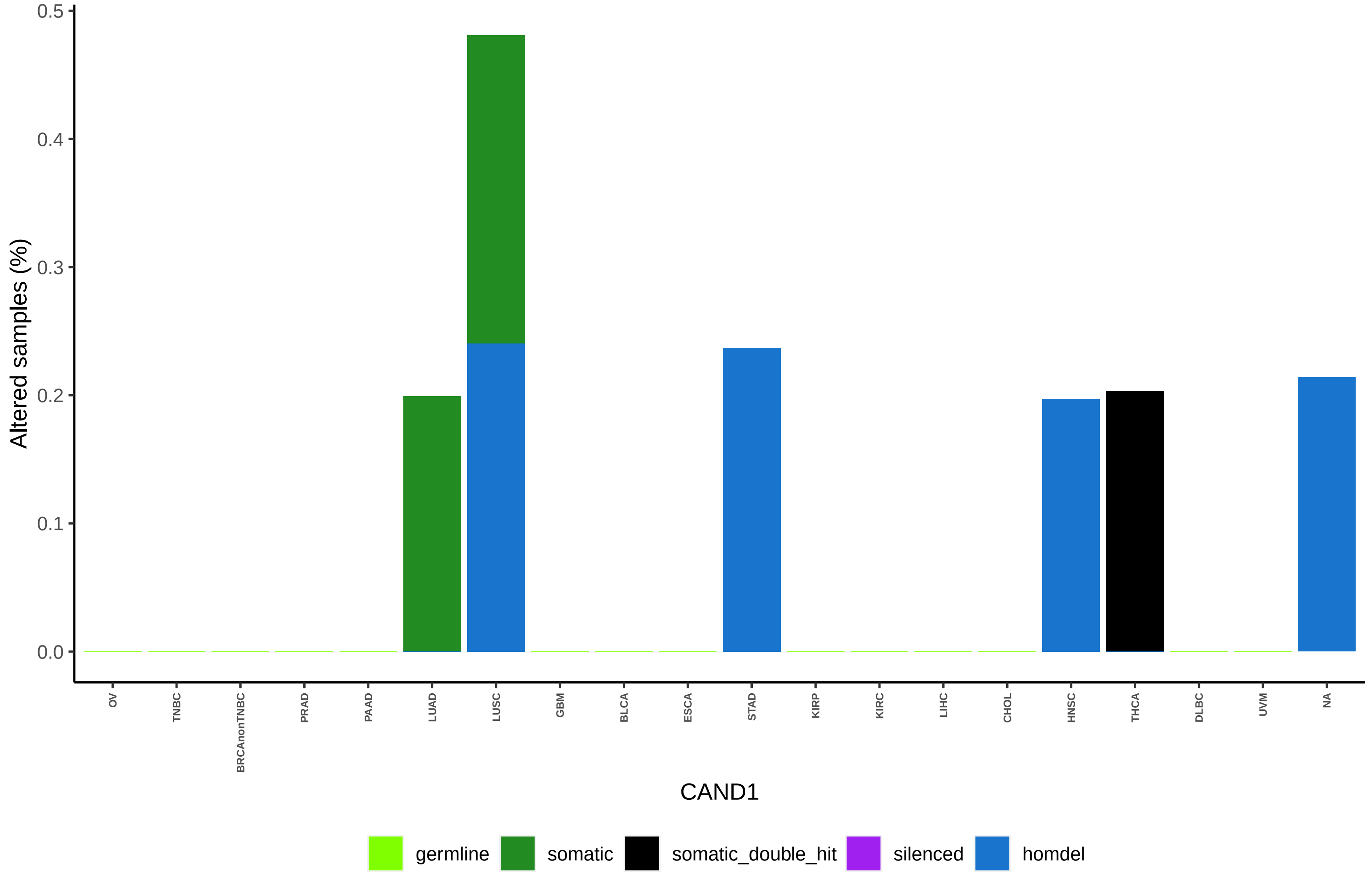
Altered samples (%)
0.0
0.1
0.2
0.3
0.4
0.5
OV
TNBC
BRCAnonTNBC
PRAD
PAAD
LUAD
LUSC
GBM
BLCA
ESCA
STAD
KIRP
KIRC
LIHC
CHOL
HNSC
THCA
DLBC
UVM
NA
CAND1
germline
somatic
somatic_double_hit
silenced
homdel

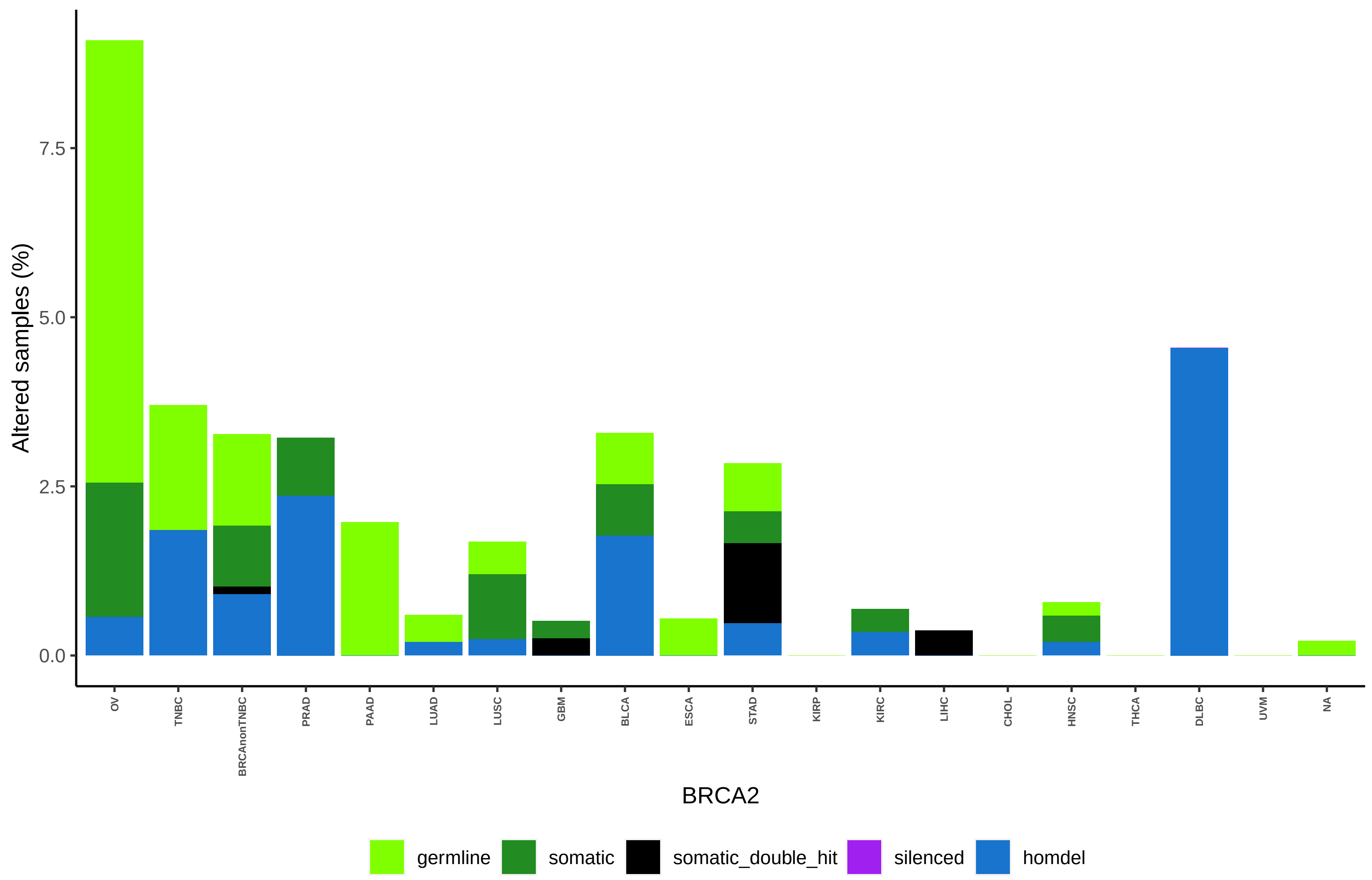
Altered samples (%)
0.0
2.5
5.0
7.5
OV
TNBC
BRCAnonTNBC
PRAD
PAAD
LUAD
LUSC
GBM
BLCA
ESCA
STAD
KIRP
KIRC
LIHC
CHOL
HNSC
THCA
DLBC
UVM
NA
BRCA2
germline
somatic
somatic_double_hit
silenced
homdel

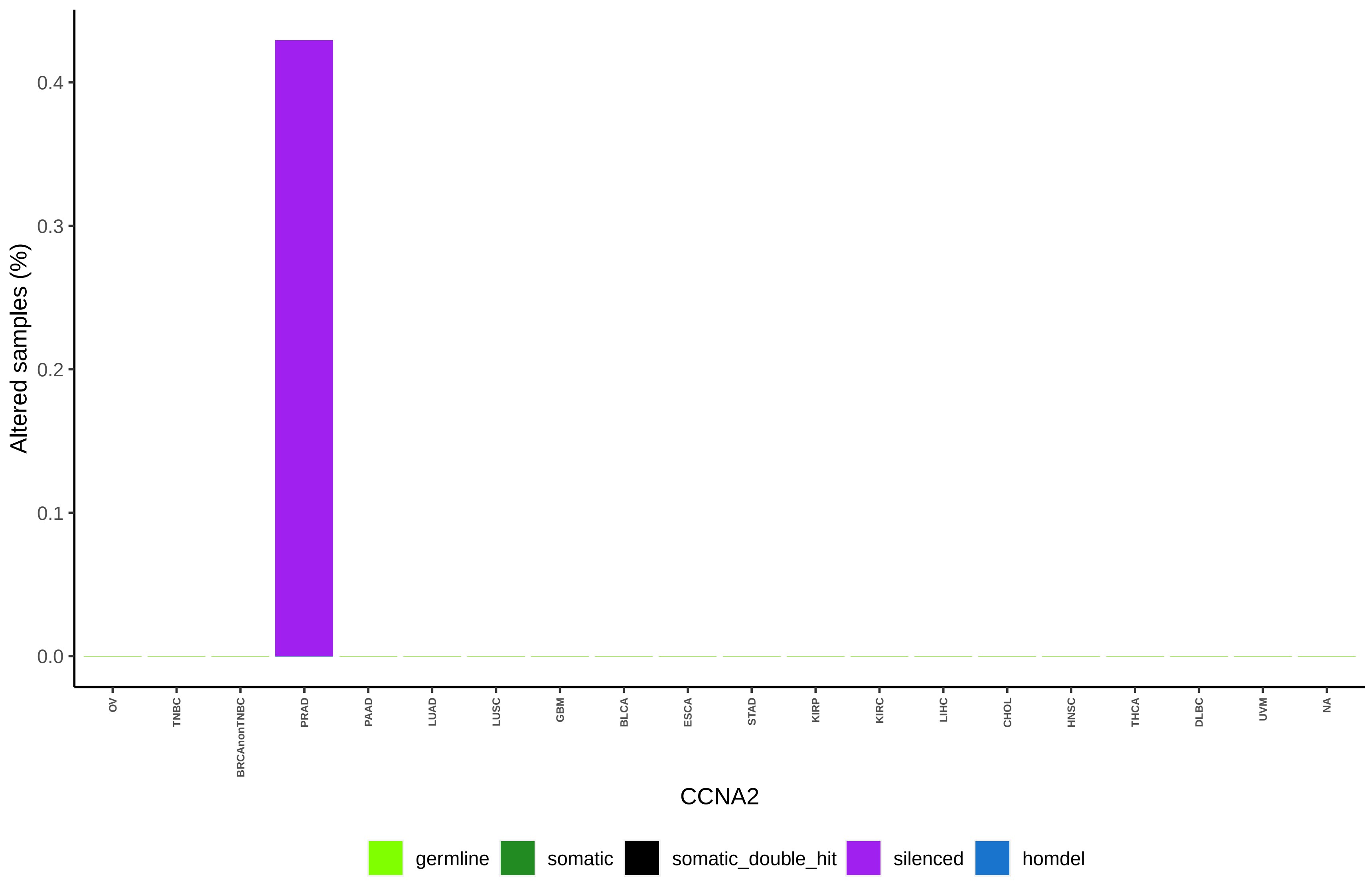
Altered samples (%)
0.0
0.1
0.2
0.3
0.4
OV
TNBC
BRCAnonTNBC
PRAD
PAAD
LUAD
LUSC
GBM
BLCA
ESCA
STAD
KIRP
KIRC
LIHC
CHOL
HNSC
THCA
DLBC
UVM
NA
CCNA2
germline
somatic
somatic_double_hit
silenced
homdel

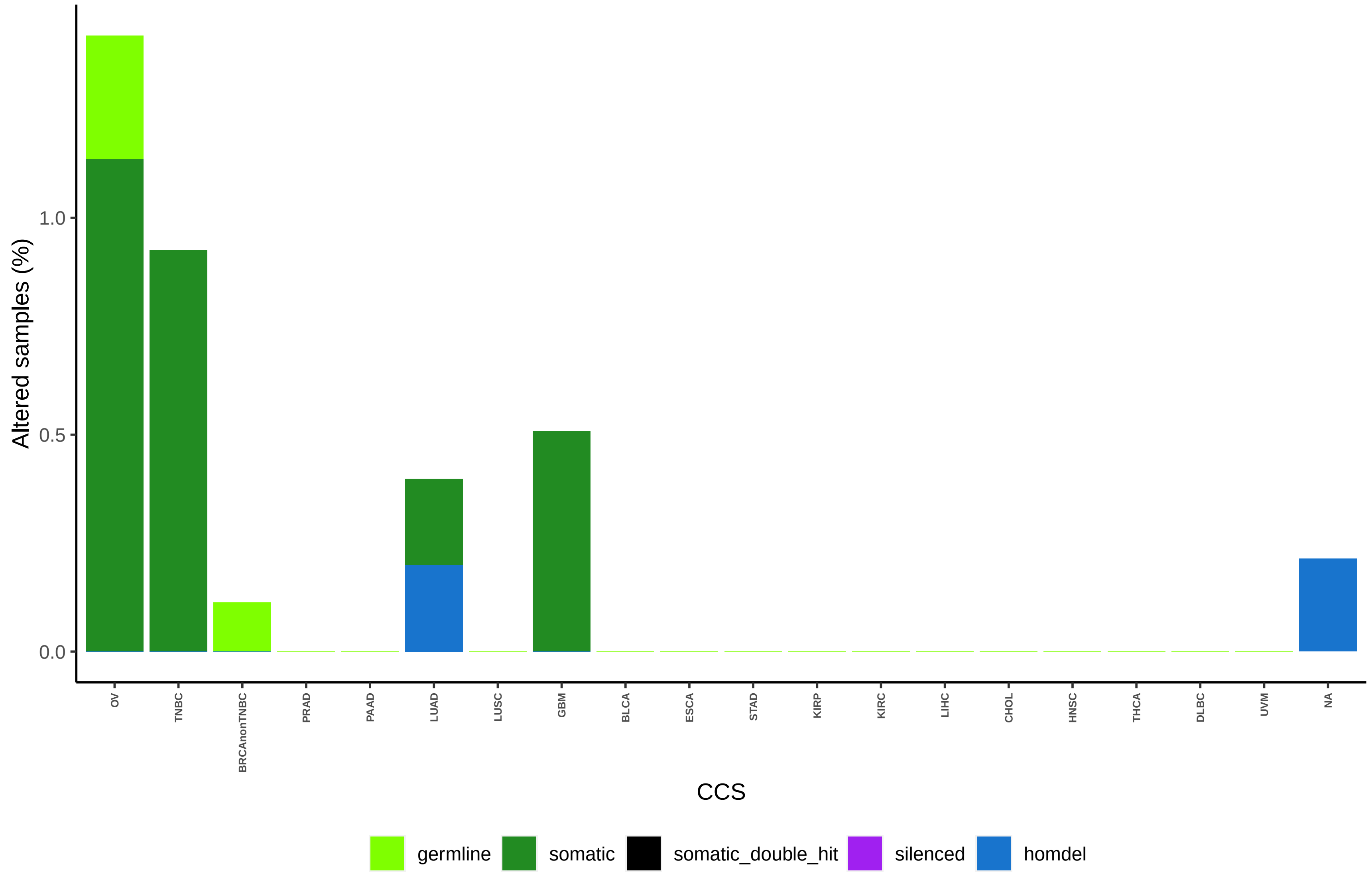

Altered samples (%)
0.0
0.5
1.0
OV
TNBC
BRCAnonTNBC
PRAD
PAAD
LUAD
LUSC
GBM
BLCA
ESCA
STAD
KIRP
KIRC
LIHC
CHOL
HNSC
THCA
DLBC
UVM
NA
CCS
germline
somatic
somatic_double_hit
silenced
homdel


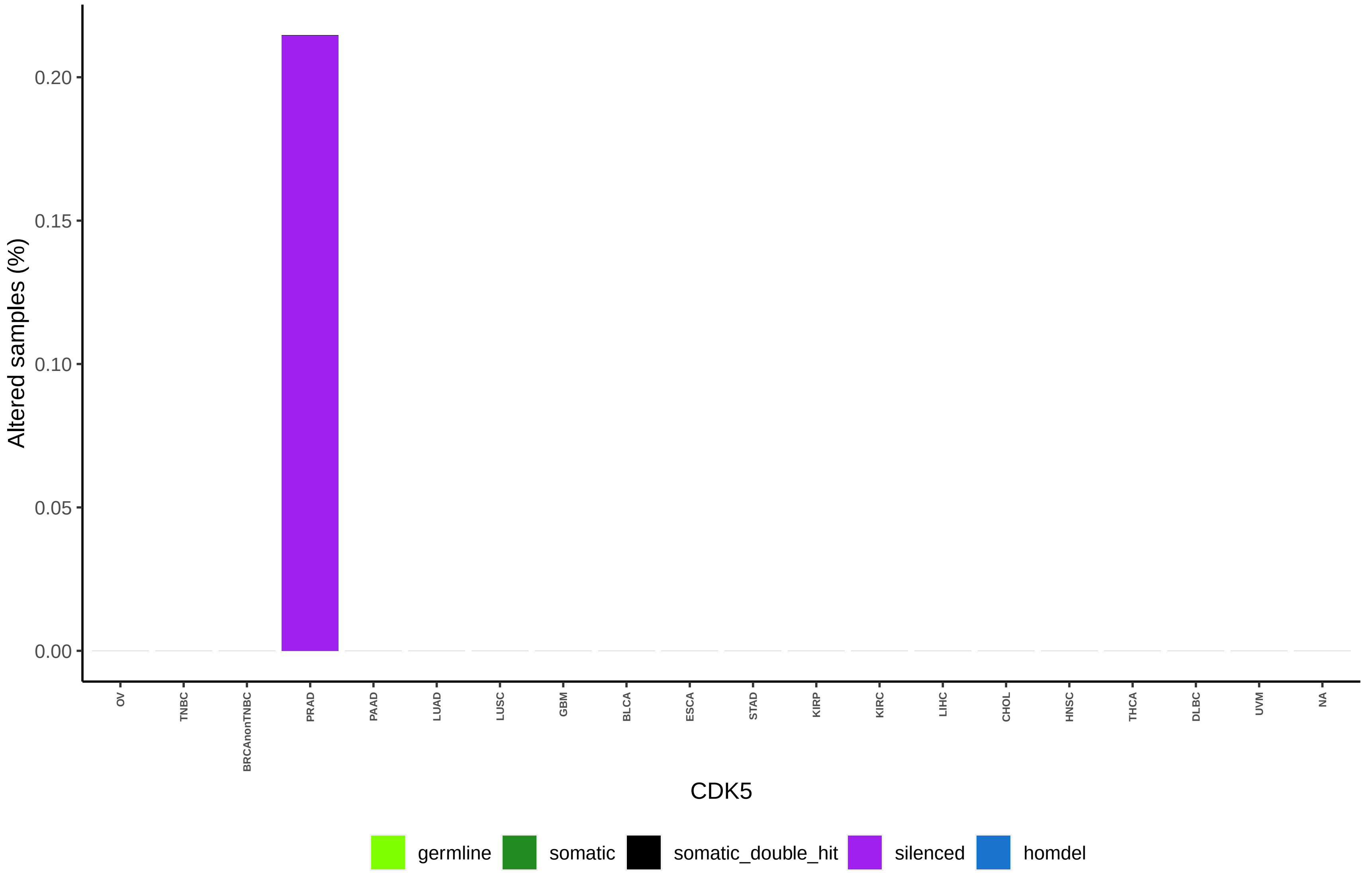

Altered samples (%)
0.00
0.05
0.10
0.15
0.20
OV
TNBC
BRCAnonTNBC
PRAD
PAAD
LUAD
LUSC
GBM
BLCA
ESCA
STAD
KIRP
KIRC
LIHC
CHOL
HNSC
THCA
DLBC
UVM
NA
CDK5
germline
somatic
somatic_double_hit
silenced
homdel


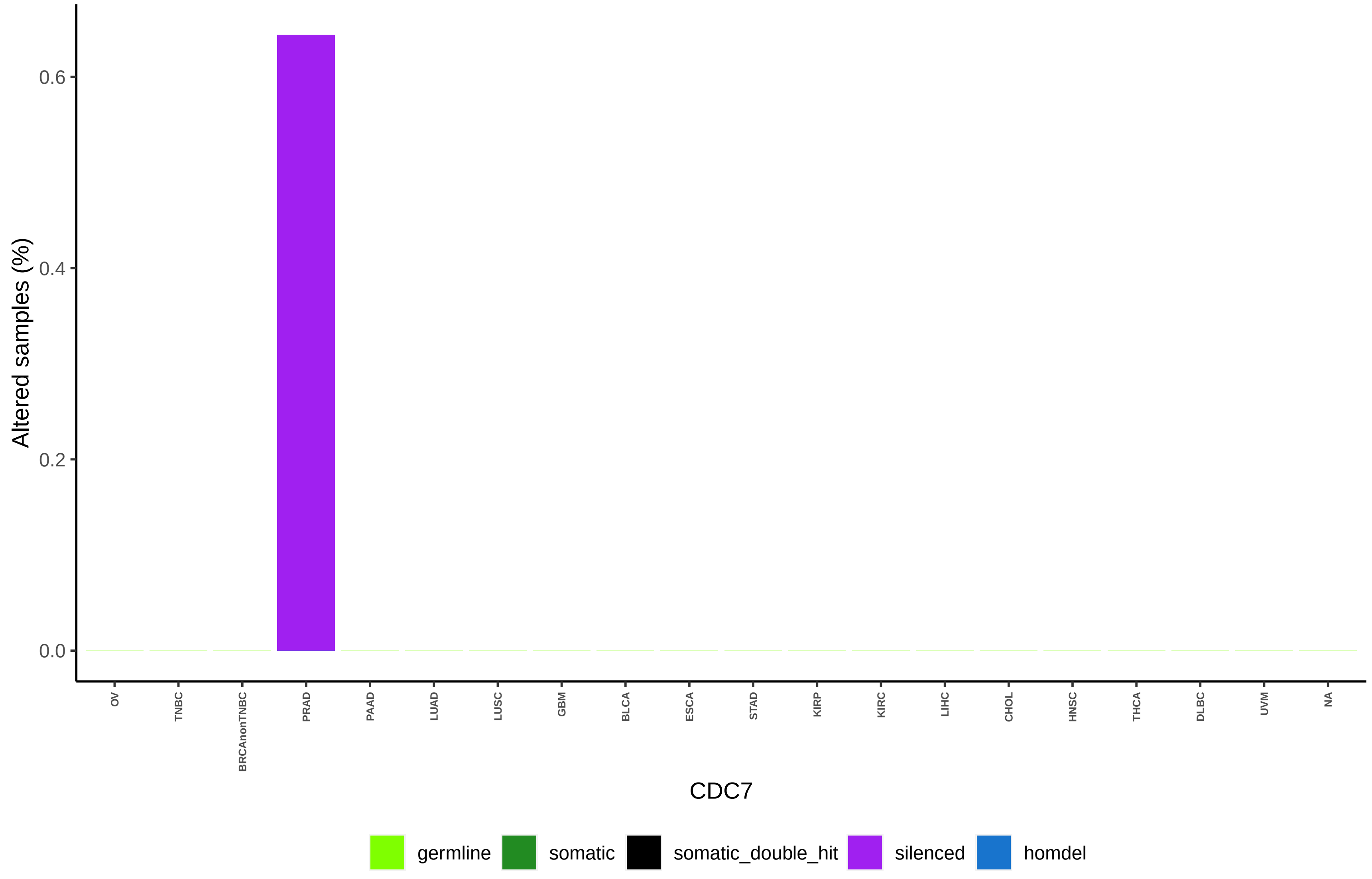

Altered samples (%)
0.0
0.2
0.4
0.6
OV
TNBC
BRCAnonTNBC
PRAD
PAAD
LUAD
LUSC
GBM
BLCA
ESCA
STAD
KIRP
KIRC
LIHC
CHOL
HNSC
THCA
DLBC
UVM
NA
CDC7
germline
somatic
somatic_double_hit
silenced
homdel


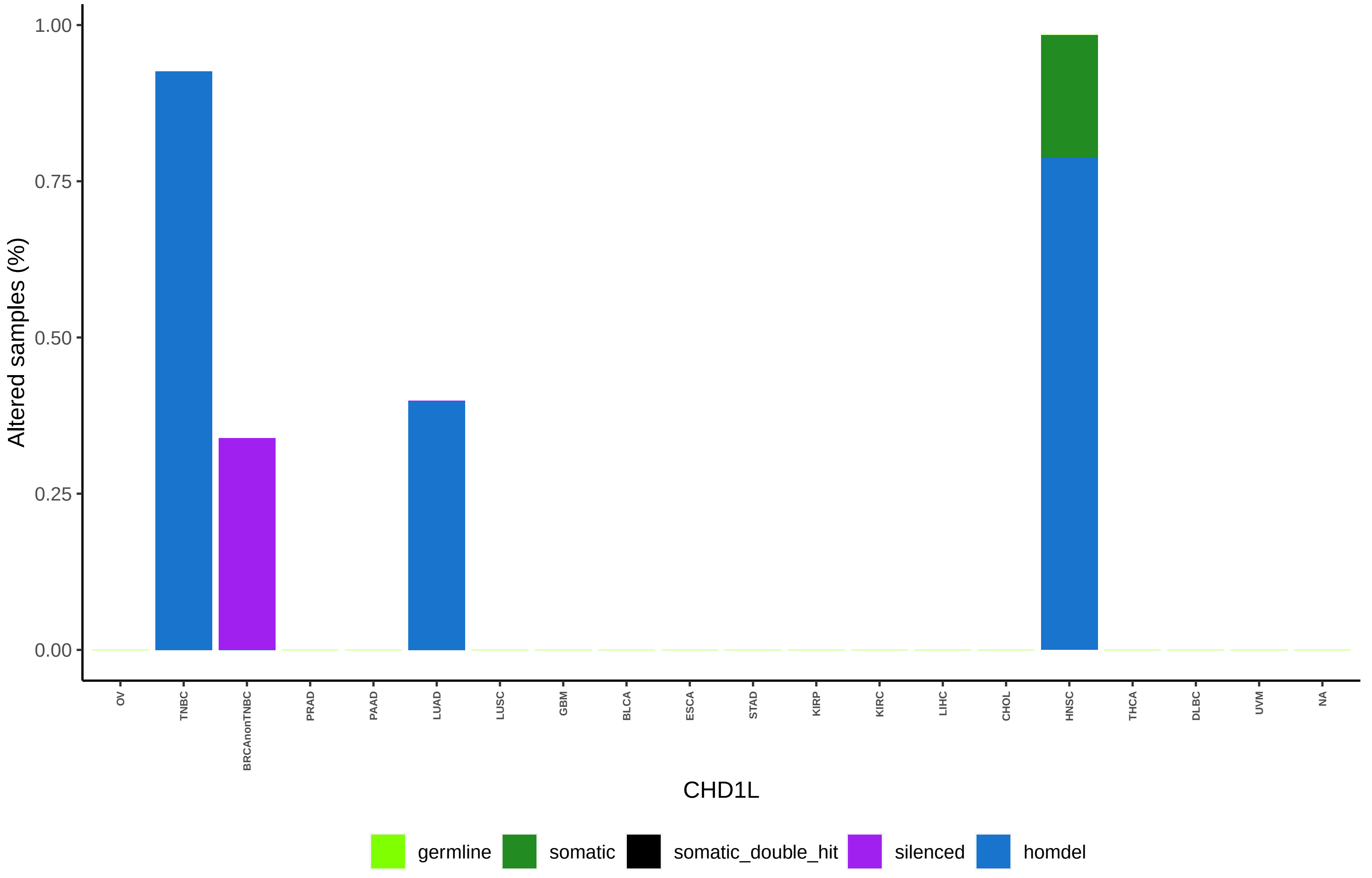

Altered samples (%)
0.00
0.25
0.50
0.75
1.00
OV
TNBC
BRCAnonTNBC
PRAD
PAAD
LUAD
LUSC
GBM
BLCA
ESCA
STAD
KIRP
KIRC
LIHC
CHOL
HNSC
THCA
DLBC
UVM
NA
CHD1L
germline
somatic
somatic_double_hit
silenced
homdel

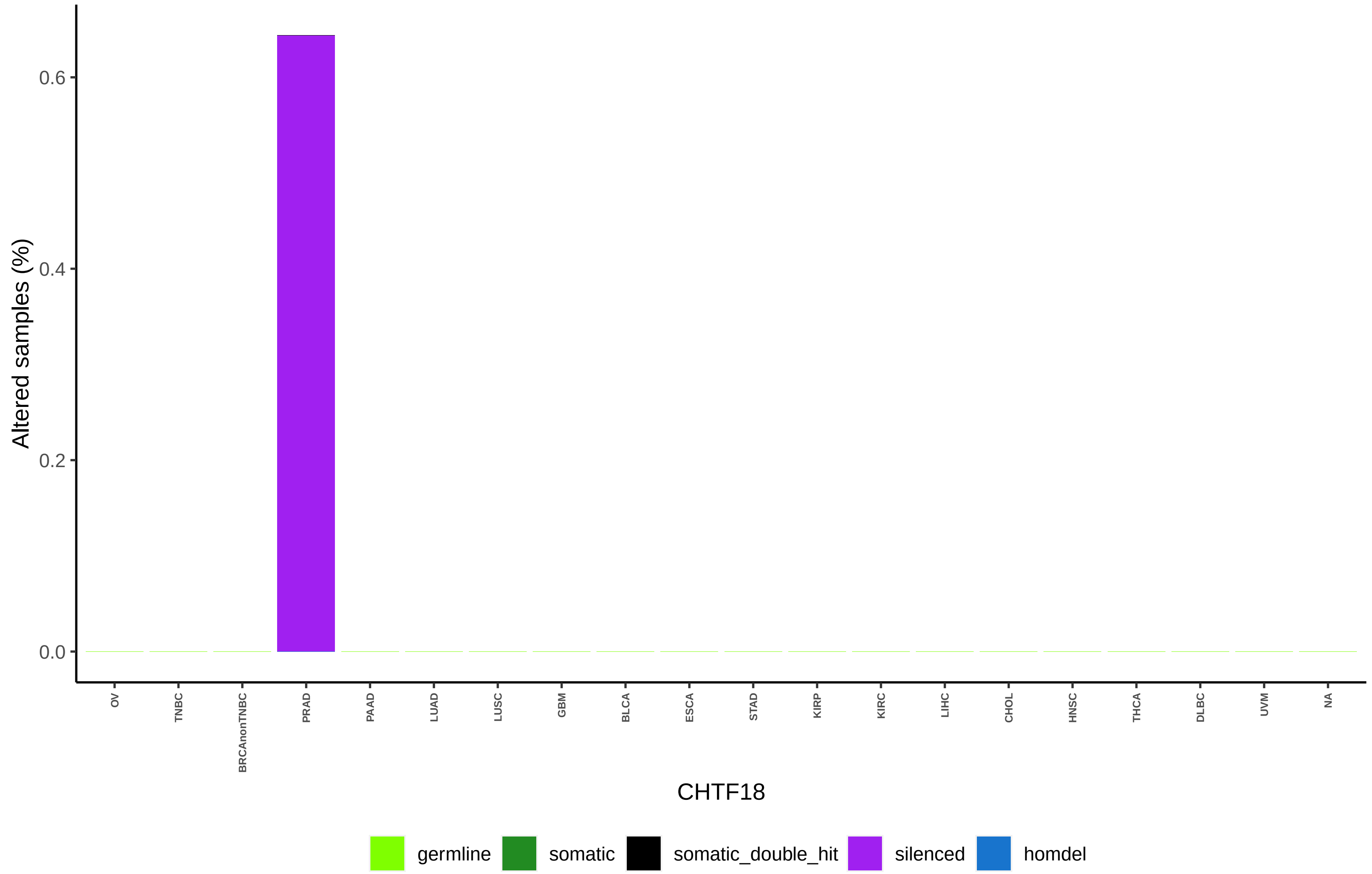
Altered samples (%)
0.0
0.2
0.4
0.6
OV
TNBC
BRCAnonTNBC
PRAD
PAAD
LUAD
LUSC
GBM
BLCA
ESCA
STAD
KIRP
KIRC
LIHC
CHOL
HNSC
THCA
DLBC
UVM
NA
CHTF18
germline
somatic
somatic_double_hit
silenced
homdel

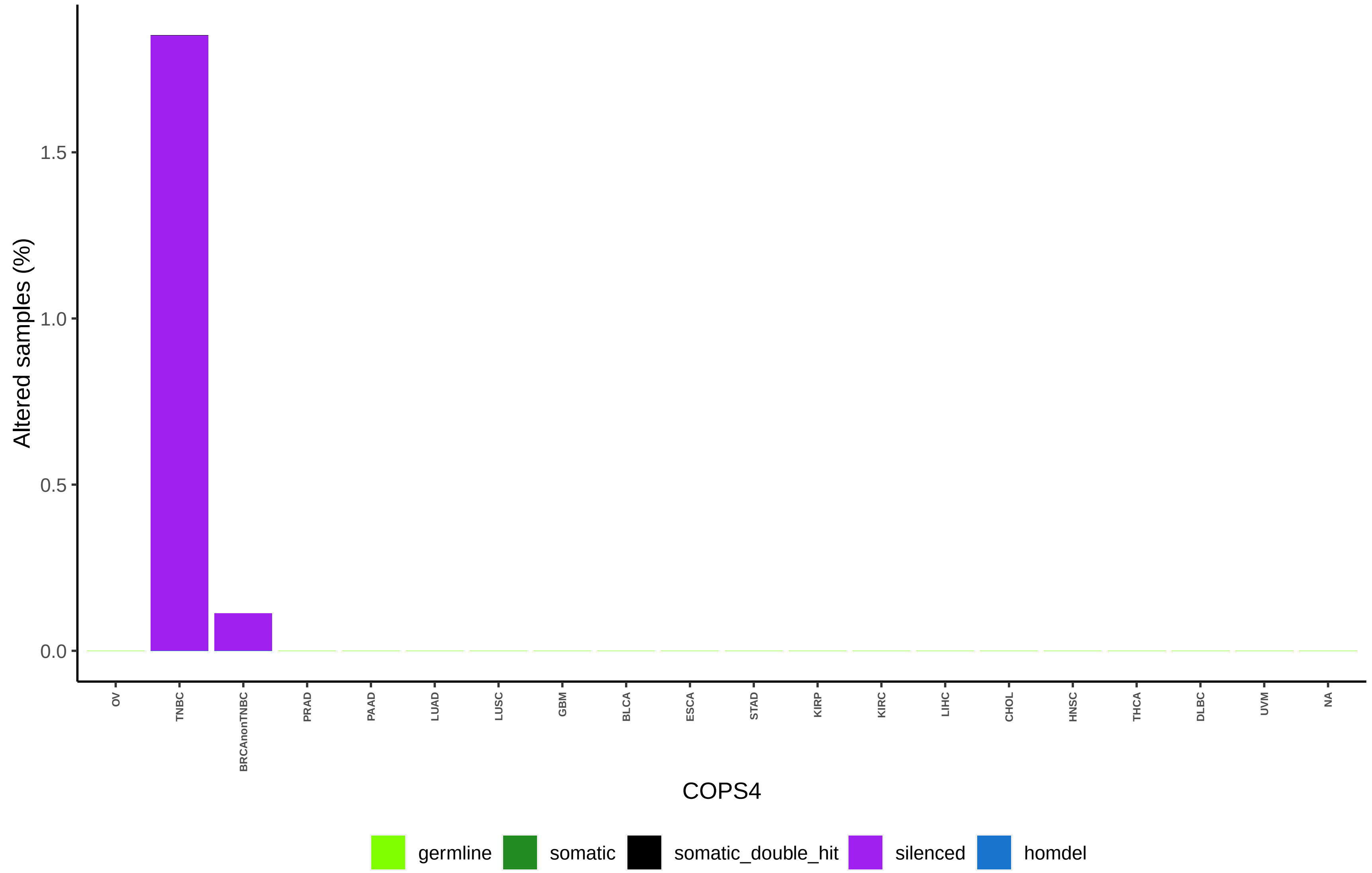
Altered samples (%)
0.0
0.5
1.0
1.5
OV
TNBC
BRCAnonTNBC
PRAD
PAAD
LUAD
LUSC
GBM
BLCA
ESCA
STAD
KIRP
KIRC
LIHC
CHOL
HNSC
THCA
DLBC
UVM
NA
COPS4
germline
somatic
somatic_double_hit
silenced
homdel

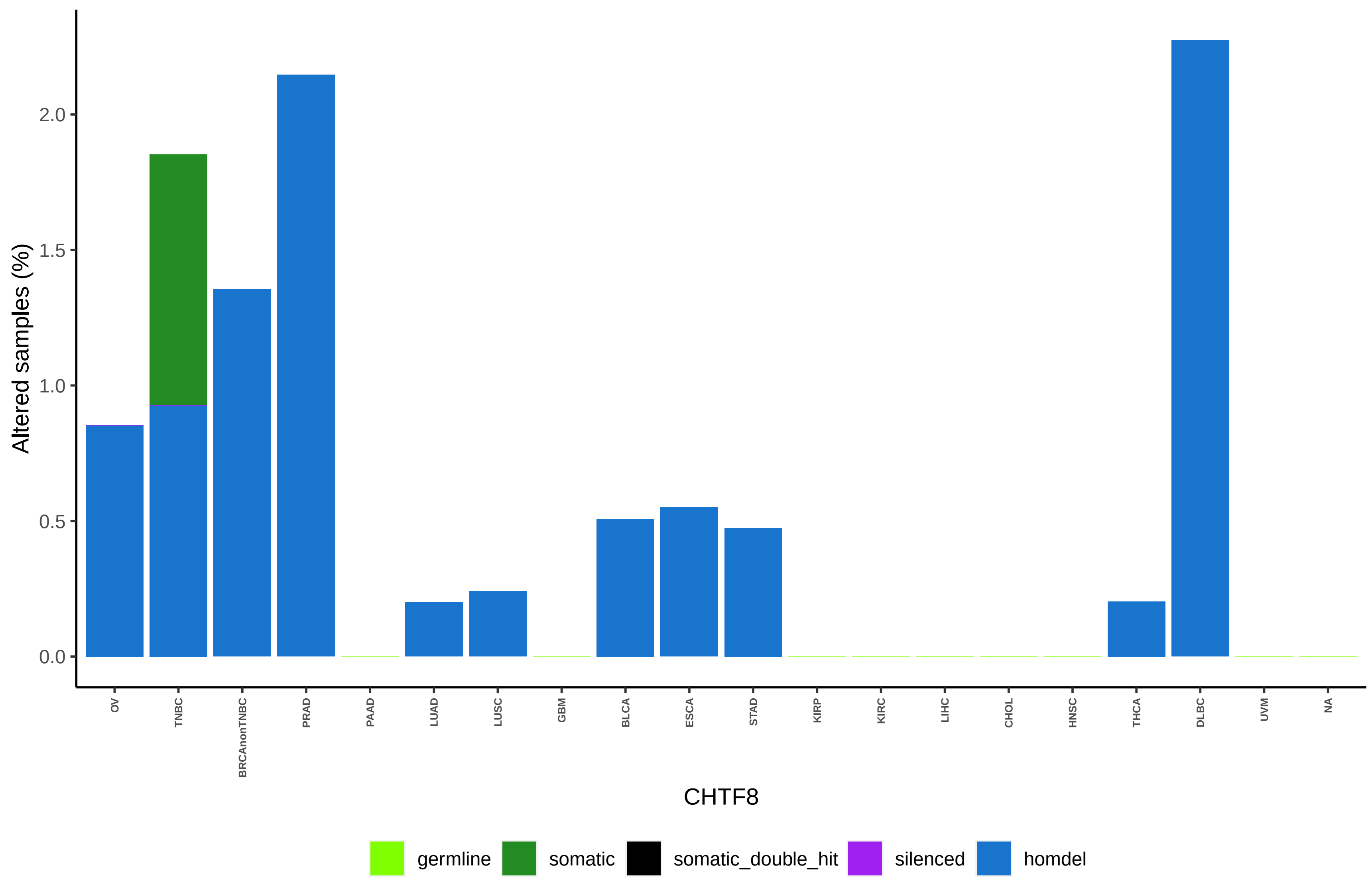
Altered samples (%)
0.0
0.5
1.0
1.5
2.0
OV
TNBC
BRCAnonTNBC
PRAD
PAAD
LUAD
LUSC
GBM
BLCA
ESCA
STAD
KIRP
KIRC
LIHC
CHOL
HNSC
THCA
DLBC
UVM
NA
CHTF8
germline
somatic
somatic_double_hit
silenced
homdel

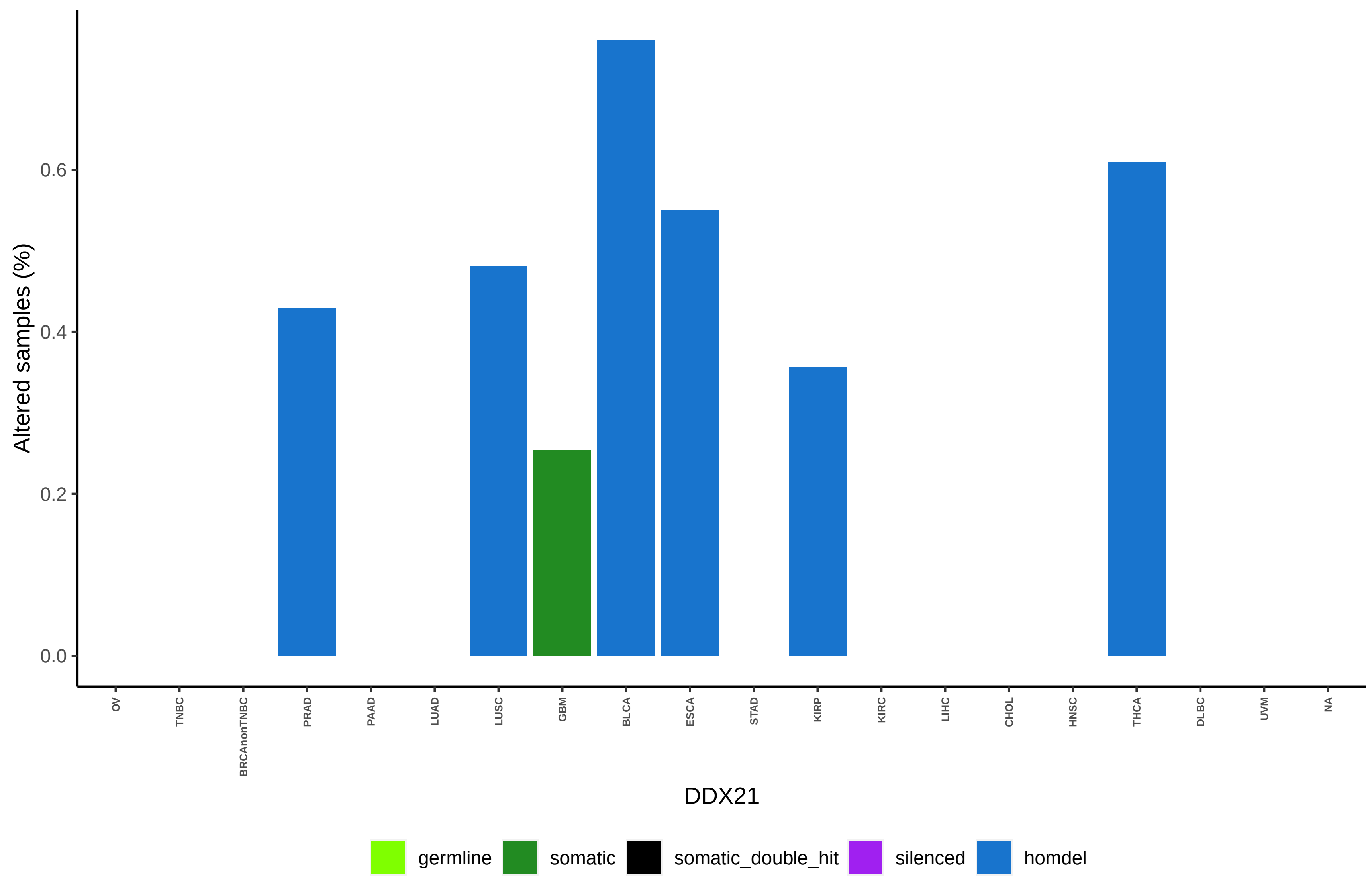
Altered samples (%)
0.0
0.2
0.4
0.6
OV
TNBC
BRCAnonTNBC
PRAD
PAAD
LUAD
LUSC
GBM
BLCA
ESCA
STAD
KIRP
KIRC
LIHC
CHOL
HNSC
THCA
DLBC
UVM
NA
DDX21
germline
somatic
somatic_double_hit
silenced
homdel

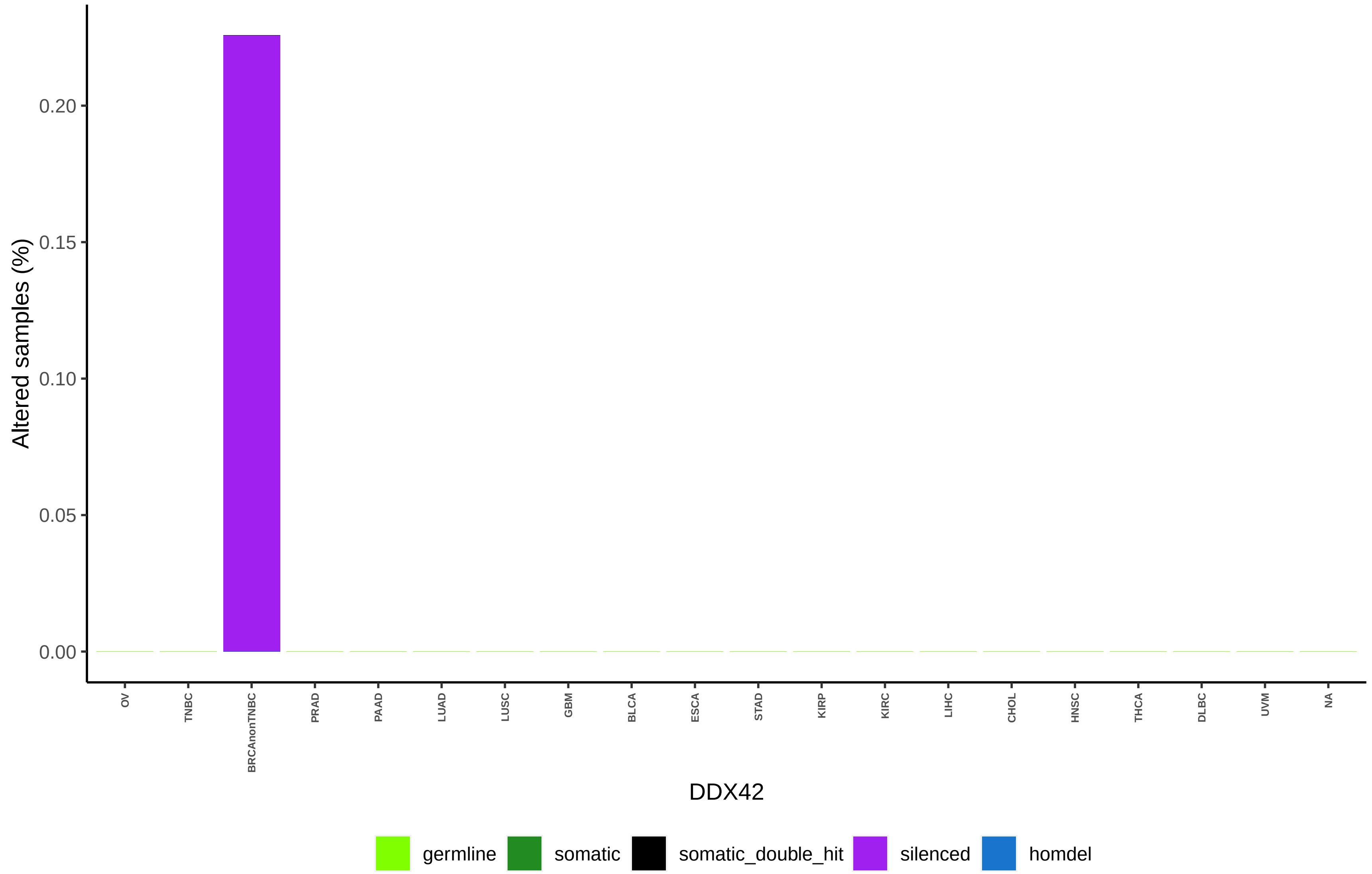

Altered samples (%)
0.20
0.15
0.10
0.05
0.00
OV
TNBC
BRCAnonTNBC
PRAD
PAAD
LUAD
LUSC
GBM
BLCA
ESCA
STAD
KIRP
KIRC
LIHC
CHOL
HNSC
THCA
DLBC
UVM
NA
DDX42
germline
somatic
somatic_double_hit
silenced
homdel


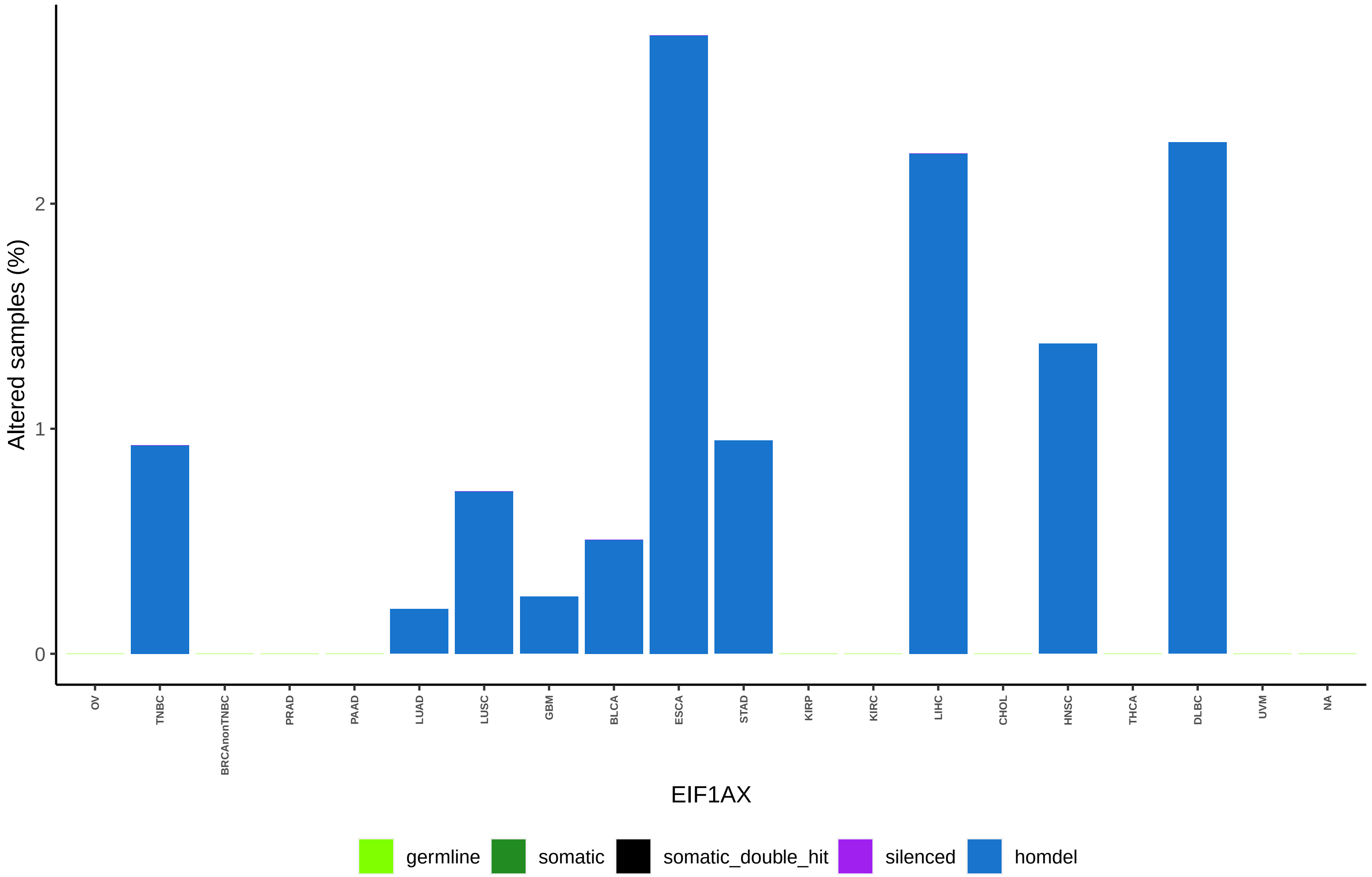

Altered samples (%)
2
1
0
OV
TNBC
BRCAnonTNBC
PRAD
PAAD
LUAD
LUSC
GBM
BLCA
ESCA
STAD
KIRP
KIRC
LIHC
CHOL
HNSC
THCA
DLBC
UVM
NA
EIF1AX
germline
somatic
somatic_double_hit
silenced
homdel


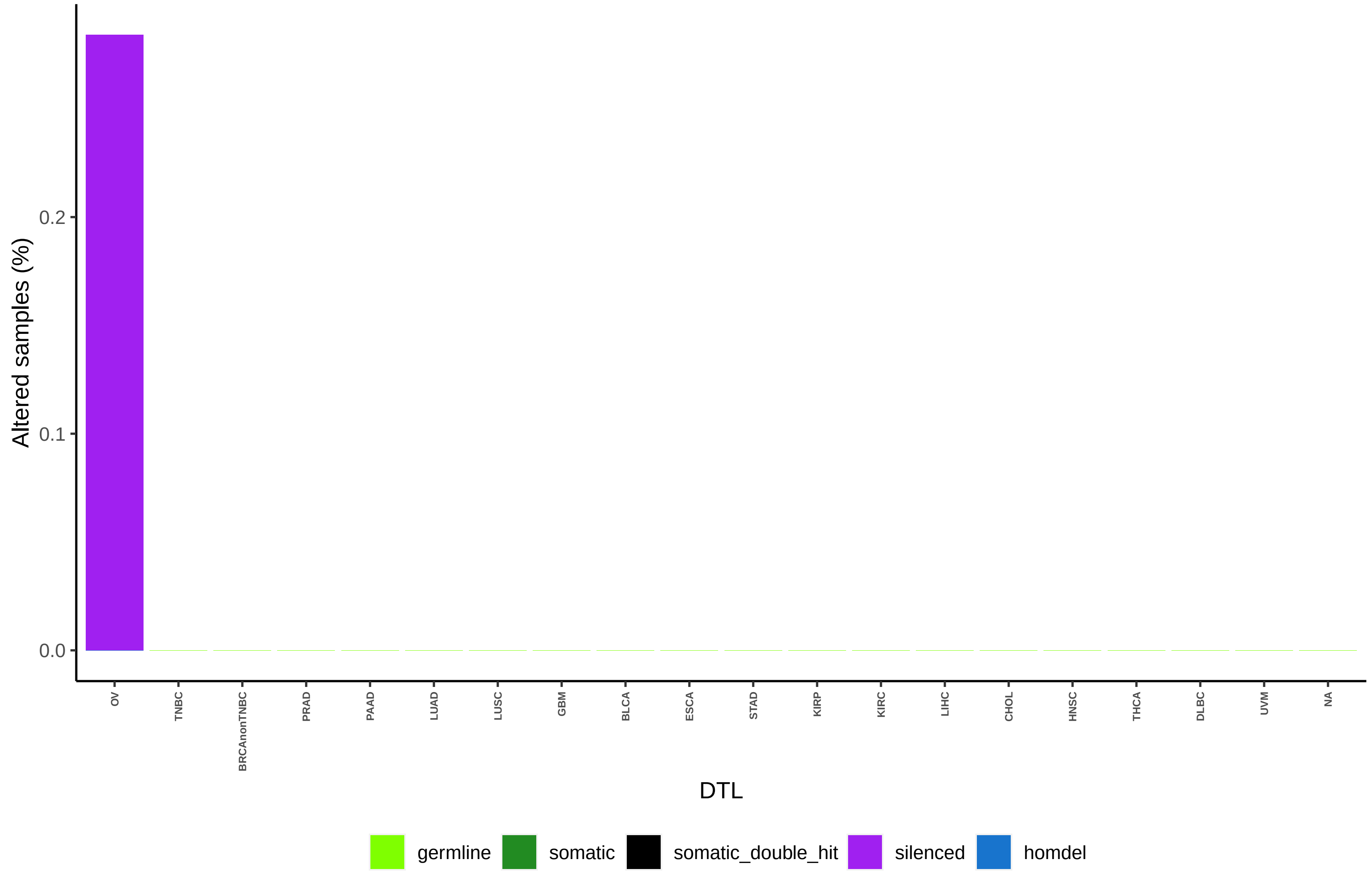

Altered samples (%)
0.2
0.1
0.0
OV
TNBC
BRCAnonTNBC
PRAD
PAAD
LUAD
LUSC
GBM
BLCA
ESCA
STAD
KIRP
KIRC
LIHC
CHOL
HNSC
THCA
DLBC
UVM
NA
DTL
germline
somatic
somatic_double_hit
silenced
homdel


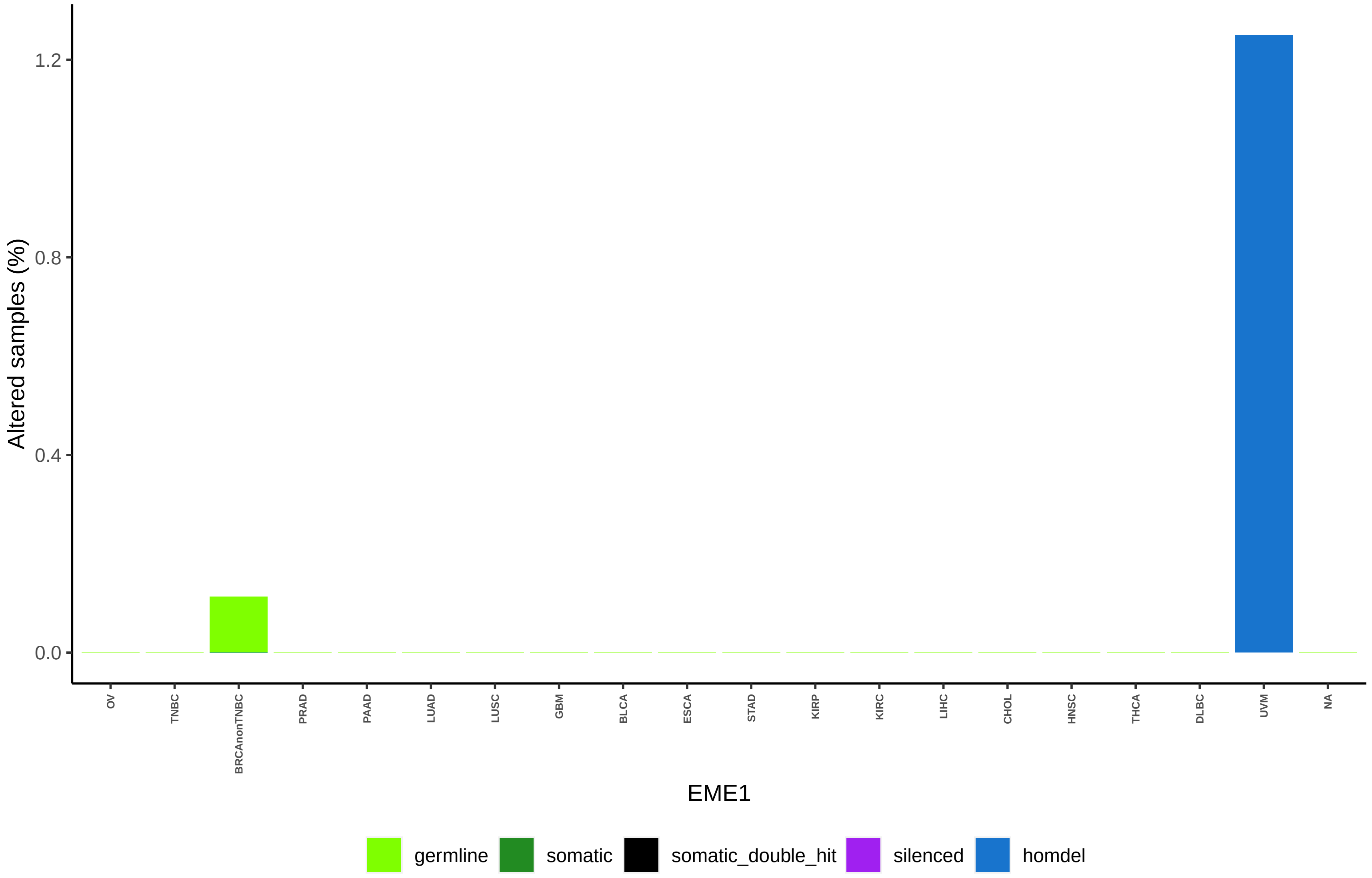

Altered samples (%)
1.2
0.8
0.4
0.0
OV
TNBC
BRCAnonTNBC
PRAD
PAAD
LUAD
LUSC
GBM
BLCA
ESCA
STAD
KIRP
KIRC
LIHC
CHOL
HNSC
THCA
DLBC
UVM
NA
EME1
germline
somatic
somatic_double_hit
silenced
homdel

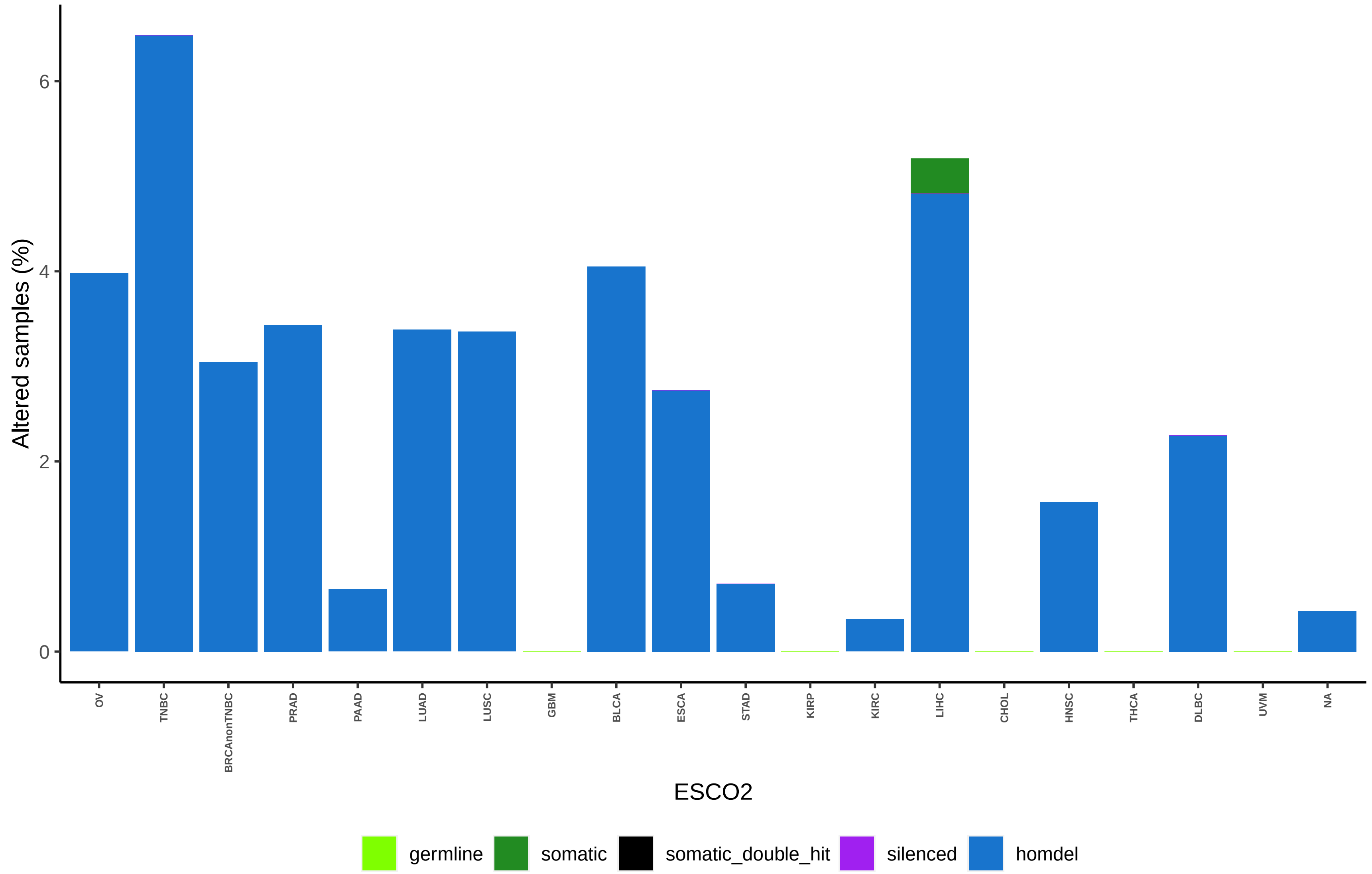
Altered samples (%)
0
2
4
6
OV
TNBC
BRCAnonTNBC
PRAD
PAAD
LUAD
LUSC
GBM
BLCA
ESCA
STAD
KIRP
KIRC
LIHC
CHOL
HNSC
THCA
DLBC
UVM
NA
ESCO2
germline
somatic
somatic_double_hit
silenced
homdel

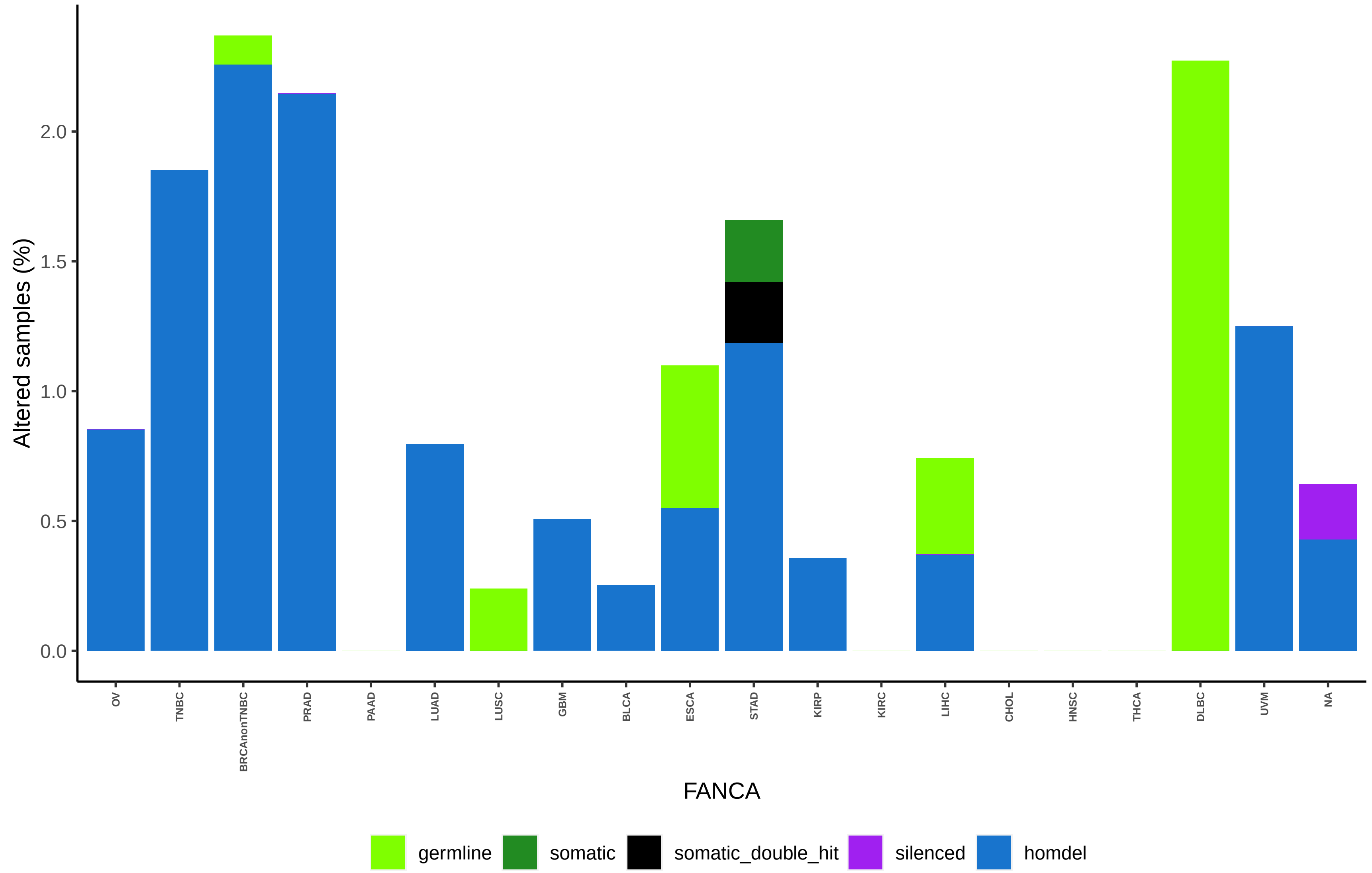
Altered samples (%)
0.0
0.5
1.0
1.5
2.0
OV
TNBC
BRCAnonTNBC
PRAD
PAAD
LUAD
LUSC
GBM
BLCA
ESCA
STAD
KIRP
KIRC
LIHC
CHOL
HNSC
THCA
DLBC
UVM
NA
FANCA
germline
somatic
somatic_double_hit
silenced
homdel

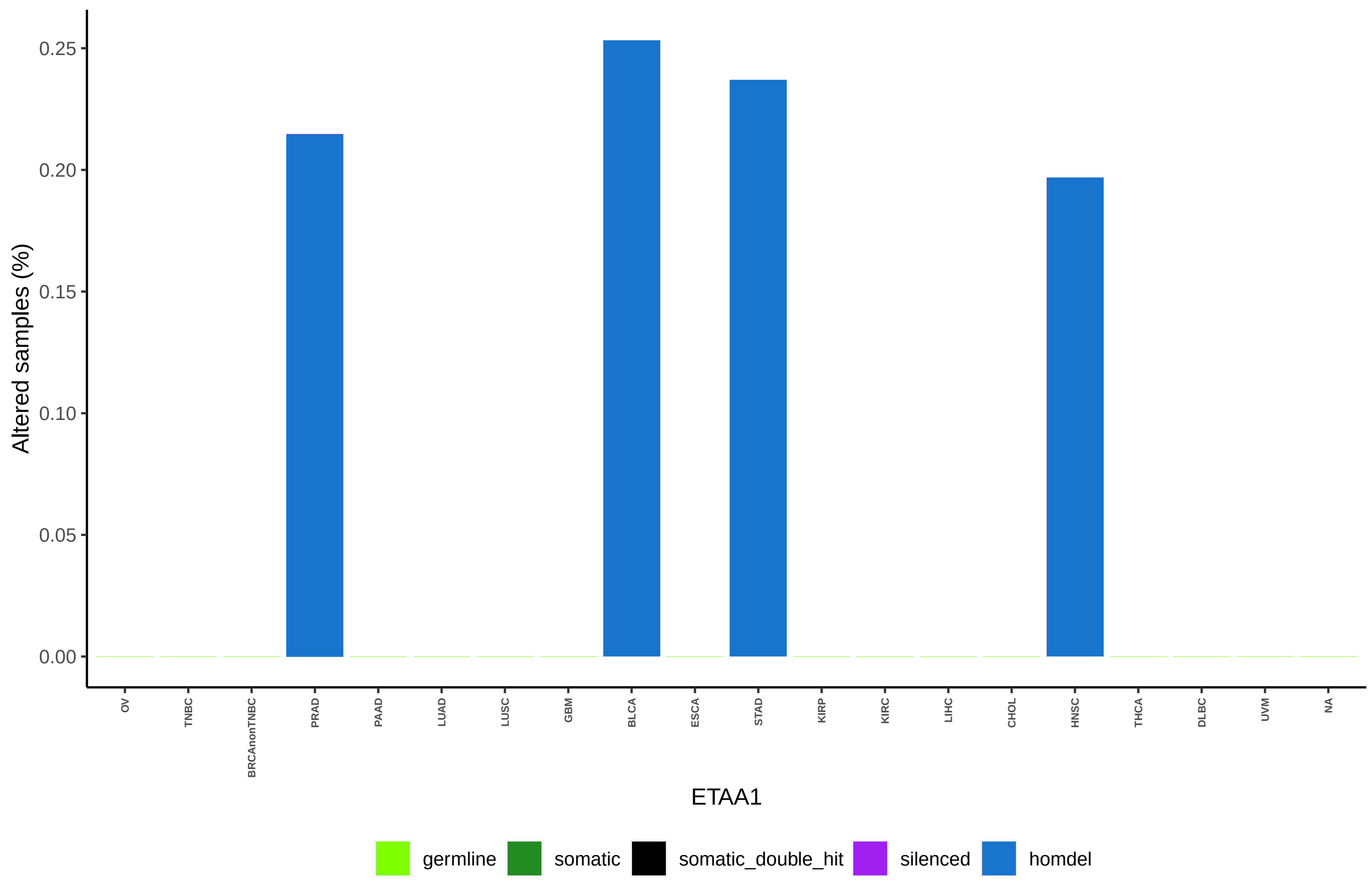
Altered samples (%)
0.00
0.05
0.10
0.15
0.20
0.25
OV
TNBC
BRCAnonTNBC
PRAD
PAAD
LUAD
LUSC
GBM
BLCA
ESCA
STAD
KIRP
KIRC
LIHC
CHOL
HNSC
THCA
DLBC
UVM
NA
ETAA1
germline
somatic
somatic_double_hit
silenced
homdel

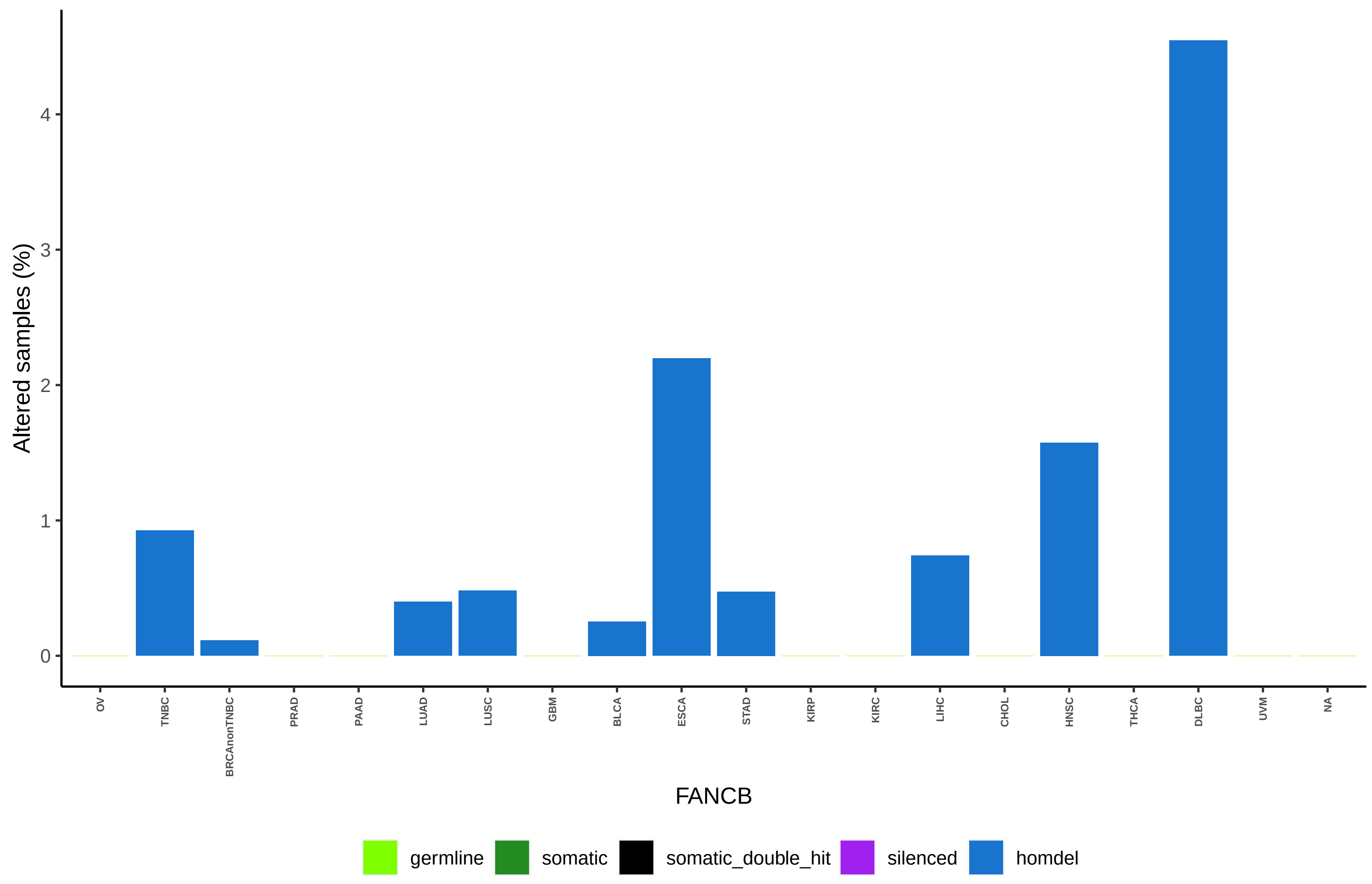
Altered samples (%)
0
1
2
3
4
OV
TNBC
BRCAnonTNBC
PRAD
PAAD
LUAD
LUSC
GBM
BLCA
ESCA
STAD
KIRP
KIRC
LIHC
CHOL
HNSC
THCA
DLBC
UVM
NA
FANCB
germline
somatic
somatic_double_hit
silenced
homdel

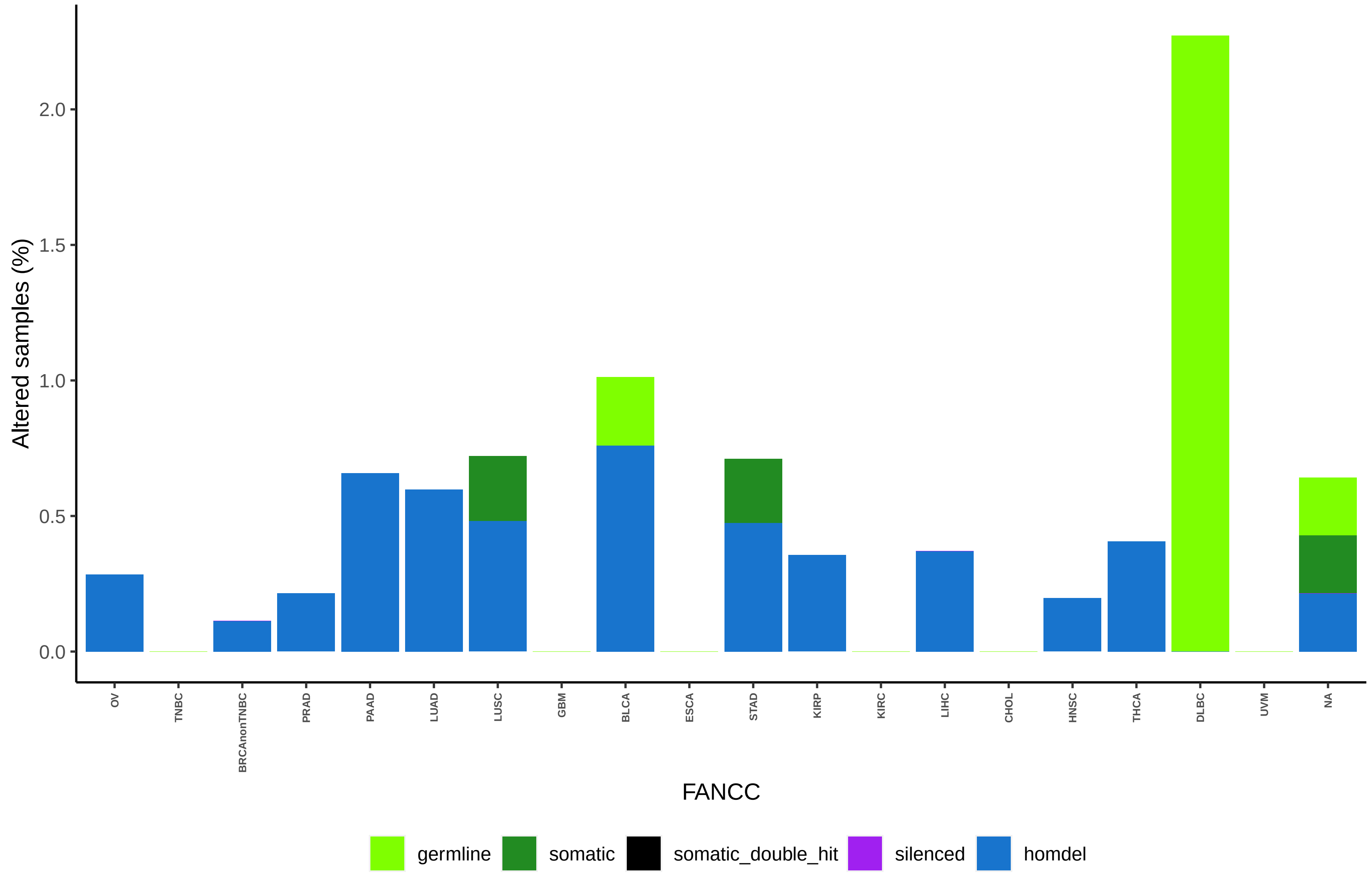
Altered samples (%)
0.0
0.5
1.0
1.5
2.0
OV TNBC BRCAnonTNBC PRAD PAAD LUAD LUSC GBM BLCA ESCA STAD KIRP KIRC LIHC CHOL HNSC THCA DLBC UVM NA
FANCC
germline
somatic
somatic_double_hit
silenced
homdel

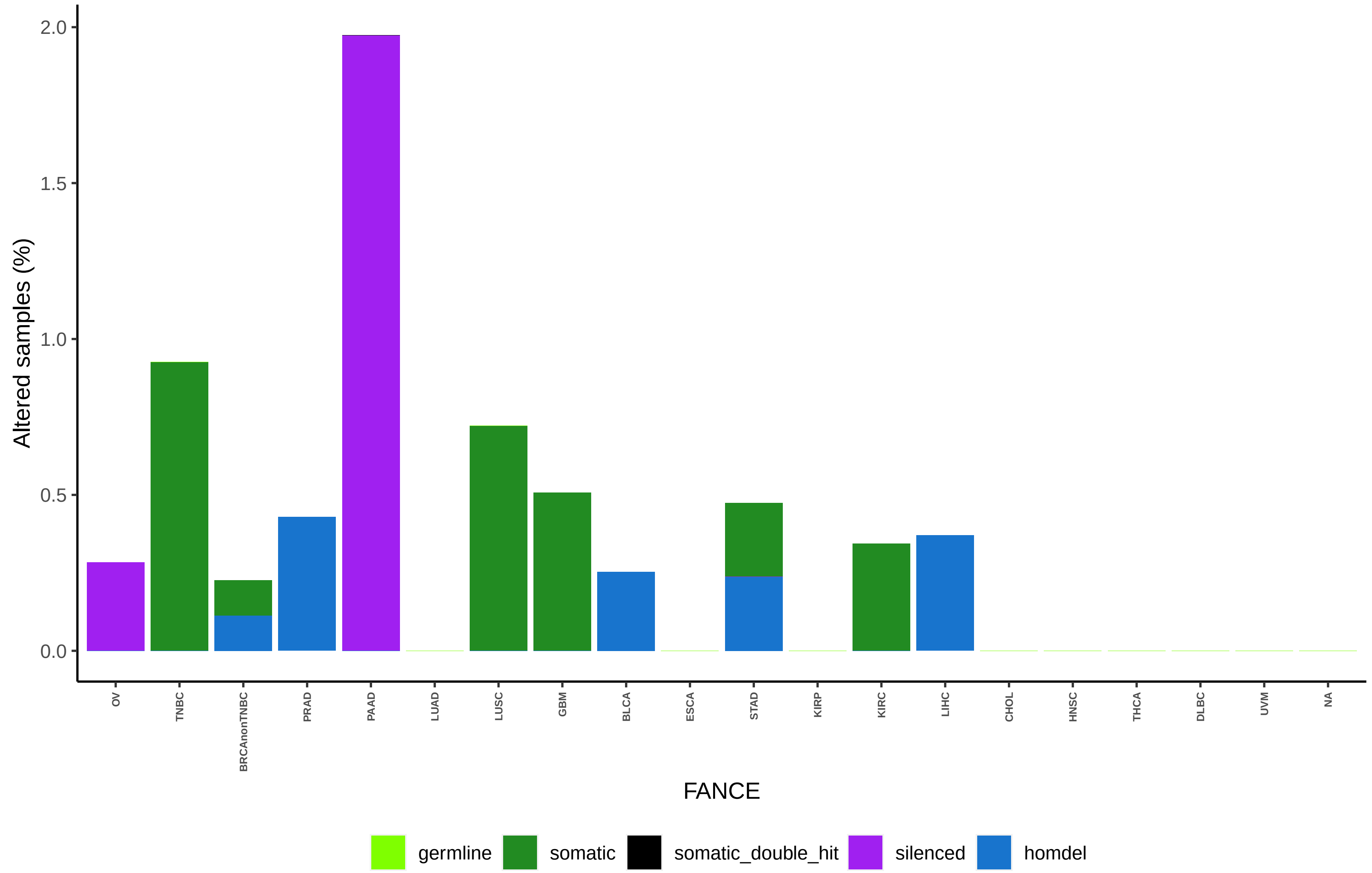
Altered samples (%)
0.0
0.5
1.0
1.5
2.0
OV TNBC BRCAnonTNBC PRAD PAAD LUAD LUSC GBM BLCA ESCA STAD KIRP KIRC LIHC CHOL HNSC THCA DLBC UVM NA
FANCE
germline
somatic
somatic_double_hit
silenced
homdel

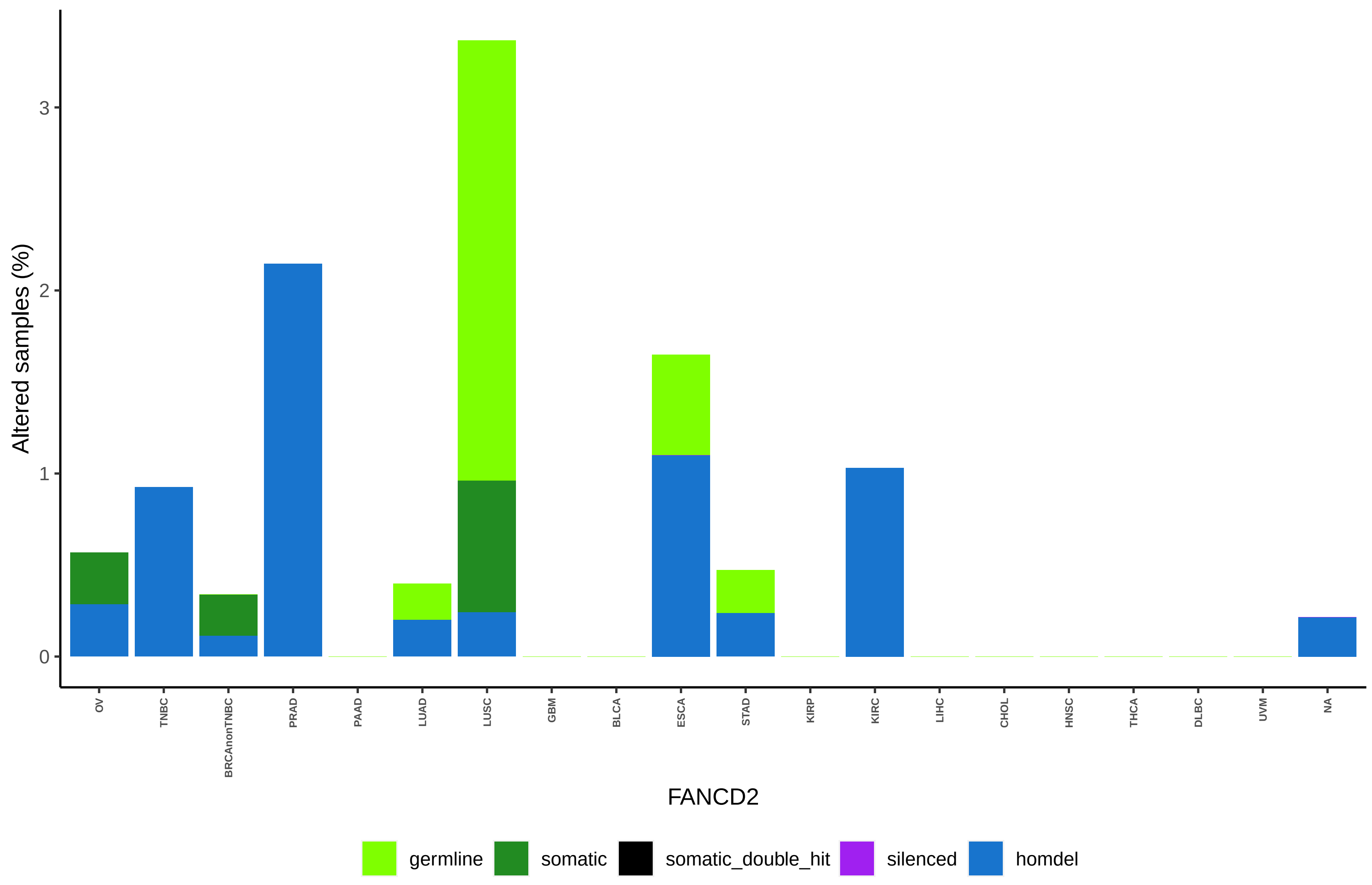
Altered samples (%)
0
1
2
3
OV TNBC BRCAnonTNBC PRAD PAAD LUAD LUSC GBM BLCA ESCA STAD KIRP KIRC LIHC CHOL HNSC THCA DLBC UVM NA
FANCD2
germline
somatic
somatic_double_hit
silenced
homdel

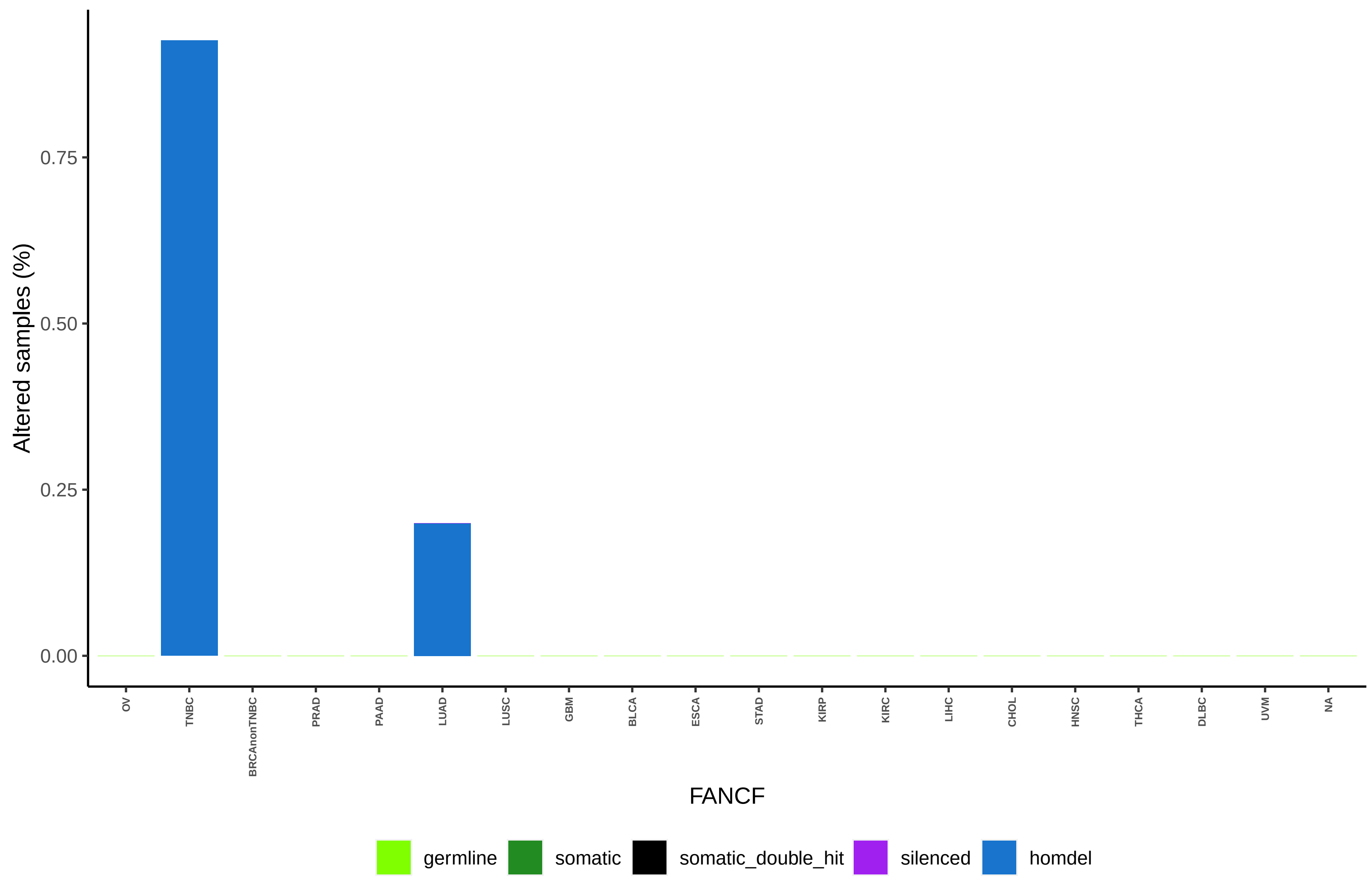
Altered samples (%)
0.00
0.25
0.50
0.75
OV TNBC BRCAnonTNBC PRAD PAAD LUAD LUSC GBM BLCA ESCA STAD KIRP KIRC LIHC CHOL HNSC THCA DLBC UVM NA
FANCF
germline
somatic
somatic_double_hit
silenced
homdel

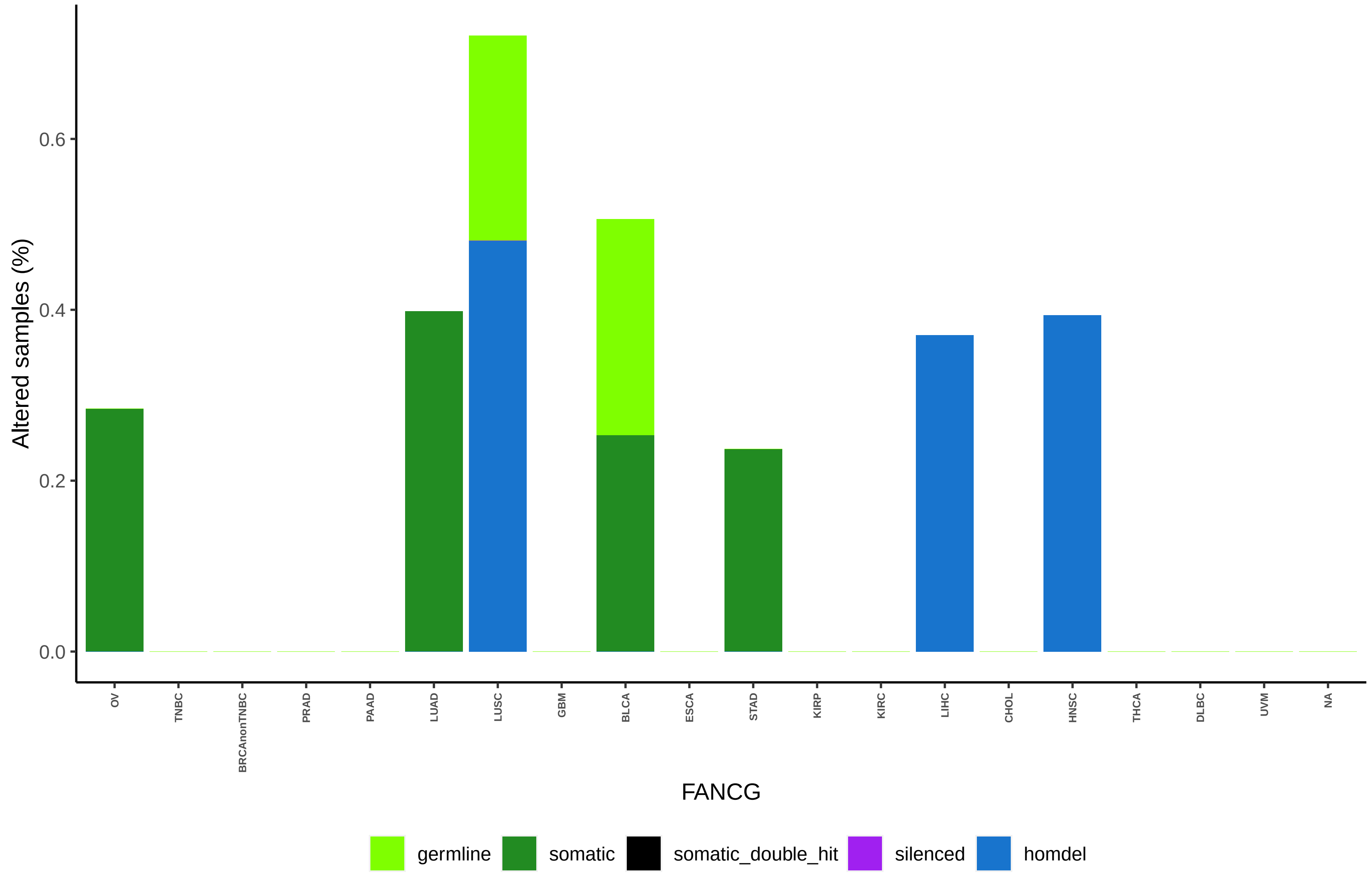
Altered samples (%)
0.0
0.2
0.4
0.6
OV
TNBC
BRCAnonTNBC
PRAD
PAAD
LUAD
LUSC
GBM
BLCA
ESCA
STAD
KIRP
KIRC
LIHC
CHOL
HNSC
THCA
DLBC
UVM
NA
FANCG
germline
somatic
somatic_double_hit
silenced
homdel

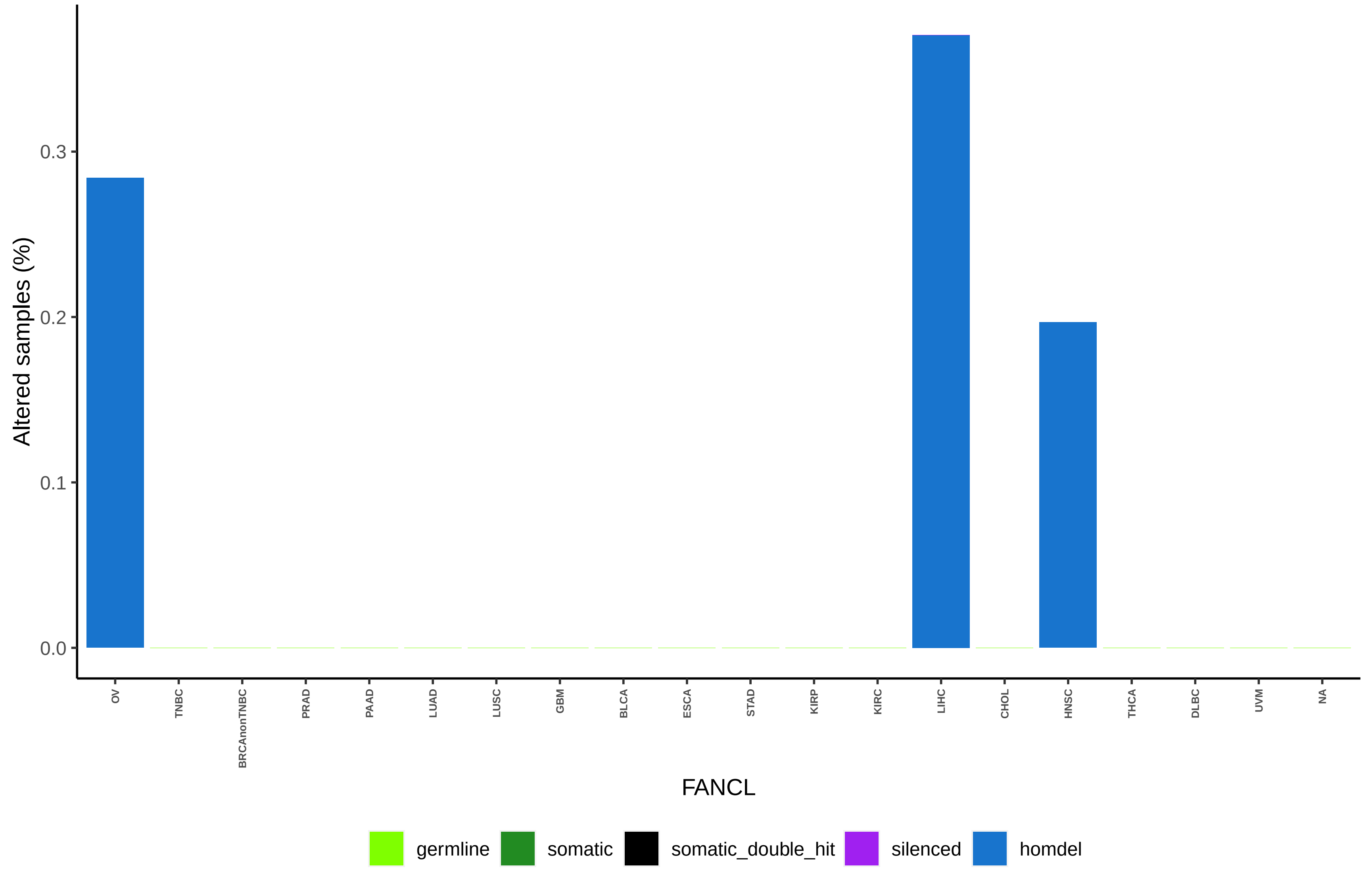
Altered samples (%)
0.0
0.1
0.2
0.3
OV
TNBC
BRCAnonTNBC
PRAD
PAAD
LUAD
LUSC
GBM
BLCA
ESCA
STAD
KIRP
KIRC
LIHC
CHOL
HNSC
THCA
DLBC
UVM
NA
FANCL
germline
somatic
somatic_double_hit
silenced
homdel

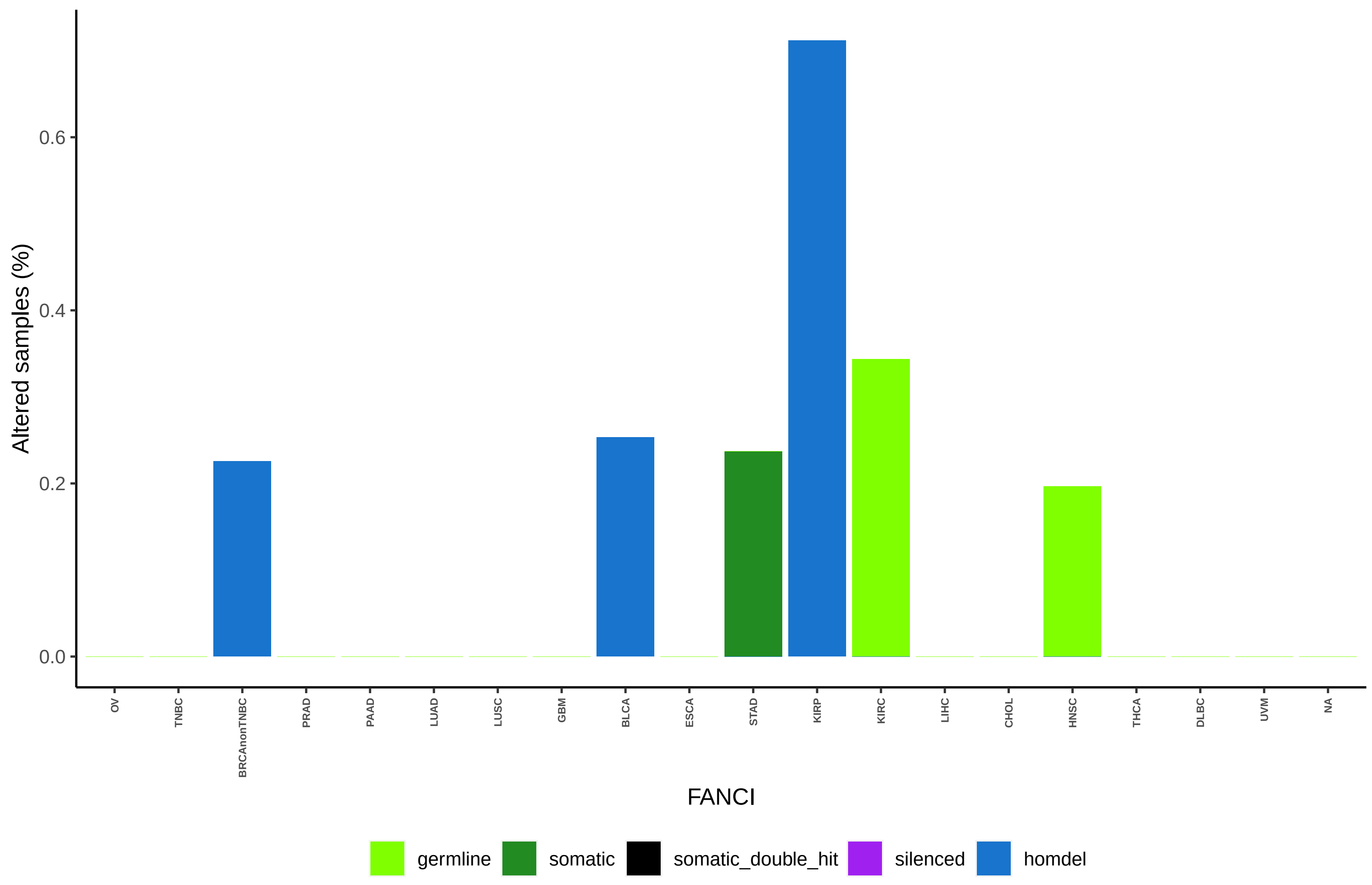
Altered samples (%)
0.0
0.2
0.4
0.6
OV
TNBC
BRCAnonTNBC
PRAD
PAAD
LUAD
LUSC
GBM
BLCA
ESCA
STAD
KIRP
KIRC
LIHC
CHOL
HNSC
THCA
DLBC
UVM
NA
FANCI
germline
somatic
somatic_double_hit
silenced
homdel

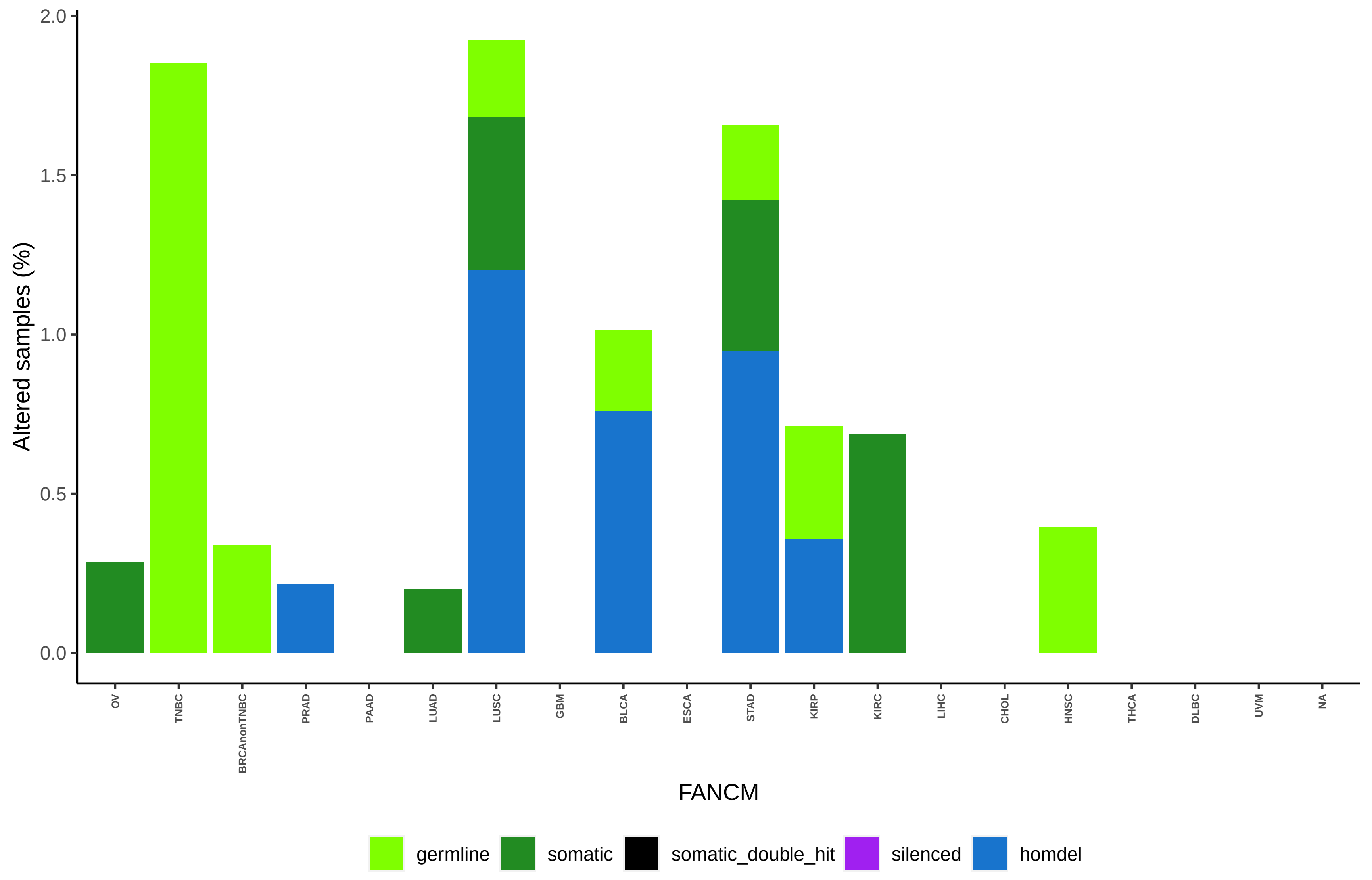
Altered samples (%)
0.0
0.5
1.0
1.5
2.0
OV
TNBC
BRCAnonTNBC
PRAD
PAAD
LUAD
LUSC
GBM
BLCA
ESCA
STAD
KIRP
KIRC
LIHC
CHOL
HNSC
THCA
DLBC
UVM
NA
FANCM
germline
somatic
somatic_double_hit
silenced
homdel

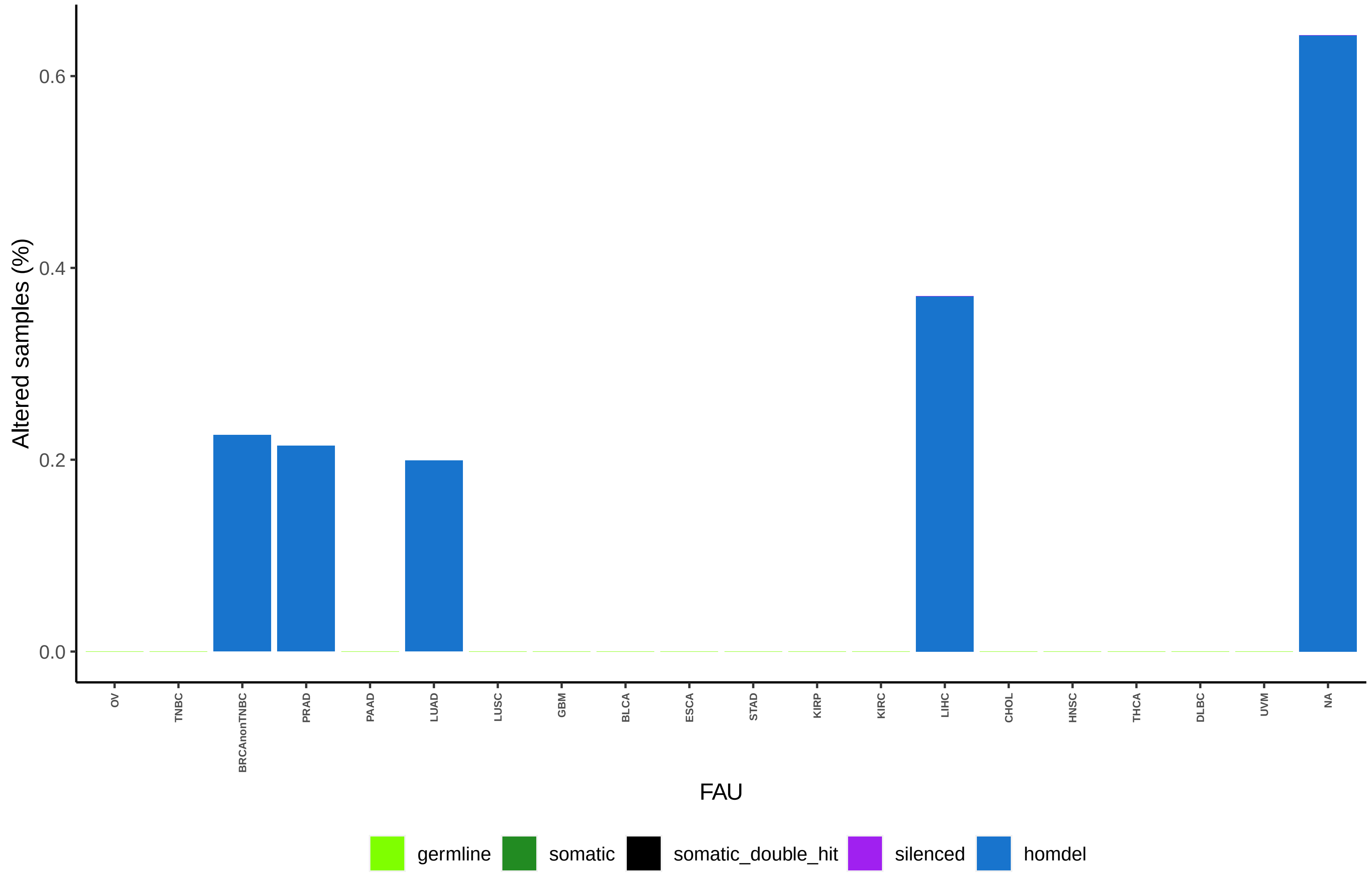
Altered samples (%)
0.0
0.2
0.4
0.6
OV TNBC BRCAnonTNBC PRAD PAAD LUAD LUSC GBM BLCA ESCA STAD KIRP KIRC LIHC CHOL HNSC THCA DLBC UVM NA
FAU
germline
somatic
somatic_double_hit
silenced
homdel

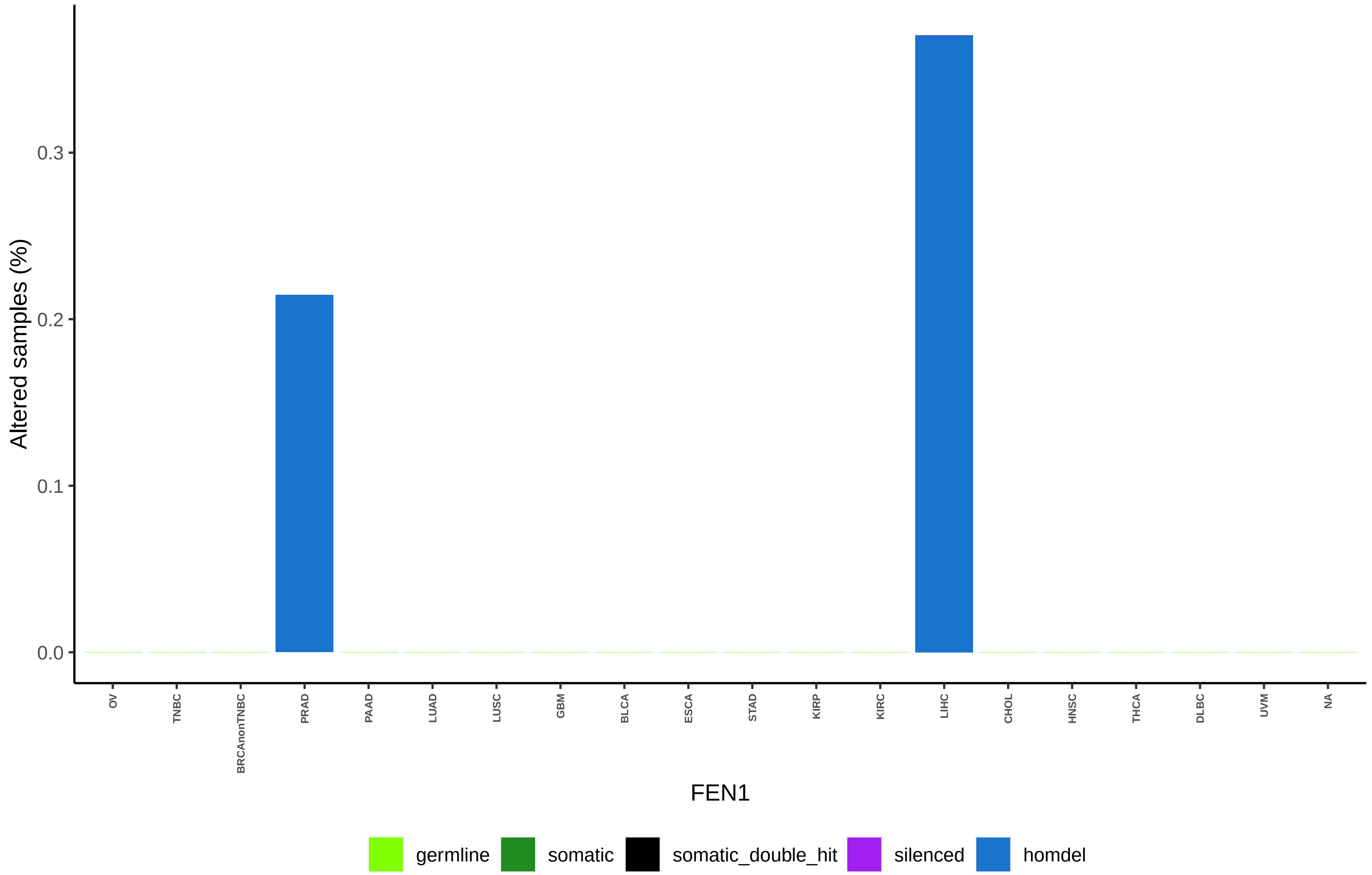
Altered samples (%)
0.0
0.1
0.2
0.3
OV TNBC BRCAnonTNBC PRAD PAAD LUAD LUSC GBM BLCA ESCA STAD KIRP KIRC LIHC CHOL HNSC THCA DLBC UVM NA
FEN1
germline
somatic
somatic_double_hit
silenced
homdel

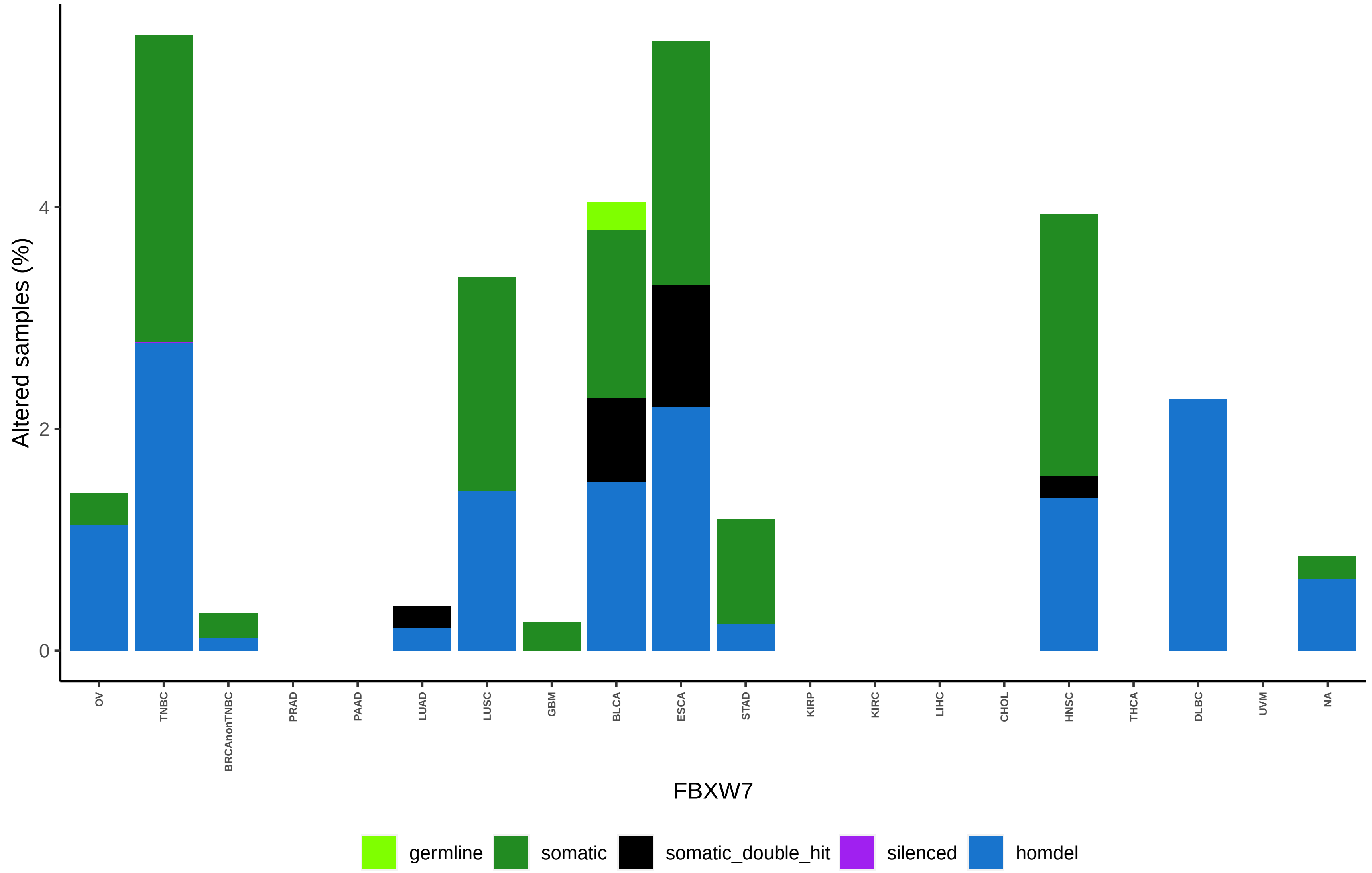
Altered samples (%)
0
2
4
OV TNBC BRCAnonTNBC PRAD PAAD LUAD LUSC GBM BLCA ESCA STAD KIRP KIRC LIHC CHOL HNSC THCA DLBC UVM NA
FBXW7
germline
somatic
somatic_double_hit
silenced
homdel

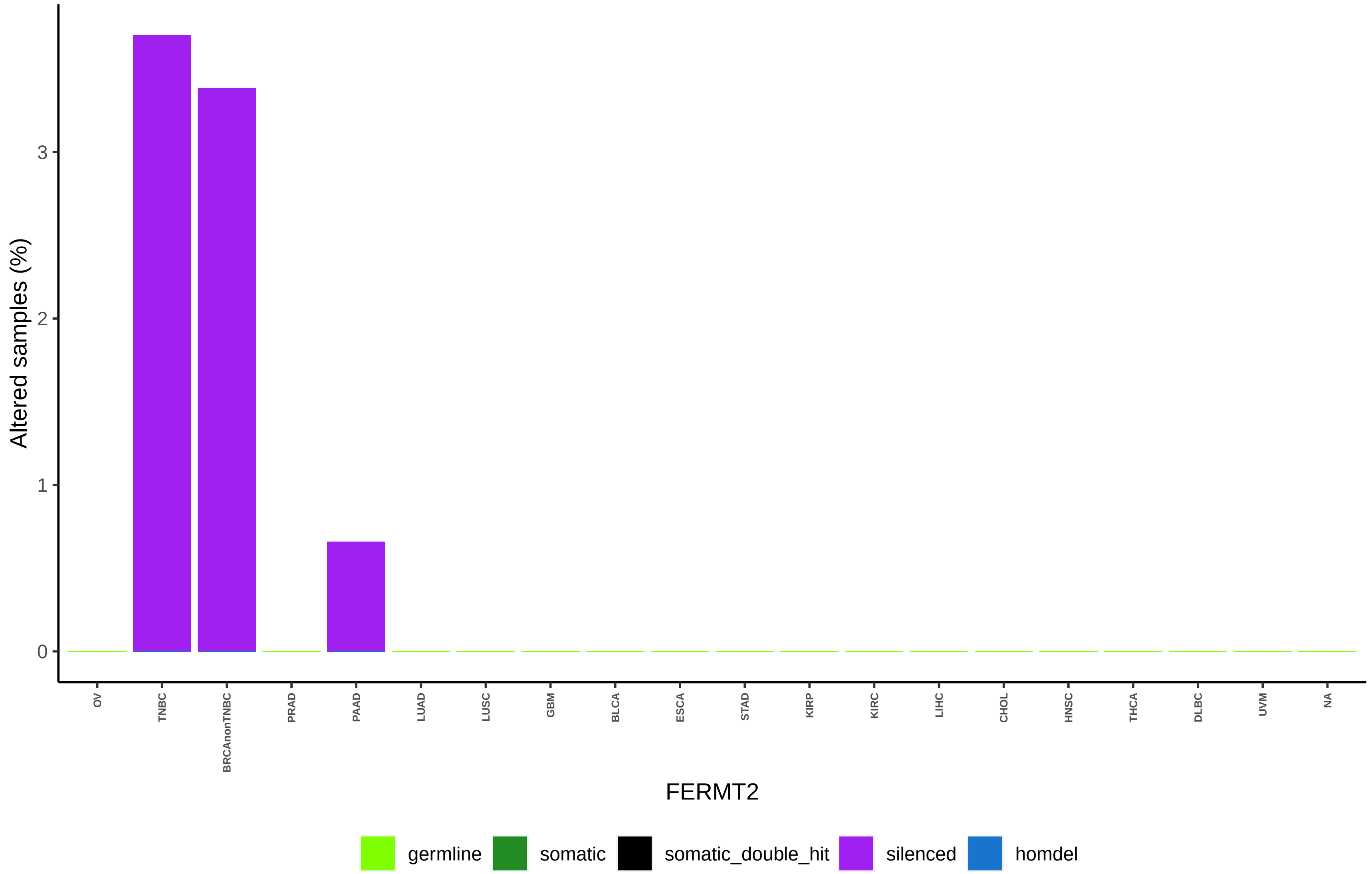
Altered samples (%)
0
1
2
3
OV TNBC BRCAnonTNBC PRAD PAAD LUAD LUSC GBM BLCA ESCA STAD KIRP KIRC LIHC CHOL HNSC THCA DLBC UVM NA
FERMT2
germline
somatic
somatic_double_hit
silenced
homdel

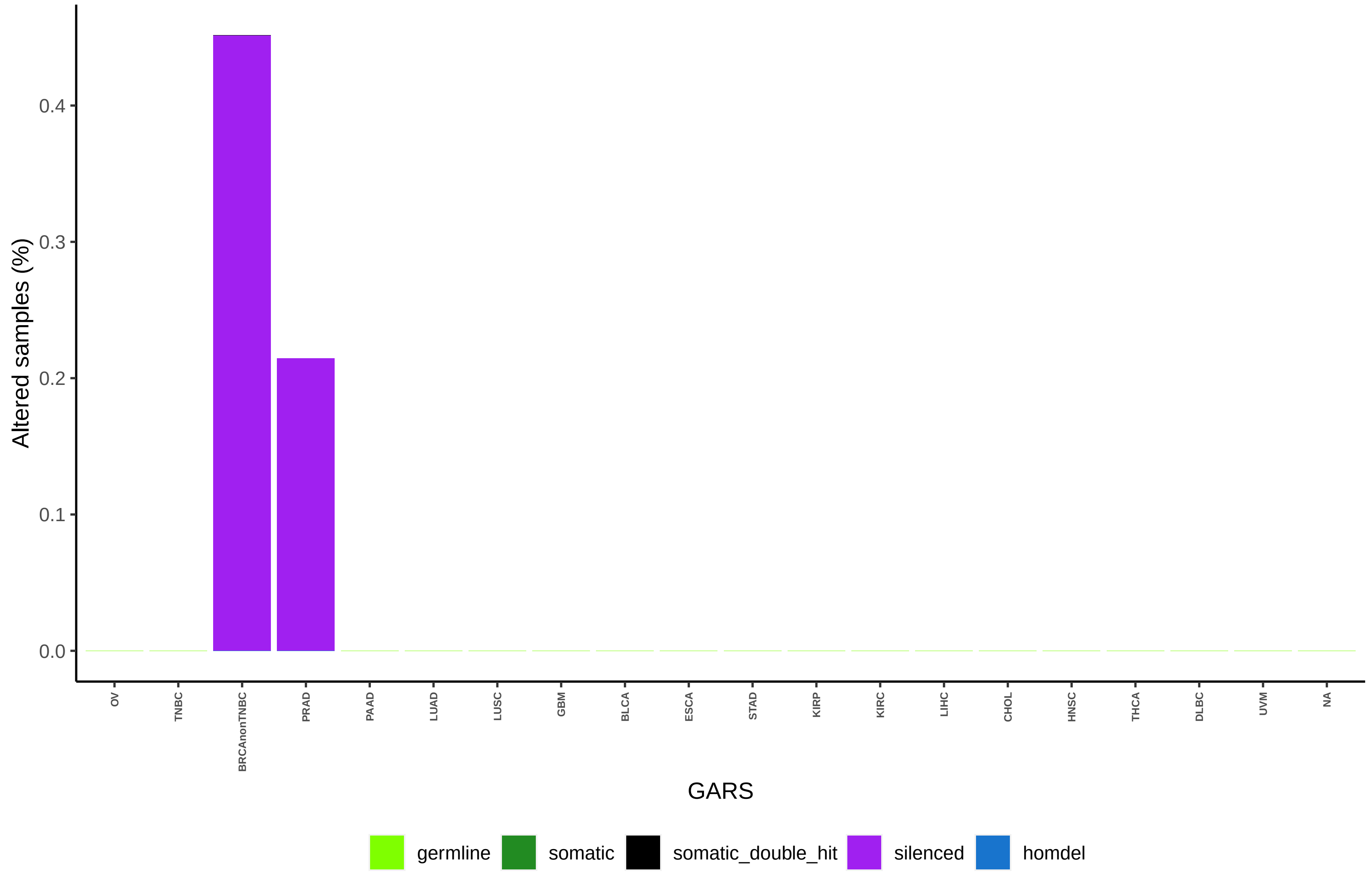
Altered samples (%)
0.0
0.1
0.2
0.3
0.4
OV
TNBC
BRCAnonTNBC
PRAD
PAAD
LUAD
LUSC
GBM
BLCA
ESCA
STAD
KIRP
KIRC
LIHC
CHOL
HNSC
THCA
DLBC
UVM
NA
GARS
germline
somatic
somatic_double_hit
silenced
homdel

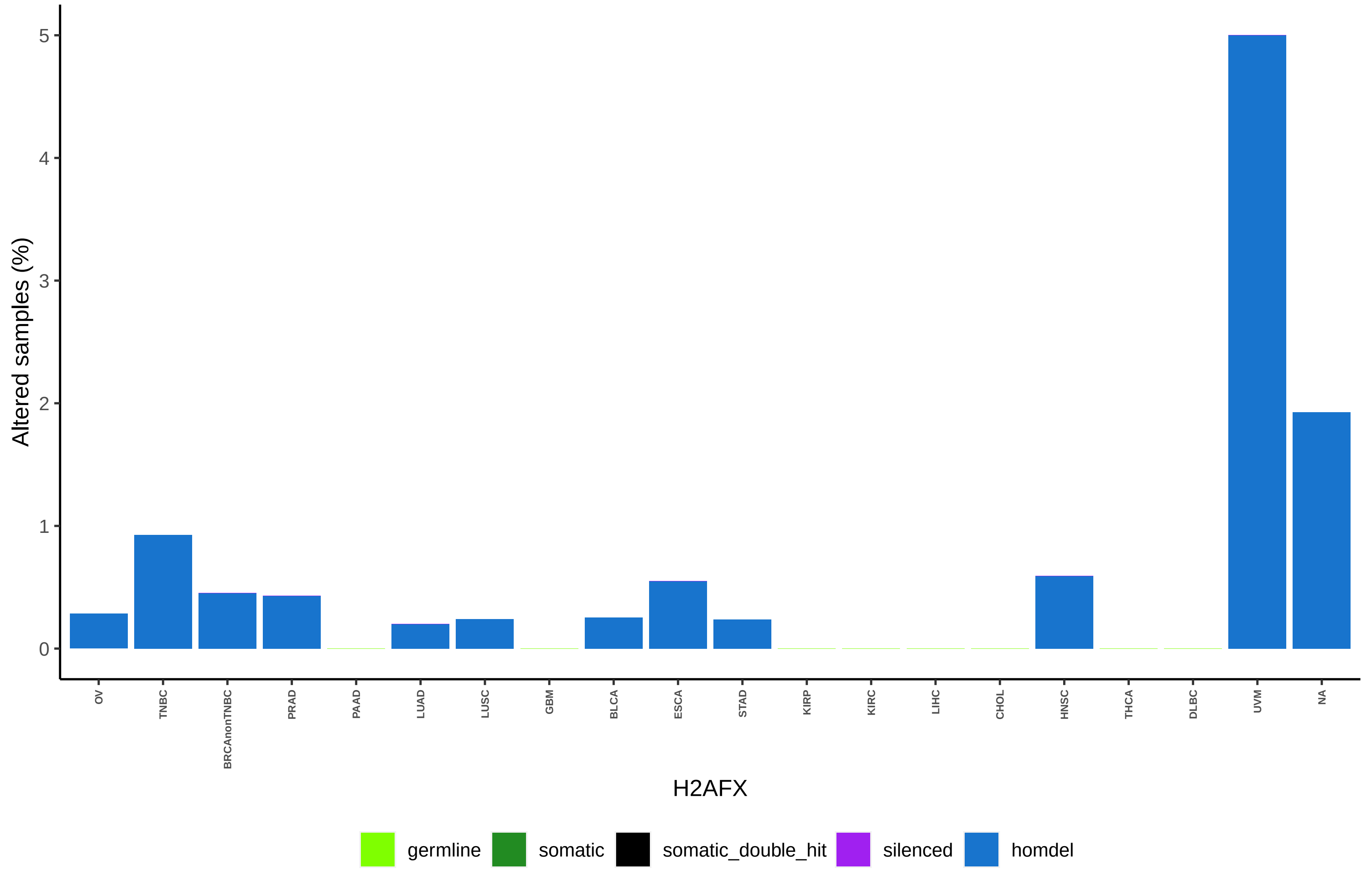
Altered samples (%)
0
1
2
3
4
5
OV
TNBC
BRCAnonTNBC
PRAD
PAAD
LUAD
LUSC
GBM
BLCA
ESCA
STAD
KIRP
KIRC
LIHC
CHOL
HNSC
THCA
DLBC
UVM
NA
H2AFX
germline
somatic
somatic_double_hit
silenced
homdel

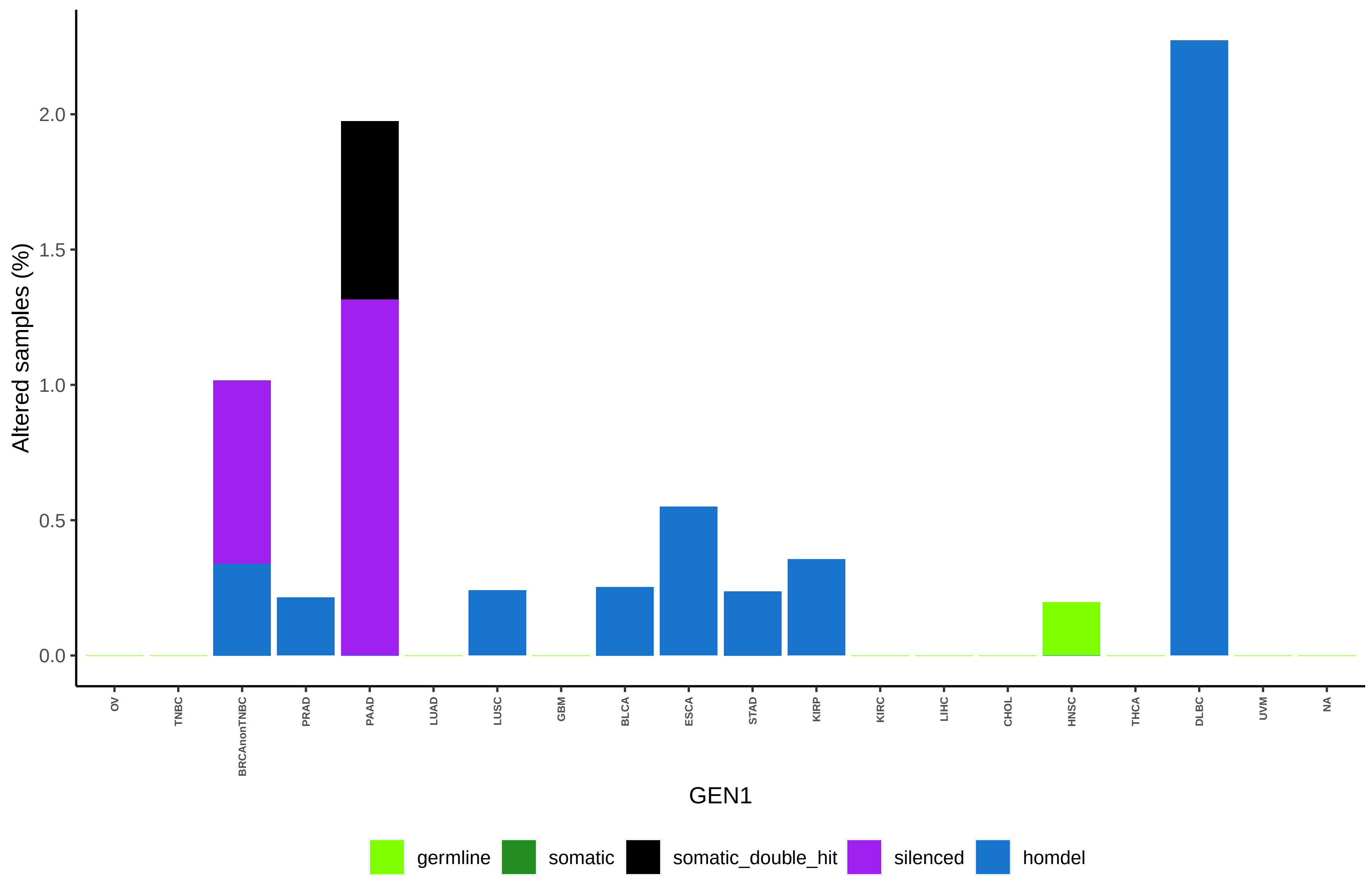
Altered samples (%)
0.0
0.5
1.0
1.5
2.0
OV
TNBC
BRCAnonTNBC
PRAD
PAAD
LUAD
LUSC
GBM
BLCA
ESCA
STAD
KIRP
KIRC
LIHC
CHOL
HNSC
THCA
DLBC
UVM
NA
GEN1
germline
somatic
somatic_double_hit
silenced
homdel

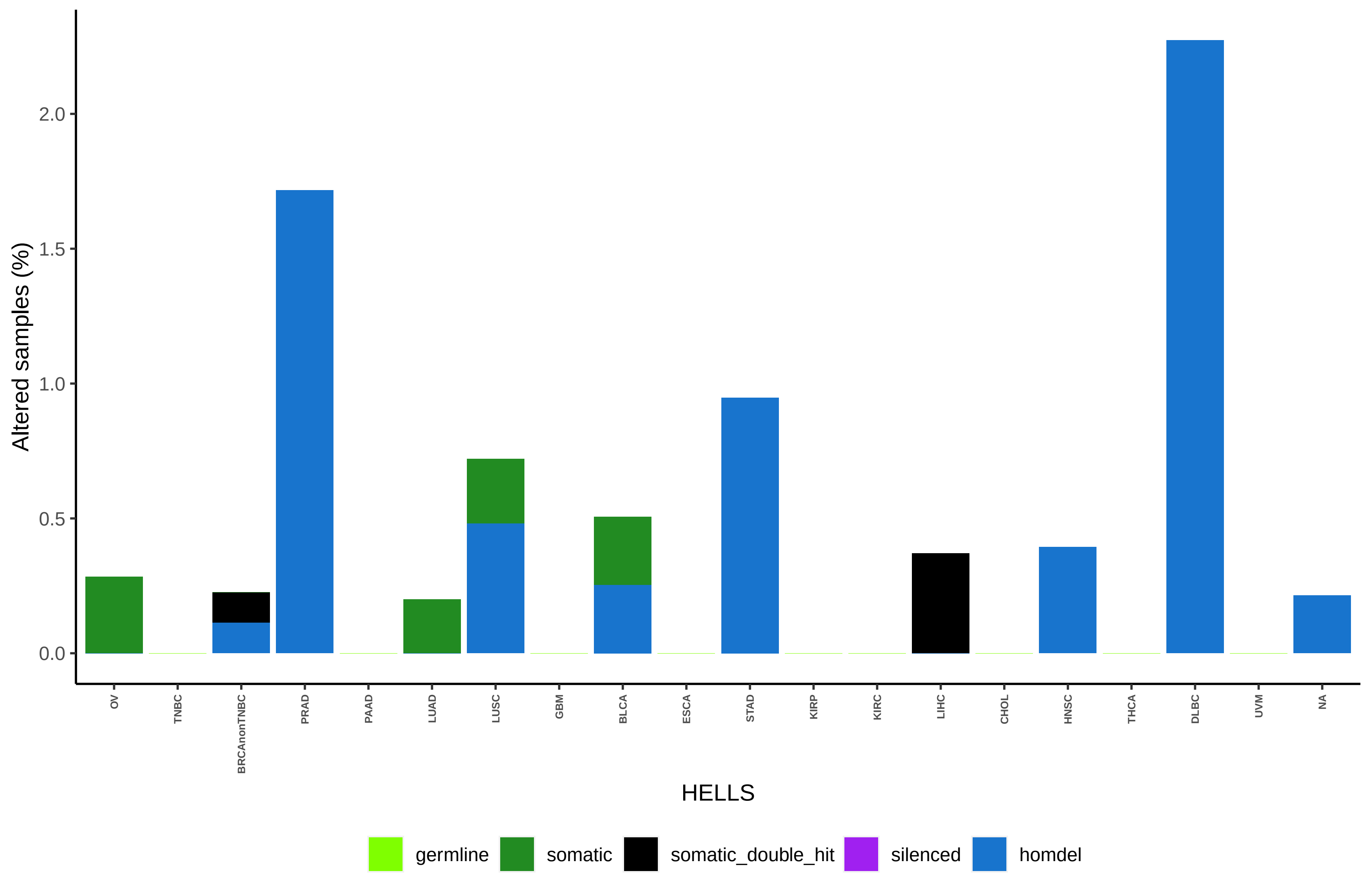
Altered samples (%)
0.0
0.5
1.0
1.5
2.0
OV
TNBC
BRCAnonTNBC
PRAD
PAAD
LUAD
LUSC
GBM
BLCA
ESCA
STAD
KIRP
KIRC
LIHC
CHOL
HNSC
THCA
DLBC
UVM
NA
HELLS
germline
somatic
somatic_double_hit
silenced
homdel

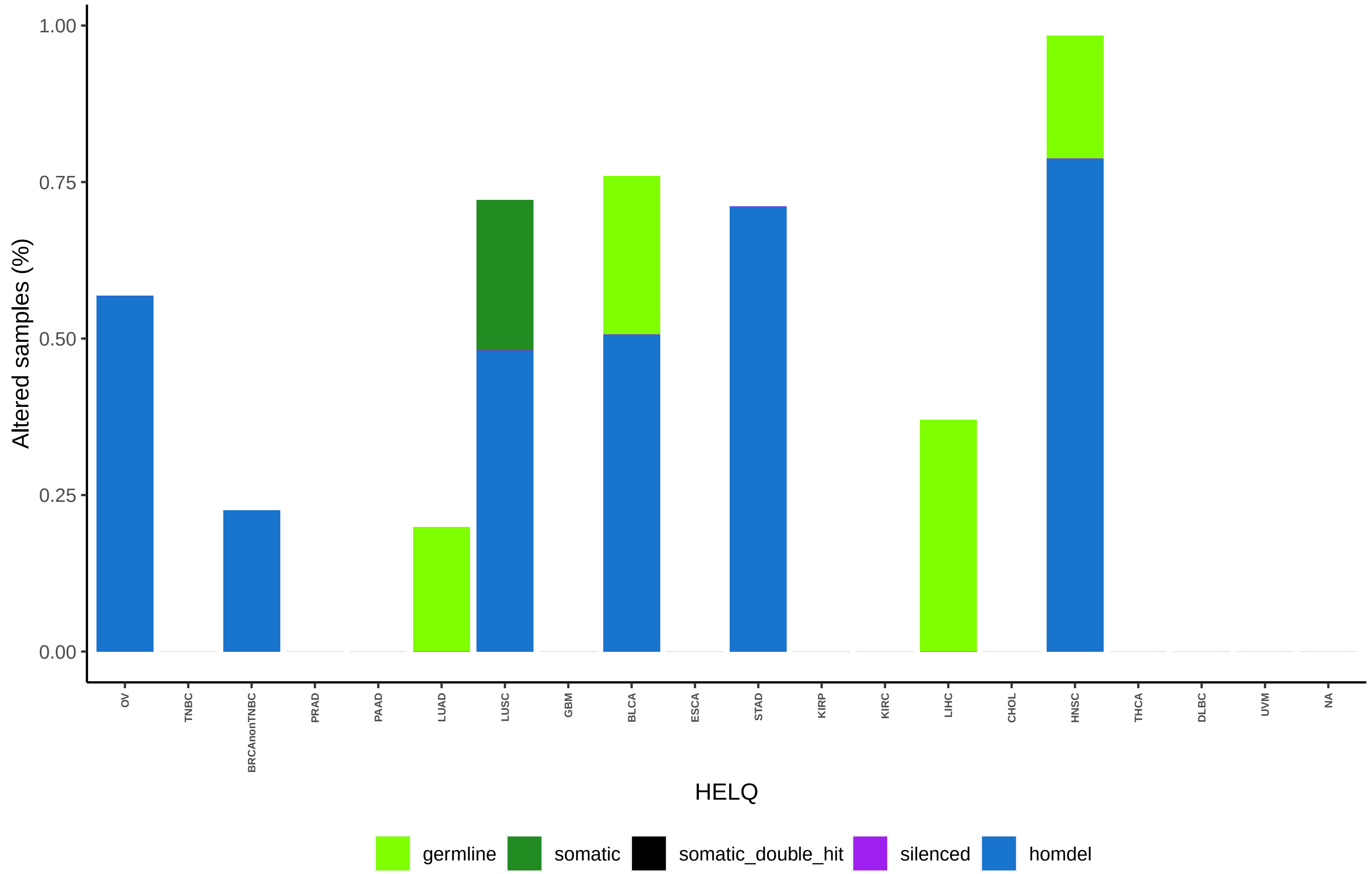

Altered samples (%)
1.00
0.75
0.50
0.25
0.00
OV
TNBC
BRCAnonTNBC
PRAD
PAAD
LUAD
LUSC
GBM
BLCA
ESCA
STAD
KIRP
KIRC
LIHC
CHOL
HNSC
THCA
DLBC
UVM
NA
HELQ
germline
somatic
somatic_double_hit
silenced
homdel


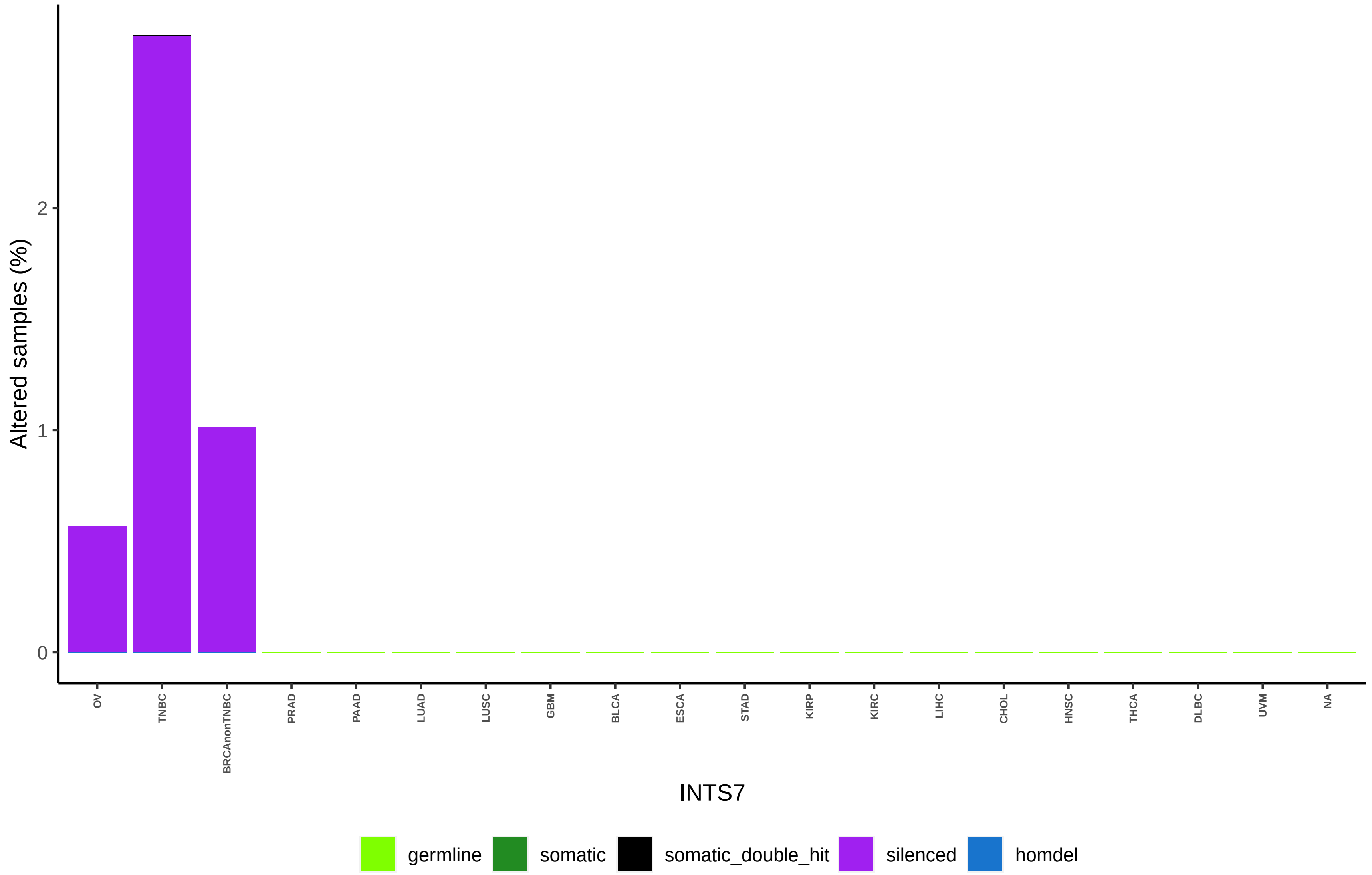

Altered samples (%)
2
1
0
OV
TNBC
BRCAnonTNBC
PRAD
PAAD
LUAD
LUSC
GBM
BLCA
ESCA
STAD
KIRP
KIRC
LIHC
CHOL
HNSC
THCA
DLBC
UVM
NA
INTS7
germline
somatic
somatic_double_hit
silenced
homdel


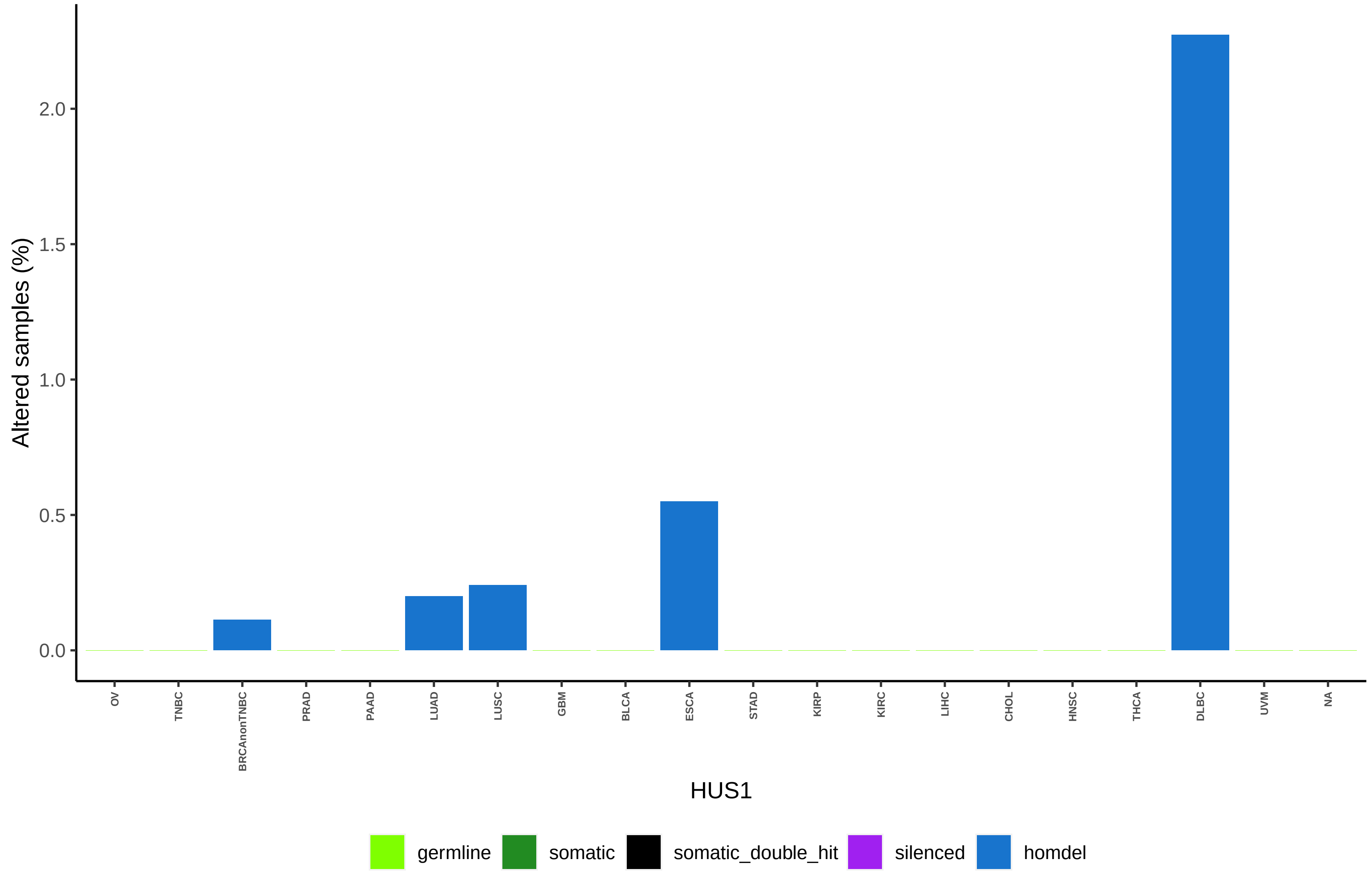

Altered samples (%)
2.0
1.5
1.0
0.5
0.0
OV
TNBC
BRCAnonTNBC
PRAD
PAAD
LUAD
LUSC
GBM
BLCA
ESCA
STAD
KIRP
KIRC
LIHC
CHOL
HNSC
THCA
DLBC
UVM
NA
HUS1
germline
somatic
somatic_double_hit
silenced
homdel


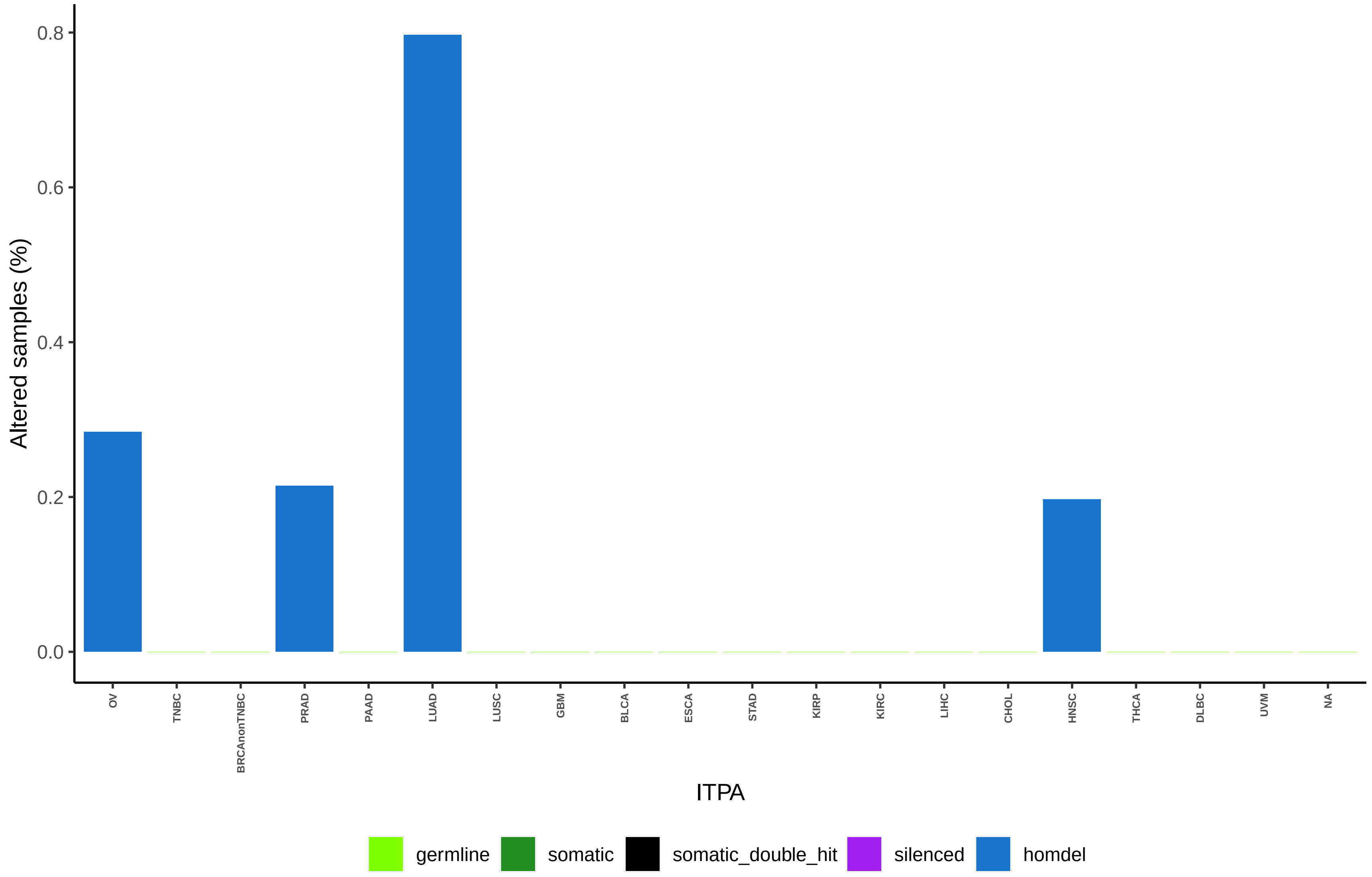

Altered samples (%)
0.8
0.6
0.4
0.2
0.0
OV
TNBC
BRCAnonTNBC
PRAD
PAAD
LUAD
LUSC
GBM
BLCA
ESCA
STAD
KIRP
KIRC
LIHC
CHOL
HNSC
THCA
DLBC
UVM
NA
ITPA
germline
somatic
somatic_double_hit
silenced
homdel

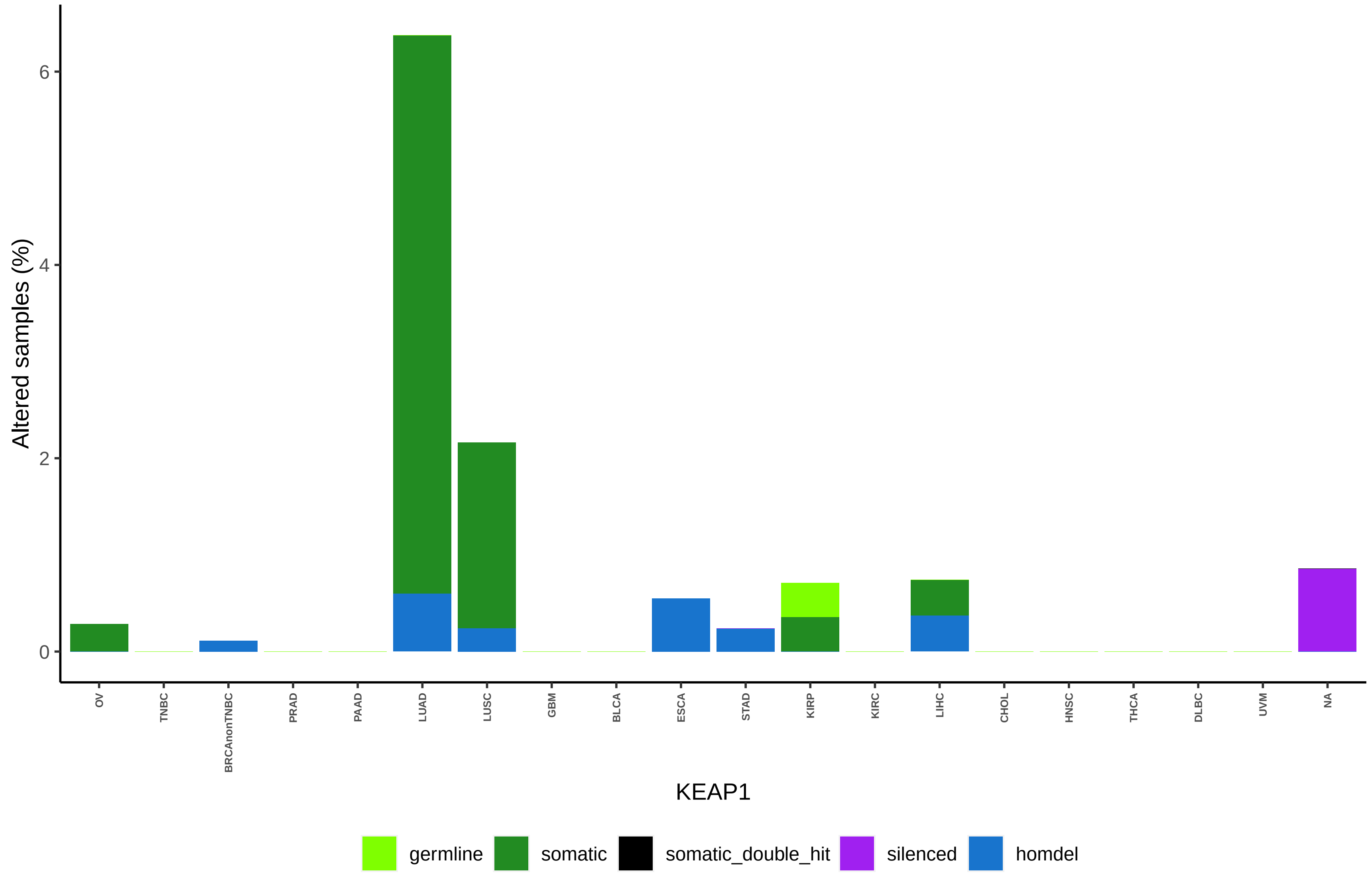
Altered samples (%)
0
2
4
6
OV
TNBC
BRCAnonTNBC
PRAD
PAAD
LUAD
LUSC
GBM
BLCA
ESCA
STAD
KIRP
KIRC
LIHC
CHOL
HNSC
THCA
DLBC
UVM
NA
KEAP1
germline
somatic
somatic_double_hit
silenced
homdel

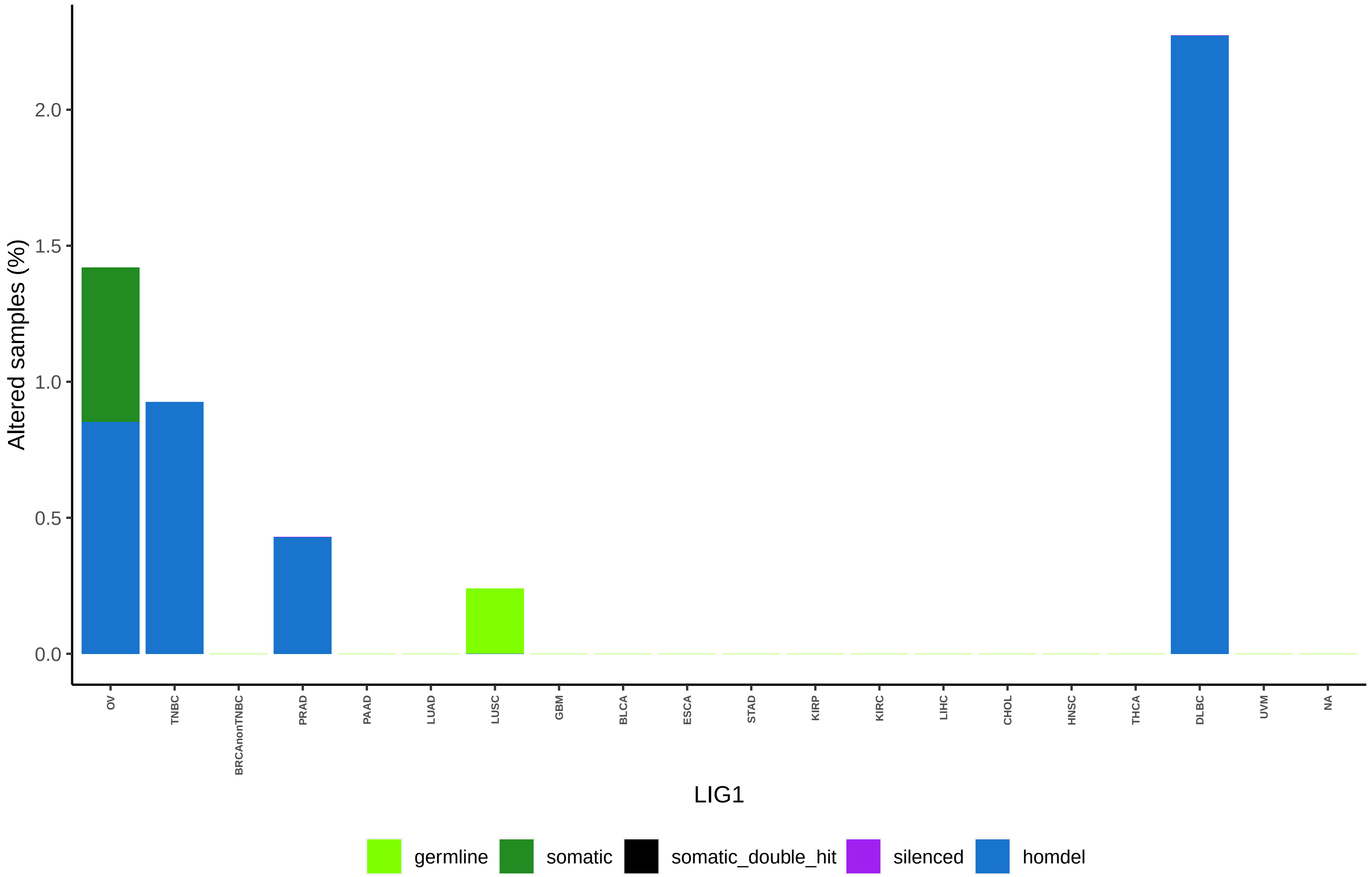
Altered samples (%)
0.0
0.5
1.0
1.5
2.0
OV
TNBC
BRCAnonTNBC
PRAD
PAAD
LUAD
LUSC
GBM
BLCA
ESCA
STAD
KIRP
KIRC
LIHC
CHOL
HNSC
THCA
DLBC
UVM
NA
LIG1
germline
somatic
somatic_double_hit
silenced
homdel

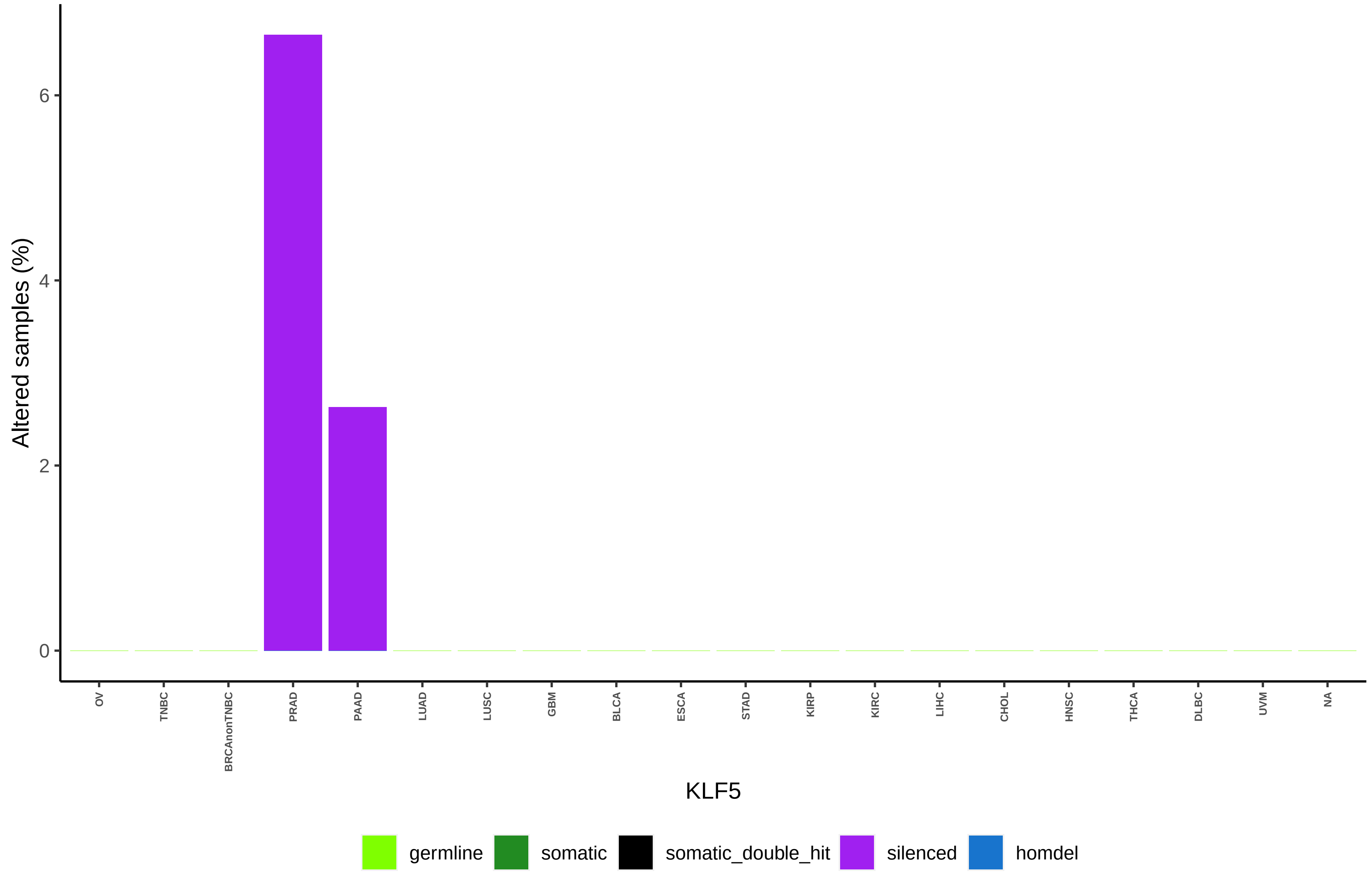
Altered samples (%)
0
2
4
6
OV
TNBC
BRCAnonTNBC
PRAD
PAAD
LUAD
LUSC
GBM
BLCA
ESCA
STAD
KIRP
KIRC
LIHC
CHOL
HNSC
THCA
DLBC
UVM
NA
KLF5
germline
somatic
somatic_double_hit
silenced
homdel

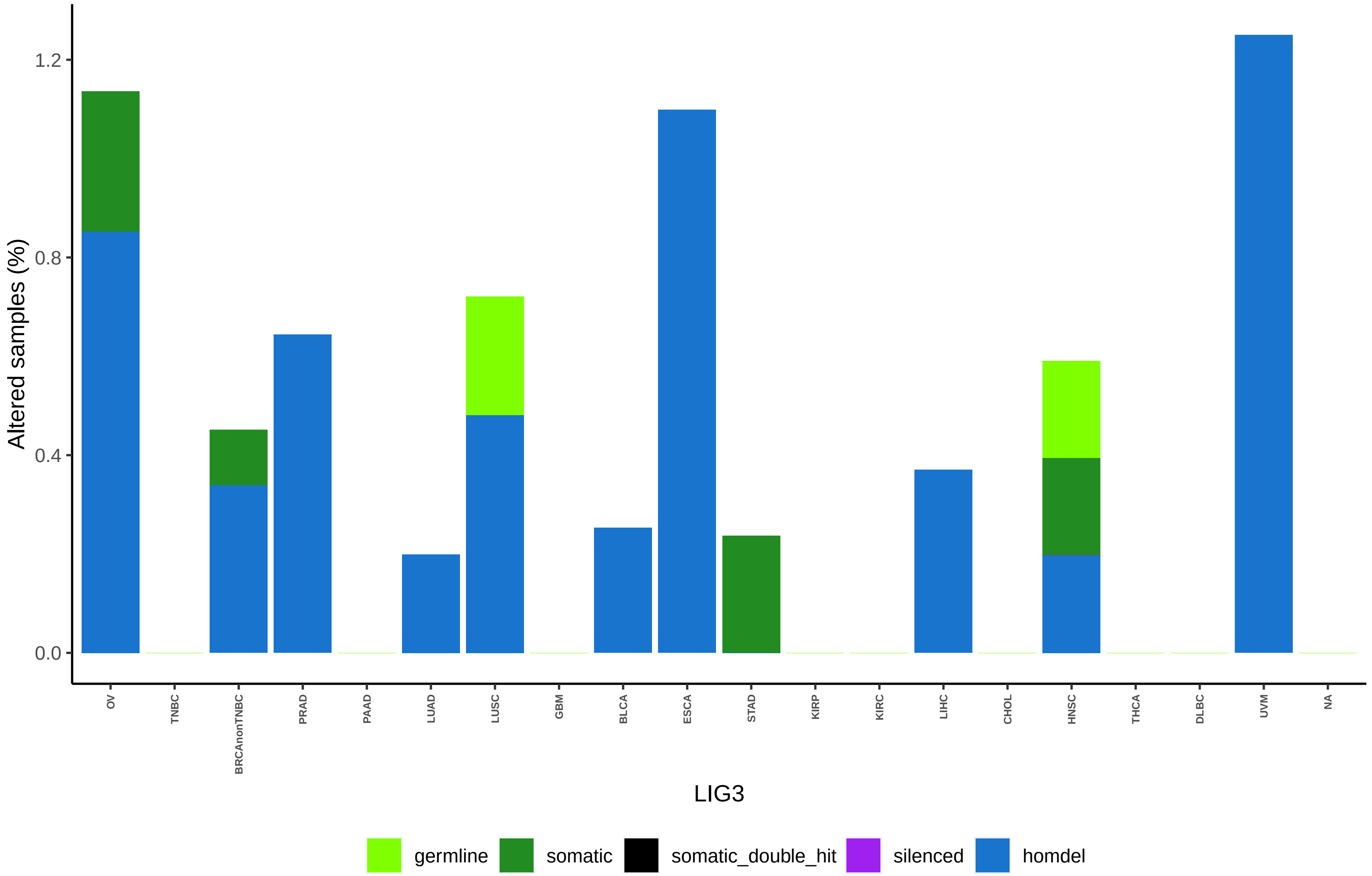
Altered samples (%)
0.0
0.4
0.8
1.2
OV
TNBC
BRCAnonTNBC
PRAD
PAAD
LUAD
LUSC
GBM
BLCA
ESCA
STAD
KIRP
KIRC
LIHC
CHOL
HNSC
THCA
DLBC
UVM
NA
LIG3
germline
somatic
somatic_double_hit
silenced
homdel

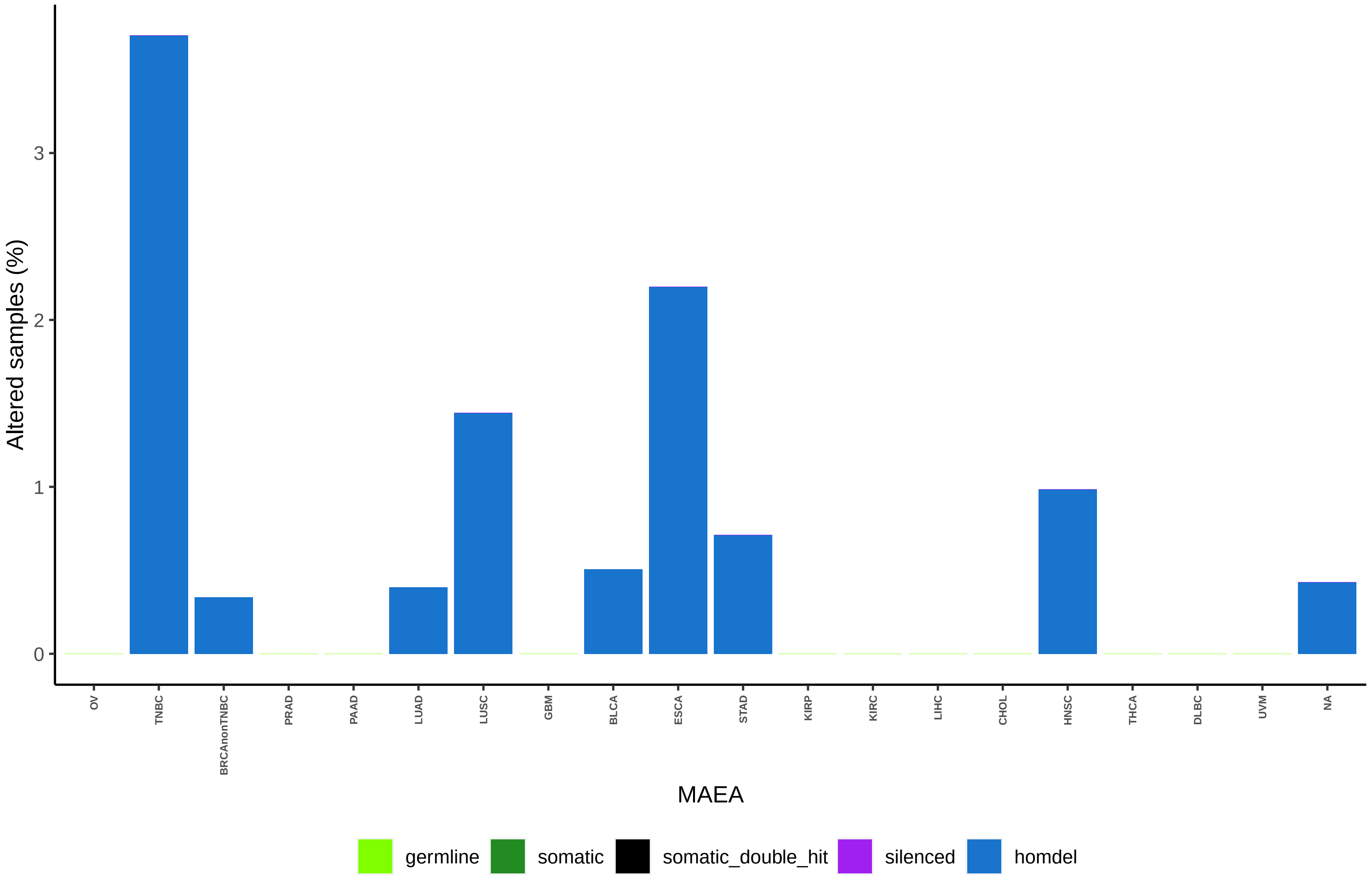
Altered samples (%)
0
1
2
3
OV
TNBC
BRCAnonTNBC
PRAD
PAAD
LUAD
LUSC
GBM
BLCA
ESCA
STAD
KIRP
KIRC
LIHC
CHOL
HNSC
THCA
DLBC
UVM
NA
MAEA
germline
somatic
somatic_double_hit
silenced
homdel

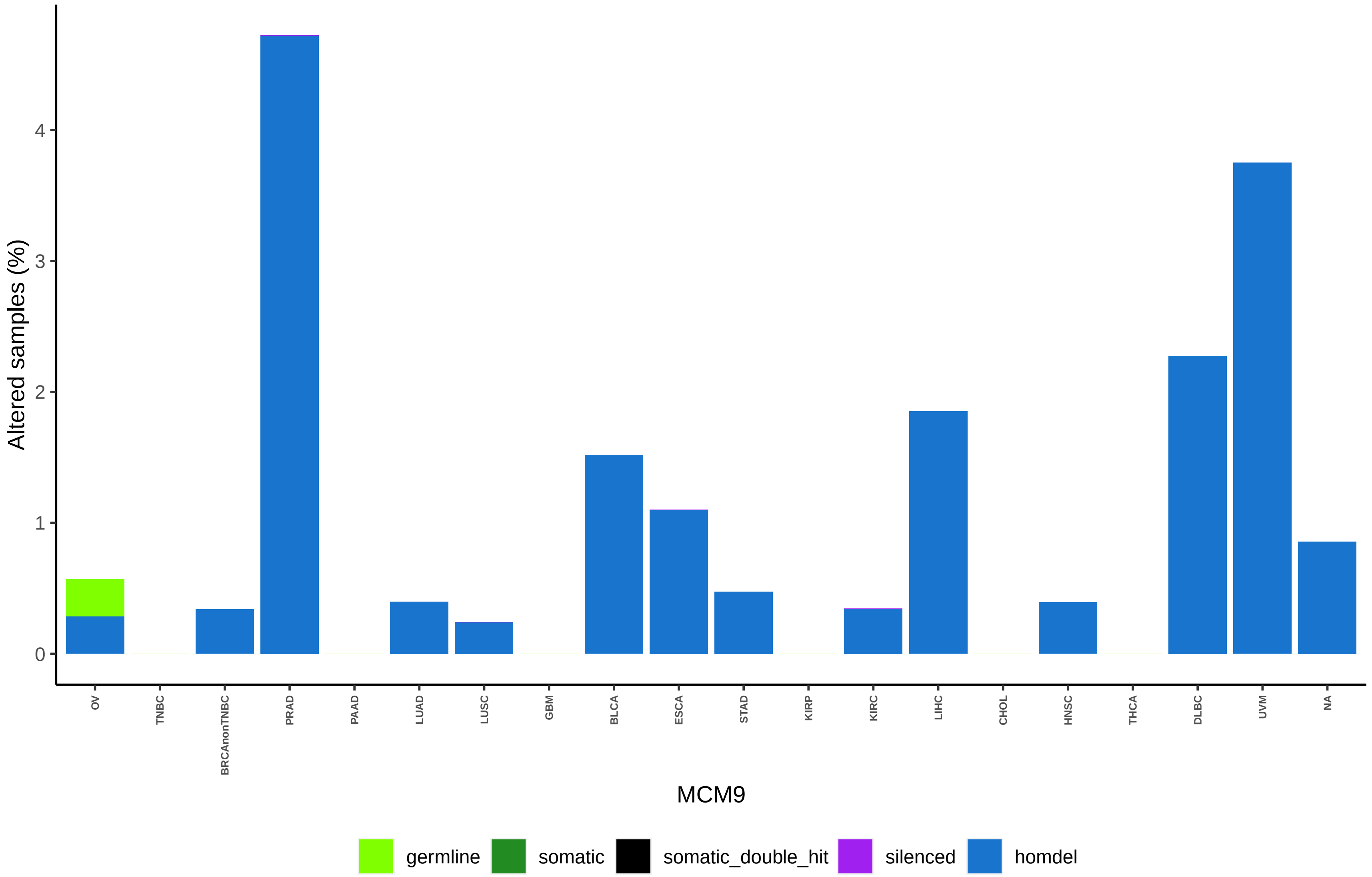
Altered samples (%)
0
1
2
3
4
OV
TNBC
BRCAnonTNBC
PRAD
PAAD
LUAD
LUSC
GBM
BLCA
ESCA
STAD
KIRP
KIRC
LIHC
CHOL
HNSC
THCA
DLBC
UVM
NA
MCM9
germline
somatic
somatic_double_hit
silenced
homdel

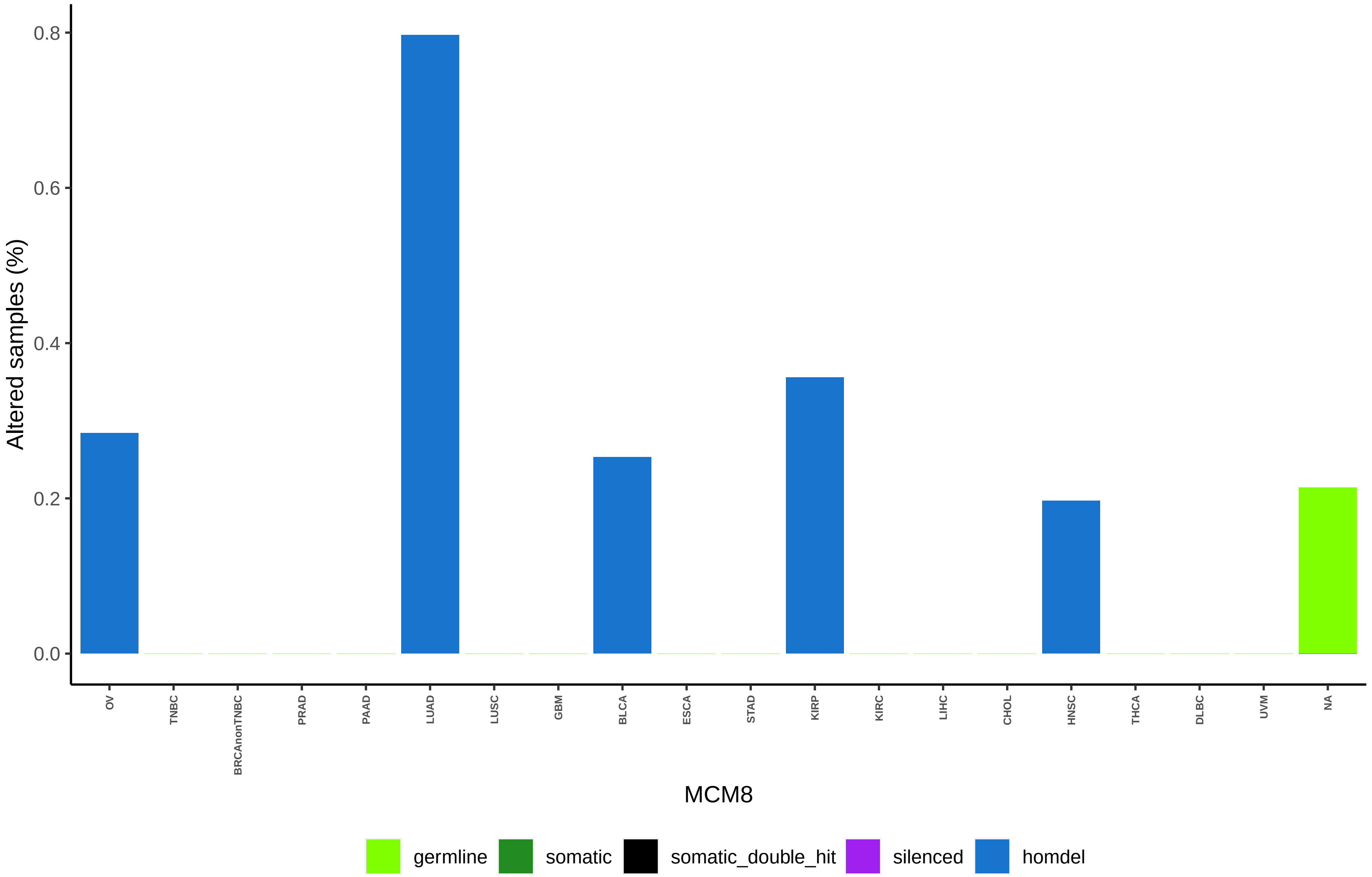
Altered samples (%)
0.0
0.2
0.4
0.6
0.8
OV
TNBC
BRCAnonTNBC
PRAD
PAAD
LUAD
LUSC
GBM
BLCA
ESCA
STAD
KIRP
KIRC
LIHC
CHOL
HNSC
THCA
DLBC
UVM
NA
MCM8
germline
somatic
somatic_double_hit
silenced
homdel

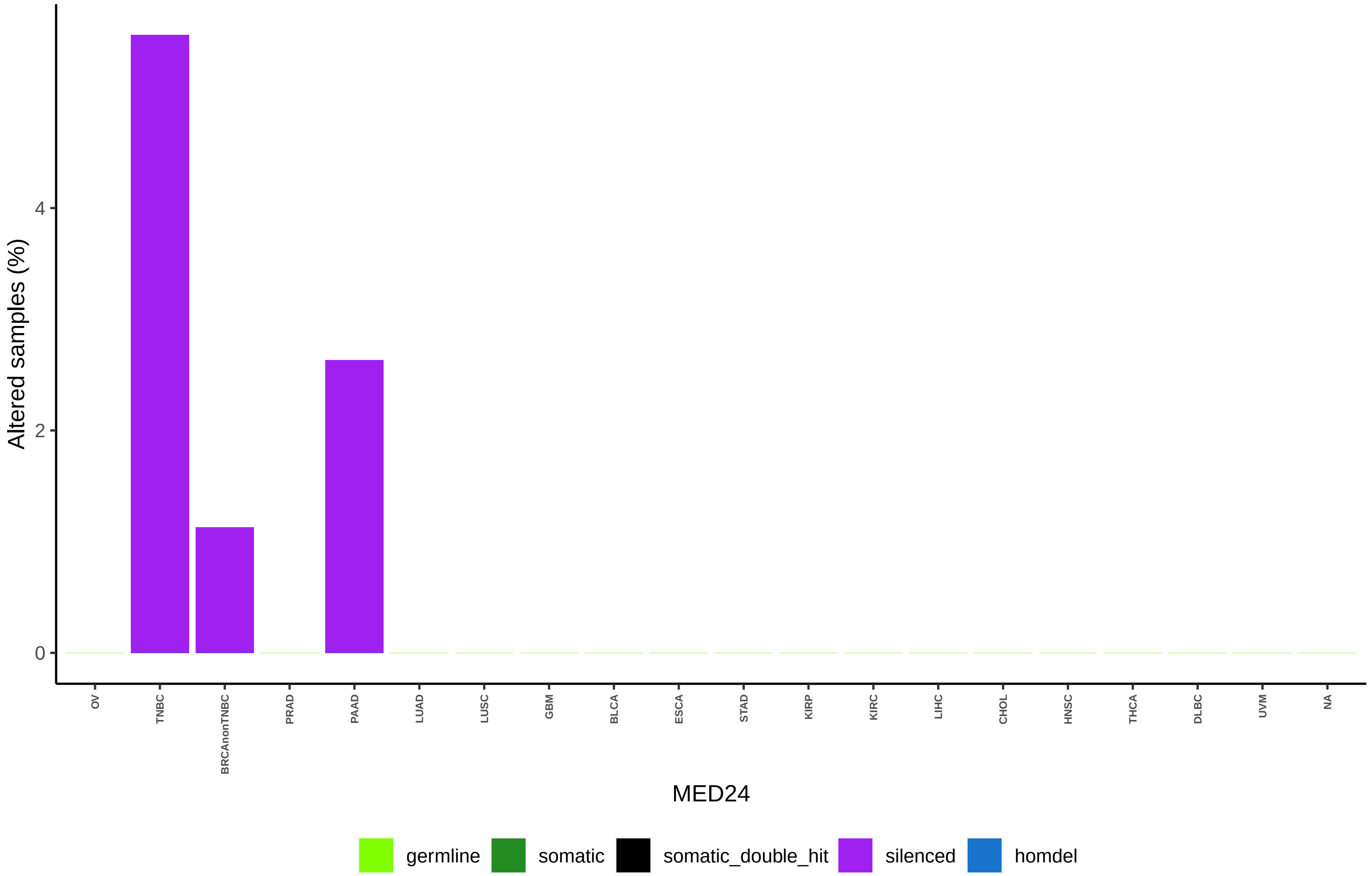
Altered samples (%)
0
2
4
OV
TNBC
BRCAnonTNBC
PRAD
PAAD
LUAD
LUSC
GBM
BLCA
ESCA
STAD
KIRP
KIRC
LIHC
CHOL
HNSC
THCA
DLBC
UVM
NA
MED24
germline
somatic
somatic_double_hit
silenced
homdel

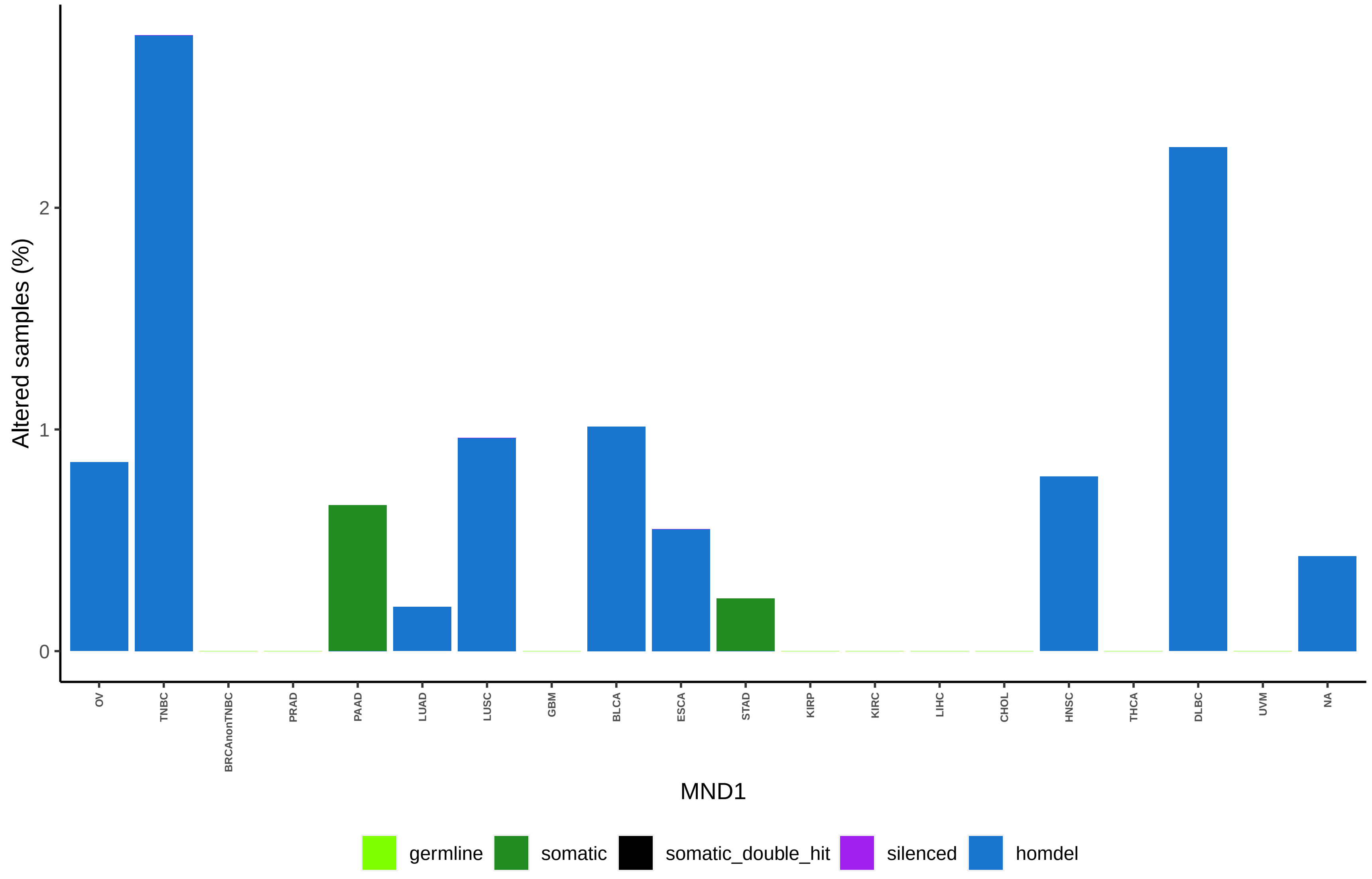
Altered samples (%)
0
1
2
OV
TNBC
BRCAnonTNBC
PRAD
PAAD
LUAD
LUSC
GBM
BLCA
ESCA
STAD
KIRP
KIRC
LIHC
CHOL
HNSC
THCA
DLBC
UVM
NA
MND1
germline
somatic
somatic_double_hit
silenced
homdel

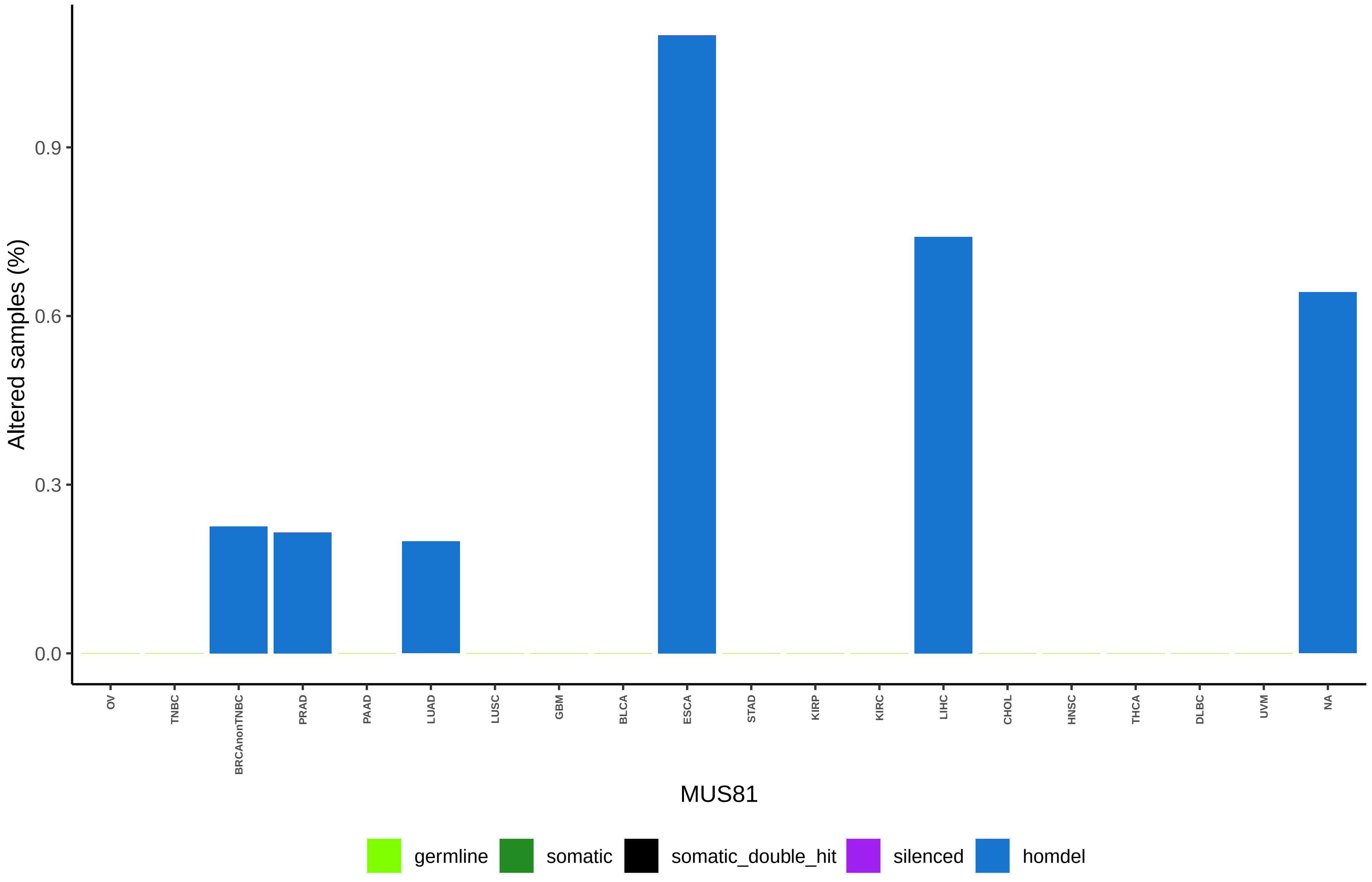
Altered samples (%)
0.0
0.3
0.6
0.9
OV
TNBC
BRCAnonTNBC
PRAD
PAAD
LUAD
LUSC
GBM
BLCA
ESCA
STAD
KIRP
KIRC
LIHC
CHOL
HNSC
THCA
DLBC
UVM
NA
MUS81
germline
somatic
somatic_double_hit
silenced
homdel

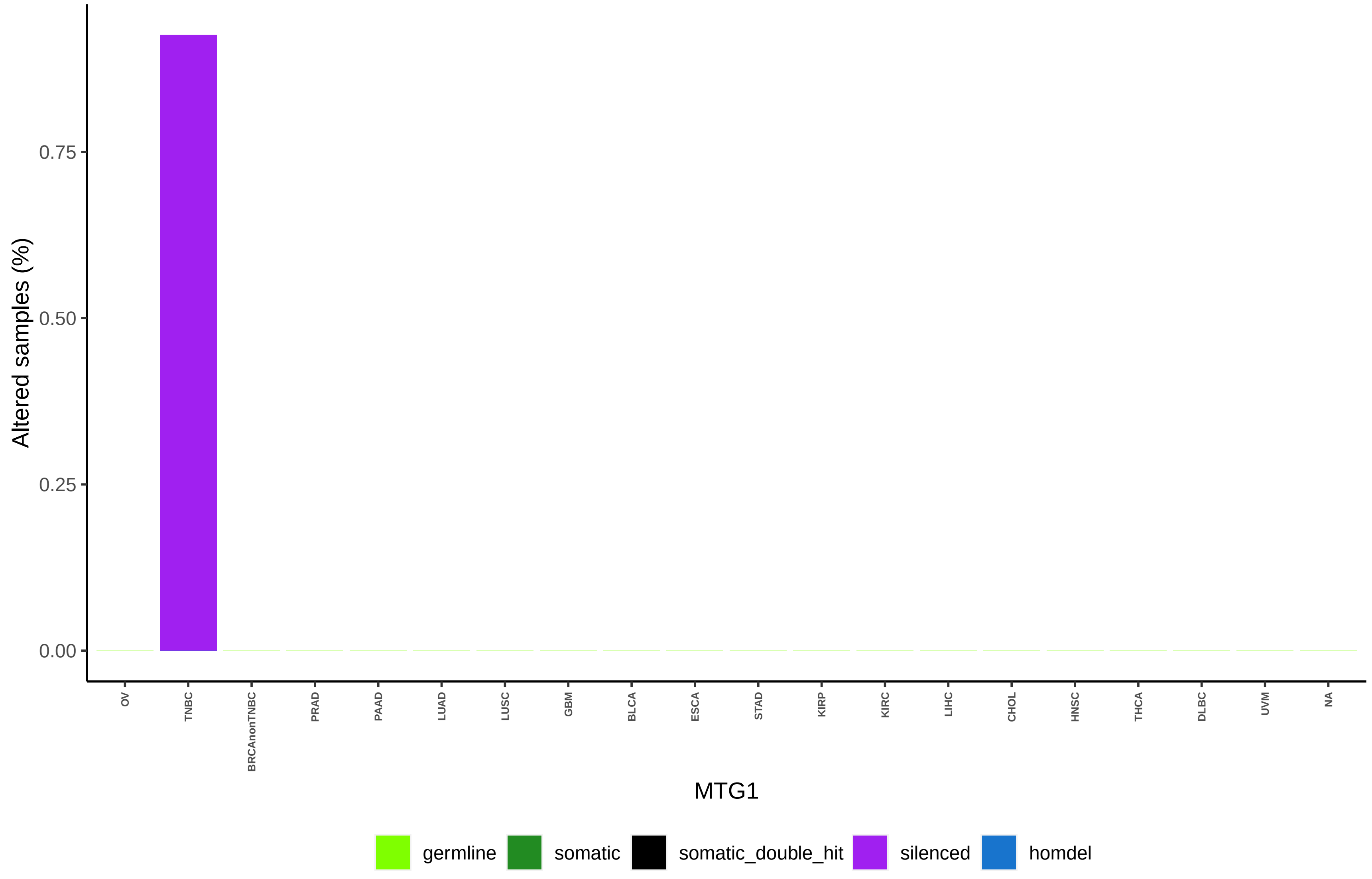
Altered samples (%)
0.00
0.25
0.50
0.75
OV
TNBC
BRCAnonTNBC
PRAD
PAAD
LUAD
LUSC
GBM
BLCA
ESCA
STAD
KIRP
KIRC
LIHC
CHOL
HNSC
THCA
DLBC
UVM
NA
MTG1
germline
somatic
somatic_double_hit
silenced
homdel

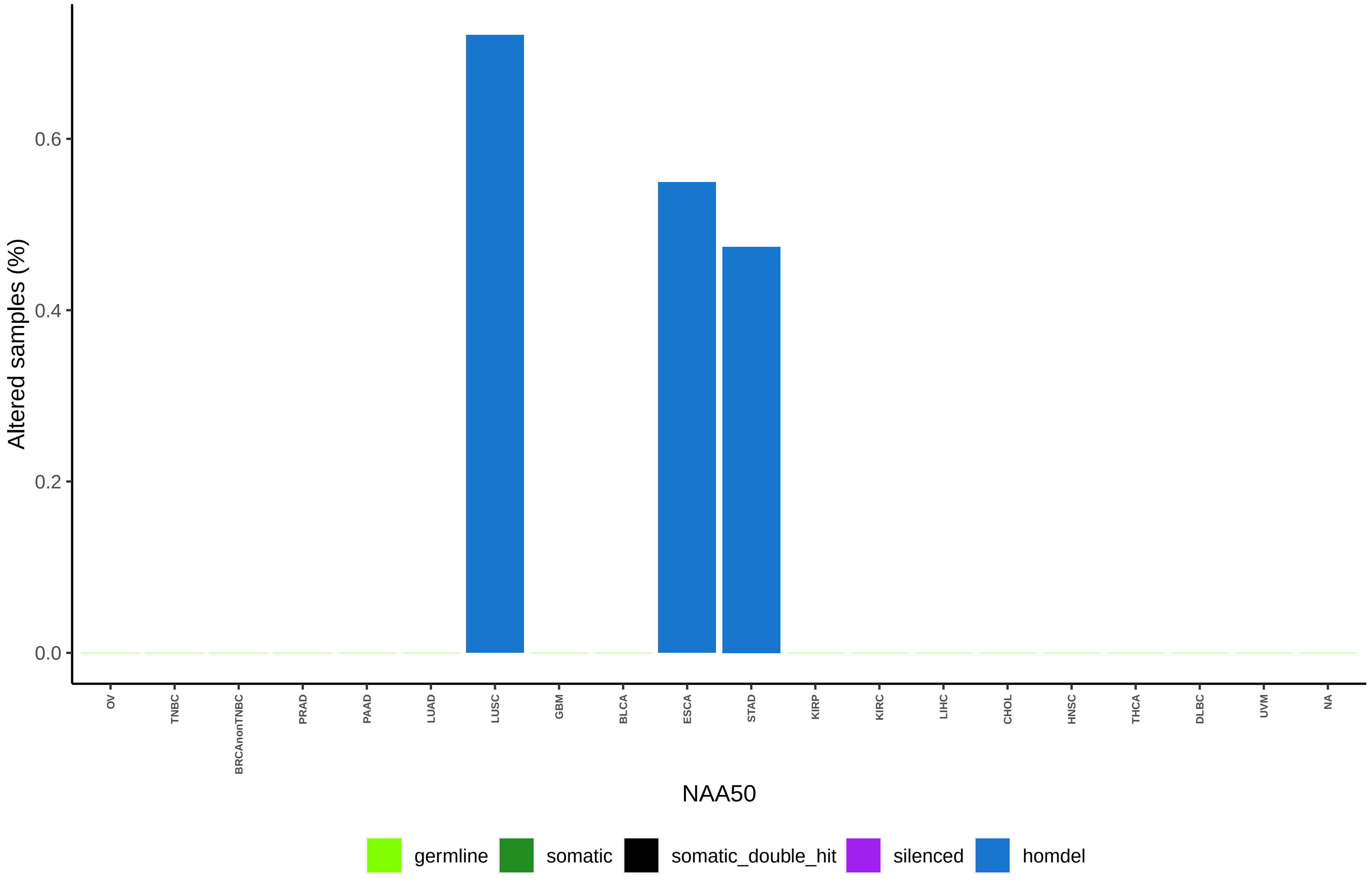
Altered samples (%)
0.0
0.2
0.4
0.6
OV
TNBC
BRCAnonTNBC
PRAD
PAAD
LUAD
LUSC
GBM
BLCA
ESCA
STAD
KIRP
KIRC
LIHC
CHOL
HNSC
THCA
DLBC
UVM
NA
NAA50
germline
somatic
somatic_double_hit
silenced
homdel

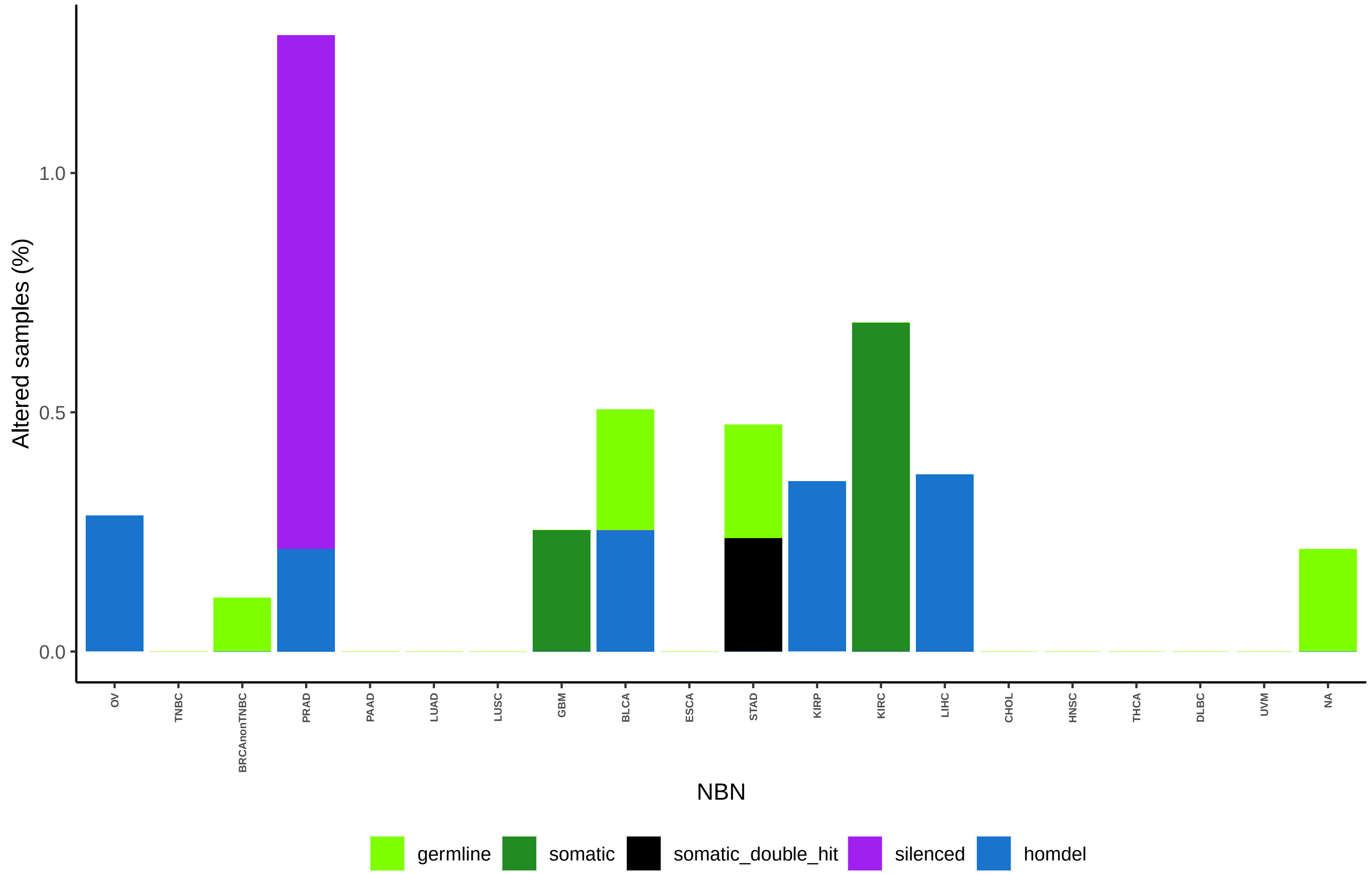
Altered samples (%)
0.0
0.5
1.0
OV TNBC BRCAnonTNBC PRAD PAAD LUAD LUSC GBM BLCA ESCA STAD KIRP KIRC LIHC CHOL HNSC THCA DLBC UVM NA
NBN
germline somatic somatic_double_hit silenced homdel

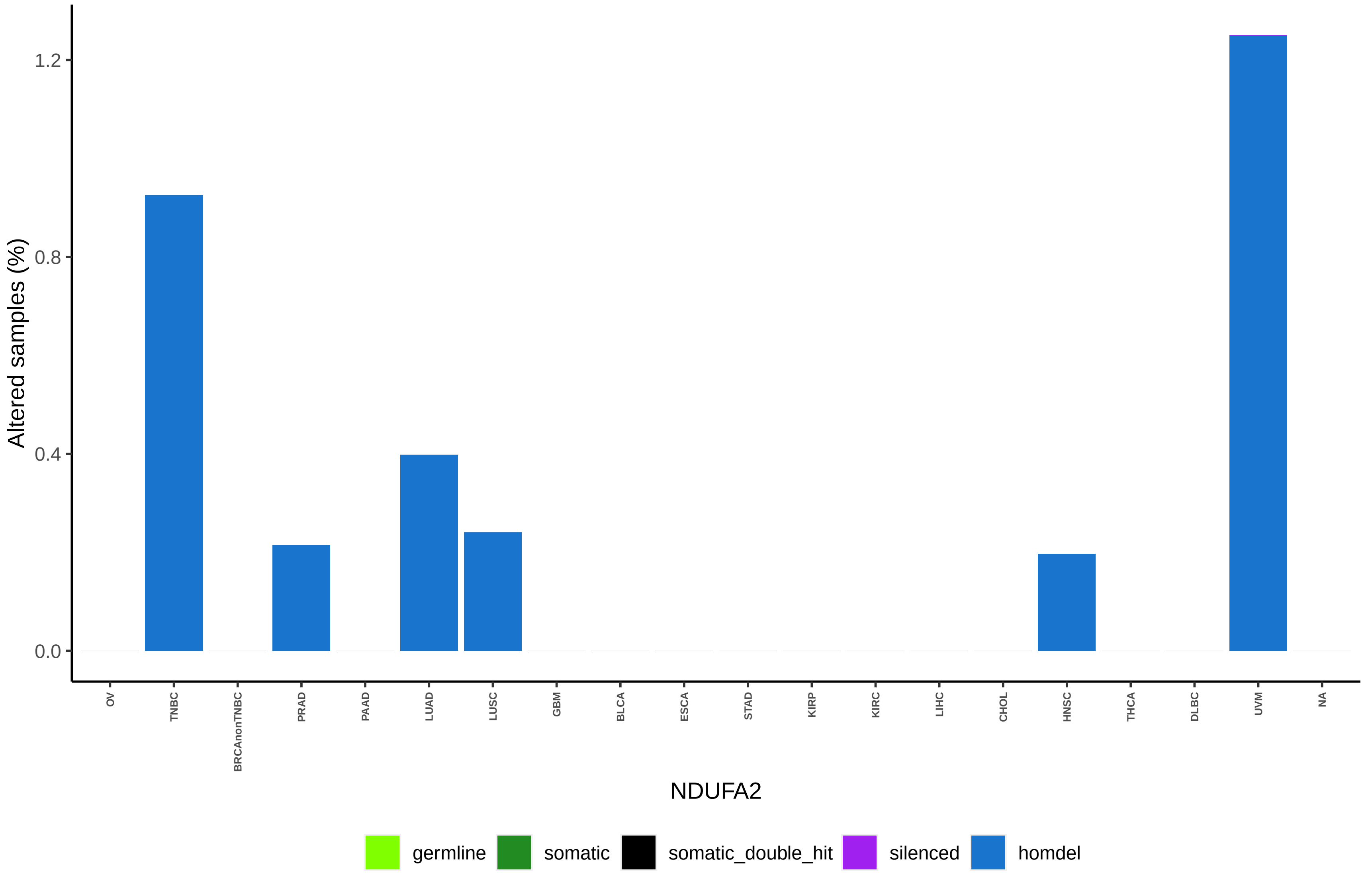
Altered samples (%)
0.0
0.4
0.8
1.2
OV TNBC BRCAnonTNBC PRAD PAAD LUAD LUSC GBM BLCA ESCA STAD KIRP KIRC LIHC CHOL HNSC THCA DLBC UVM NA
NDUFA2
germline somatic somatic_double_hit silenced homdel

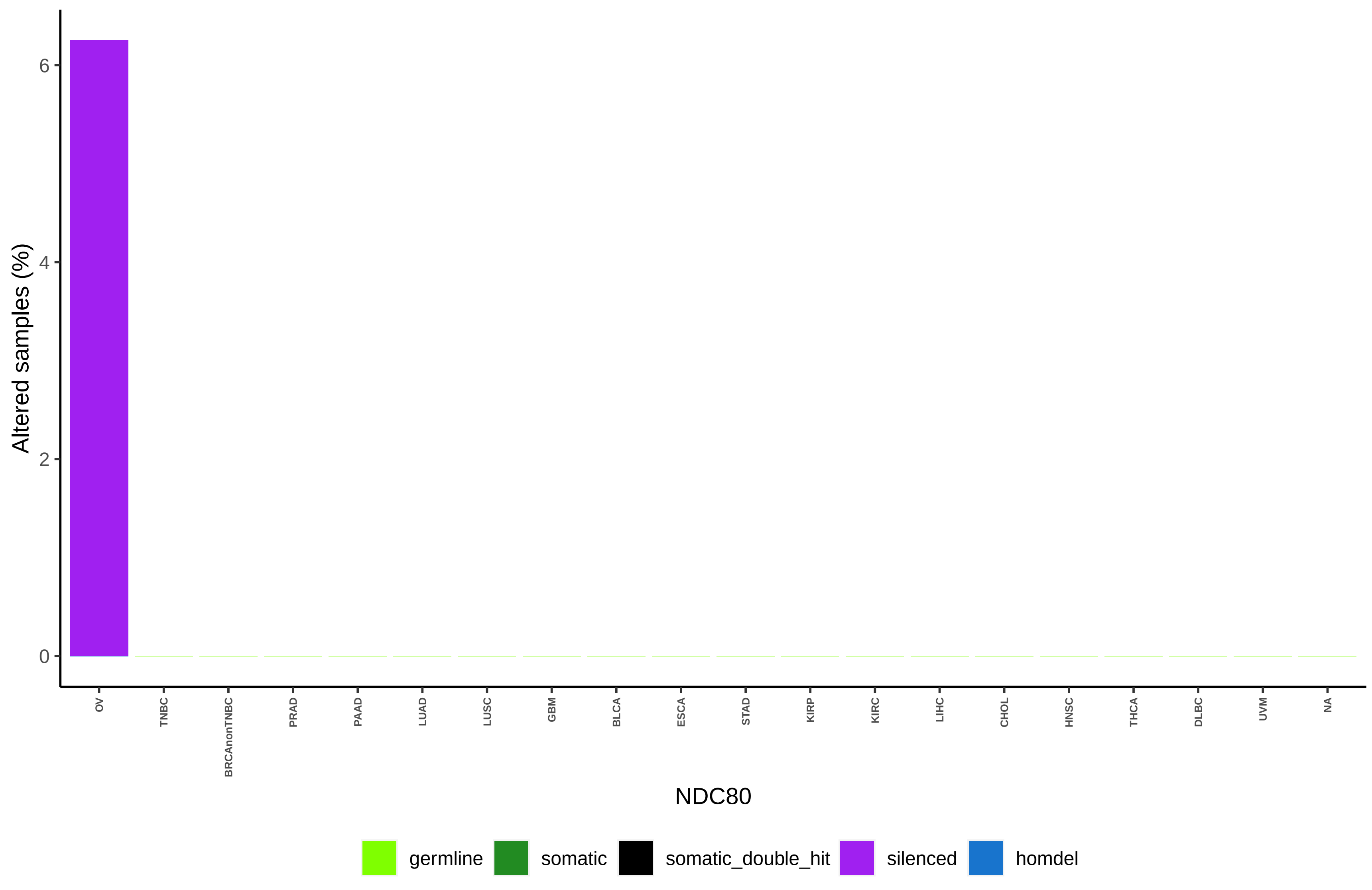
Altered samples (%)
0
2
4
6
OV TNBC BRCAnonTNBC PRAD PAAD LUAD LUSC GBM BLCA ESCA STAD KIRP KIRC LIHC CHOL HNSC THCA DLBC UVM NA
NDC80
germline somatic somatic_double_hit silenced homdel

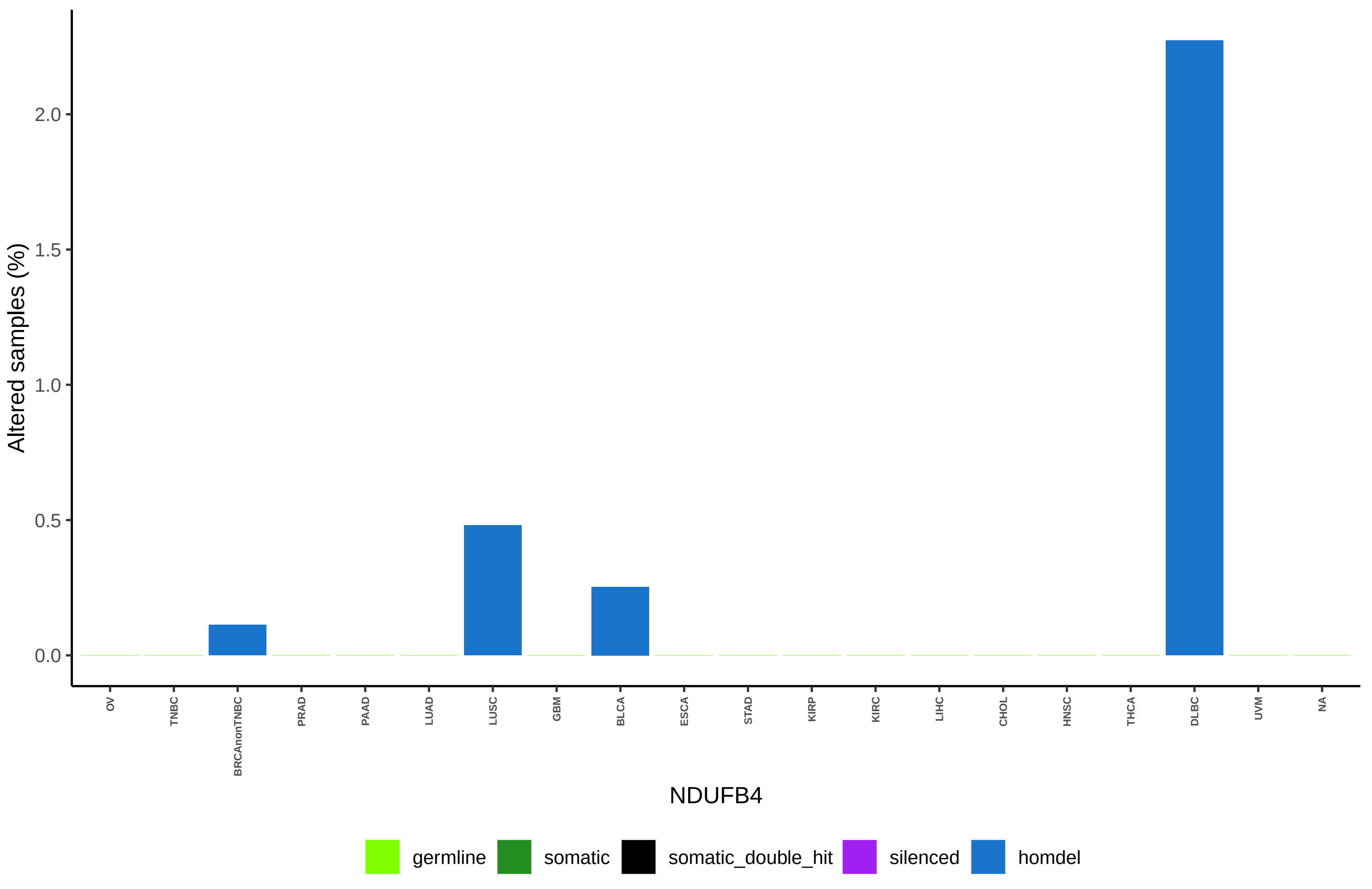
Altered samples (%)
0.0
0.5
1.0
1.5
2.0
OV TNBC BRCAnonTNBC PRAD PAAD LUAD LUSC GBM BLCA ESCA STAD KIRP KIRC LIHC CHOL HNSC THCA DLBC UVM NA
NDUFB4
germline somatic somatic_double_hit silenced homdel

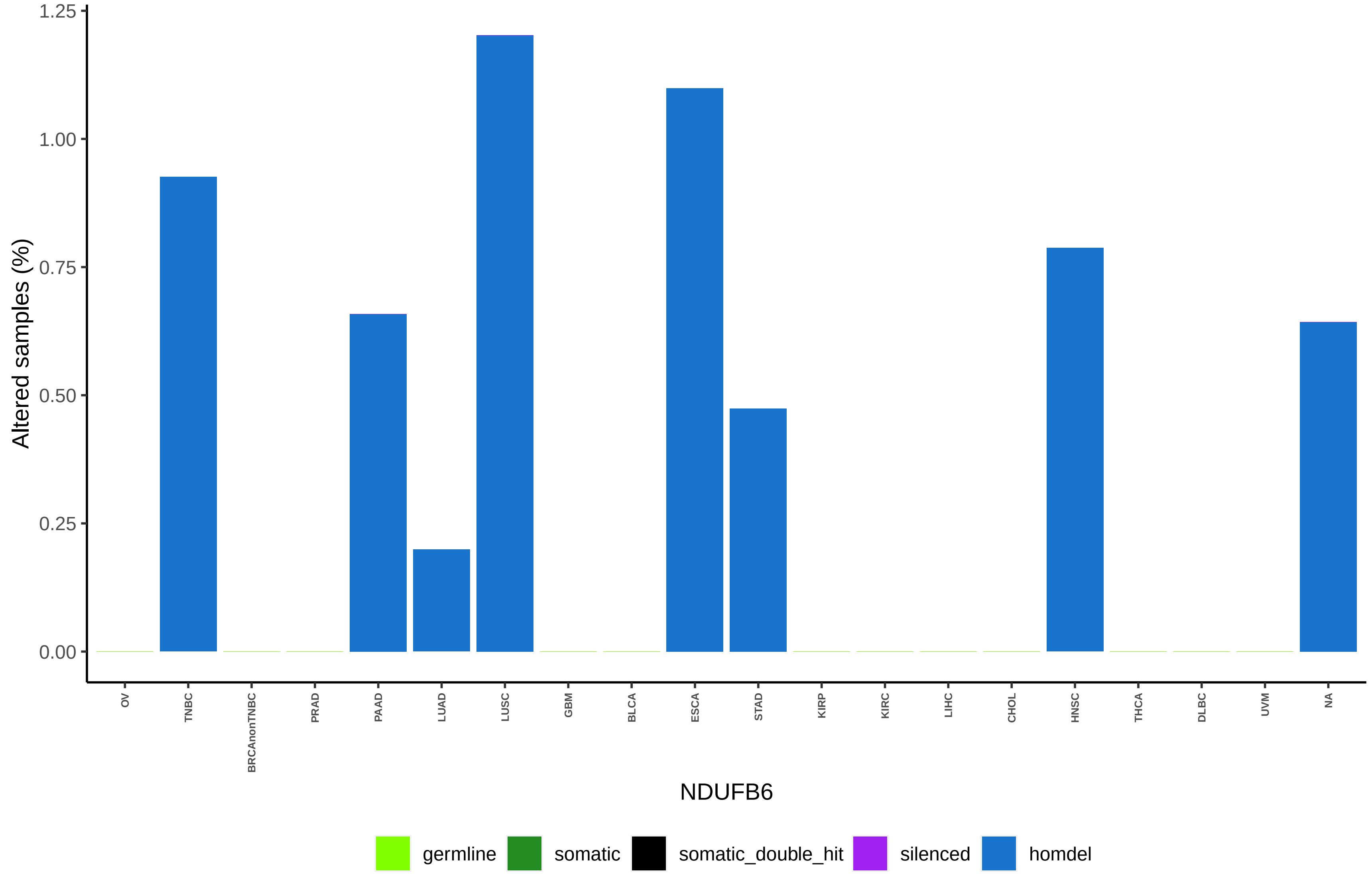
Altered samples (%)
1.25
1.00
0.75
0.50
0.25
0.00
OV
TNBC
BRCAnonTNBC
PRAD
PAAD
LUAD
LUSC
GBM
BLCA
ESCA
STAD
KIRP
KIRC
LIHC
CHOL
HNSC
THCA
DLBC
UVM
NA
NDUFB6
germline
somatic
somatic_double_hit
silenced
homdel

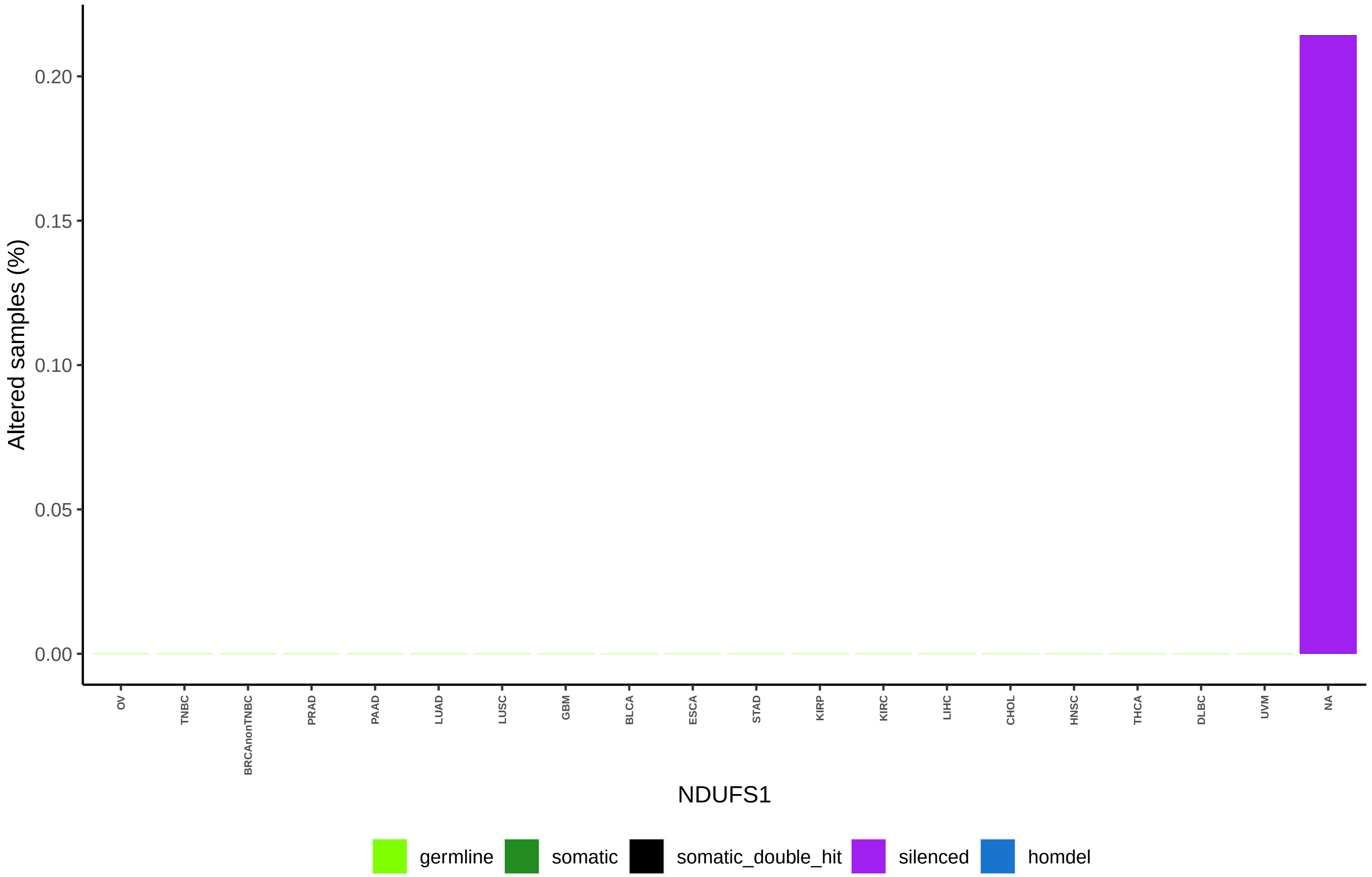
Altered samples (%)
0.20
0.15
0.10
0.05
0.00
OV
TNBC
BRCAnonTNBC
PRAD
PAAD
LUAD
LUSC
GBM
BLCA
ESCA
STAD
KIRP
KIRC
LIHC
CHOL
HNSC
THCA
DLBC
UVM
NA
NDUFS1
germline
somatic
somatic_double_hit
silenced
homdel

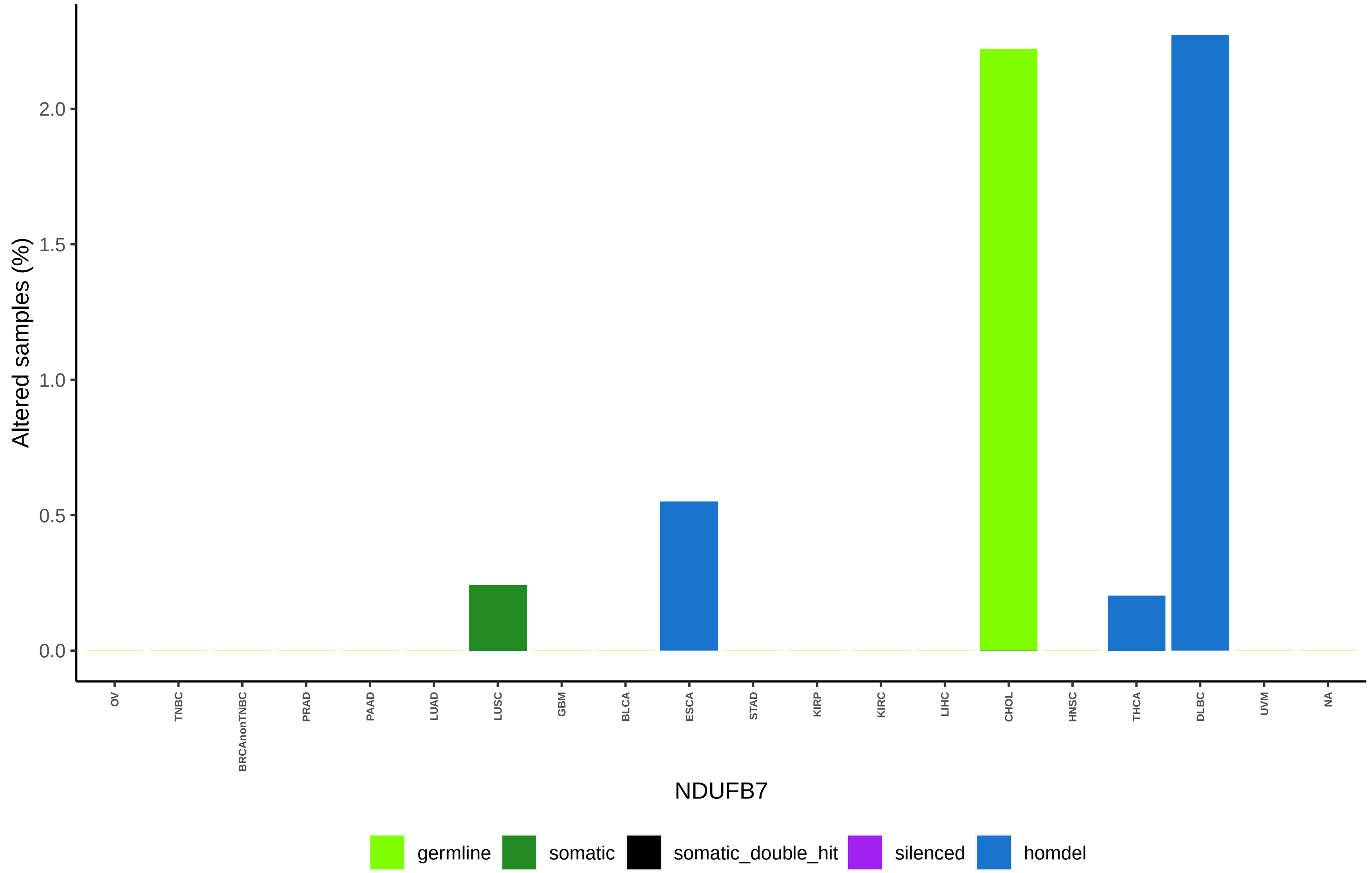
Altered samples (%)
2.0
1.5
1.0
0.5
0.0
OV
TNBC
BRCAnonTNBC
PRAD
PAAD
LUAD
LUSC
GBM
BLCA
ESCA
STAD
KIRP
KIRC
LIHC
CHOL
HNSC
THCA
DLBC
UVM
NA
NDUFB7
germline
somatic
somatic_double_hit
silenced
homdel

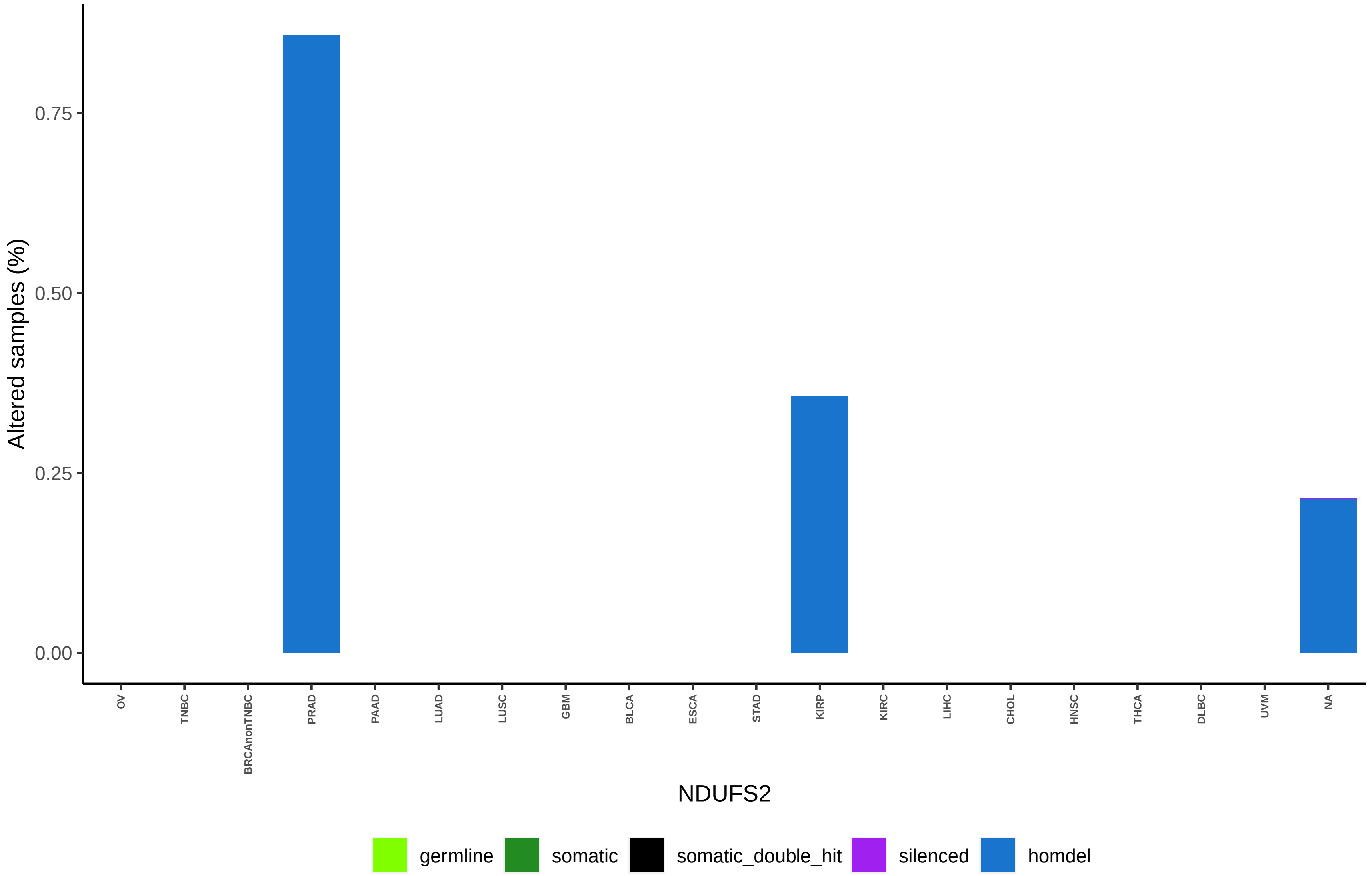
Altered samples (%)
0.75
0.50
0.25
0.00
OV
TNBC
BRCAnonTNBC
PRAD
PAAD
LUAD
LUSC
GBM
BLCA
ESCA
STAD
KIRP
KIRC
LIHC
CHOL
HNSC
THCA
DLBC
UVM
NA
NDUFS2
germline
somatic
somatic_double_hit
silenced
homdel

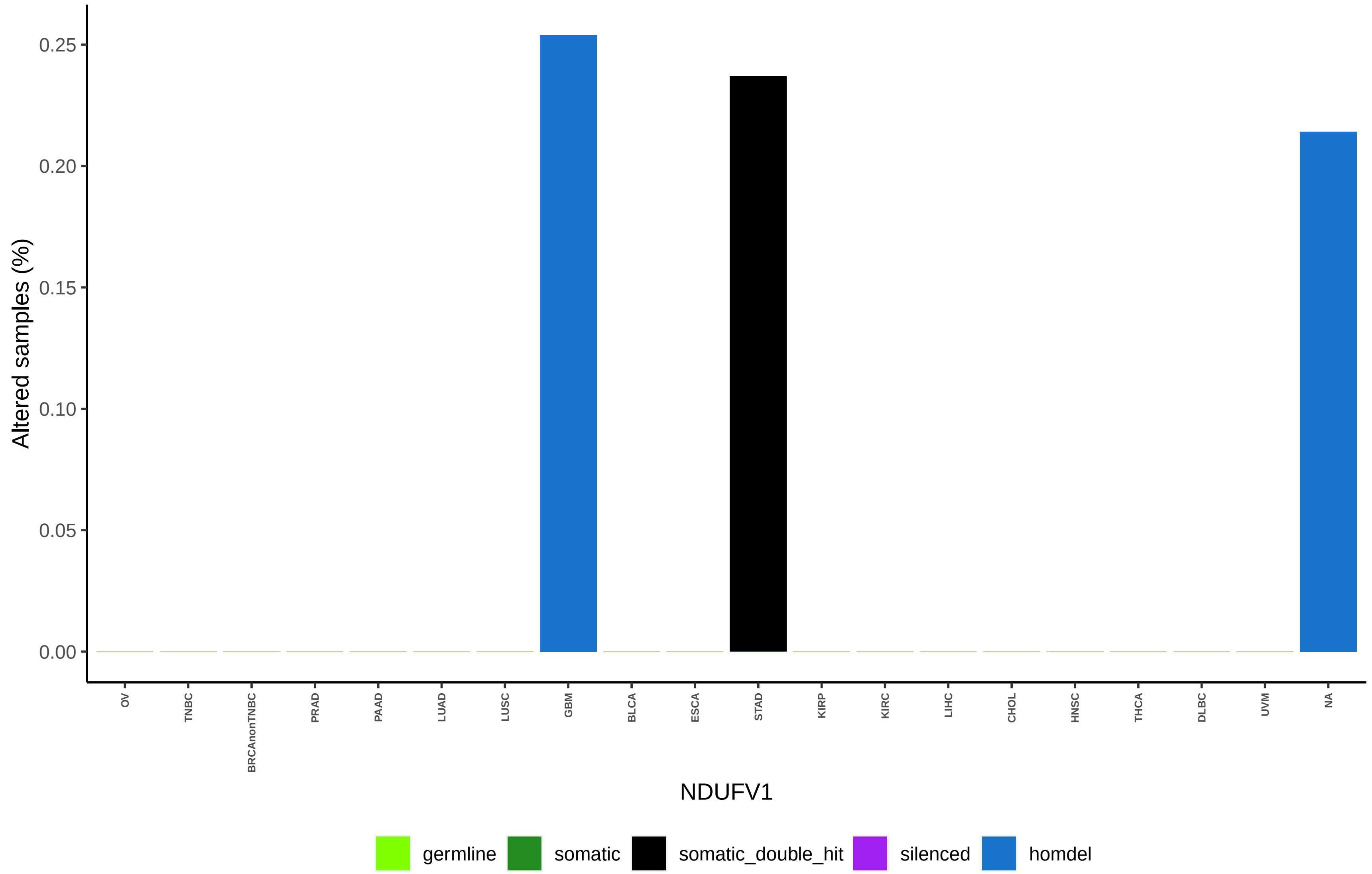
Altered samples (%)
0.00
0.05
0.10
0.15
0.20
0.25
OV
TNBC
BRCAnonTNBC
PRAD
PAAD
LUAD
LUSC
GBM
BLCA
ESCA
STAD
KIRP
KIRC
LIHC
CHOL
HNSC
THCA
DLBC
UVM
NA
NDUFV1
germline
somatic
somatic_double_hit
silenced
homdel

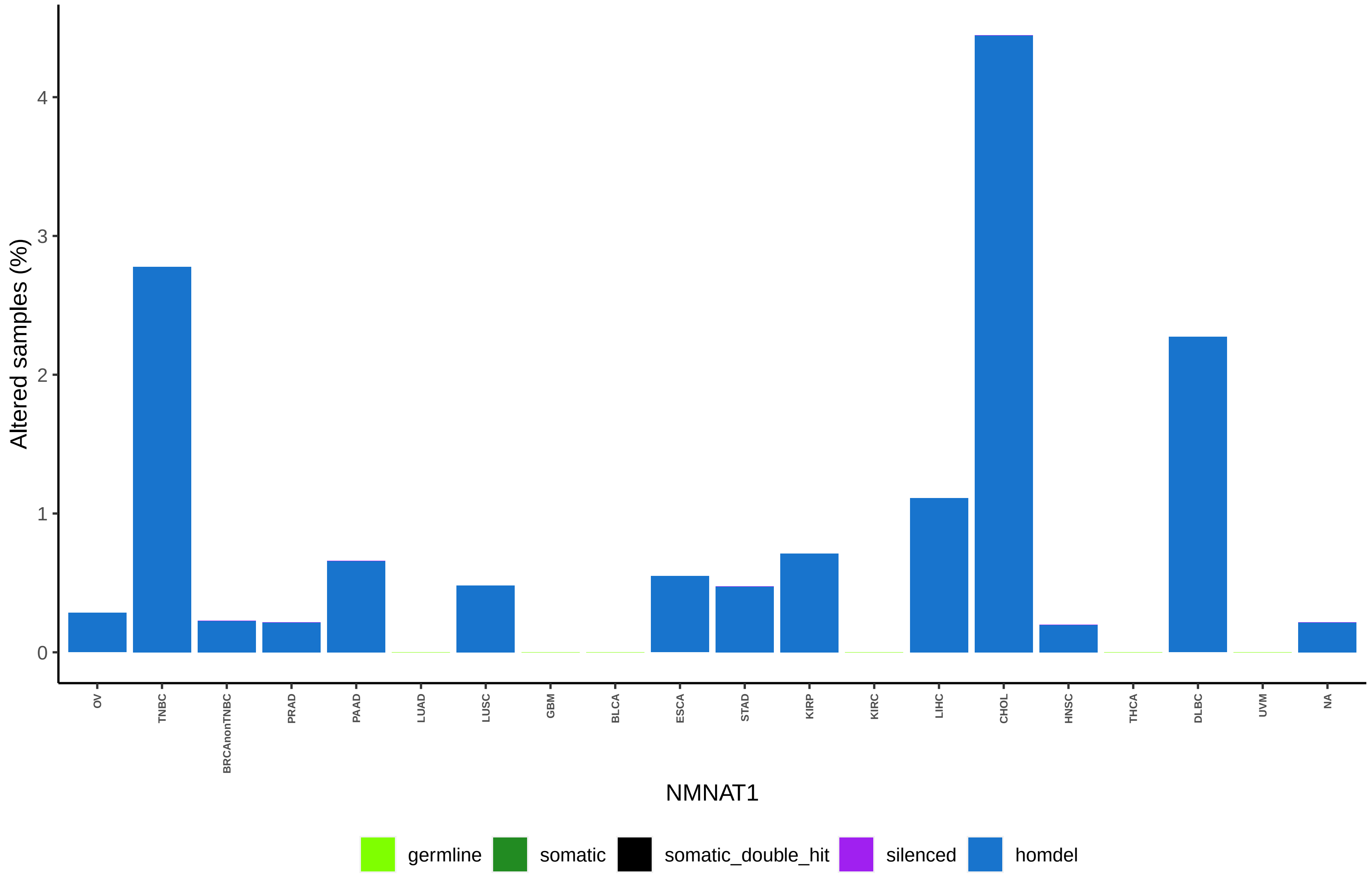
Altered samples (%)
0
1
2
3
4
OV
TNBC
BRCAnonTNBC
PRAD
PAAD
LUAD
LUSC
GBM
BLCA
ESCA
STAD
KIRP
KIRC
LIHC
CHOL
HNSC
THCA
DLBC
UVM
NA
NMNAT1
germline
somatic
somatic_double_hit
silenced
homdel

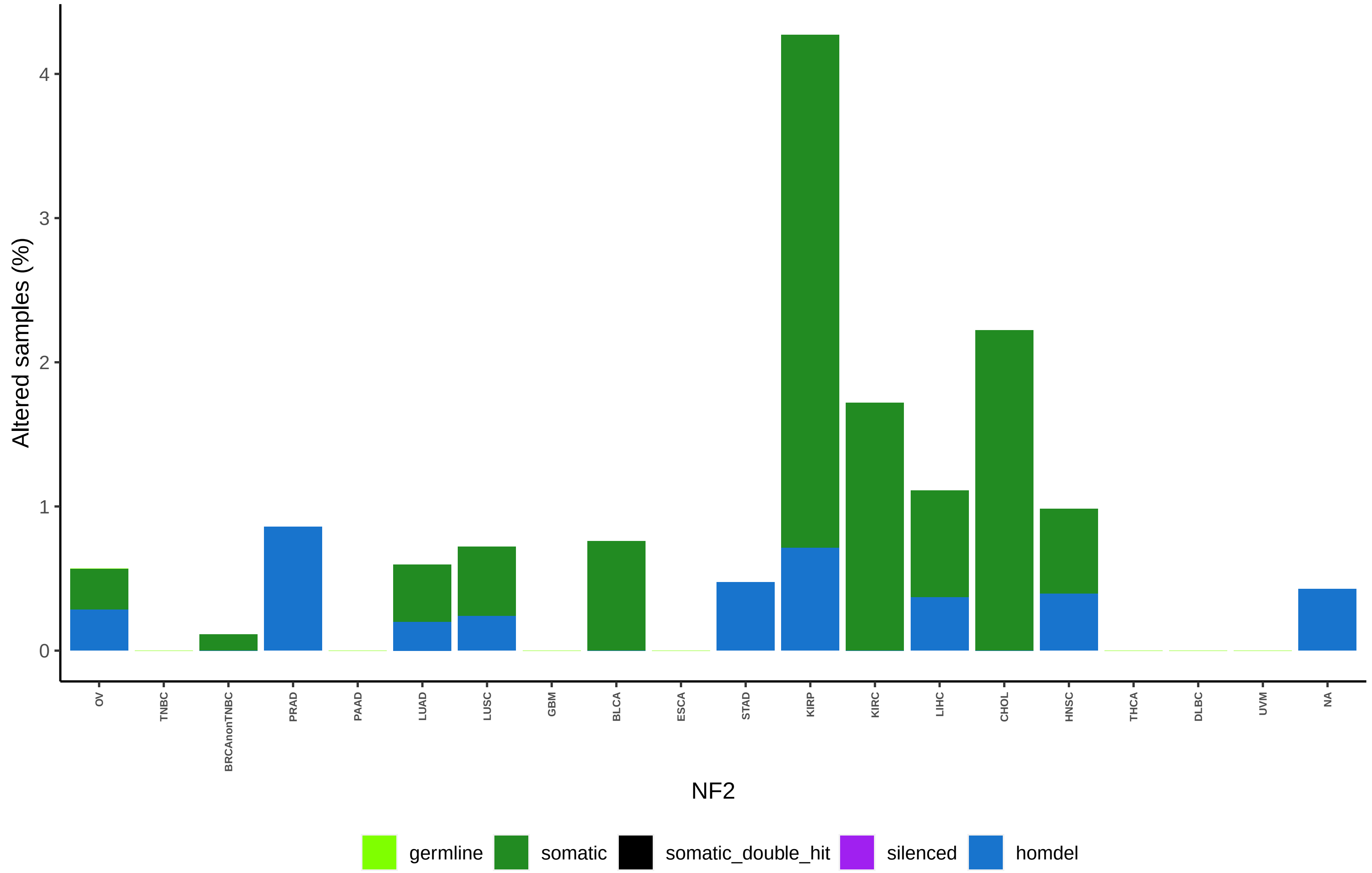
Altered samples (%)
0
1
2
3
4
OV
TNBC
BRCAnonTNBC
PRAD
PAAD
LUAD
LUSC
GBM
BLCA
ESCA
STAD
KIRP
KIRC
LIHC
CHOL
HNSC
THCA
DLBC
UVM
NA
NF2
germline
somatic
somatic_double_hit
silenced
homdel

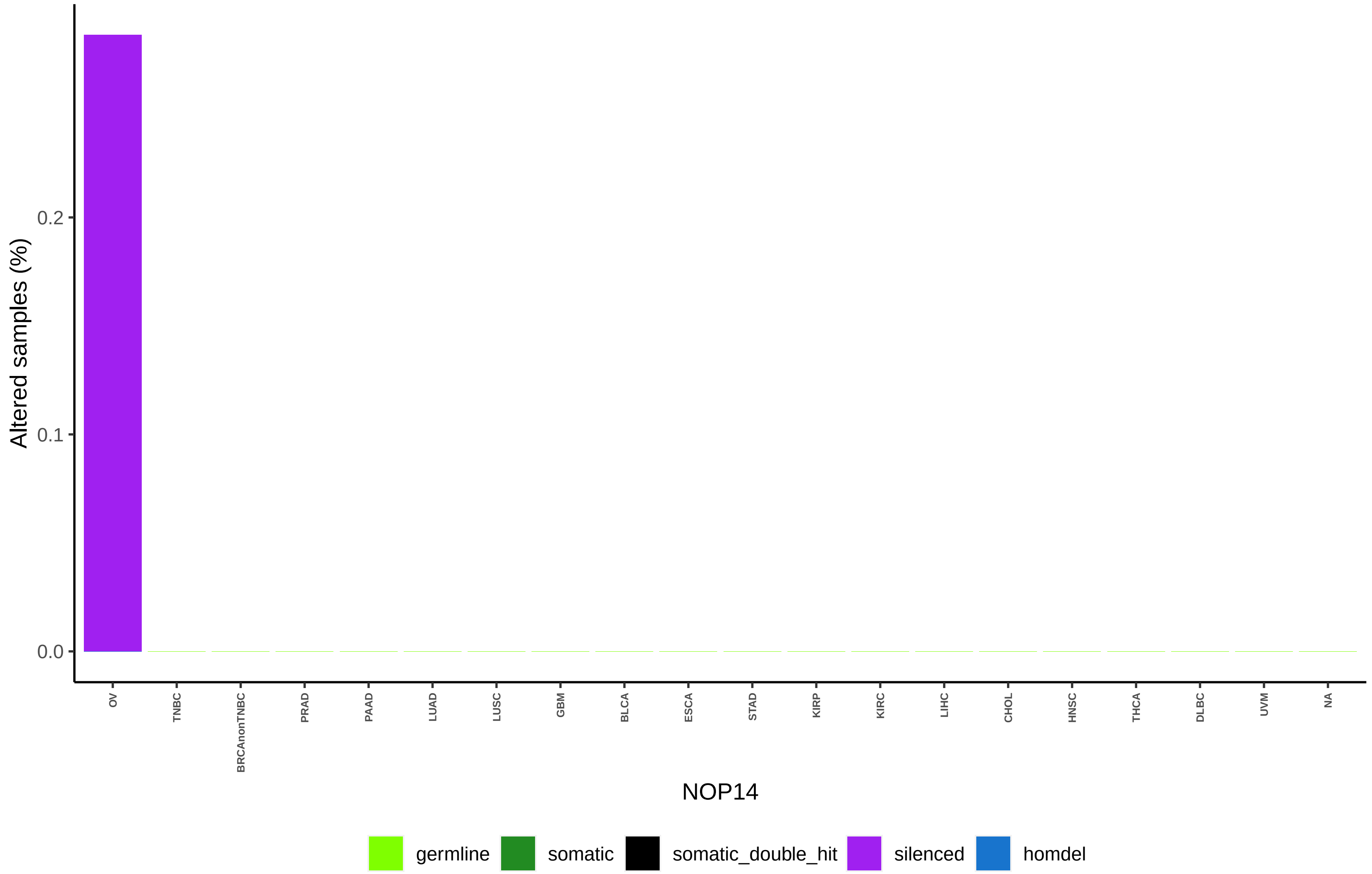
Altered samples (%)
0.0
0.1
0.2
OV
TNBC
BRCAnonTNBC
PRAD
PAAD
LUAD
LUSC
GBM
BLCA
ESCA
STAD
KIRP
KIRC
LIHC
CHOL
HNSC
THCA
DLBC
UVM
NA
NOP14
germline
somatic
somatic_double_hit
silenced
homdel

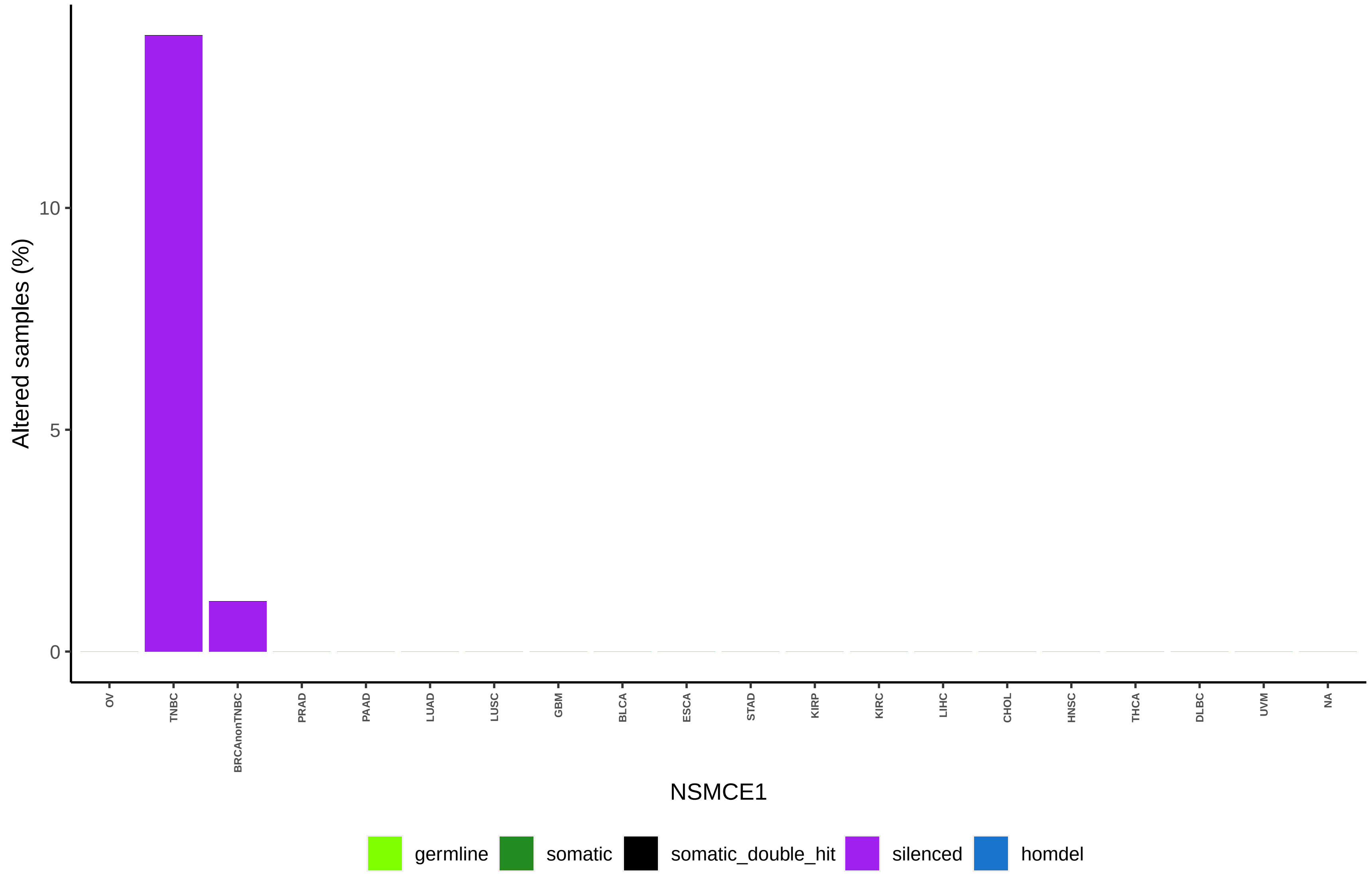
Altered samples (%)
0
5
10
OV
TNBC
BRCAnonTNBC
PRAD
PAAD
LUAD
LUSC
GBM
BLCA
ESCA
STAD
KIRP
KIRC
LIHC
CHOL
HNSC
THCA
DLBC
UVM
NA
NSMCE1
germline
somatic
somatic_double_hit
silenced
homdel

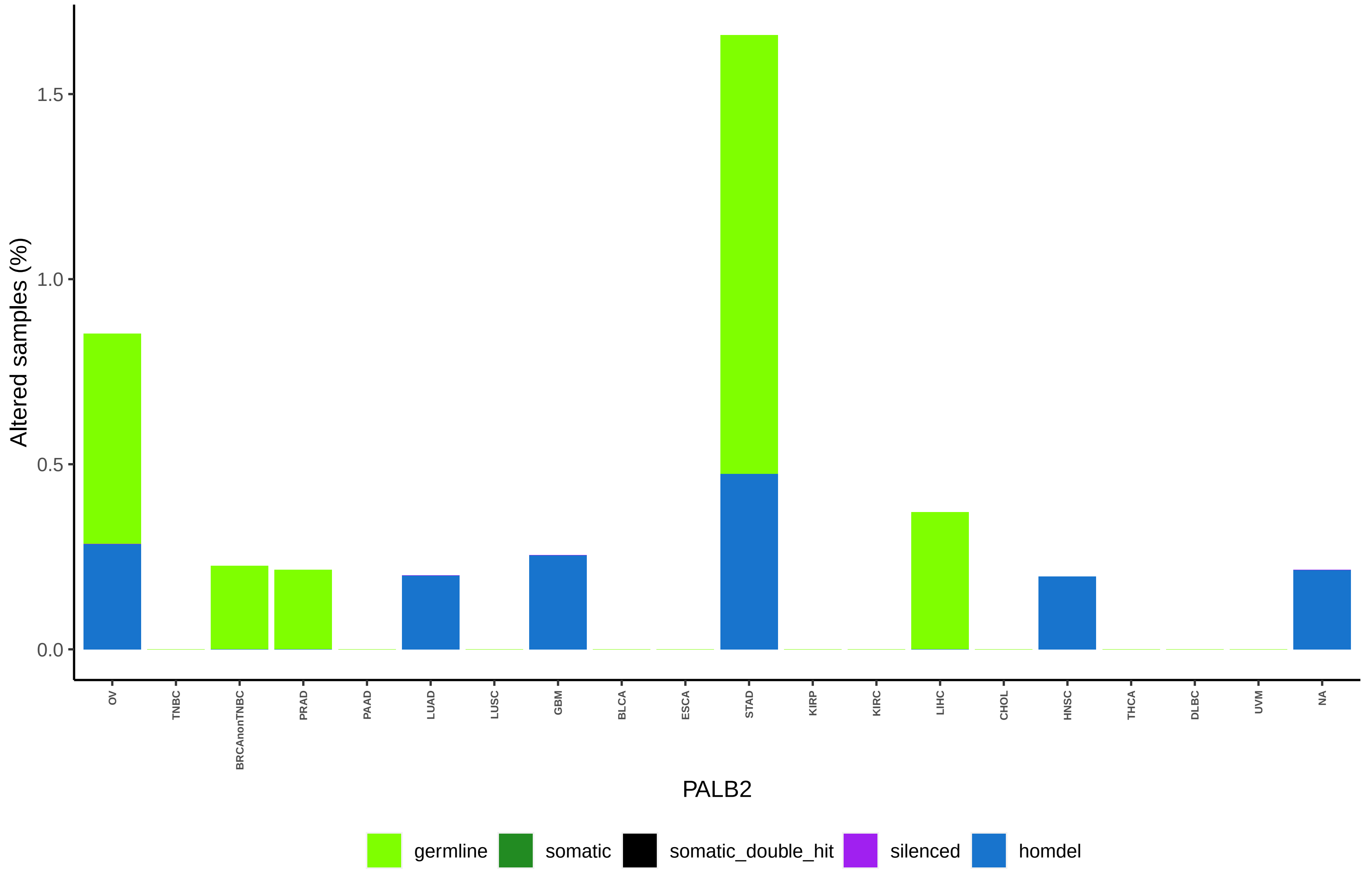
Altered samples (%)
0.0
0.5
1.0
1.5
OV
TNBC
BRCAnonTNBC
PRAD
PAAD
LUAD
LUSC
GBM
BLCA
ESCA
STAD
KIRP
KIRC
LIHC
CHOL
HNSC
THCA
DLBC
UVM
NA
PALB2
germline
somatic
somatic_double_hit
silenced
homdel

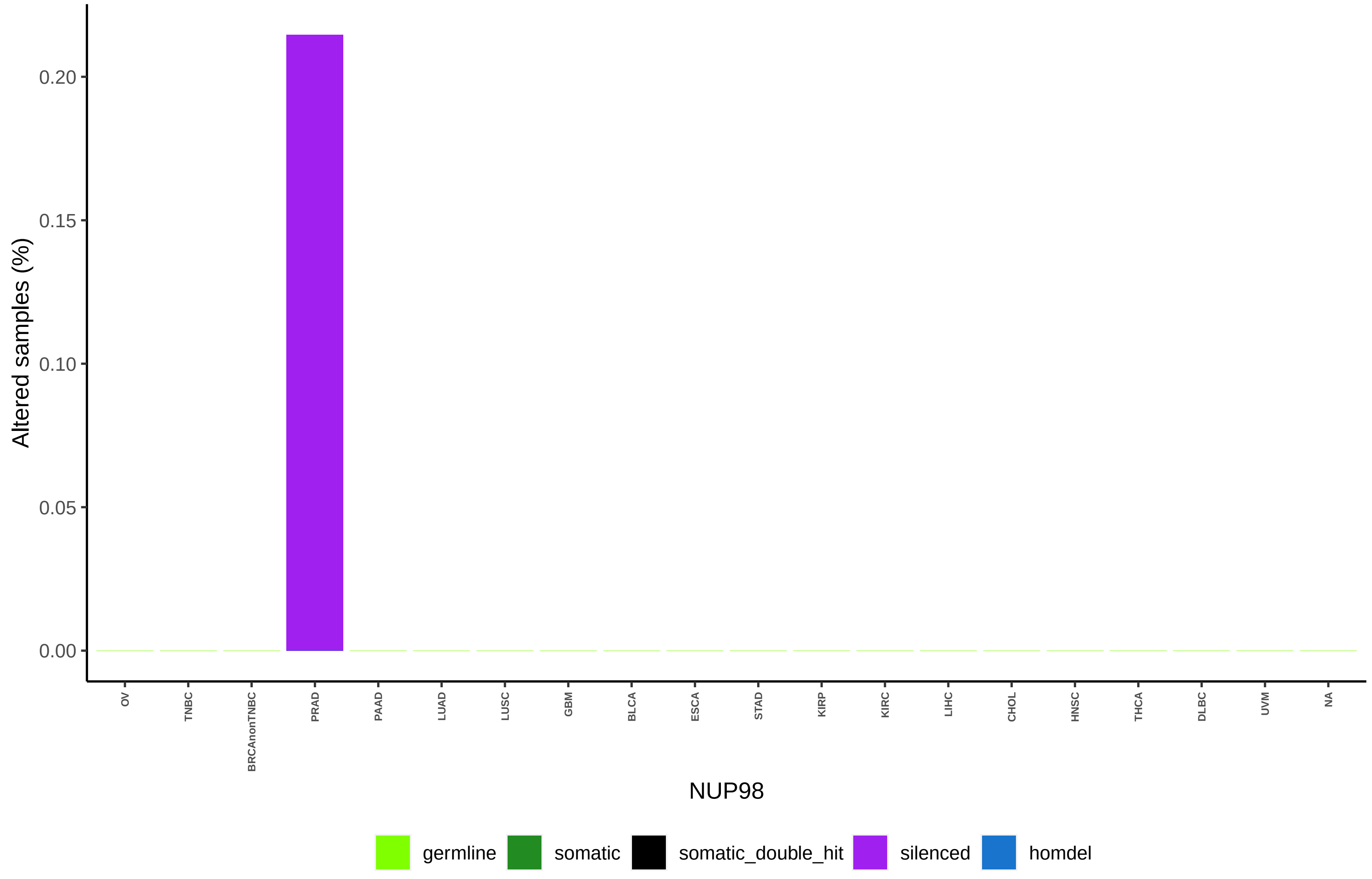
Altered samples (%)
0.00
0.05
0.10
0.15
0.20
OV
TNBC
BRCAnonTNBC
PRAD
PAAD
LUAD
LUSC
GBM
BLCA
ESCA
STAD
KIRP
KIRC
LIHC
CHOL
HNSC
THCA
DLBC
UVM
NA
NUP98
germline
somatic
somatic_double_hit
silenced
homdel

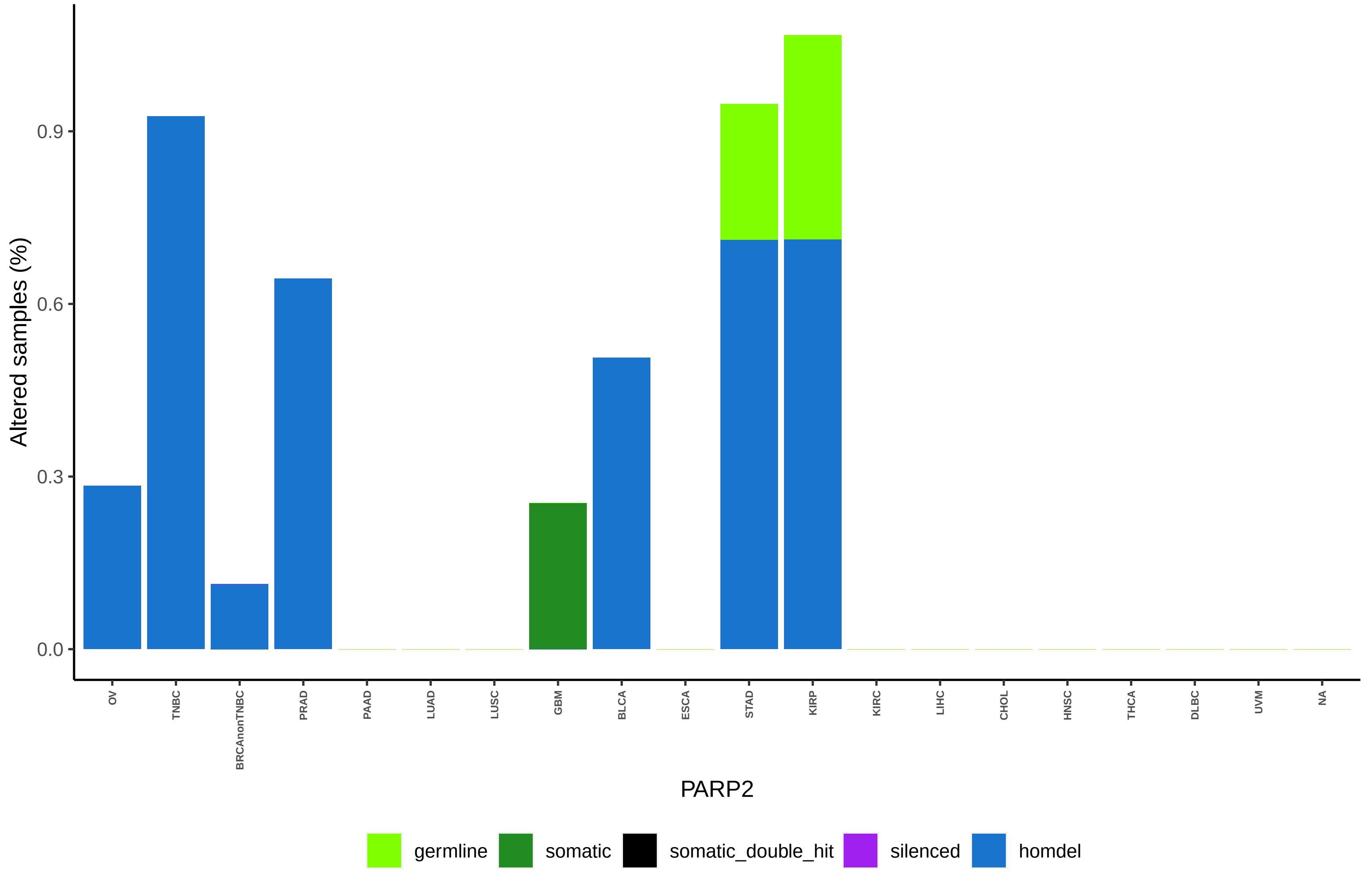
Altered samples (%)
0.0
0.3
0.6
0.9
OV
TNBC
BRCAnonTNBC
PRAD
PAAD
LUAD
LUSC
GBM
BLCA
ESCA
STAD
KIRP
KIRC
LIHC
CHOL
HNSC
THCA
DLBC
UVM
NA
PARP2
germline
somatic
somatic_double_hit
silenced
homdel

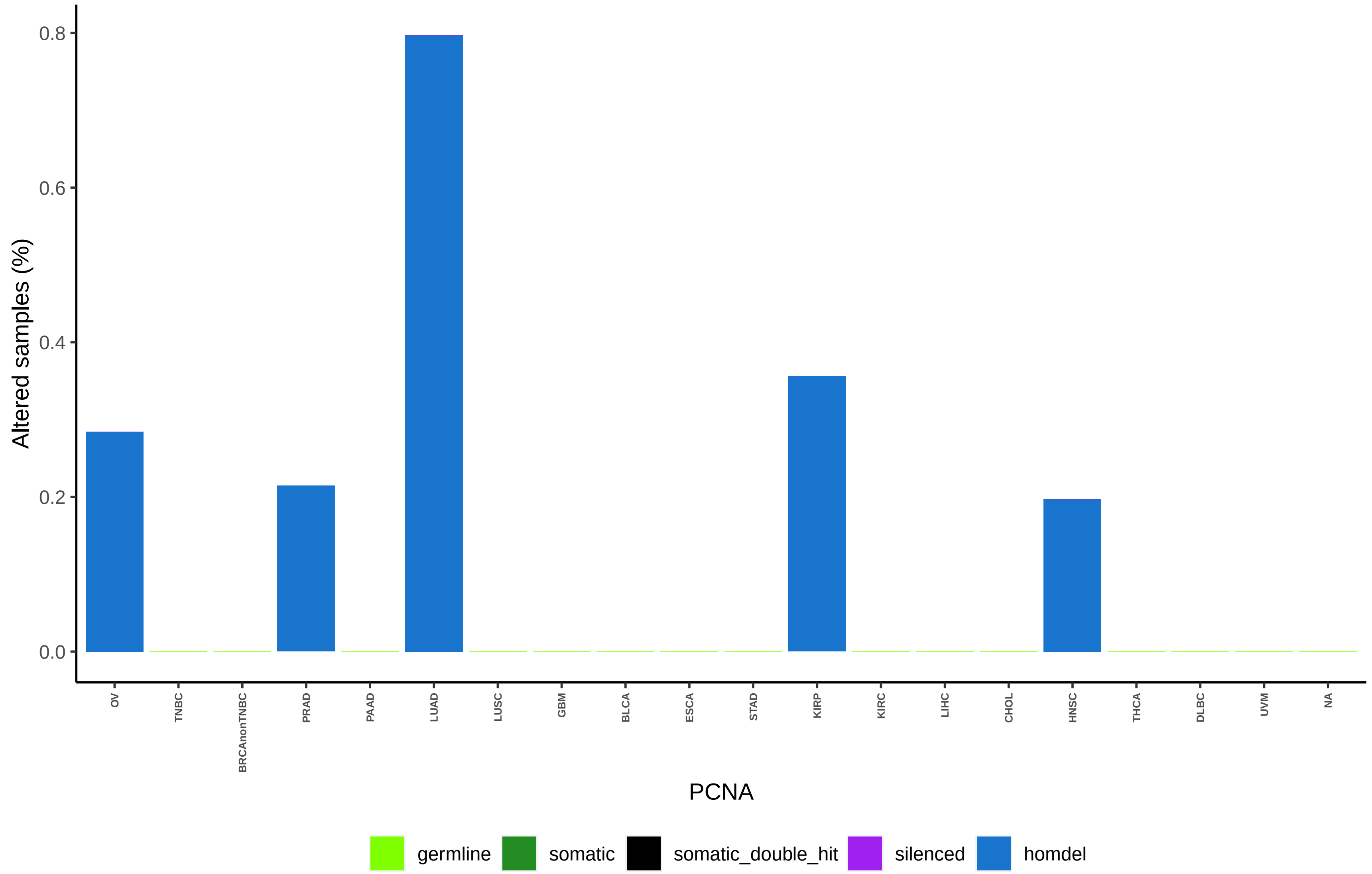
Altered samples (%)
0.0
0.2
0.4
0.6
0.8
OV TNBC BRCAnonTNBC PRAD PAAD LUAD LUSC GBM BLCA ESCA STAD KIRP KIRC LIHC CHOL HNSC THCA DLBC UVM NA
PCNA
germline
somatic
somatic_double_hit
silenced
homdel

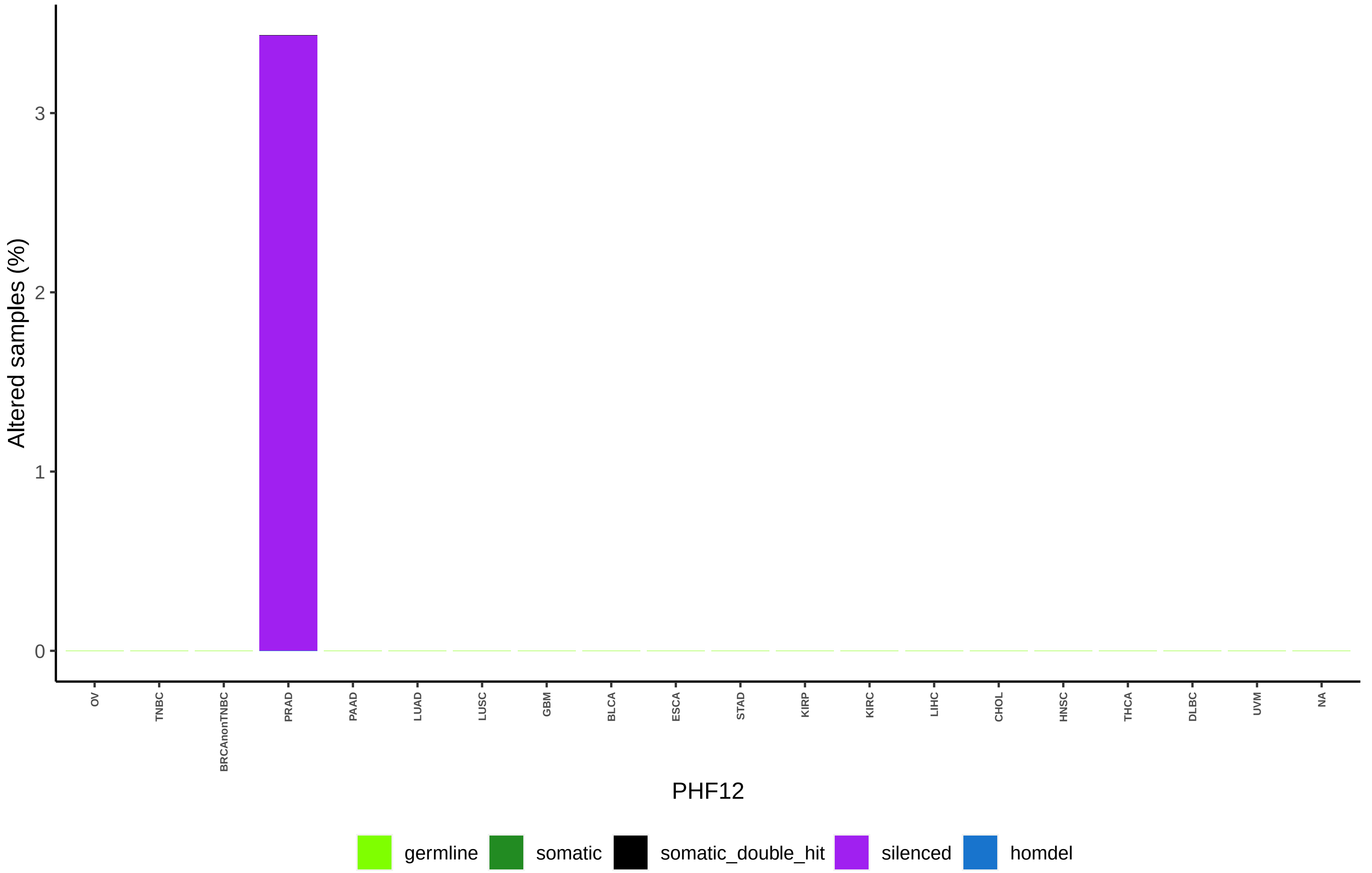
Altered samples (%)
0
1
2
3
OV TNBC BRCAnonTNBC PRAD PAAD LUAD LUSC GBM BLCA ESCA STAD KIRP KIRC LIHC CHOL HNSC THCA DLBC UVM NA
PHF12
germline
somatic
somatic_double_hit
silenced
homdel

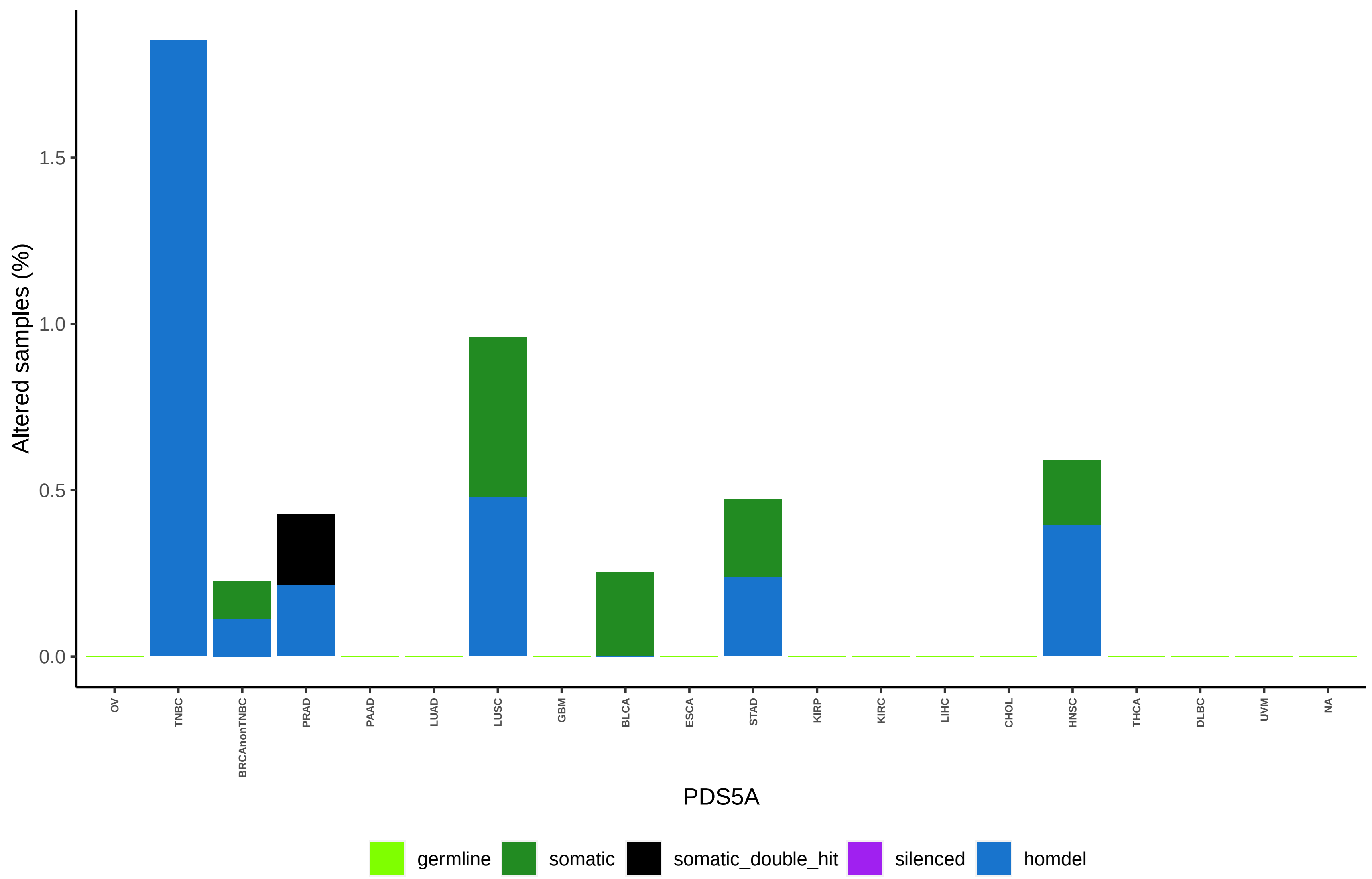
Altered samples (%)
0.0
0.5
1.0
1.5
OV TNBC BRCAnonTNBC PRAD PAAD LUAD LUSC GBM BLCA ESCA STAD KIRP KIRC LIHC CHOL HNSC THCA DLBC UVM NA
PDS5A
germline
somatic
somatic_double_hit
silenced
homdel

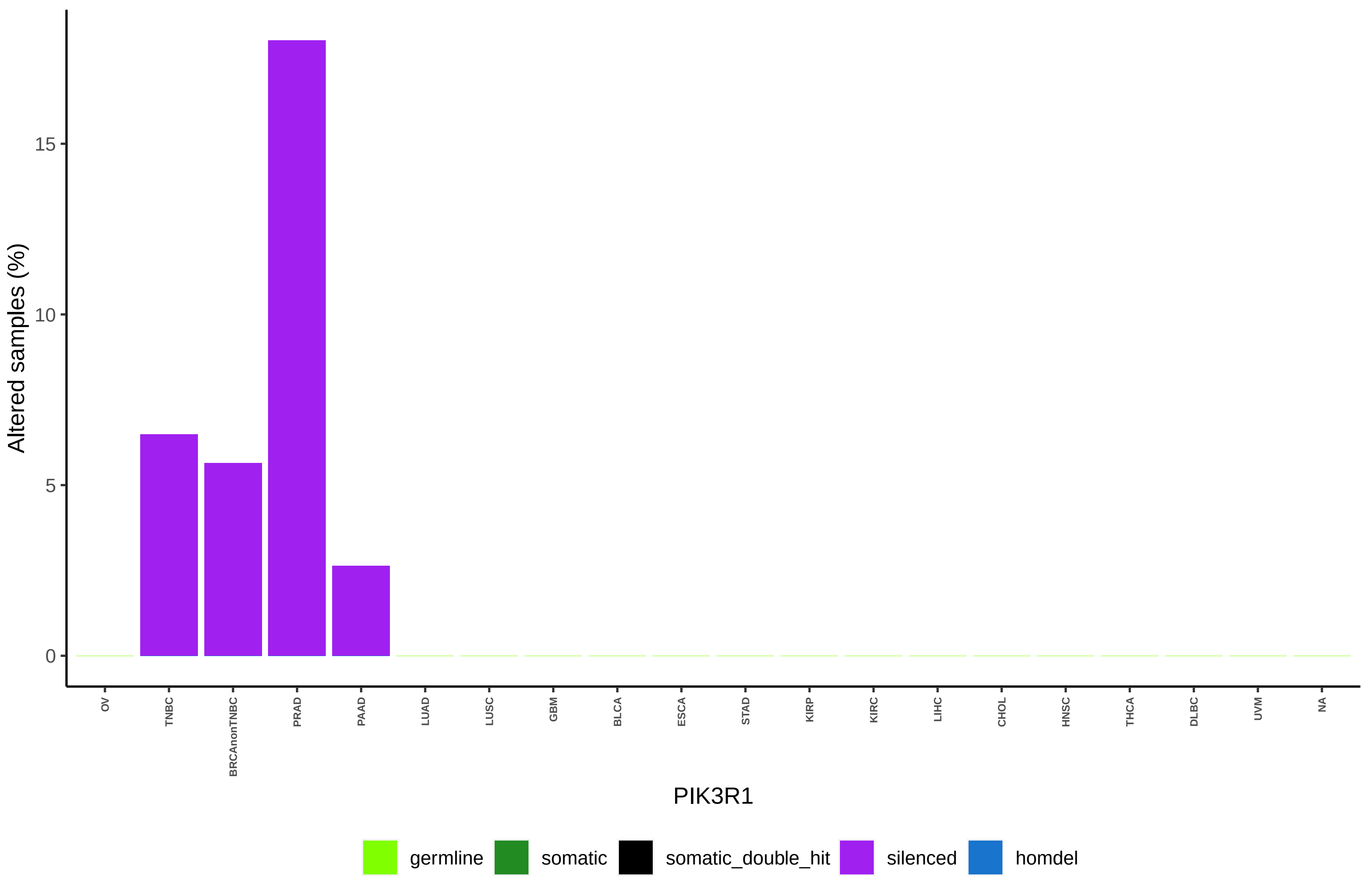
Altered samples (%)
0
5
10
15
OV TNBC BRCAnonTNBC PRAD PAAD LUAD LUSC GBM BLCA ESCA STAD KIRP KIRC LIHC CHOL HNSC THCA DLBC UVM NA
PIK3R1
germline
somatic
somatic_double_hit
silenced
homdel

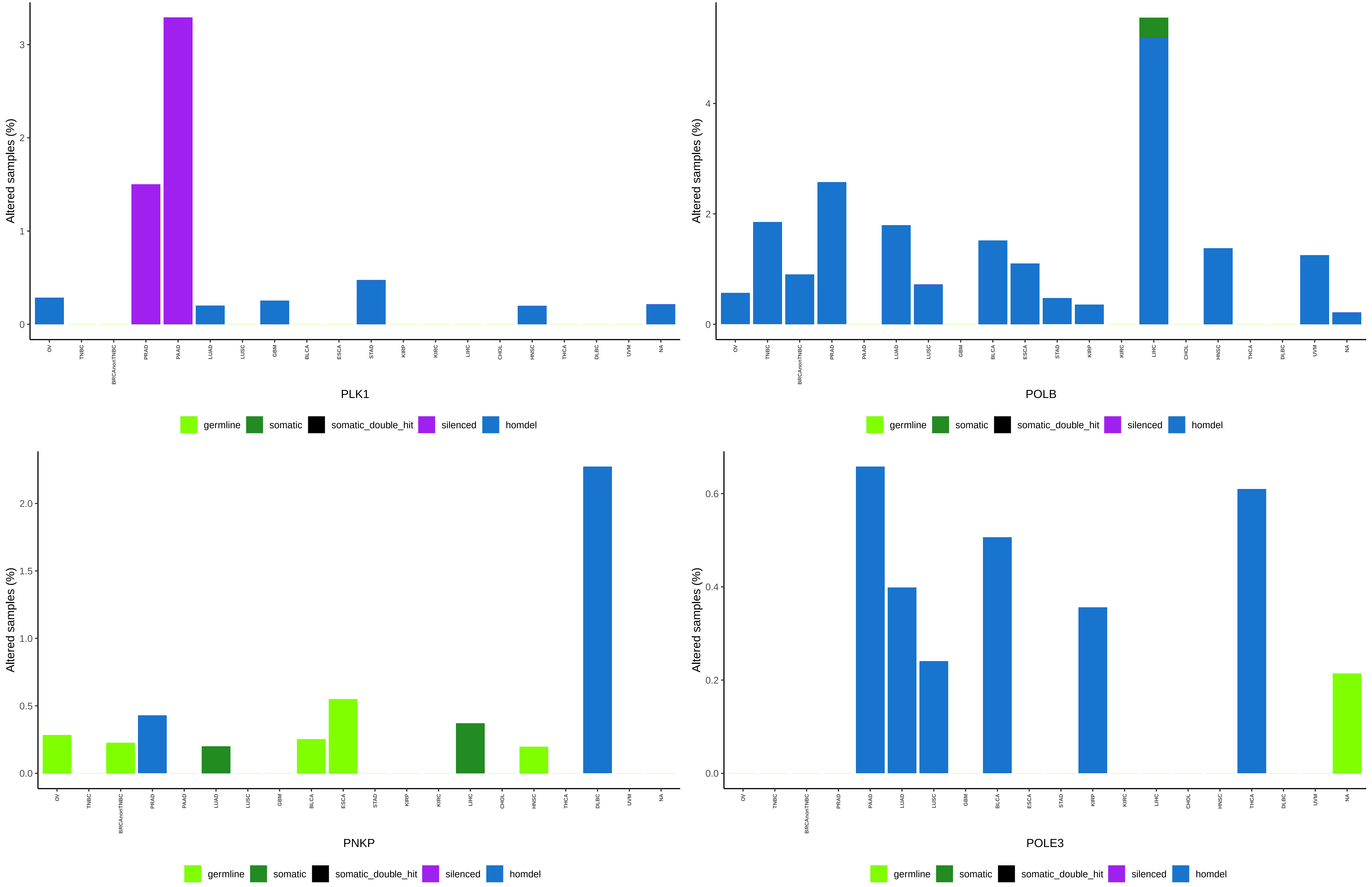

Altered samples (%)
OV
TNBC
BRCAnonTNBC
PRAD
PAAD
LUAD
LUSC
GBM
BLCA
ESCA
STAD
KIRP
KIRC
LIHC
CHOL
HNSC
THCA
DLBC
UVM
NA
PLK1
POLB
PNKP
POLE3
germline
somatic
somatic_double_hit
silenced
homdel

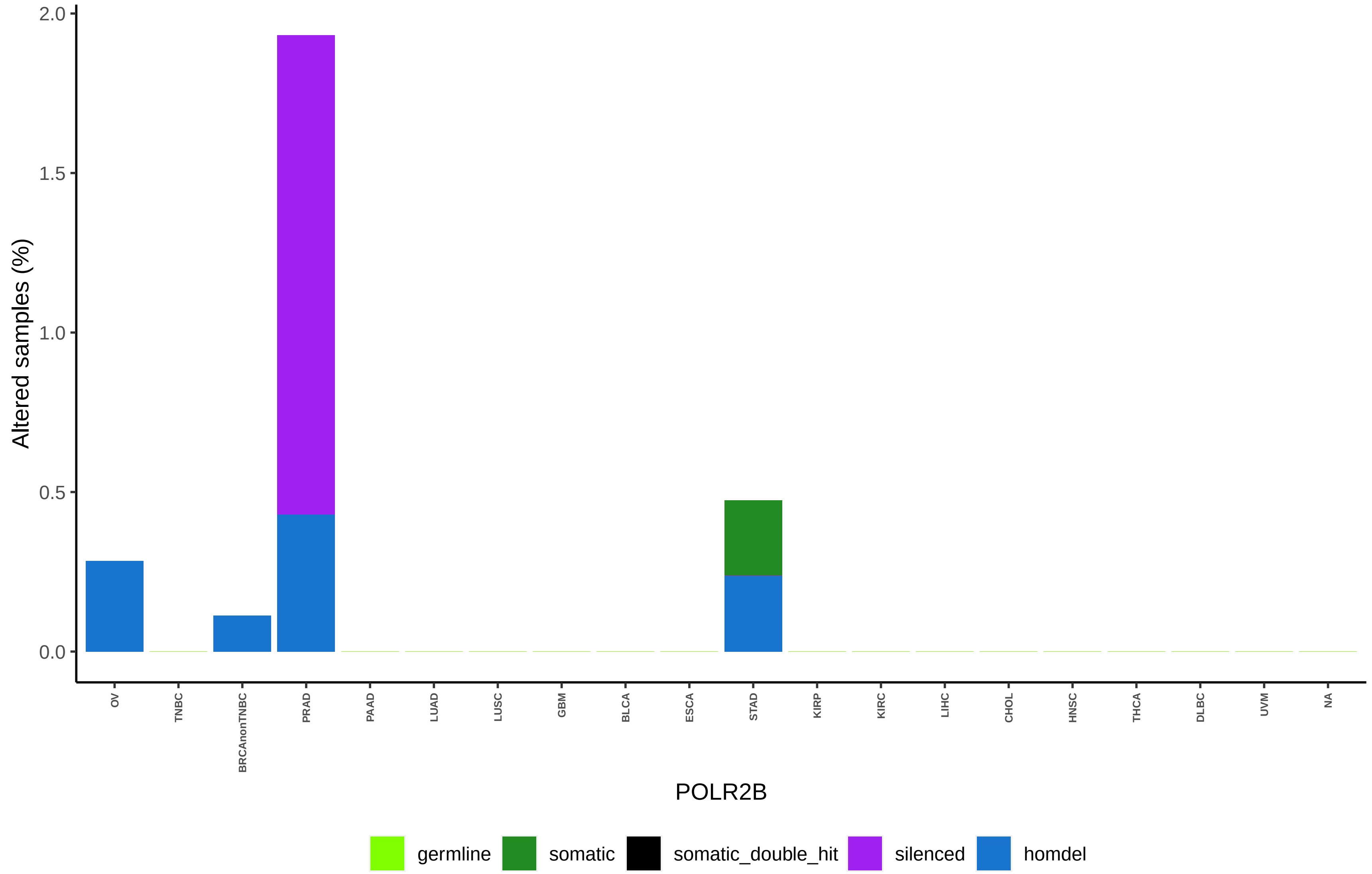
Altered samples (%)
2.0
1.5
1.0
0.5
0.0
OV
TNBC
BRCAnonTNBC
PRAD
PAAD
LUAD
LUSC
GBM
BLCA
ESCA
STAD
KIRP
KIRC
LIHC
CHOL
HNSC
THCA
DLBC
UVM
NA
POLR2B
germline
somatic
somatic_double_hit
silenced
homdel

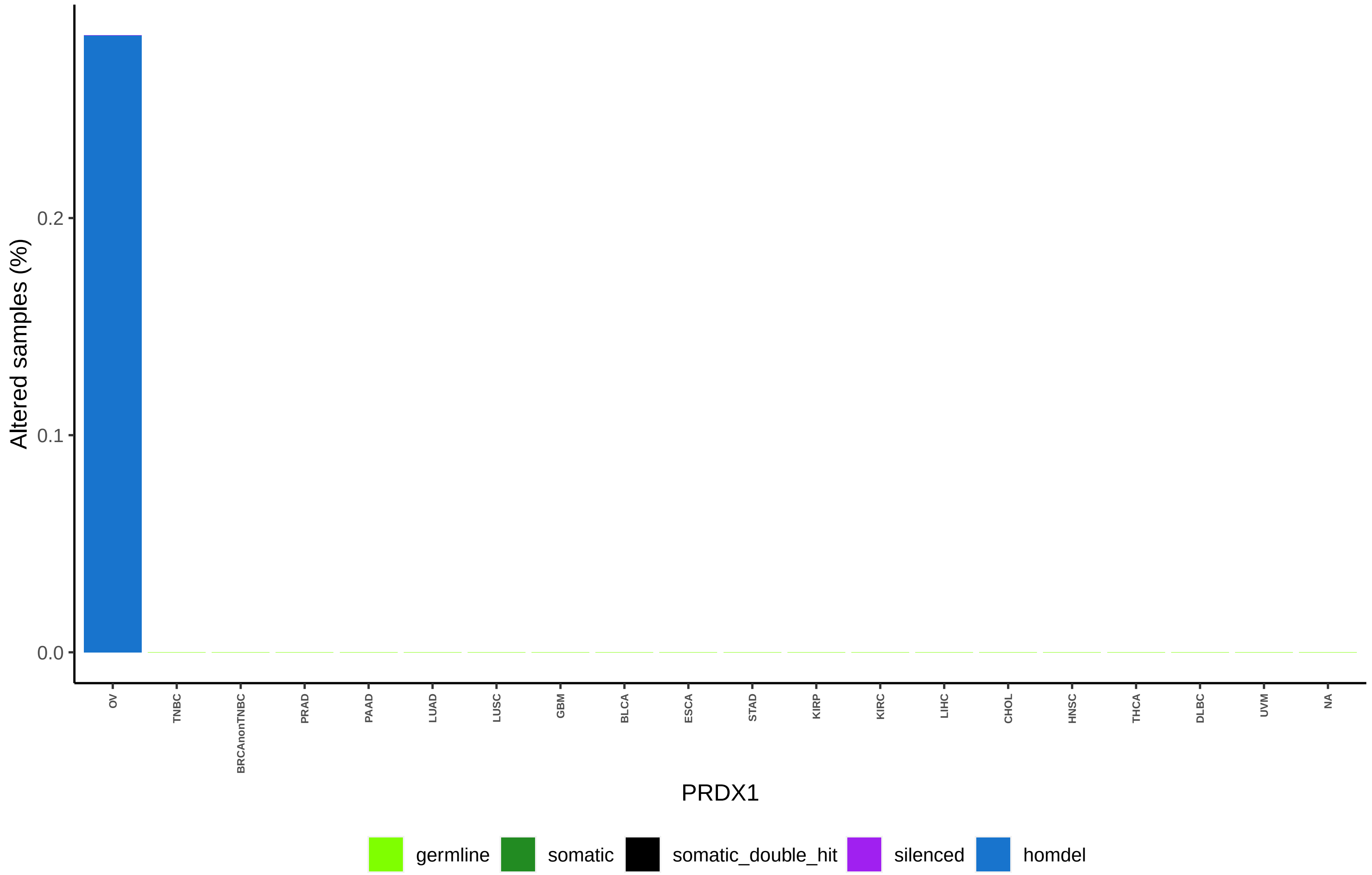
Altered samples (%)
0.2
0.1
0.0
OV
TNBC
BRCAnonTNBC
PRAD
PAAD
LUAD
LUSC
GBM
BLCA
ESCA
STAD
KIRP
KIRC
LIHC
CHOL
HNSC
THCA
DLBC
UVM
NA
PRDX1
germline
somatic
somatic_double_hit
silenced
homdel

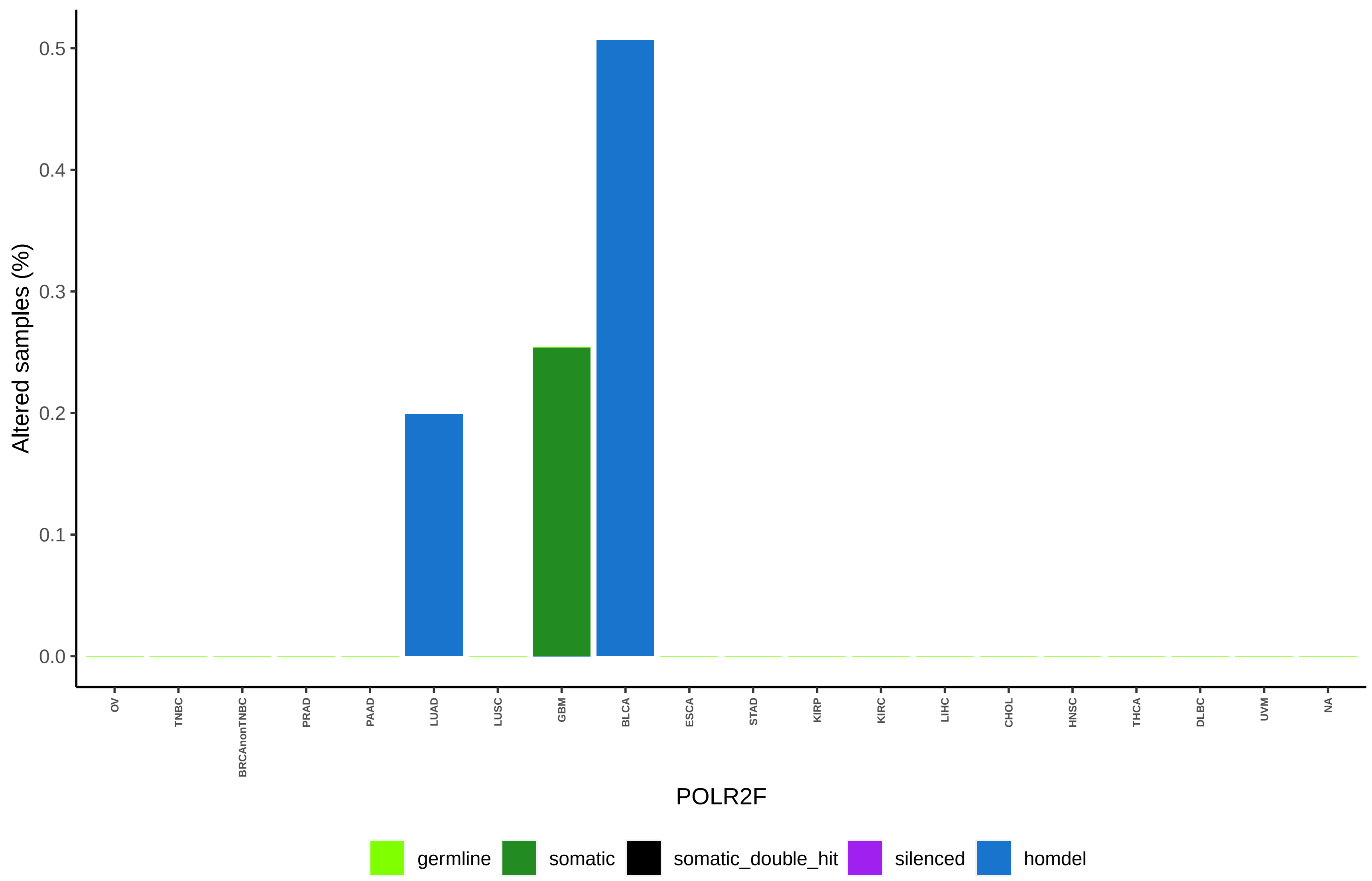
Altered samples (%)
0.5
0.4
0.3
0.2
0.1
0.0
OV
TNBC
BRCAnonTNBC
PRAD
PAAD
LUAD
LUSC
GBM
BLCA
ESCA
STAD
KIRP
KIRC
LIHC
CHOL
HNSC
THCA
DLBC
UVM
NA
POLR2F
germline
somatic
somatic_double_hit
silenced
homdel

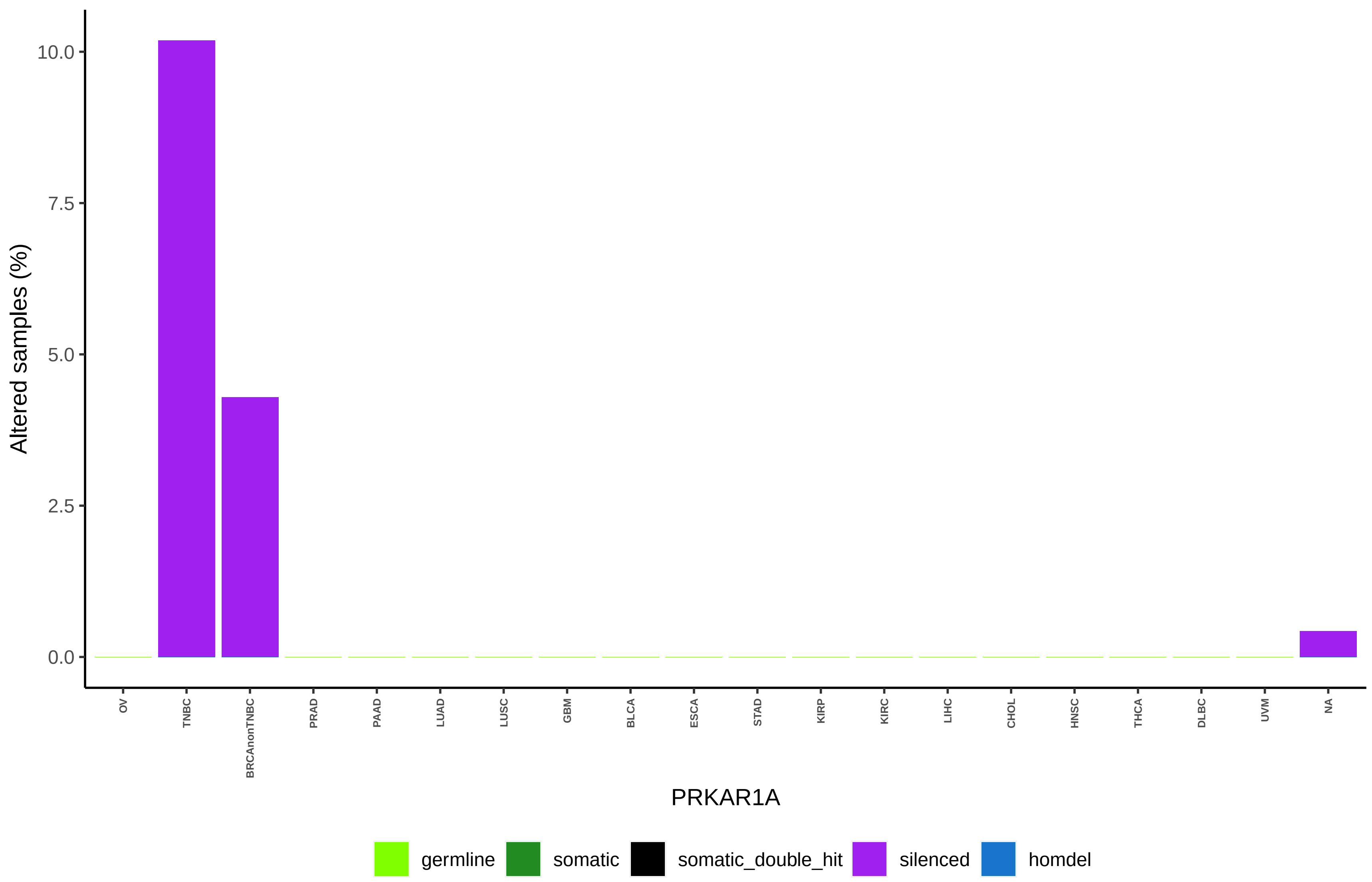
Altered samples (%)
10.0
7.5
5.0
2.5
0.0
OV
TNBC
BRCAnonTNBC
PRAD
PAAD
LUAD
LUSC
GBM
BLCA
ESCA
STAD
KIRP
KIRC
LIHC
CHOL
HNSC
THCA
DLBC
UVM
NA
PRKAR1A
germline
somatic
somatic_double_hit
silenced
homdel

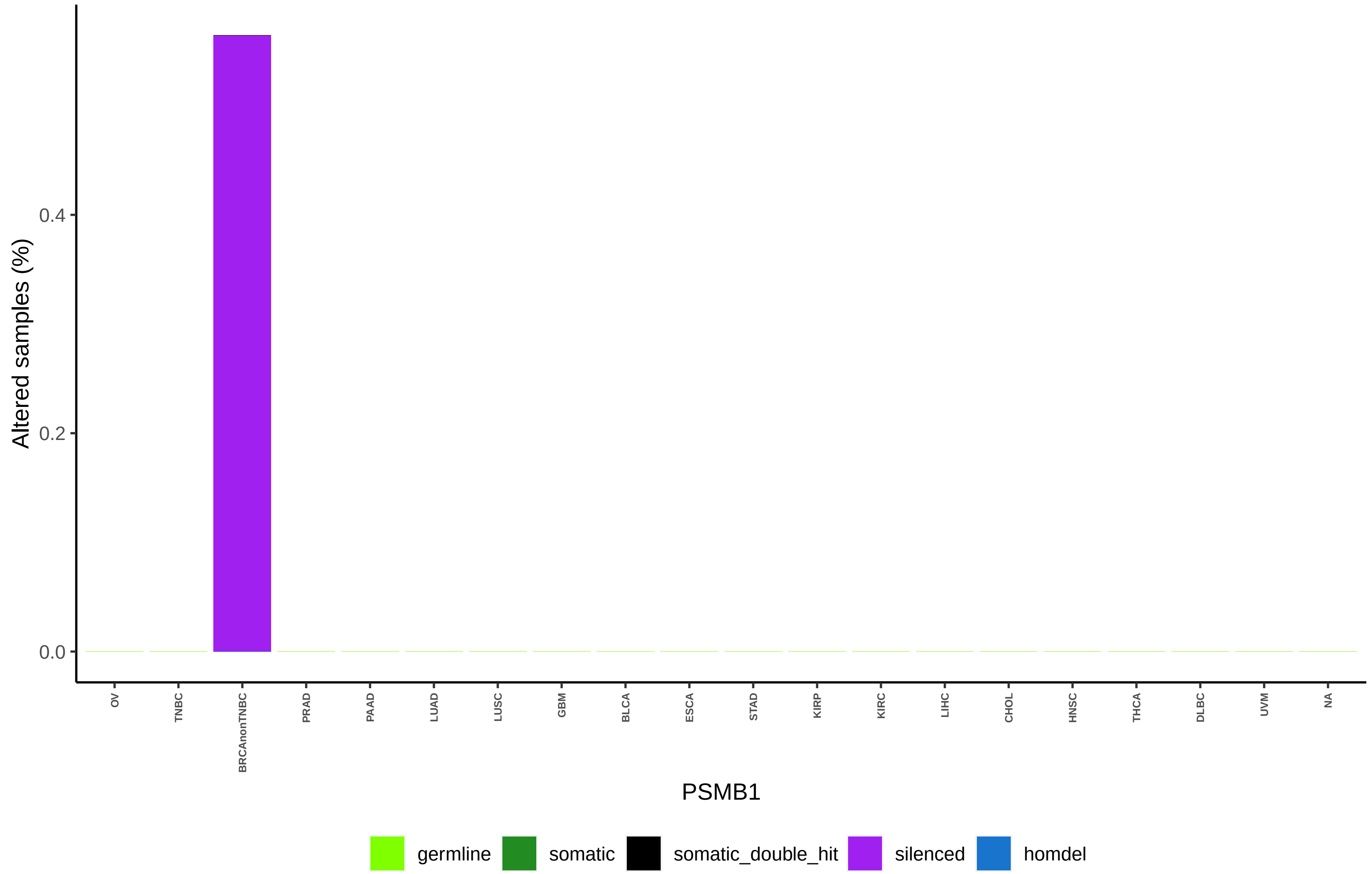
Altered samples (%)
0.0
0.2
0.4
OV
TNBC
BRCAnonTNBC
PRAD
PAAD
LUAD
LUSC
GBM
BLCA
ESCA
STAD
KIRP
KIRC
LIHC
CHOL
HNSC
THCA
DLBC
UVM
NA
PSMB1
germline
somatic
somatic_double_hit
silenced
homdel

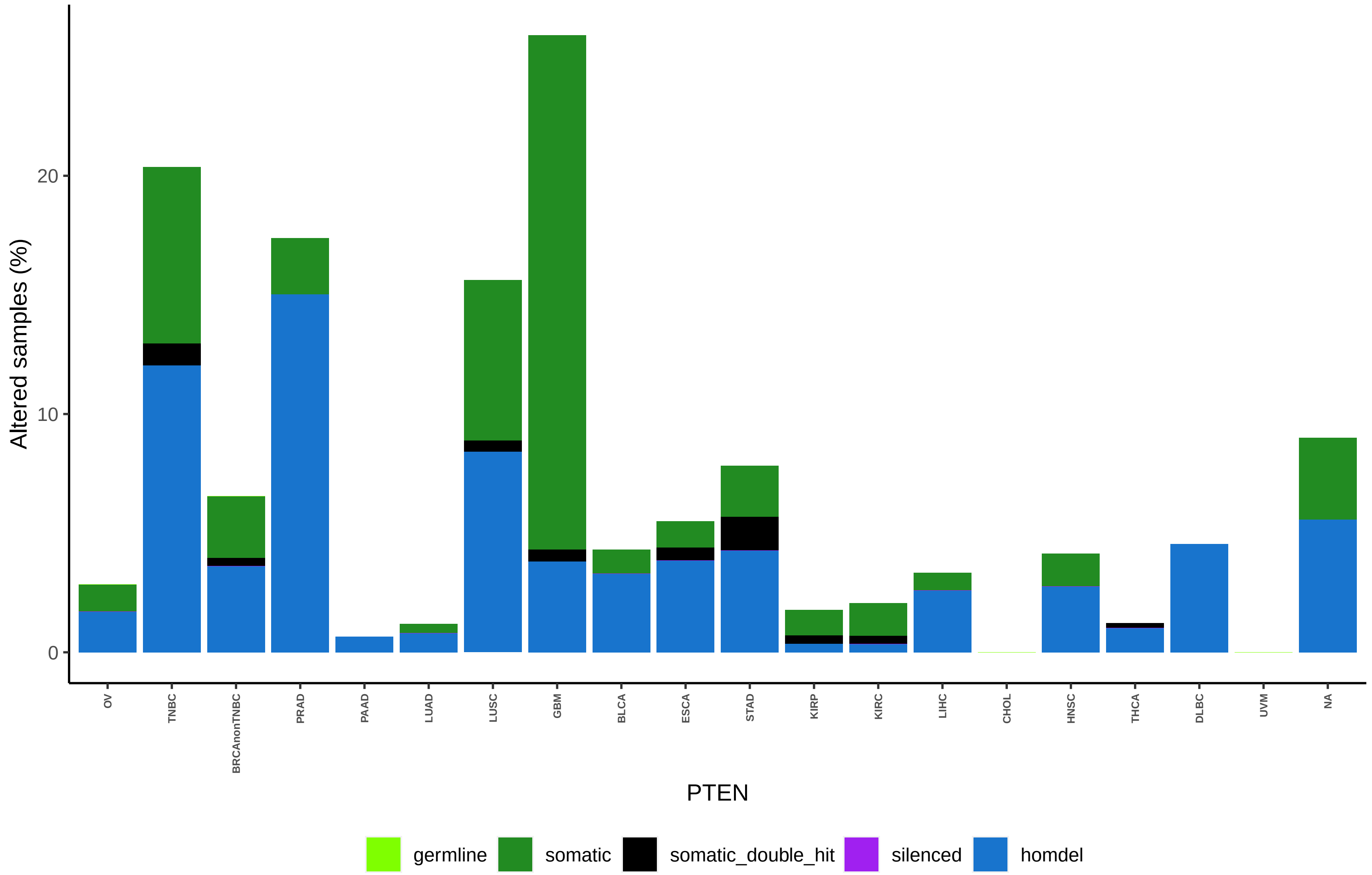
Altered samples (%)
0
10
20
OV
TNBC
BRCAnonTNBC
PRAD
PAAD
LUAD
LUSC
GBM
BLCA
ESCA
STAD
KIRP
KIRC
LIHC
CHOL
HNSC
THCA
DLBC
UVM
NA
PTEN
germline
somatic
somatic_double_hit
silenced
homdel

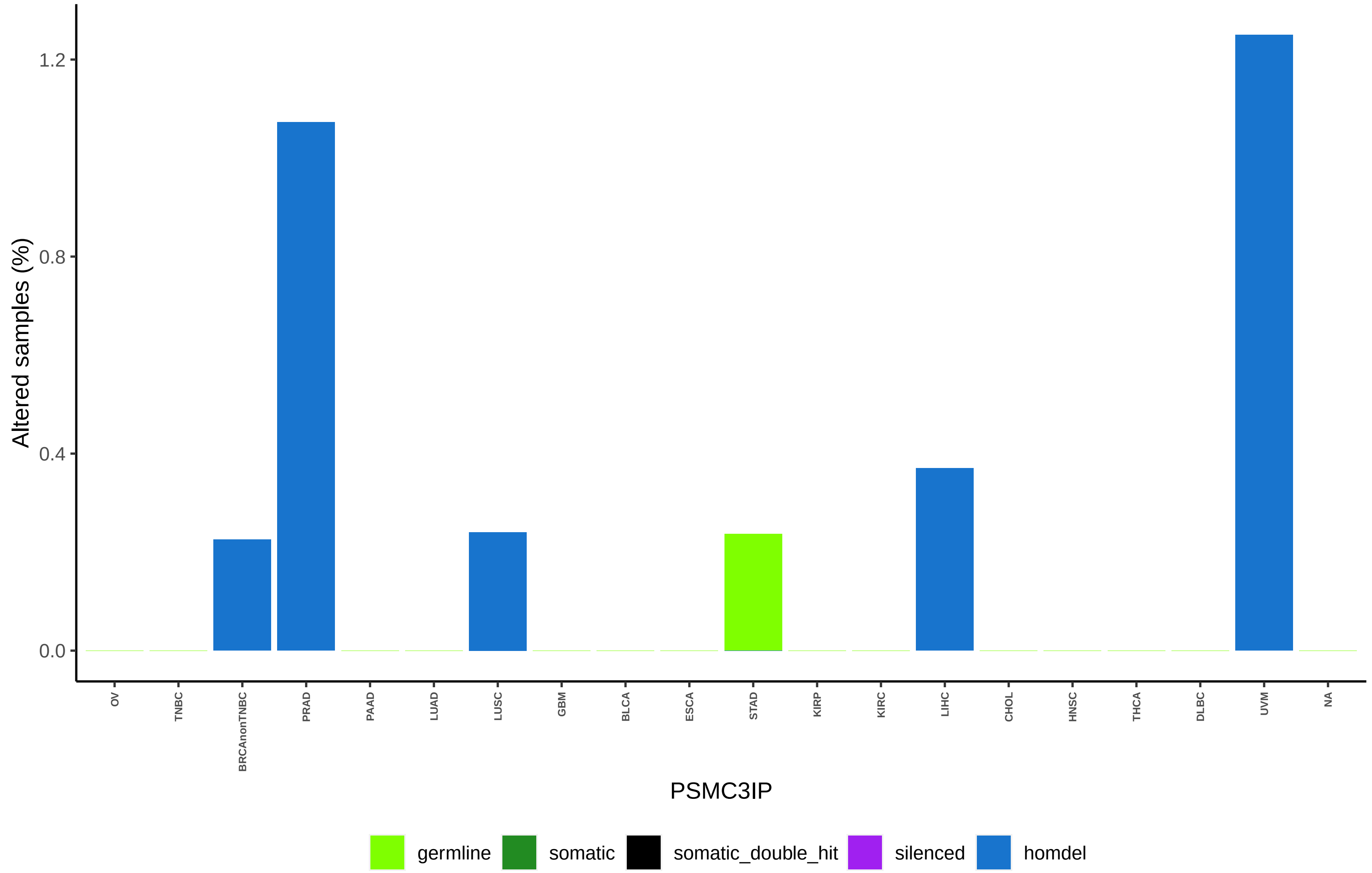
Altered samples (%)
0.0
0.4
0.8
1.2
OV
TNBC
BRCAnonTNBC
PRAD
PAAD
LUAD
LUSC
GBM
BLCA
ESCA
STAD
KIRP
KIRC
LIHC
CHOL
HNSC
THCA
DLBC
UVM
NA
PSMC3IP
germline
somatic
somatic_double_hit
silenced
homdel

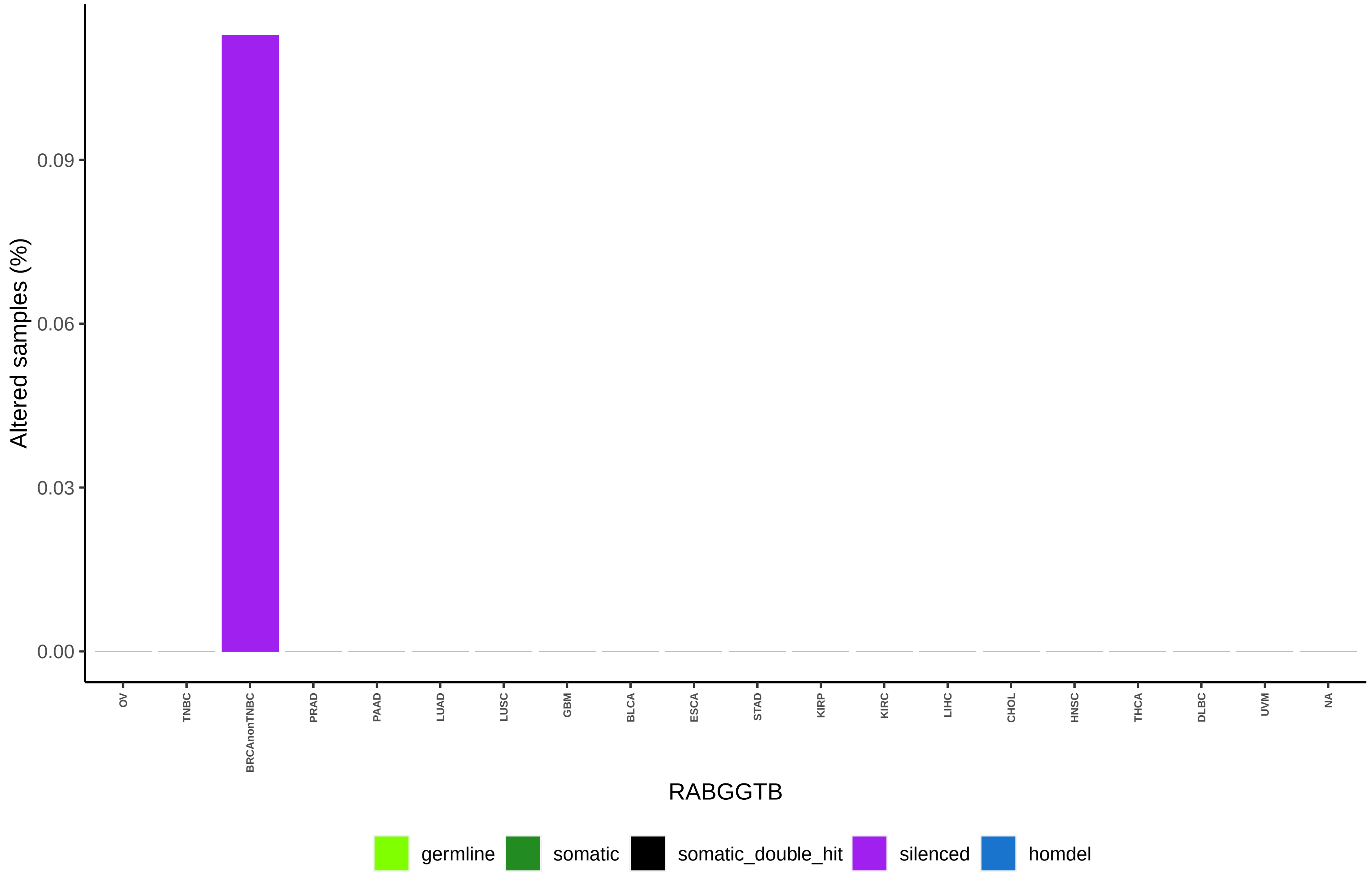
Altered samples (%)
0.00
0.03
0.06
0.09
OV
TNBC
BRCAnonTNBC
PRAD
PAAD
LUAD
LUSC
GBM
BLCA
ESCA
STAD
KIRP
KIRC
LIHC
CHOL
HNSC
THCA
DLBC
UVM
NA
RABGGTB
germline
somatic
somatic_double_hit
silenced
homdel

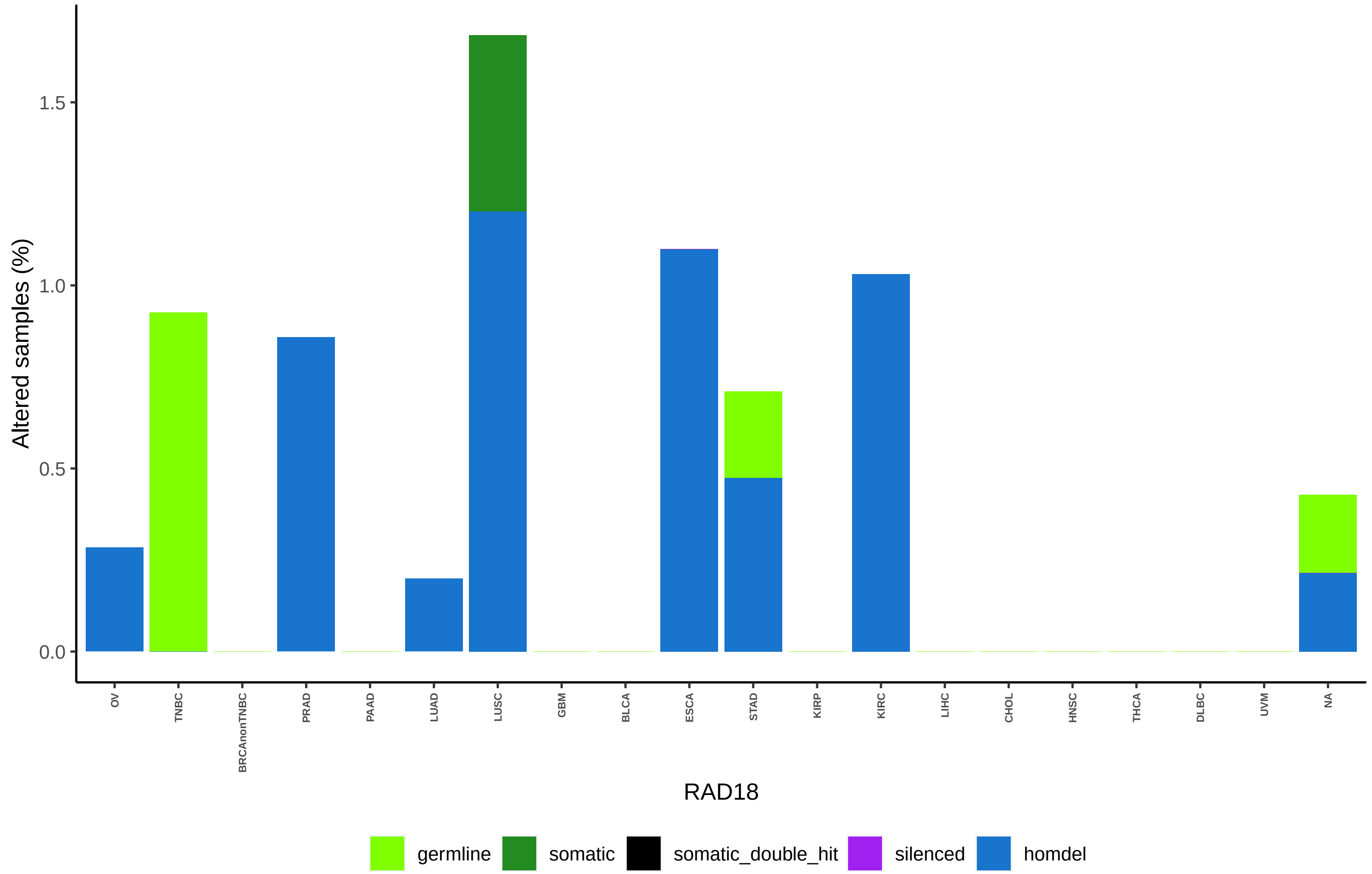
Altered samples (%)
0.0
0.5
1.0
1.5
OV
TNBC
BRCAnonTNBC
PRAD
PAAD
LUAD
LUSC
GBM
BLCA
ESCA
STAD
KIRP
KIRC
LIHC
CHOL
HNSC
THCA
DLBC
UVM
NA
RAD18
germline
somatic
somatic_double_hit
silenced
homdel

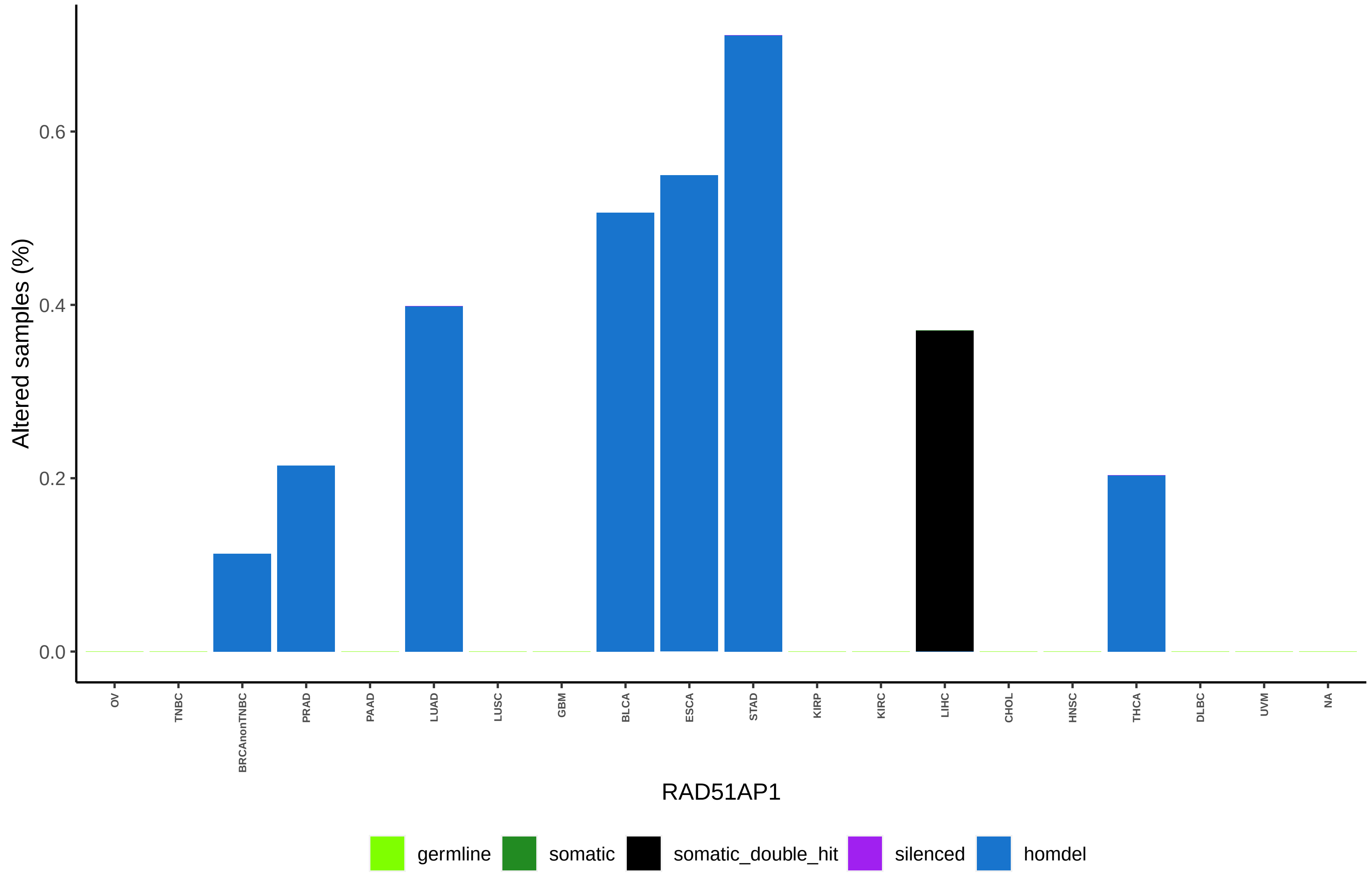
Altered samples (%)
0.0
0.2
0.4
0.6
OV
TNBC
BRCAnonTNBC
PRAD
PAAD
LUAD
LUSC
GBM
BLCA
ESCA
STAD
KIRP
KIRC
LIHC
CHOL
HNSC
THCA
DLBC
UVM
NA
RAD51AP1
germline
somatic
somatic_double_hit
silenced
homdel

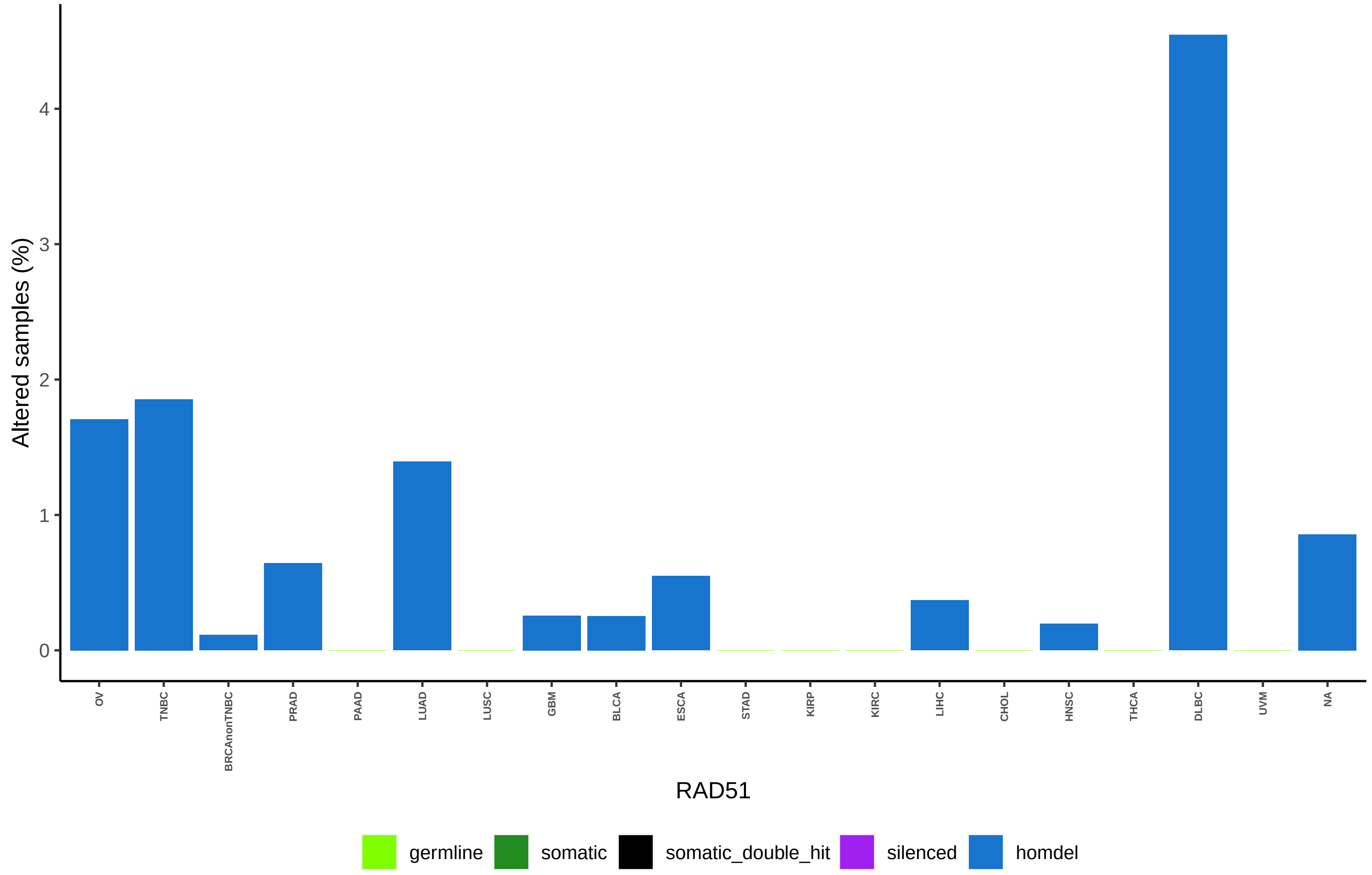
Altered samples (%)
0
1
2
3
4
OV
TNBC
BRCAnonTNBC
PRAD
PAAD
LUAD
LUSC
GBM
BLCA
ESCA
STAD
KIRP
KIRC
LIHC
CHOL
HNSC
THCA
DLBC
UVM
NA
RAD51
germline
somatic
somatic_double_hit
silenced
homdel

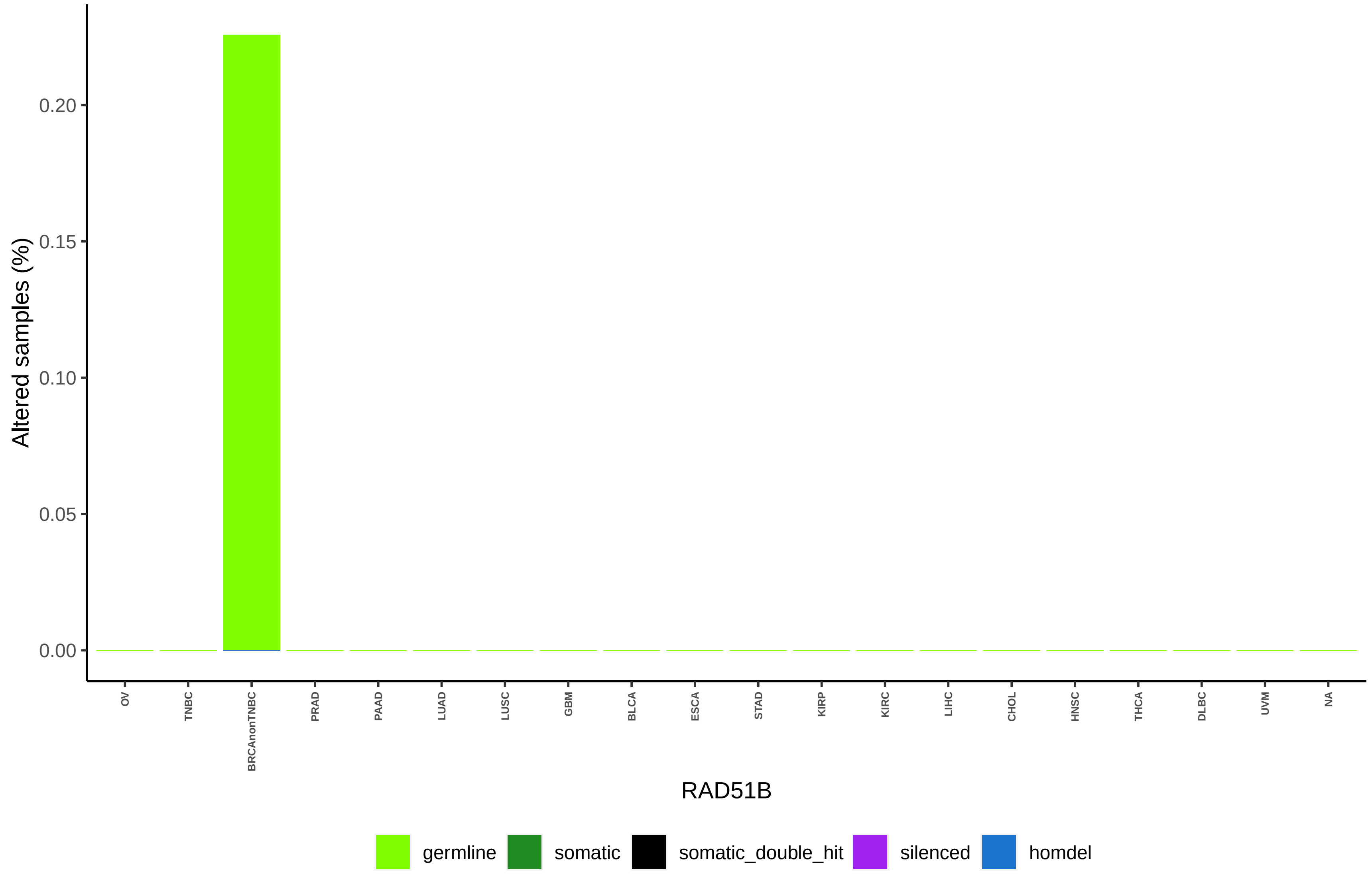
Altered samples (%)
0.00
0.05
0.10
0.15
0.20
OV
TNBC
BRCAnonTNBC
PRAD
PAAD
LUAD
LUSC
GBM
BLCA
ESCA
STAD
KIRP
KIRC
LIHC
CHOL
HNSC
THCA
DLBC
UVM
NA
RAD51B
germline
somatic
somatic_double_hit
silenced
homdel

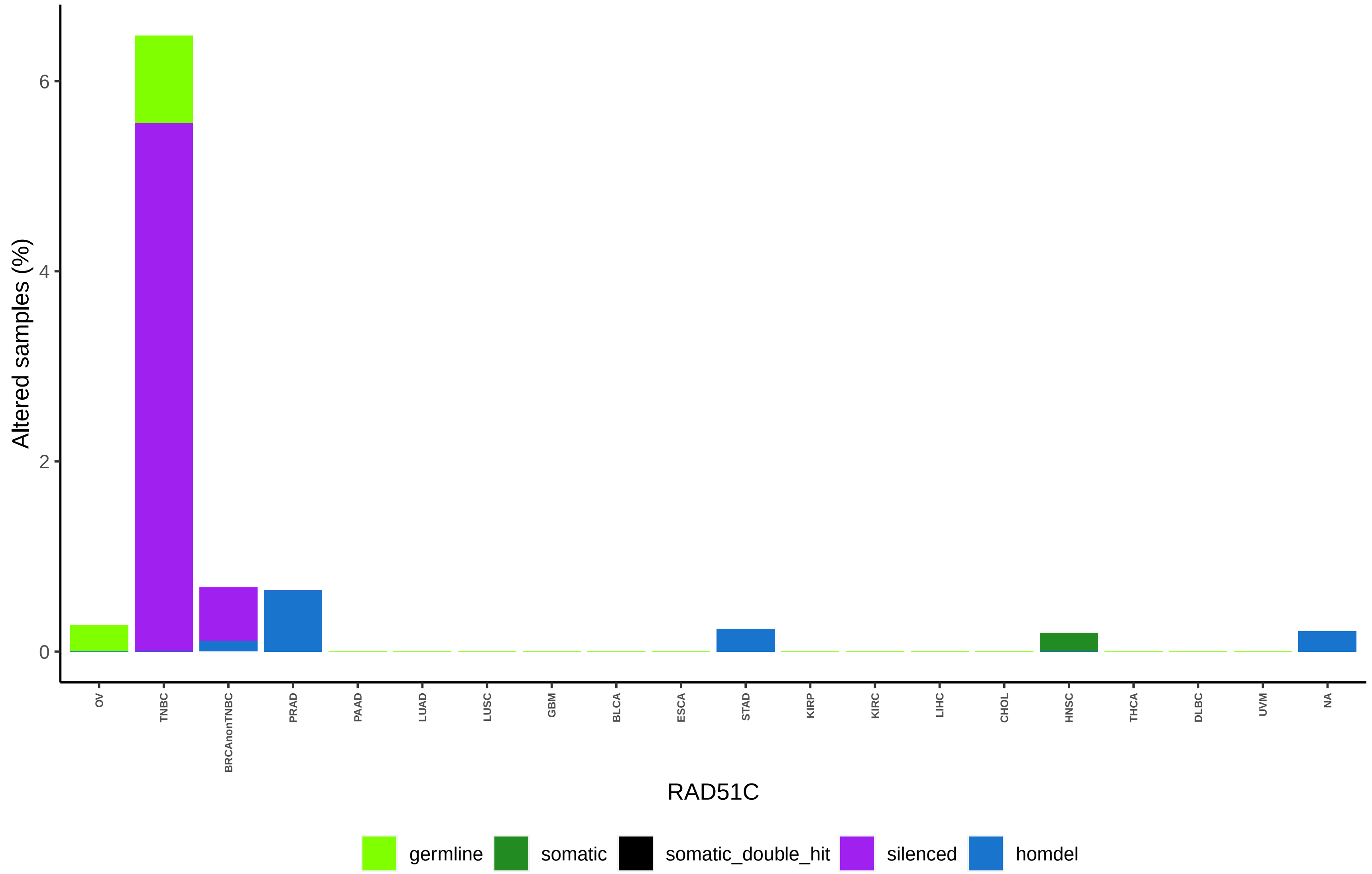
Altered samples (%)
0
2
4
6
OV
TNBC
BRCAnonTNBC
PRAD
PAAD
LUAD
LUSC
GBM
BLCA
ESCA
STAD
KIRP
KIRC
LIHC
CHOL
HNSC
THCA
DLBC
UVM
NA
RAD51C
germline
somatic
somatic_double_hit
silenced
homdel

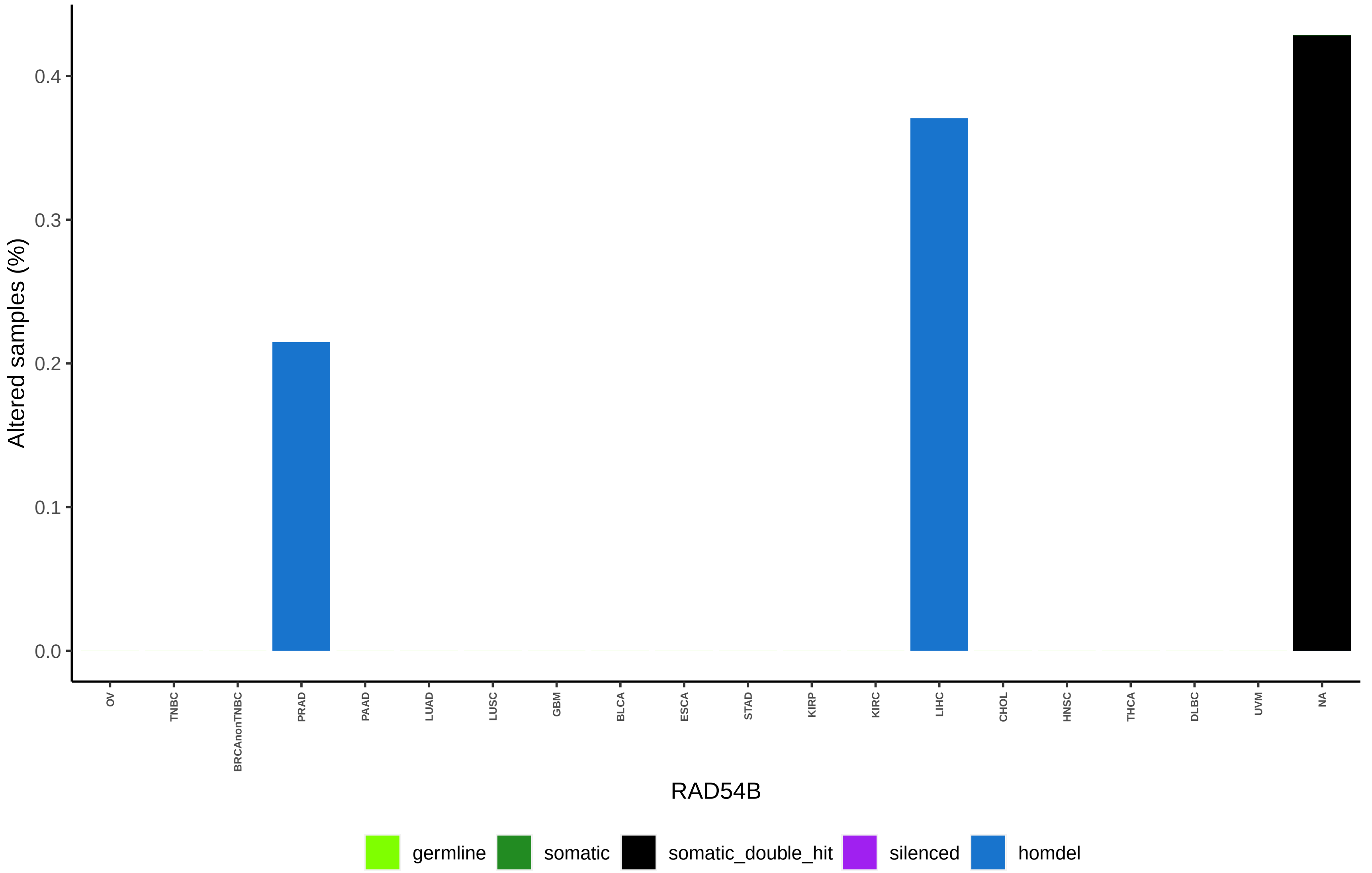
Altered samples (%)
0.0
0.1
0.2
0.3
0.4
OV
TNBC
BRCAnonTNBC
PRAD
PAAD
LUAD
LUSC
GBM
BLCA
ESCA
STAD
KIRP
KIRC
LIHC
CHOL
HNSC
THCA
DLBC
UVM
NA
RAD54B
germline
somatic
somatic_double_hit
silenced
homdel

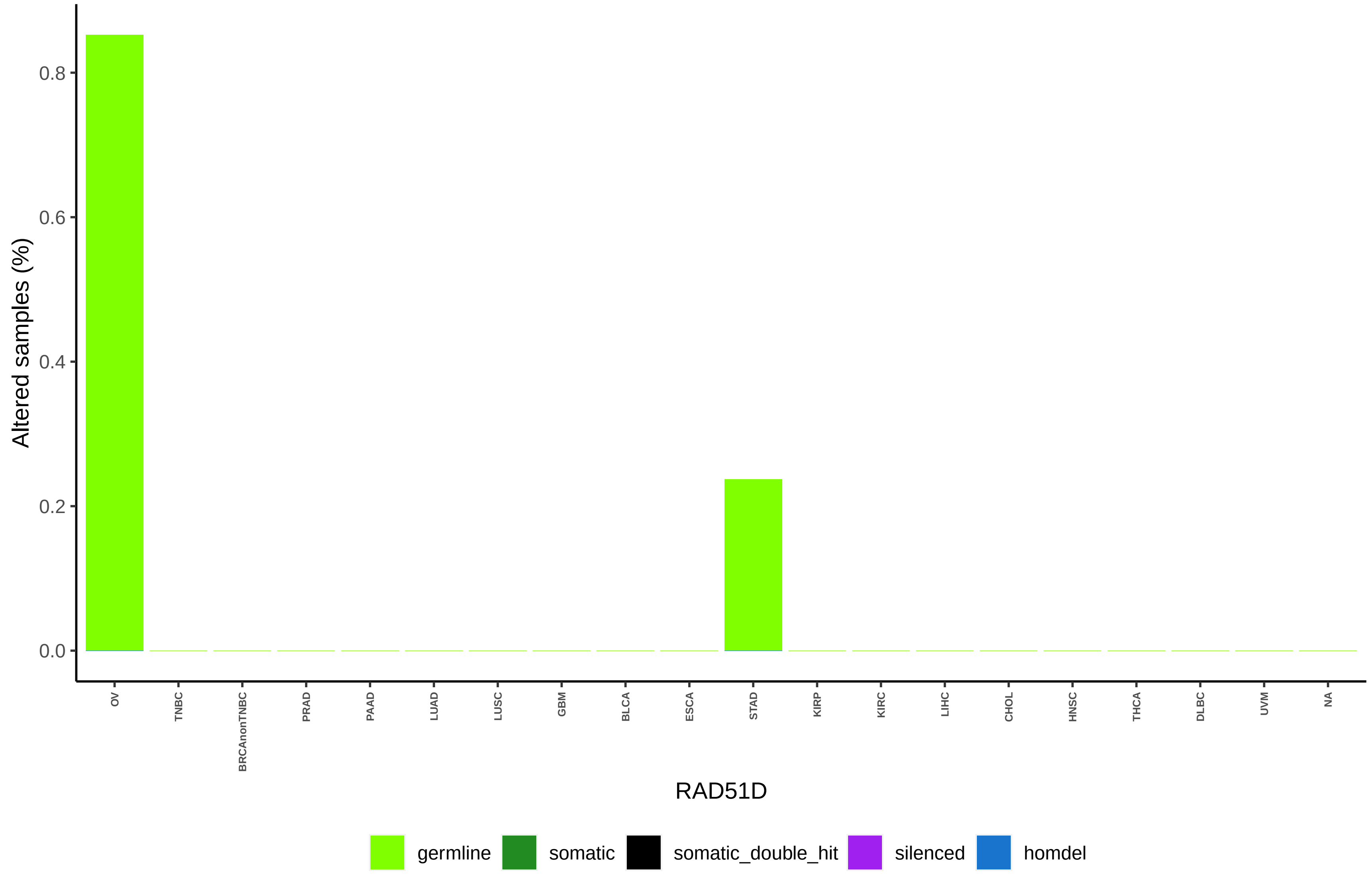
Altered samples (%)
0.0
0.2
0.4
0.6
0.8
OV
TNBC
BRCAnonTNBC
PRAD
PAAD
LUAD
LUSC
GBM
BLCA
ESCA
STAD
KIRP
KIRC
LIHC
CHOL
HNSC
THCA
DLBC
UVM
NA
RAD51D
germline
somatic
somatic_double_hit
silenced
homdel

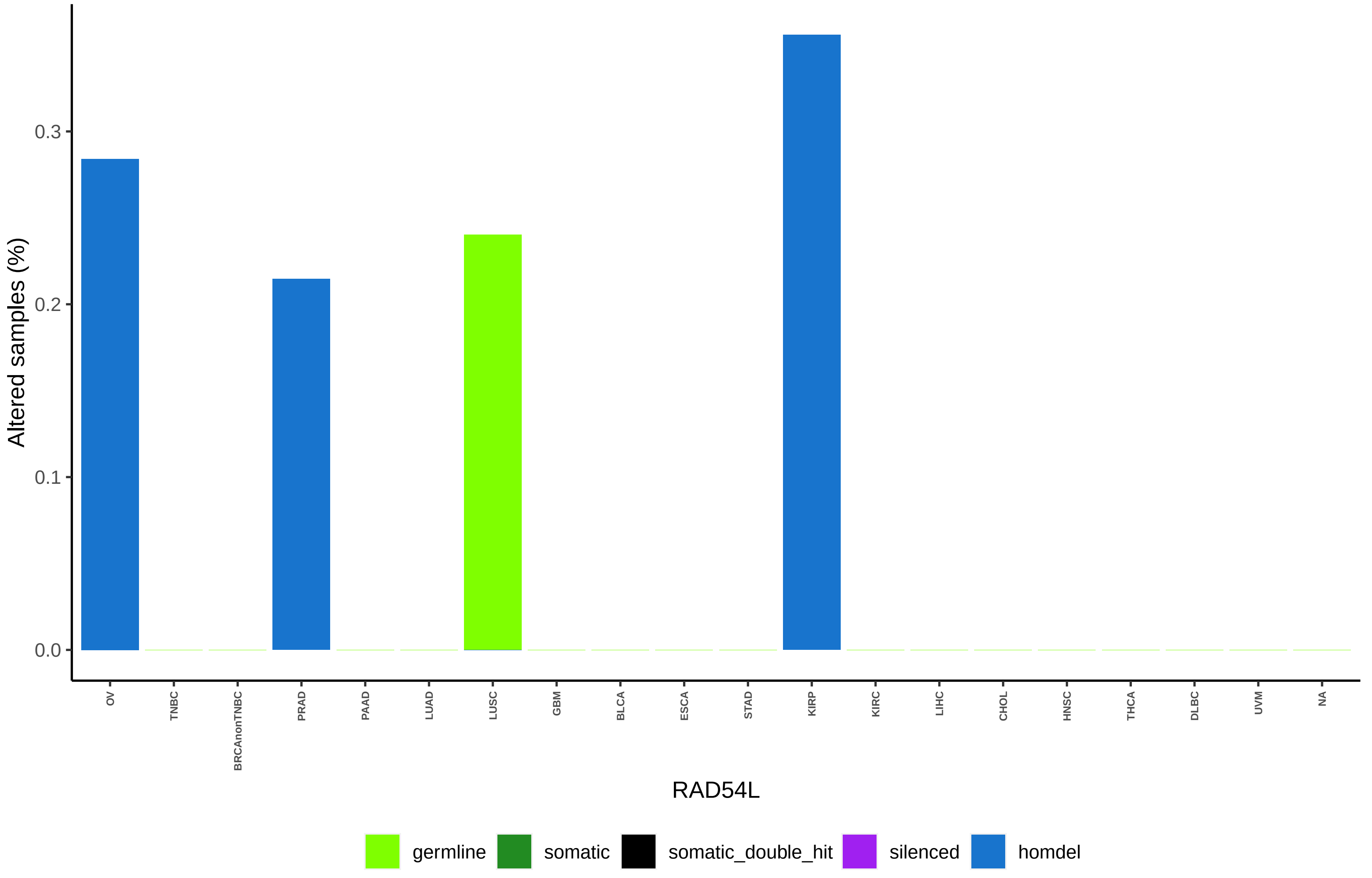
Altered samples (%)
0.0
0.1
0.2
0.3
OV
TNBC
BRCAnonTNBC
PRAD
PAAD
LUAD
LUSC
GBM
BLCA
ESCA
STAD
KIRP
KIRC
LIHC
CHOL
HNSC
THCA
DLBC
UVM
NA
RAD54L
germline
somatic
somatic_double_hit
silenced
homdel

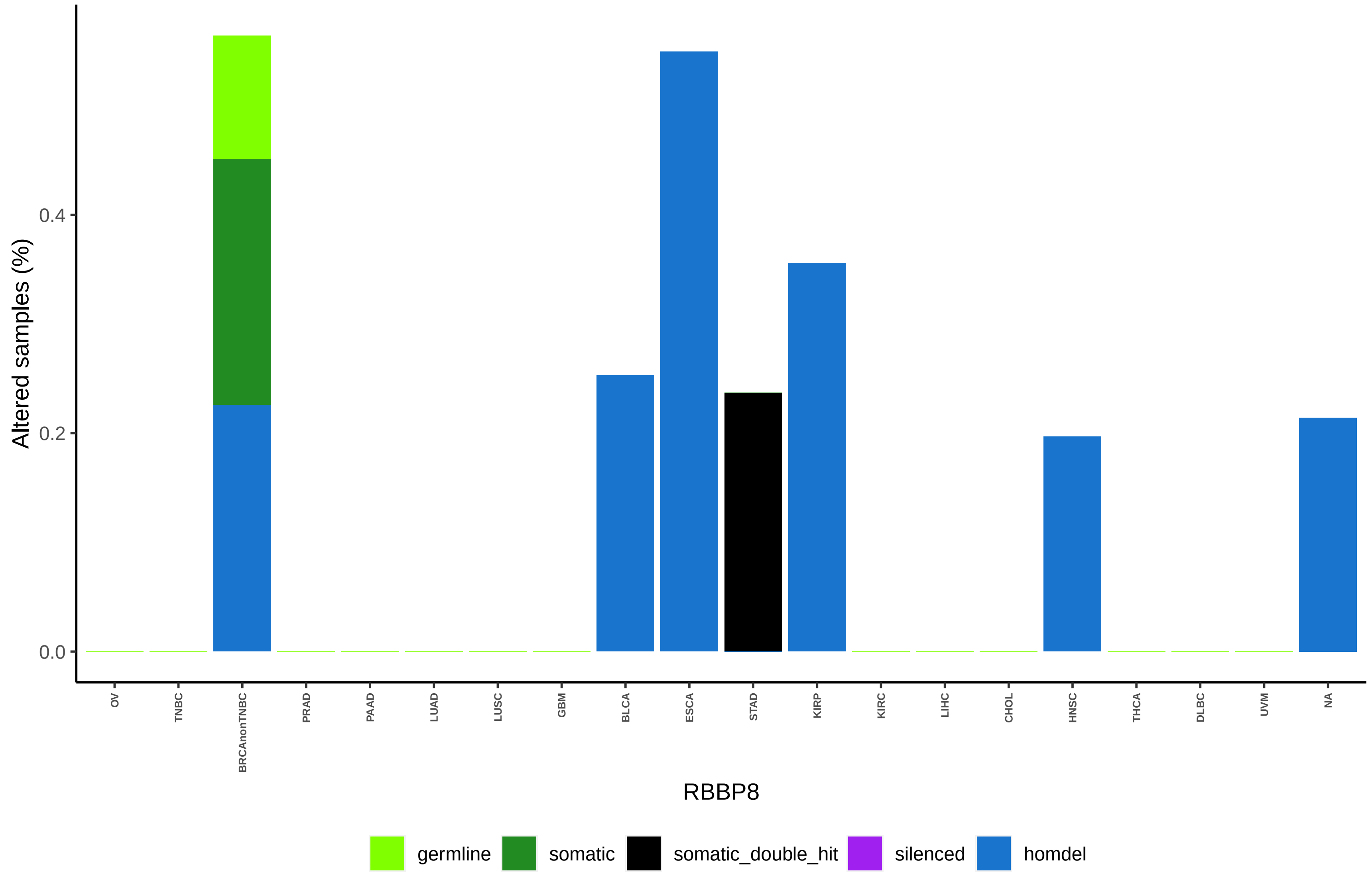
Altered samples (%)
0.0
0.2
0.4
OV
TNBC
BRCAnonTNBC
PRAD
PAAD
LUAD
LUSC
GBM
BLCA
ESCA
STAD
KIRP
KIRC
LIHC
CHOL
HNSC
THCA
DLBC
UVM
NA
RBBP8
germline
somatic
somatic_double_hit
silenced
homdel

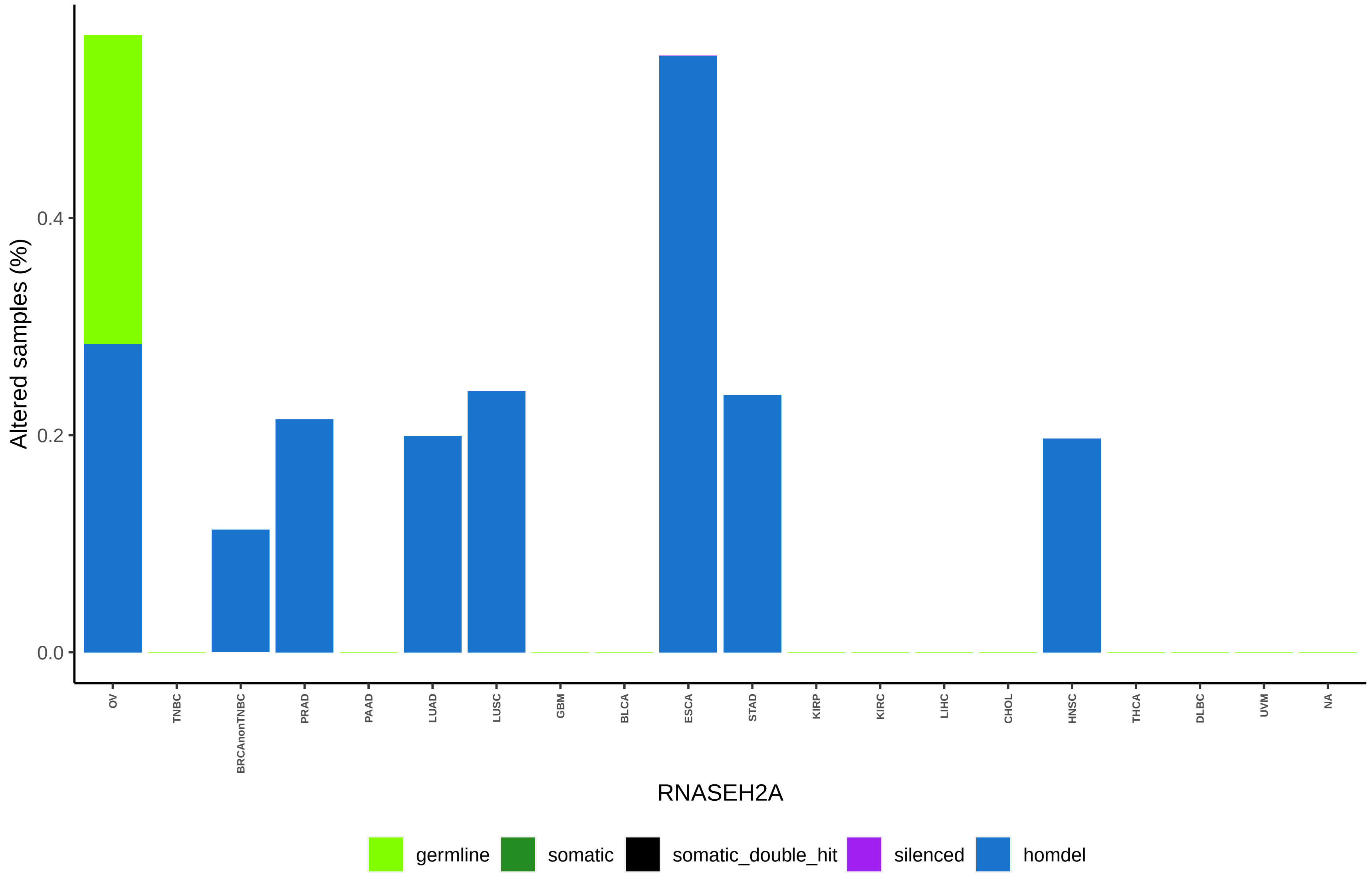
Altered samples (%)
0.0
0.2
0.4
OV
TNBC
BRCAnonTNBC
PRAD
PAAD
LUAD
LUSC
GBM
BLCA
ESCA
STAD
KIRP
KIRC
LIHC
CHOL
HNSC
THCA
DLBC
UVM
NA
RNASEH2A
germline
somatic
somatic_double_hit
silenced
homdel

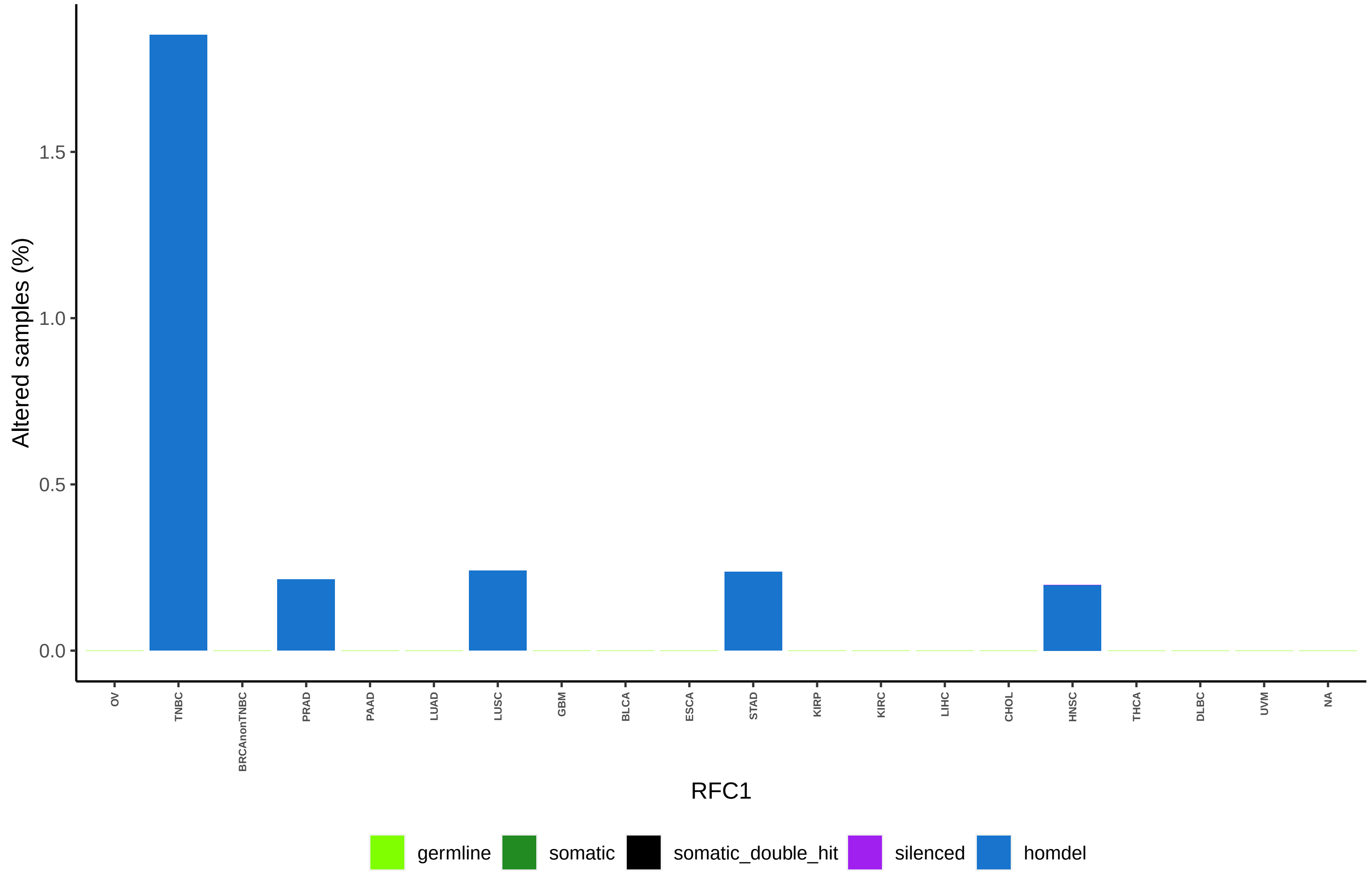
Altered samples (%)
0.0
0.5
1.0
1.5
OV
TNBC
BRCAnonTNBC
PRAD
PAAD
LUAD
LUSC
GBM
BLCA
ESCA
STAD
KIRP
KIRC
LIHC
CHOL
HNSC
THCA
DLBC
UVM
NA
RFC1
germline
somatic
somatic_double_hit
silenced
homdel

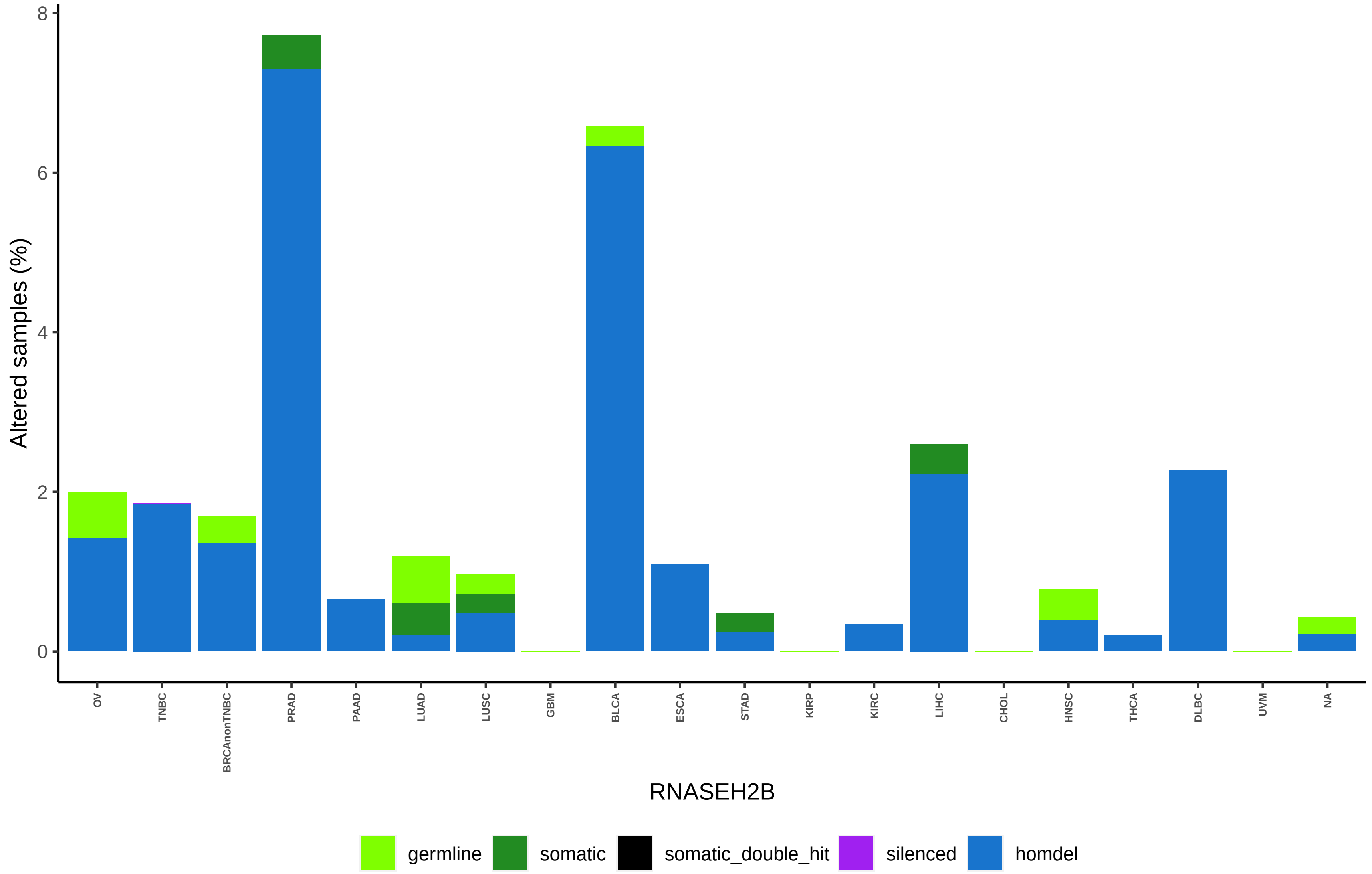
Altered samples (%)
0
2
4
6
8
OV
TNBC
BRCAnonTNBC
PRAD
PAAD
LUAD
LUSC
GBM
BLCA
ESCA
STAD
KIRP
KIRC
LIHC
CHOL
HNSC
THCA
DLBC
UVM
NA
RNASEH2B
germline
somatic
somatic_double_hit
silenced
homdel

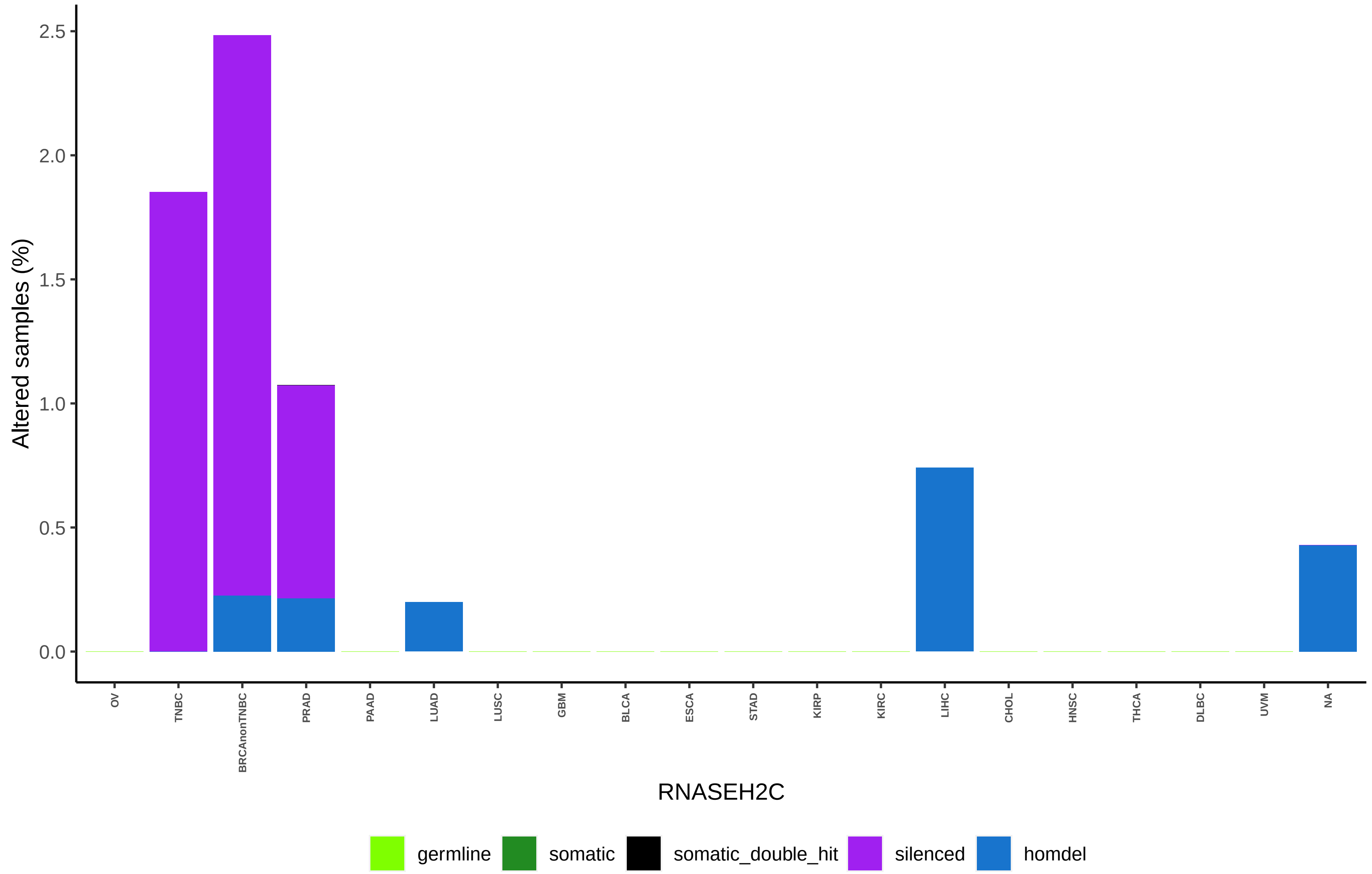
Altered samples (%)
0.0
0.5
1.0
1.5
2.0
2.5
OV TNBC BRCAnonTNBC PRAD PAAD LUAD LUSC GBM BLCA ESCA STAD KIRP KIRC LIHC CHOL HNSC THCA DLBC UVM NA
RNASEH2C
germline somatic somatic_double_hit silenced homdel

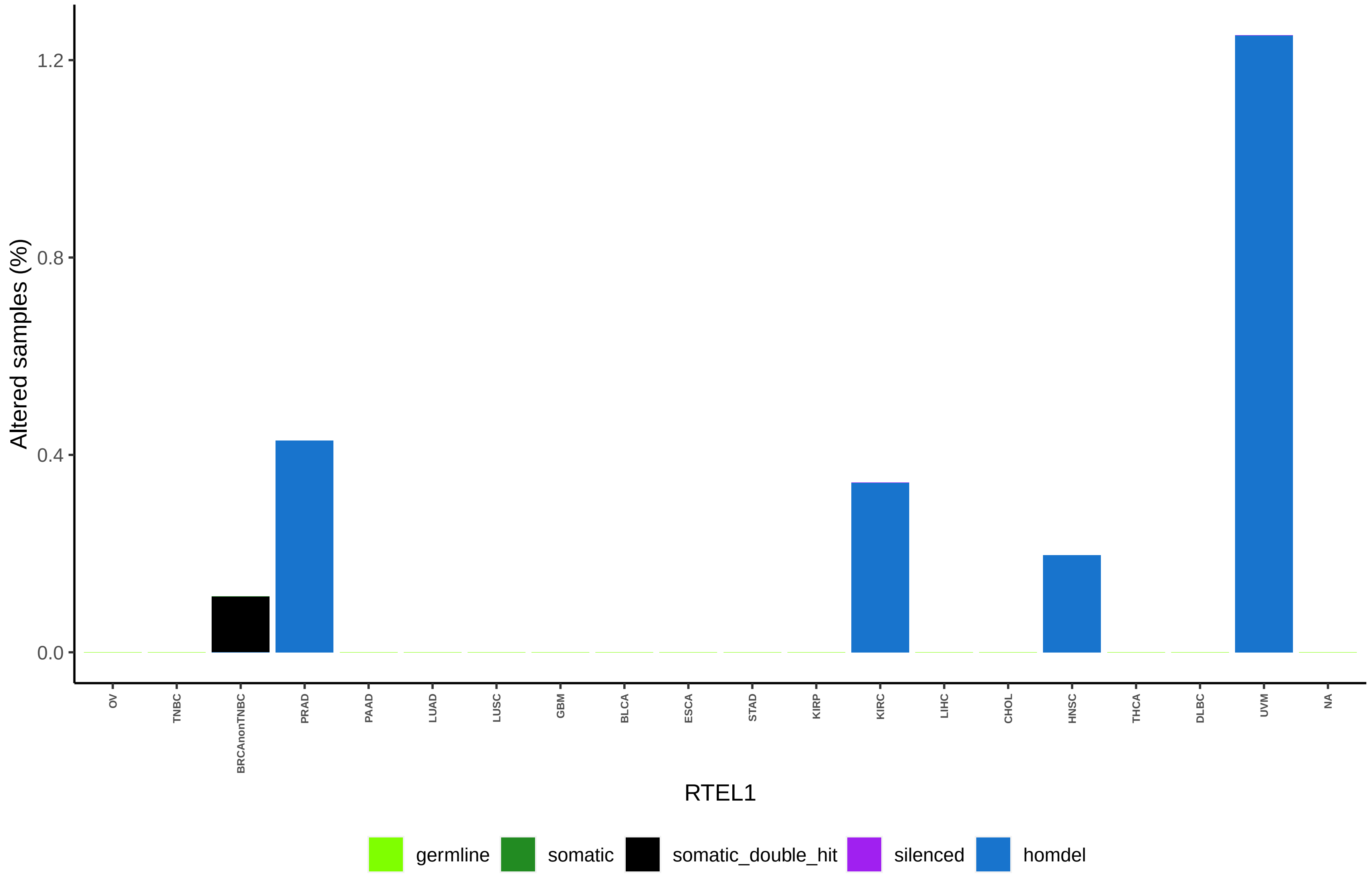
Altered samples (%)
0.0
0.4
0.8
1.2
OV TNBC BRCAnonTNBC PRAD PAAD LUAD LUSC GBM BLCA ESCA STAD KIRP KIRC LIHC CHOL HNSC THCA DLBC UVM NA
RTEL1
germline somatic somatic_double_hit silenced homdel

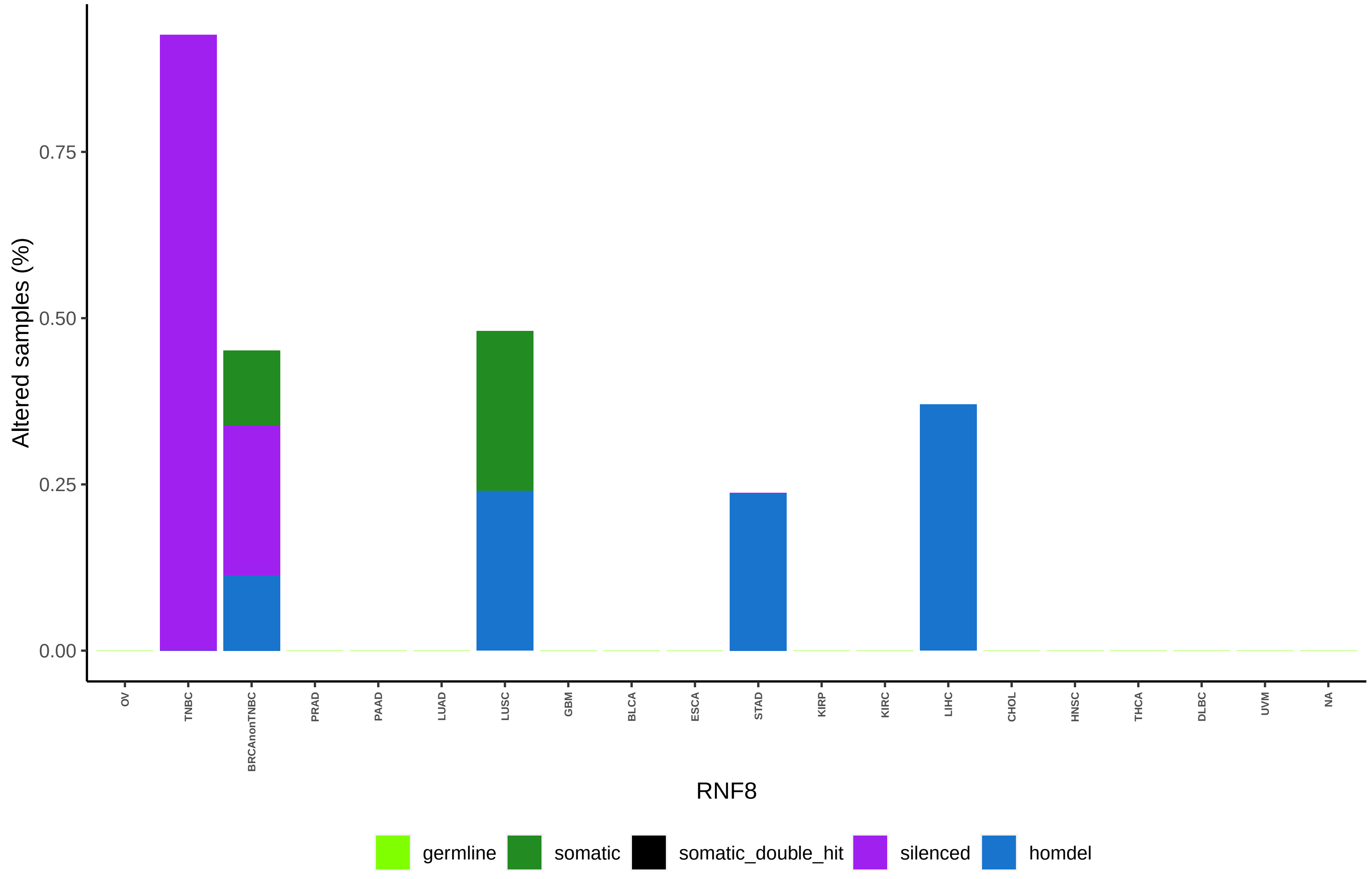
Altered samples (%)
0.00
0.25
0.50
0.75
OV TNBC BRCAnonTNBC PRAD PAAD LUAD LUSC GBM BLCA ESCA STAD KIRP KIRC LIHC CHOL HNSC THCA DLBC UVM NA
RNF8
germline somatic somatic_double_hit silenced homdel

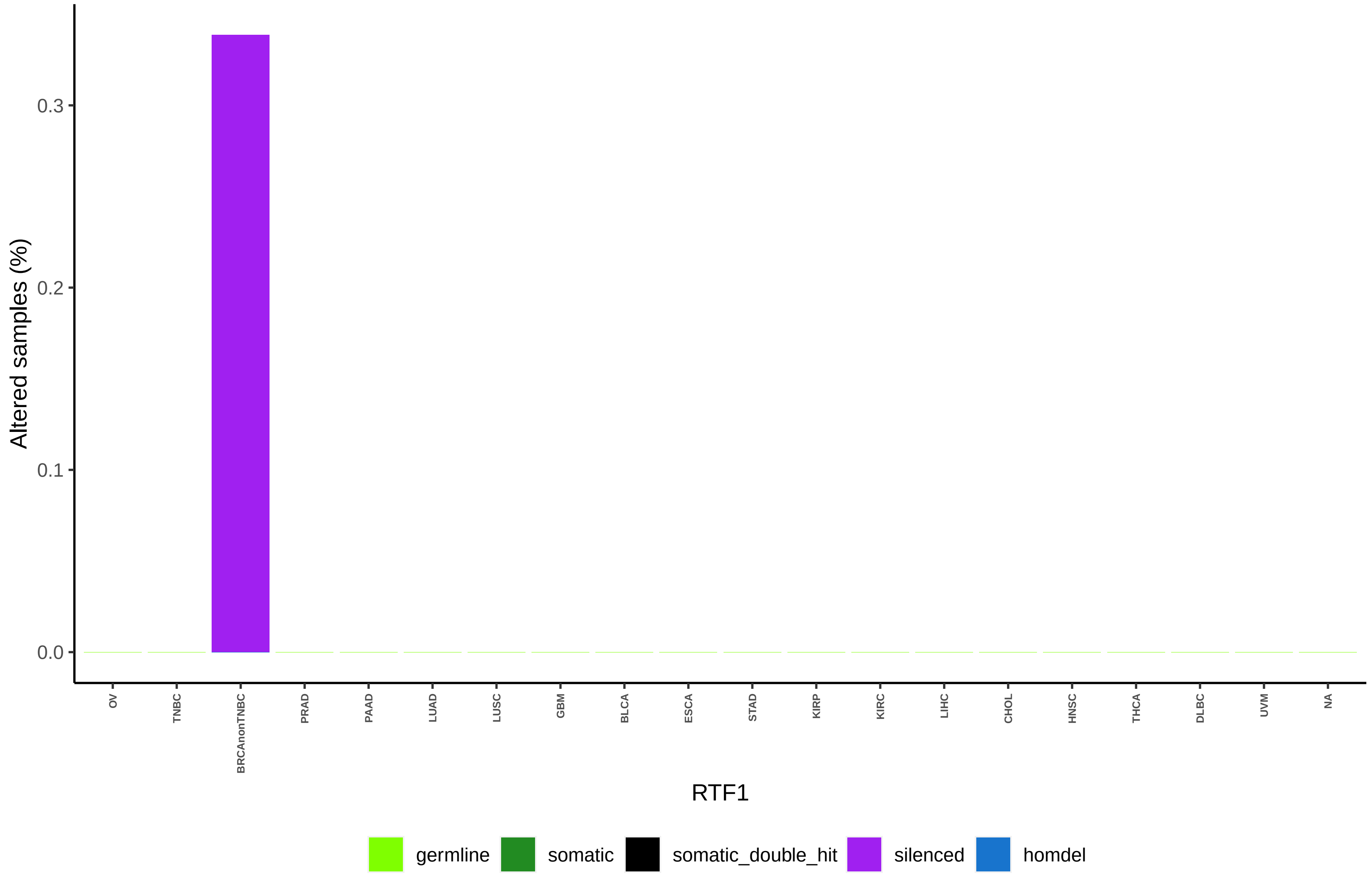
Altered samples (%)
0.0
0.1
0.2
0.3
OV TNBC BRCAnonTNBC PRAD PAAD LUAD LUSC GBM BLCA ESCA STAD KIRP KIRC LIHC CHOL HNSC THCA DLBC UVM NA
RTF1
germline somatic somatic_double_hit silenced homdel

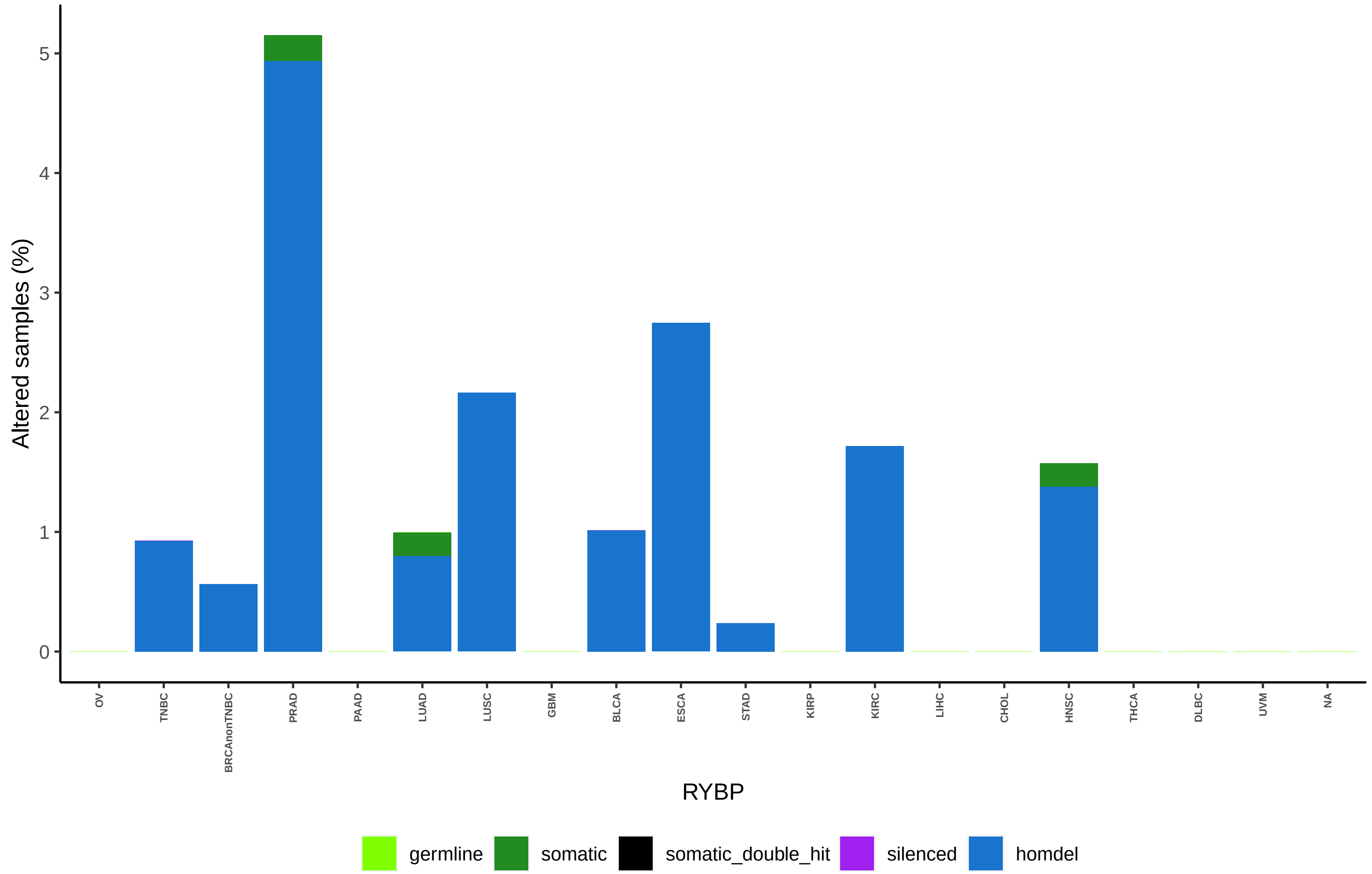
Altered samples (%)
0
1
2
3
4
5
OV
TNBC
BRCAnonTNBC
PRAD
PAAD
LUAD
LUSC
GBM
BLCA
ESCA
STAD
KIRP
KIRC
LIHC
CHOL
HNSC
THCA
DLBC
UVM
NA
RYBP
germline
somatic
somatic_double_hit
silenced
homdel

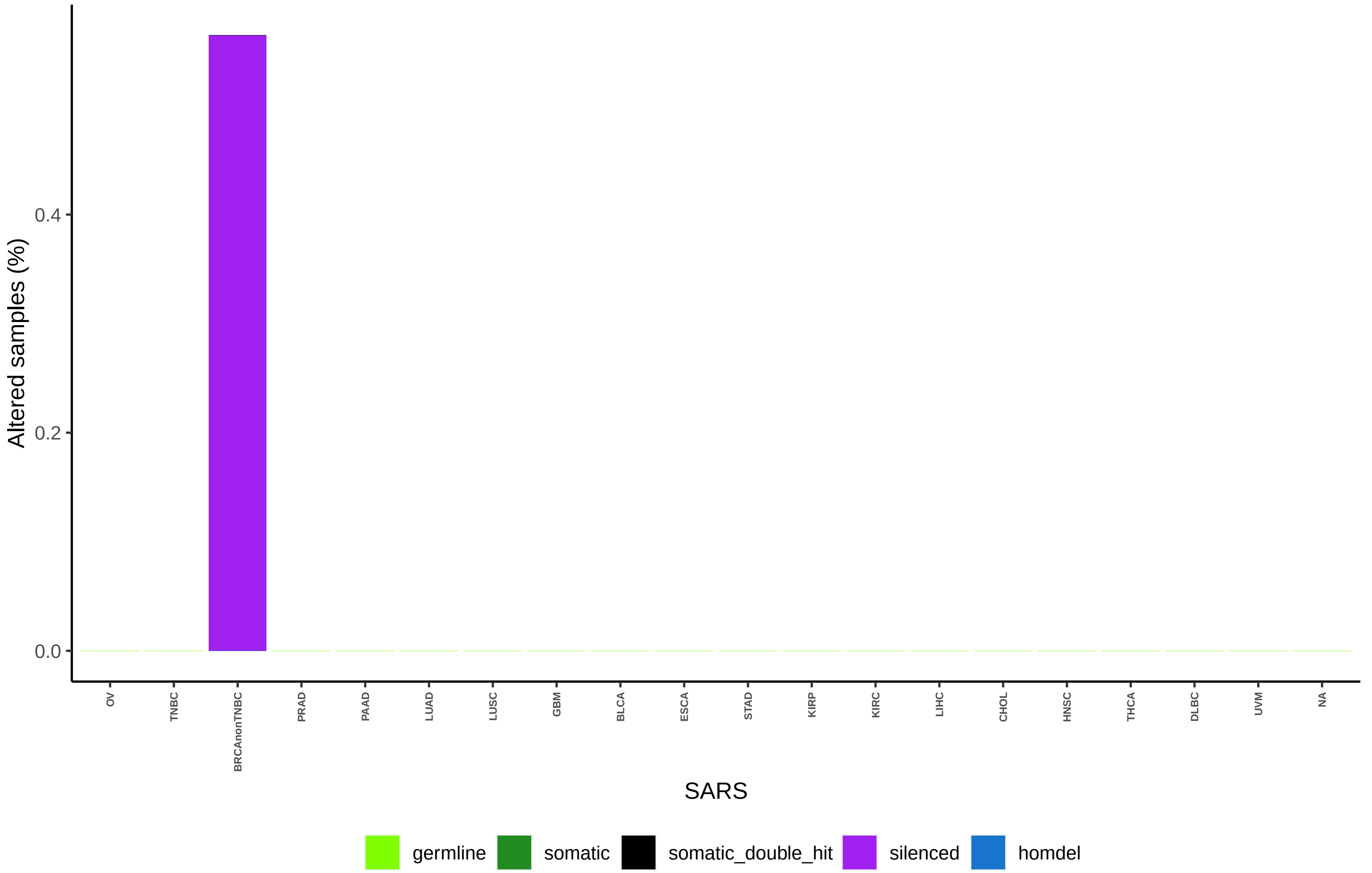
Altered samples (%)
0.0
0.2
0.4
OV
TNBC
BRCAnonTNBC
PRAD
PAAD
LUAD
LUSC
GBM
BLCA
ESCA
STAD
KIRP
KIRC
LIHC
CHOL
HNSC
THCA
DLBC
UVM
NA
SARS
germline
somatic
somatic_double_hit
silenced
homdel

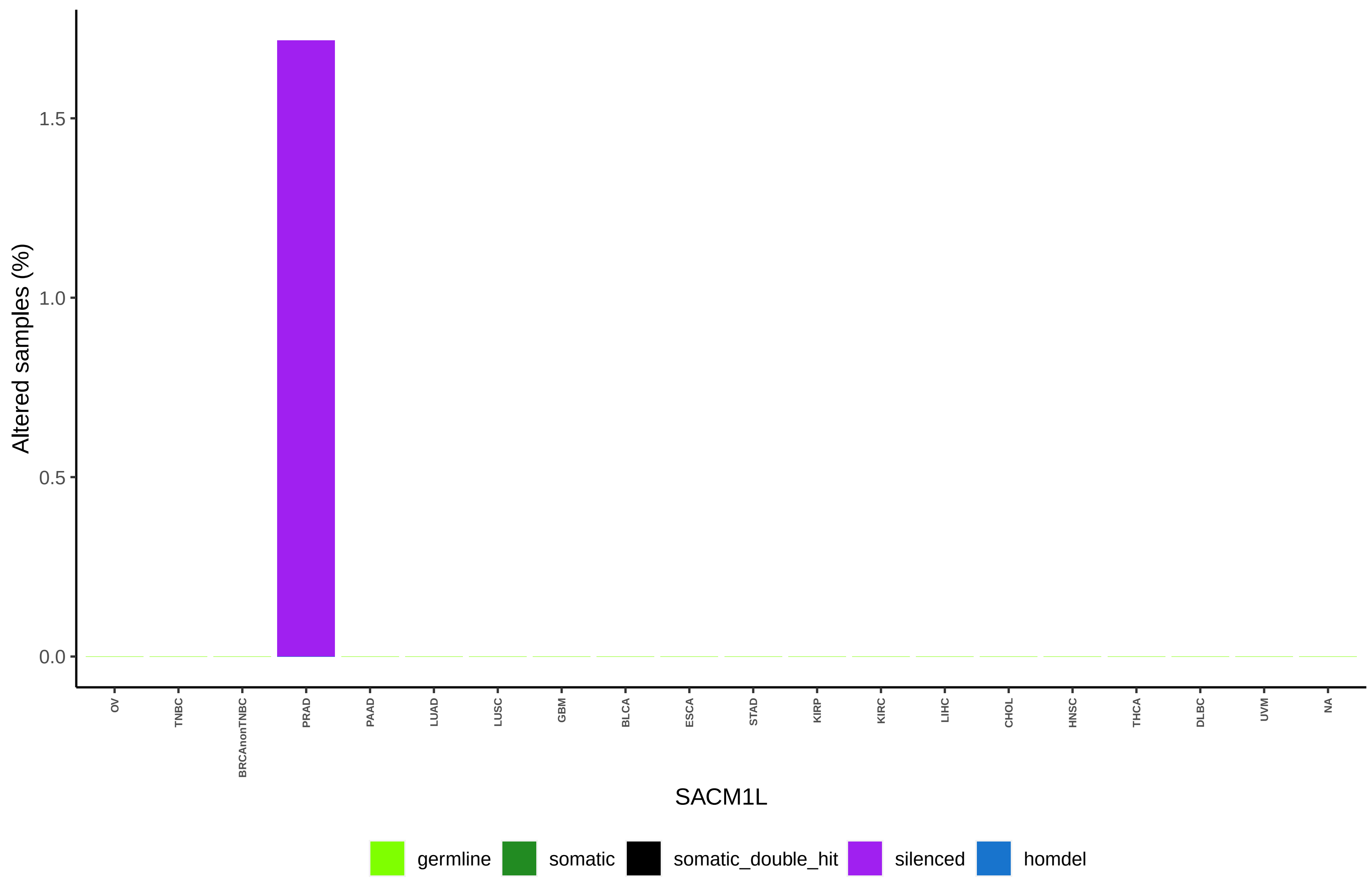
Altered samples (%)
0.0
0.5
1.0
1.5
OV
TNBC
BRCAnonTNBC
PRAD
PAAD
LUAD
LUSC
GBM
BLCA
ESCA
STAD
KIRP
KIRC
LIHC
CHOL
HNSC
THCA
DLBC
UVM
NA
SACM1L
germline
somatic
somatic_double_hit
silenced
homdel

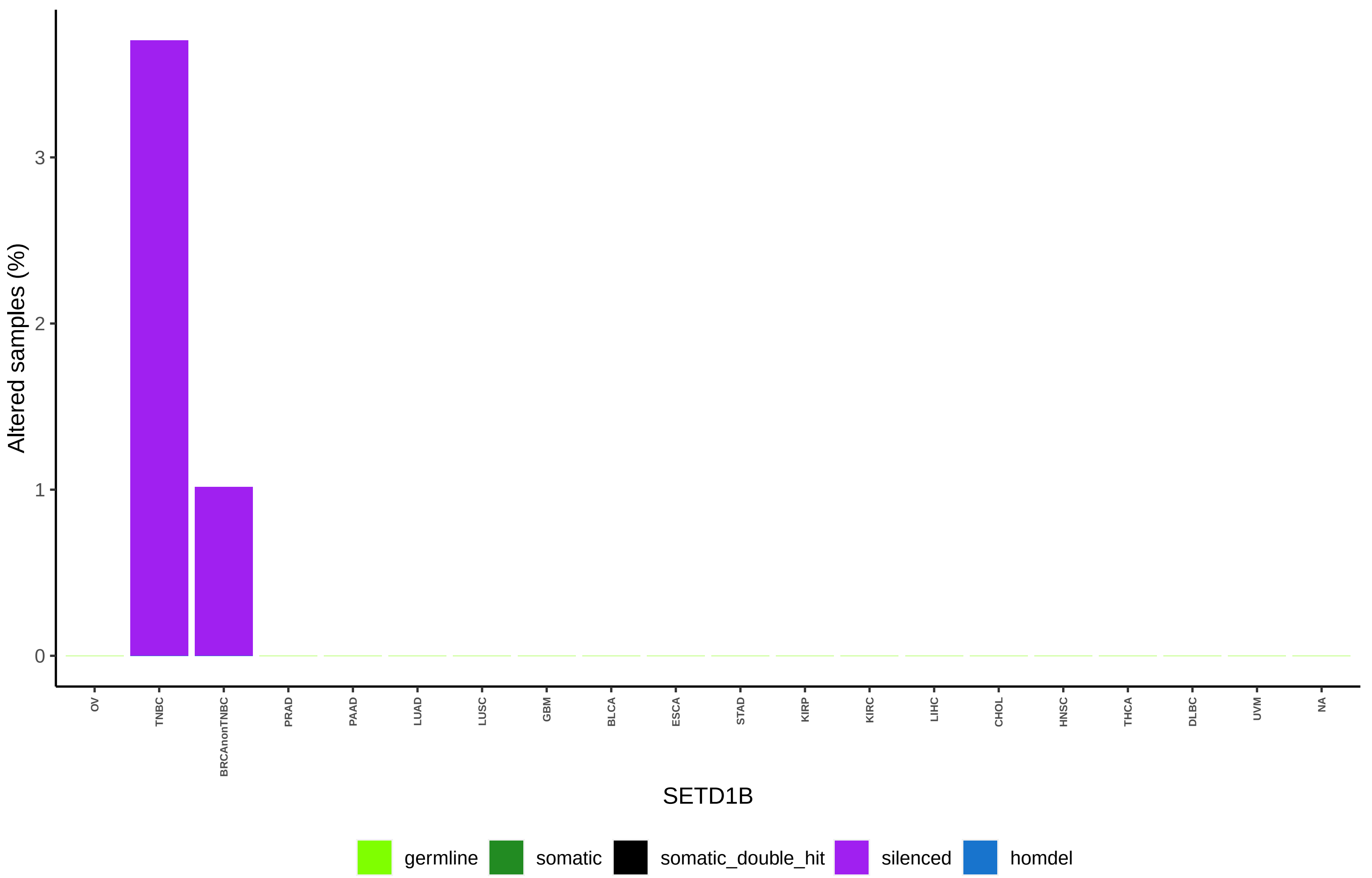
Altered samples (%)
0
1
2
3
OV
TNBC
BRCAnonTNBC
PRAD
PAAD
LUAD
LUSC
GBM
BLCA
ESCA
STAD
KIRP
KIRC
LIHC
CHOL
HNSC
THCA
DLBC
UVM
NA
SETD1B
germline
somatic
somatic_double_hit
silenced
homdel

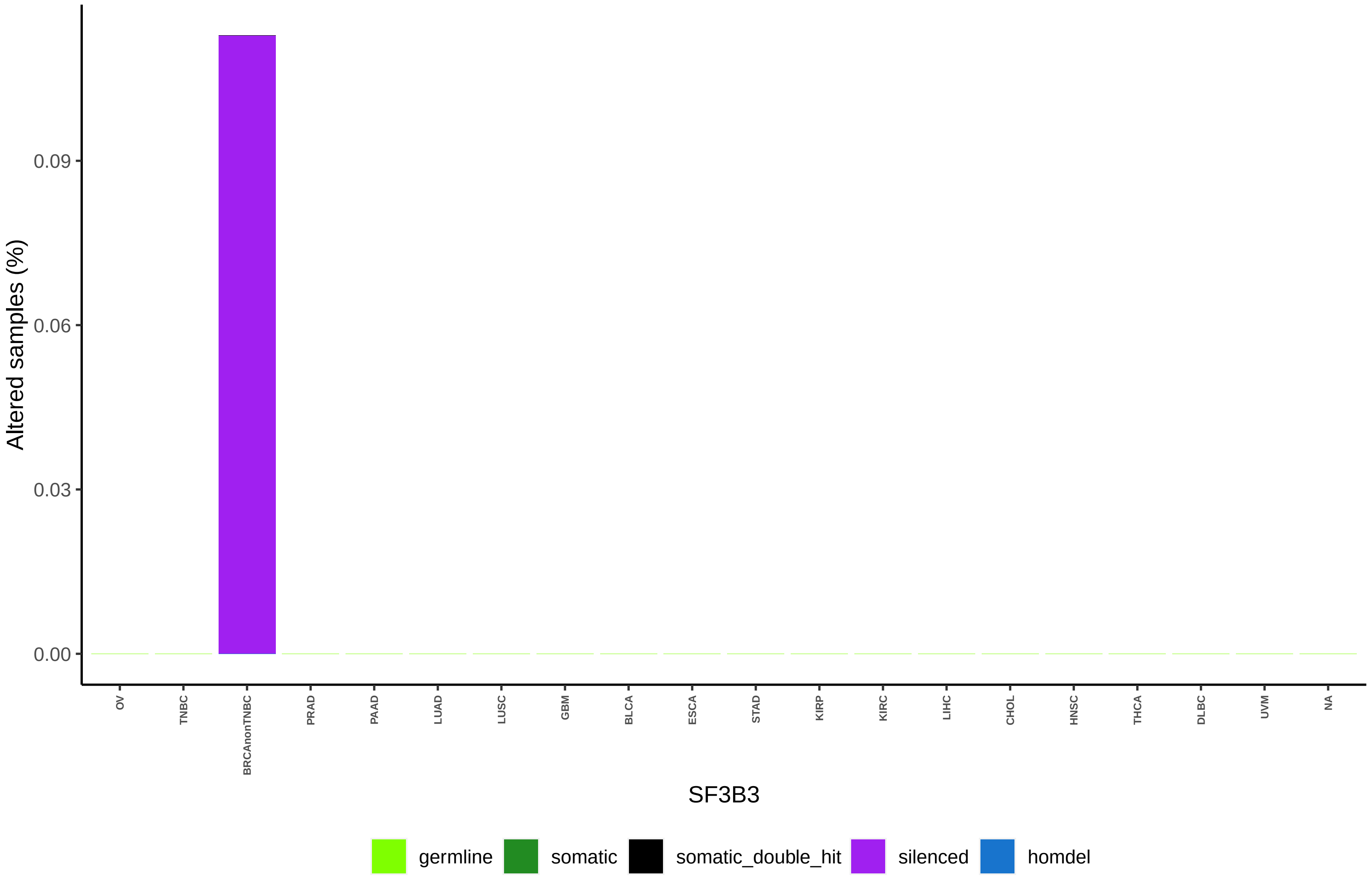
Altered samples (%)
0.00
0.03
0.06
0.09
OV
TNBC
BRCAnonTNBC
PRAD
PAAD
LUAD
LUSC
GBM
BLCA
ESCA
STAD
KIRP
KIRC
LIHC
CHOL
HNSC
THCA
DLBC
UVM
NA
SF3B3
germline
somatic
somatic_double_hit
silenced
homdel

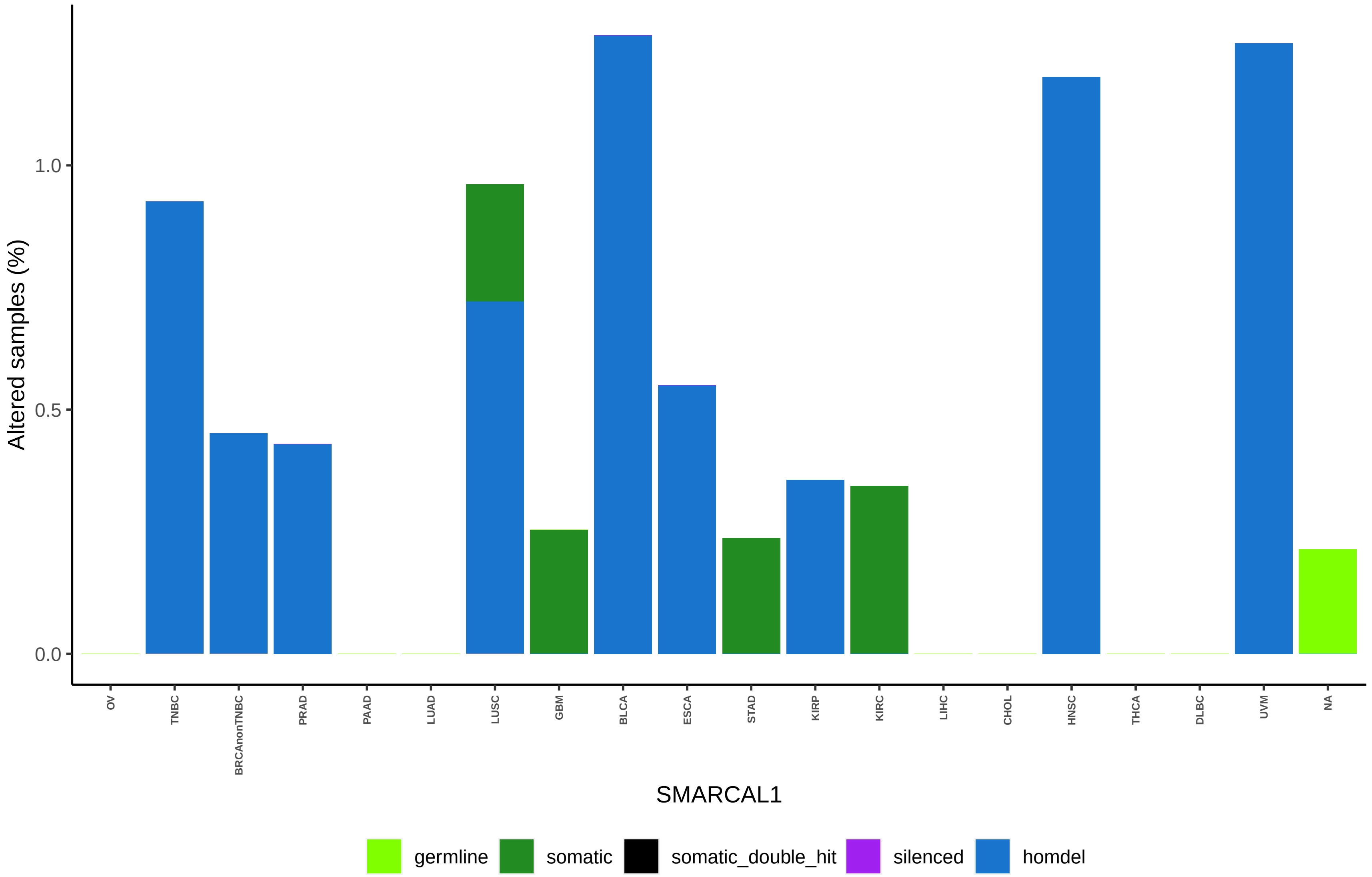
Altered samples (%)
0.0
0.5
1.0
OV
TNBC
BRCAnonTNBC
PRAD
PAAD
LUAD
LUSC
GBM
BLCA
ESCA
STAD
KIRP
KIRC
LIHC
CHOL
HNSC
THCA
DLBC
UVM
NA
SMARCAL1
germline
somatic
somatic_double_hit
silenced
homdel

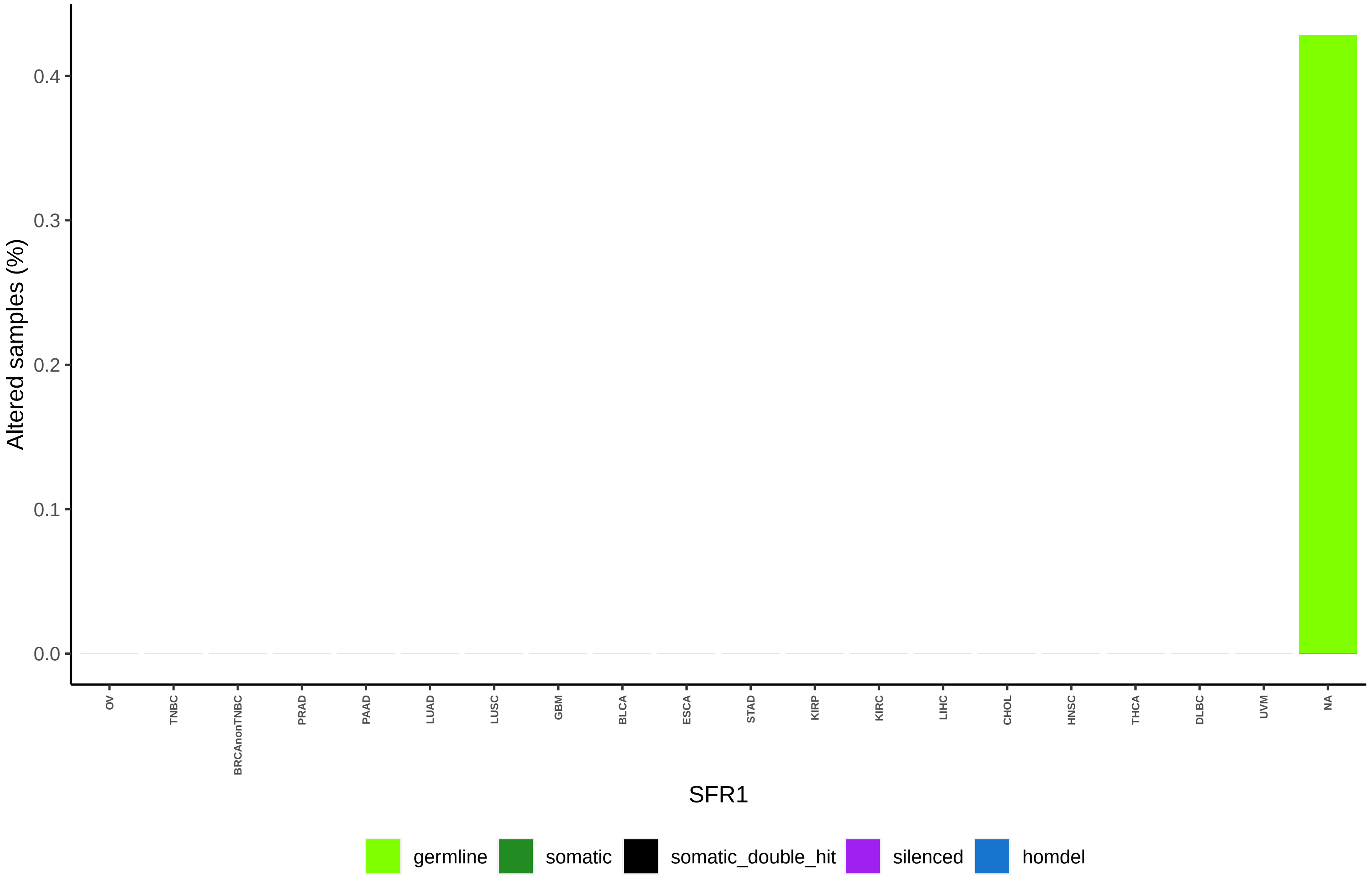
Altered samples (%)
0.0
0.1
0.2
0.3
0.4
OV
TNBC
BRCAnonTNBC
PRAD
PAAD
LUAD
LUSC
GBM
BLCA
ESCA
STAD
KIRP
KIRC
LIHC
CHOL
HNSC
THCA
DLBC
UVM
NA
SFR1
germline
somatic
somatic_double_hit
silenced
homdel

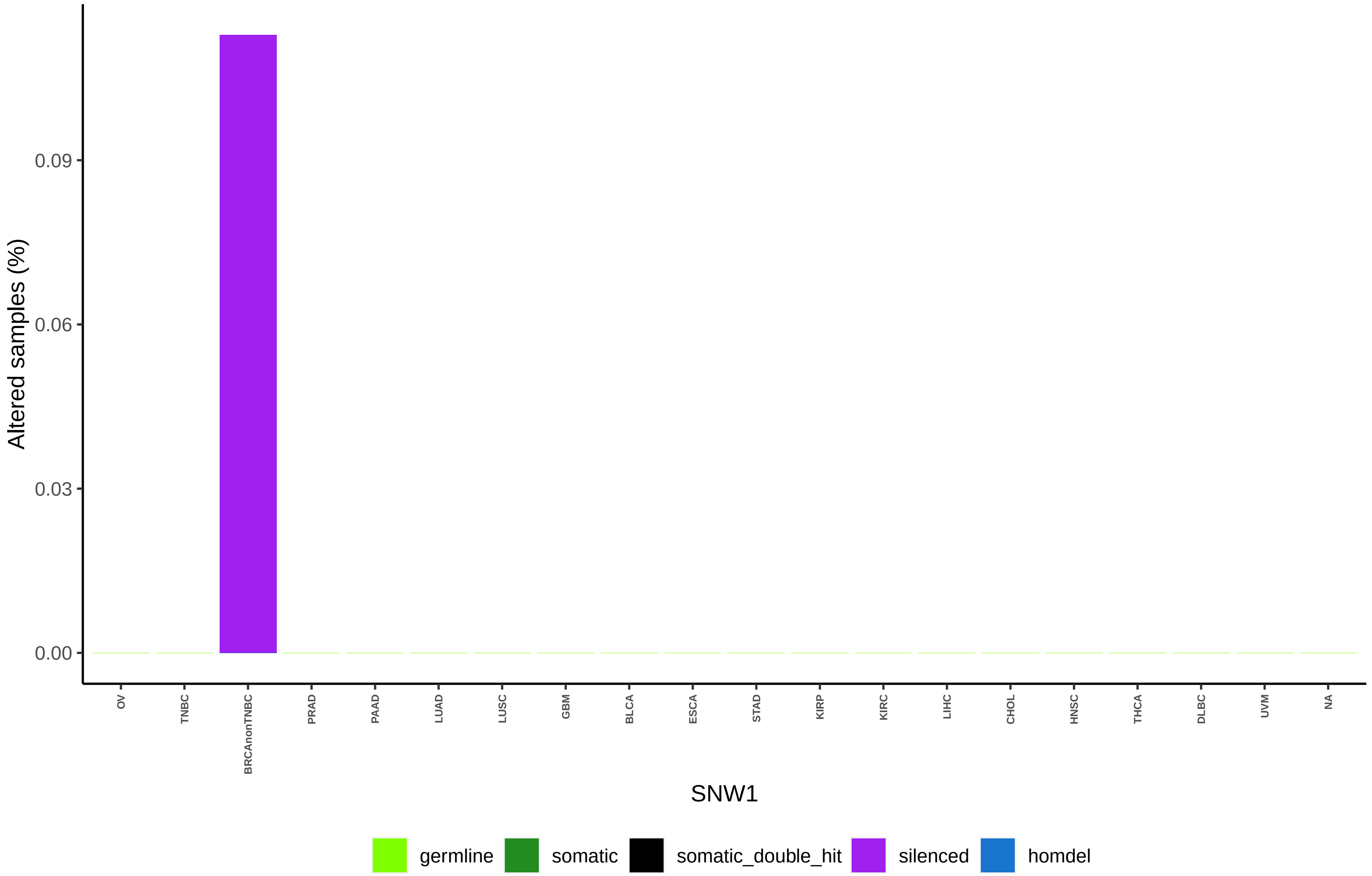
Altered samples (%)
0.00
0.03
0.06
0.09
OV
TNBC
BRCAnonTNBC
PRAD
PAAD
LUAD
LUSC
GBM
BLCA
ESCA
STAD
KIRP
KIRC
LIHC
CHOL
HNSC
THCA
DLBC
UVM
NA
SNW1
germline
somatic
somatic_double_hit
silenced
homdel

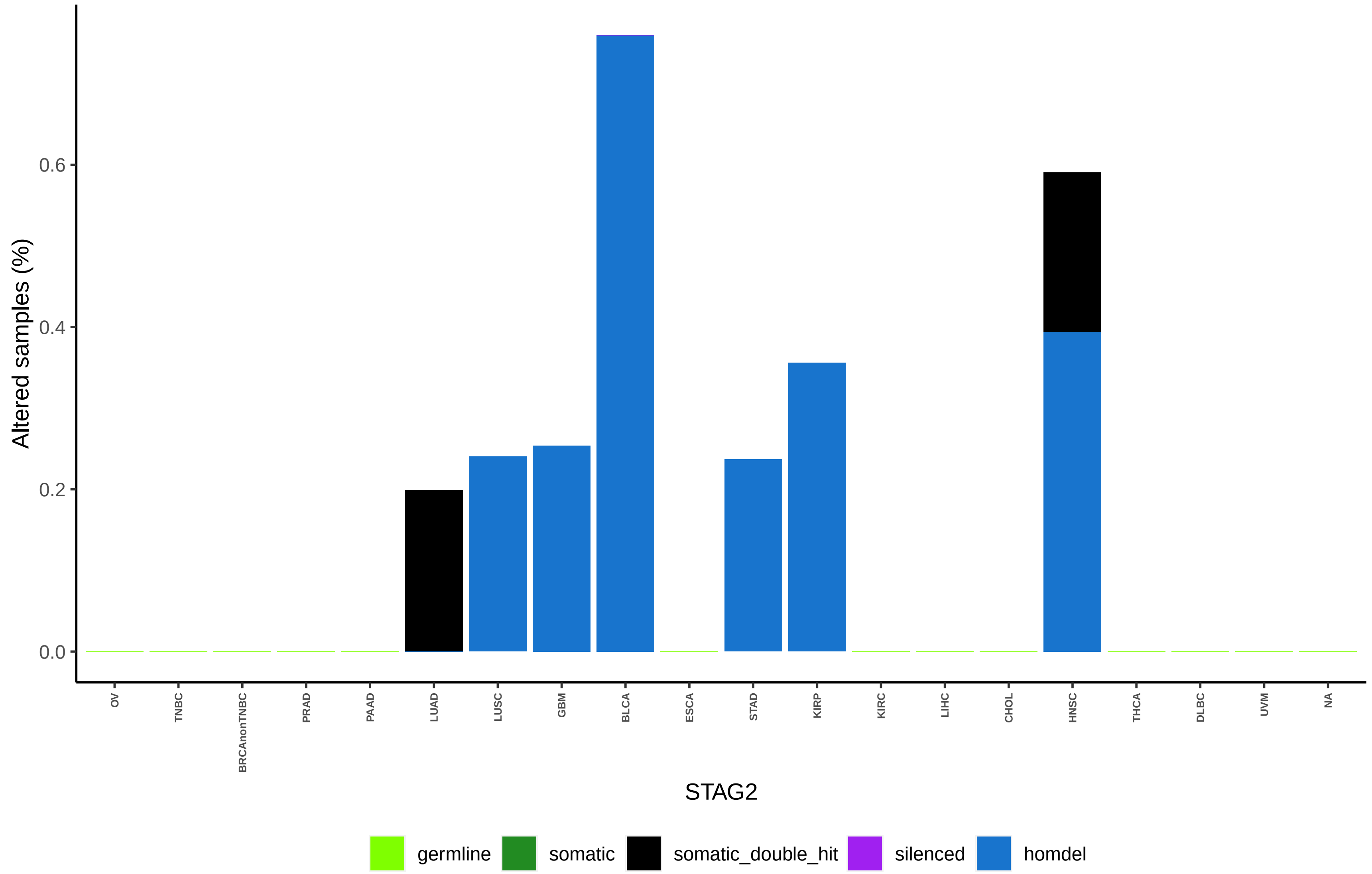
Altered samples (%)
0.0
0.2
0.4
0.6
OV
TNBC
BRCAnonTNBC
PRAD
PAAD
LUAD
LUSC
GBM
BLCA
ESCA
STAD
KIRP
KIRC
LIHC
CHOL
HNSC
THCA
DLBC
UVM
NA
STAG2
germline
somatic
somatic_double_hit
silenced
homdel

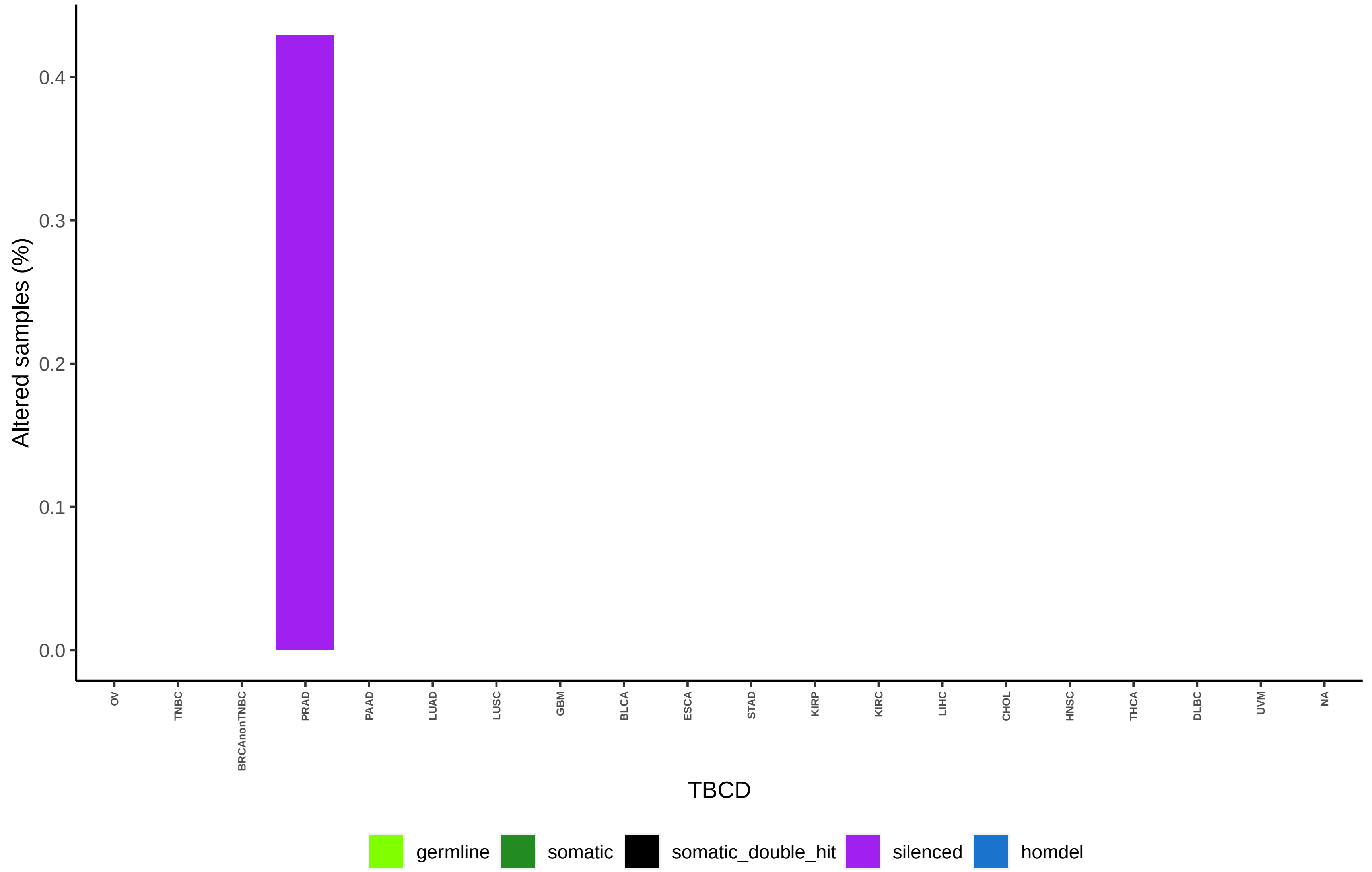
Altered samples (%)
0.0
0.1
0.2
0.3
0.4
OV
TNBC
BRCAnonTNBC
PRAD
PAAD
LUAD
LUSC
GBM
BLCA
ESCA
STAD
KIRP
KIRC
LIHC
CHOL
HNSC
THCA
DLBC
UVM
NA
TBCD
germline
somatic
somatic_double_hit
silenced
homdel

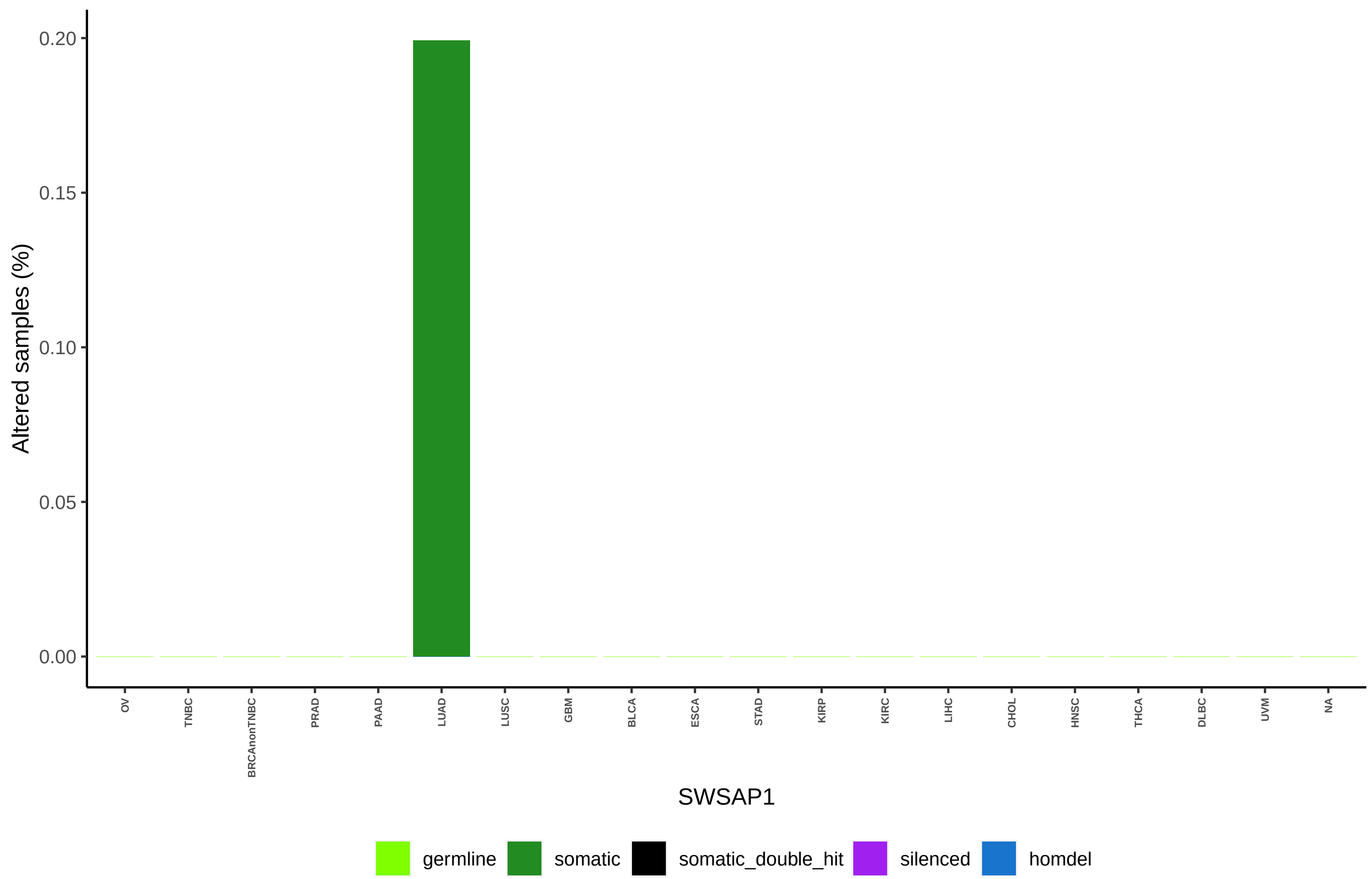
Altered samples (%)
0.00
0.05
0.10
0.15
0.20
OV
TNBC
BRCAnonTNBC
PRAD
PAAD
LUAD
LUSC
GBM
BLCA
ESCA
STAD
KIRP
KIRC
LIHC
CHOL
HNSC
THCA
DLBC
UVM
NA
SWSAP1
germline
somatic
somatic_double_hit
silenced
homdel

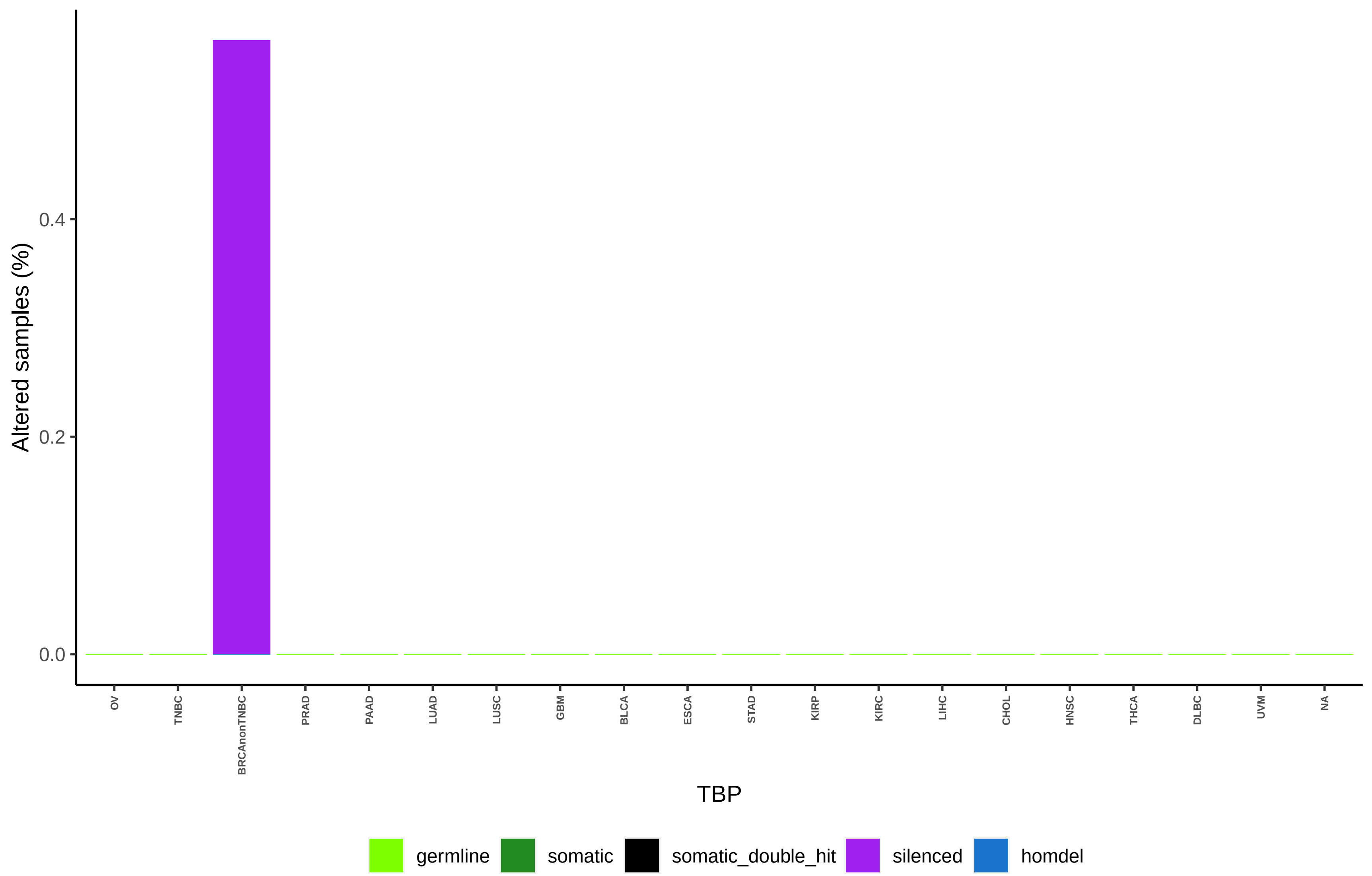
Altered samples (%)
0.0
0.2
0.4
OV
TNBC
BRCAnonTNBC
PRAD
PAAD
LUAD
LUSC
GBM
BLCA
ESCA
STAD
KIRP
KIRC
LIHC
CHOL
HNSC
THCA
DLBC
UVM
NA
TBP
germline
somatic
somatic_double_hit
silenced
homdel

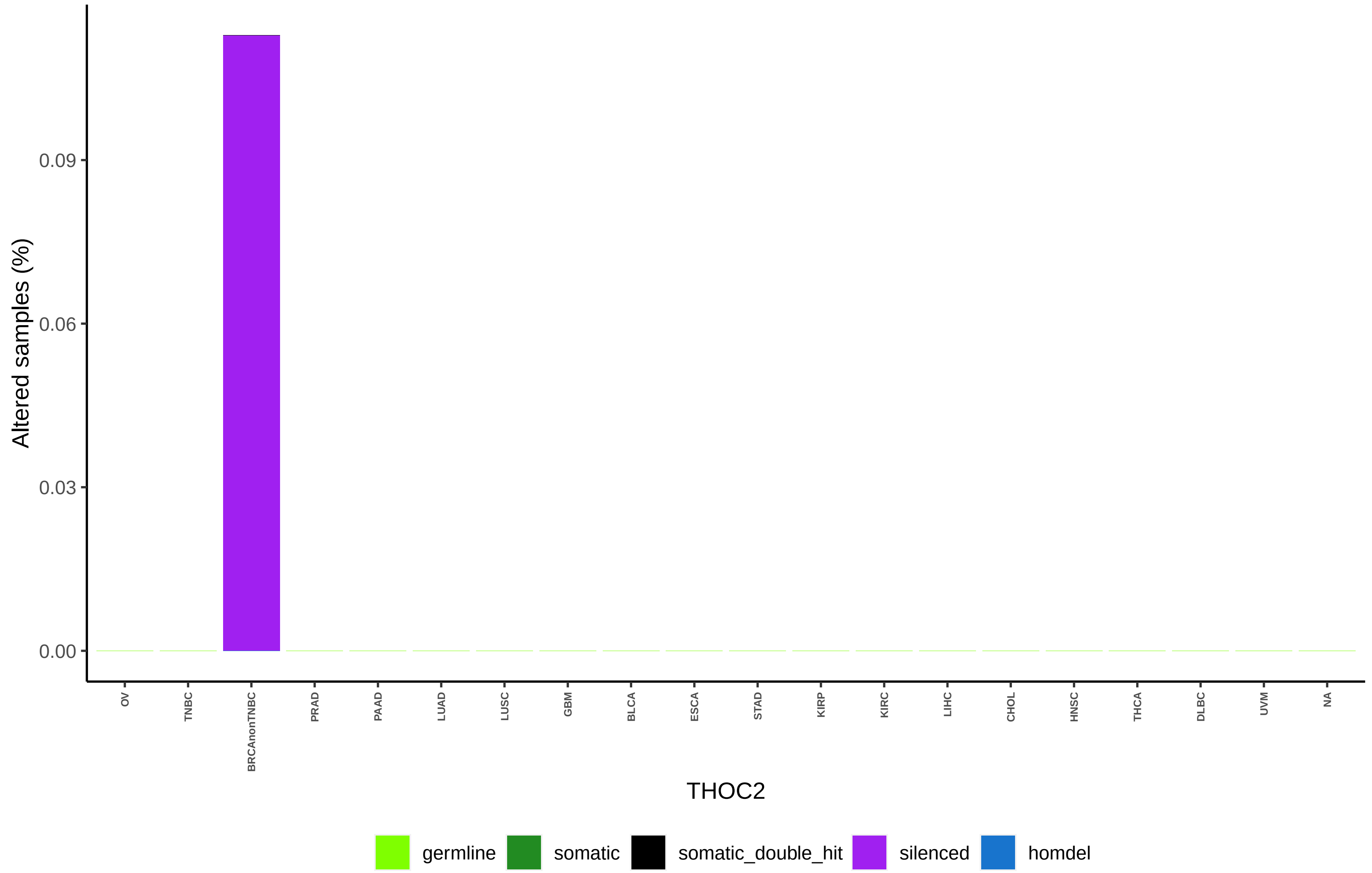
Altered samples (%)
0.00
0.03
0.06
0.09
OV
TNBC
BRCAnonTNBC
PRAD
PAAD
LUAD
LUSC
GBM
BLCA
ESCA
STAD
KIRP
KIRC
LIHC
CHOL
HNSC
THCA
DLBC
UVM
NA
THOC2
germline
somatic
somatic_double_hit
silenced
homdel

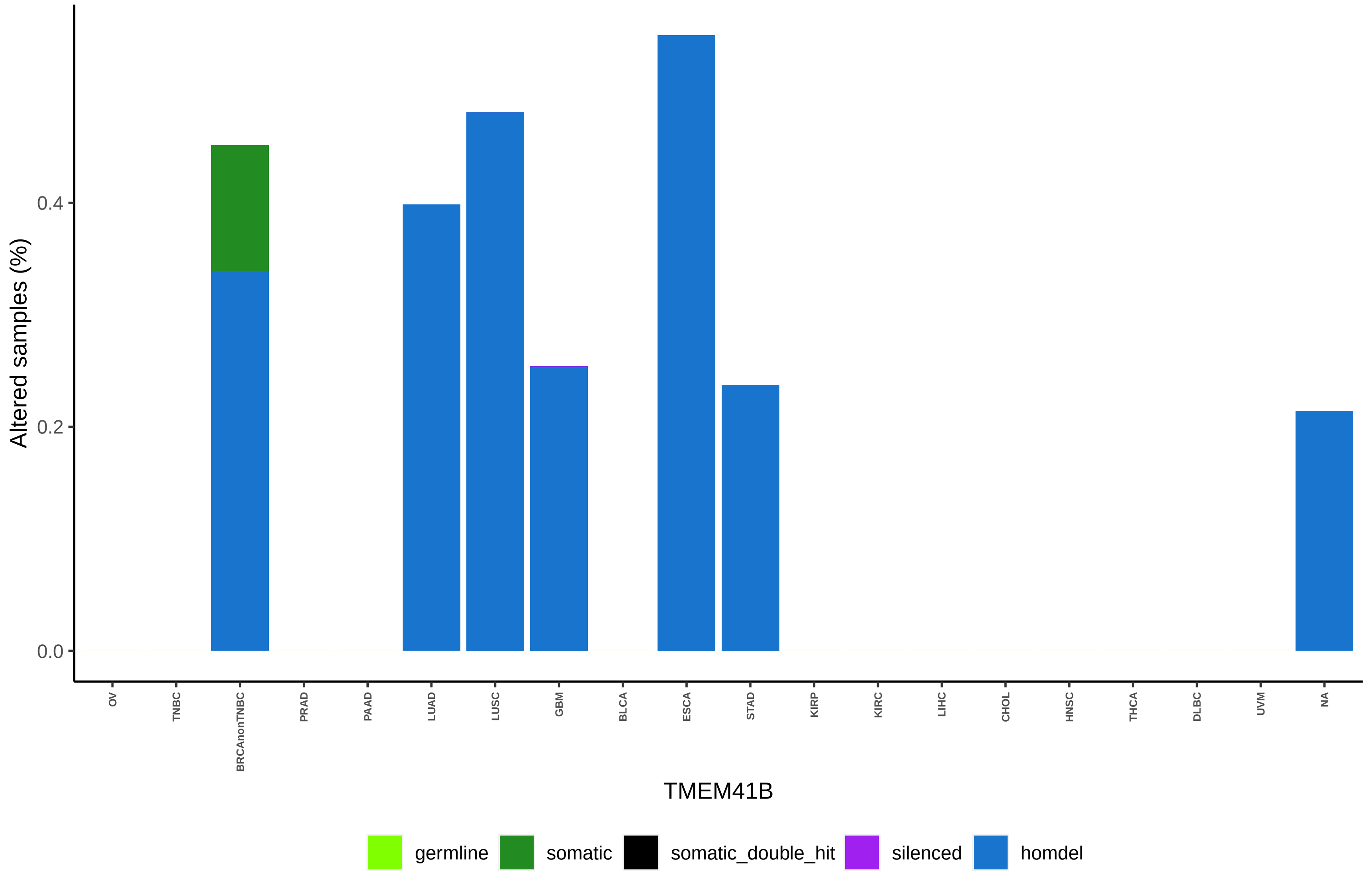
Altered samples (%)
0.0
0.2
0.4
OV
TNBC
BRCAnonTNBC
PRAD
PAAD
LUAD
LUSC
GBM
BLCA
ESCA
STAD
KIRP
KIRC
LIHC
CHOL
HNSC
THCA
DLBC
UVM
NA
TMEM41B
germline
somatic
somatic_double_hit
silenced
homdel

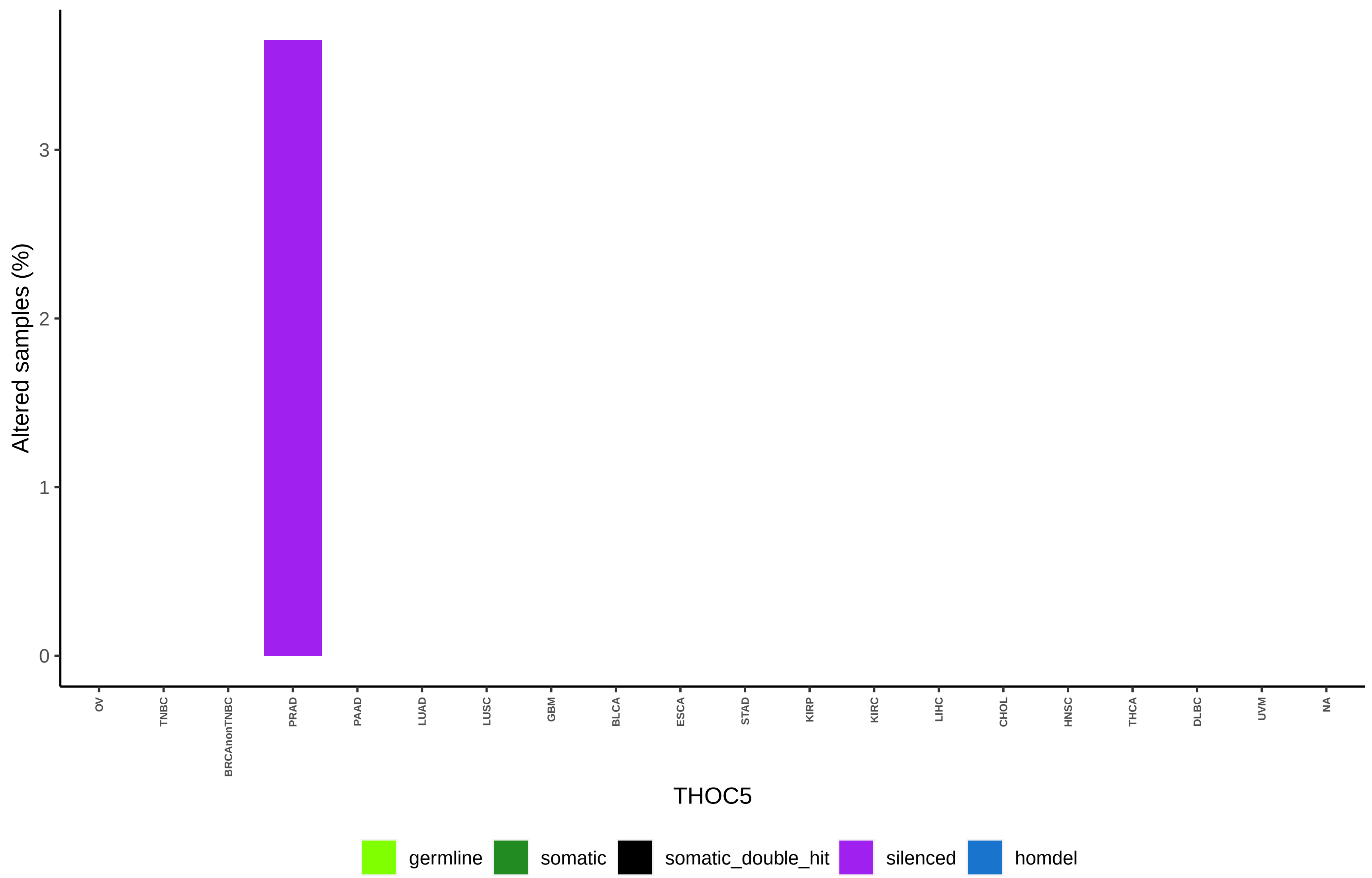
Altered samples (%)
0
1
2
3
OV
TNBC
BRCAnonTNBC
PRAD
PAAD
LUAD
LUSC
GBM
BLCA
ESCA
STAD
KIRP
KIRC
LIHC
CHOL
HNSC
THCA
DLBC
UVM
NA
THOC5
germline
somatic
somatic_double_hit
silenced
homdel

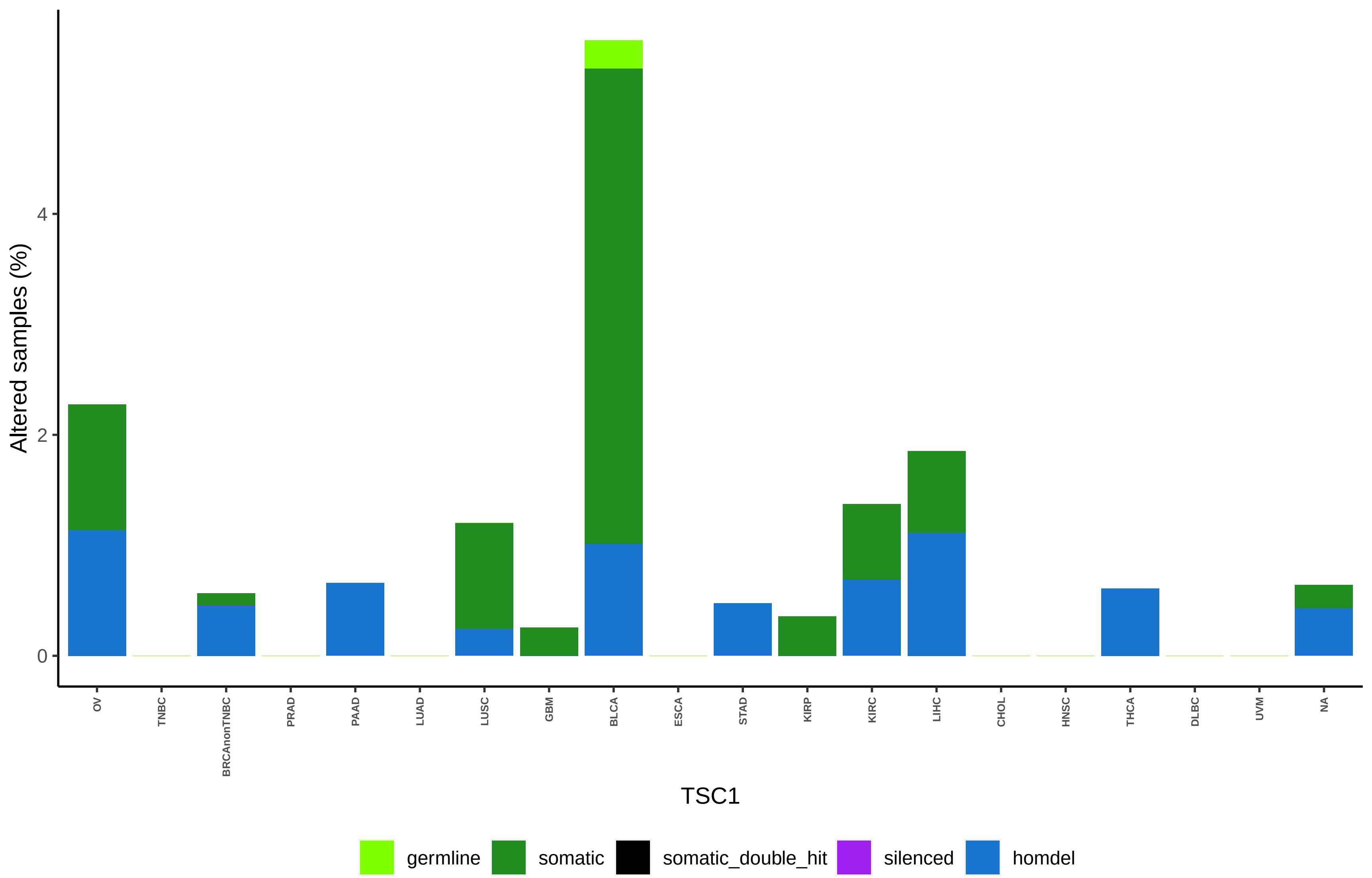
Altered samples (%)
0
2
4
OV
TNBC
BRCAnonTNBC
PRAD
PAAD
LUAD
LUSC
GBM
BLCA
ESCA
STAD
KIRP
KIRC
LIHC
CHOL
HNSC
THCA
DLBC
UVM
NA
TSC1
germline
somatic
somatic_double_hit
silenced
homdel

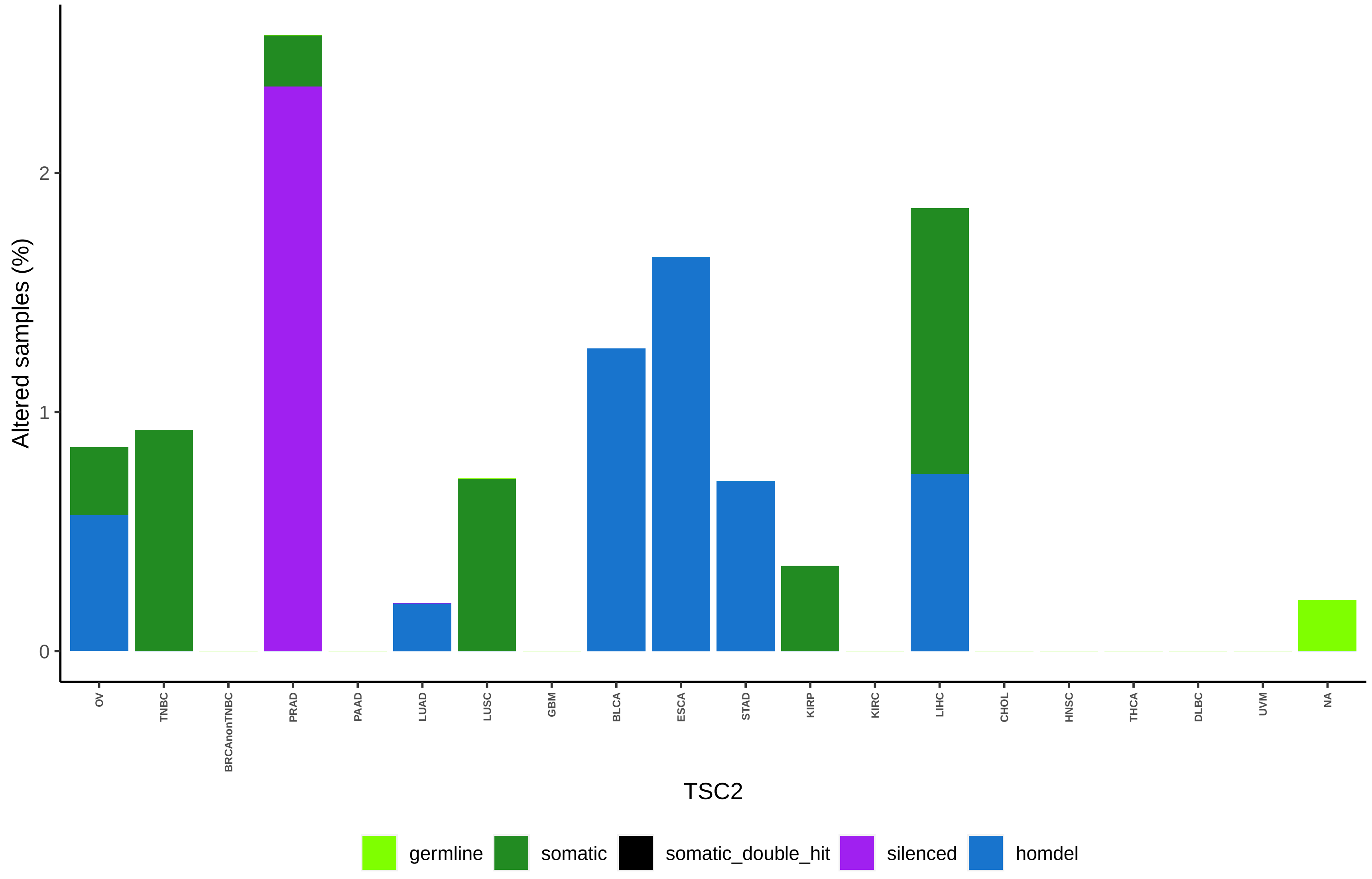
Altered samples (%)
0
1
2
OV
TNBC
BRCAnonTNBC
PRAD
PAAD
LUAD
LUSC
GBM
BLCA
ESCA
STAD
KIRP
KIRC
LIHC
CHOL
HNSC
THCA
DLBC
UVM
NA
TSC2
germline
somatic
somatic_double_hit
silenced
homdel

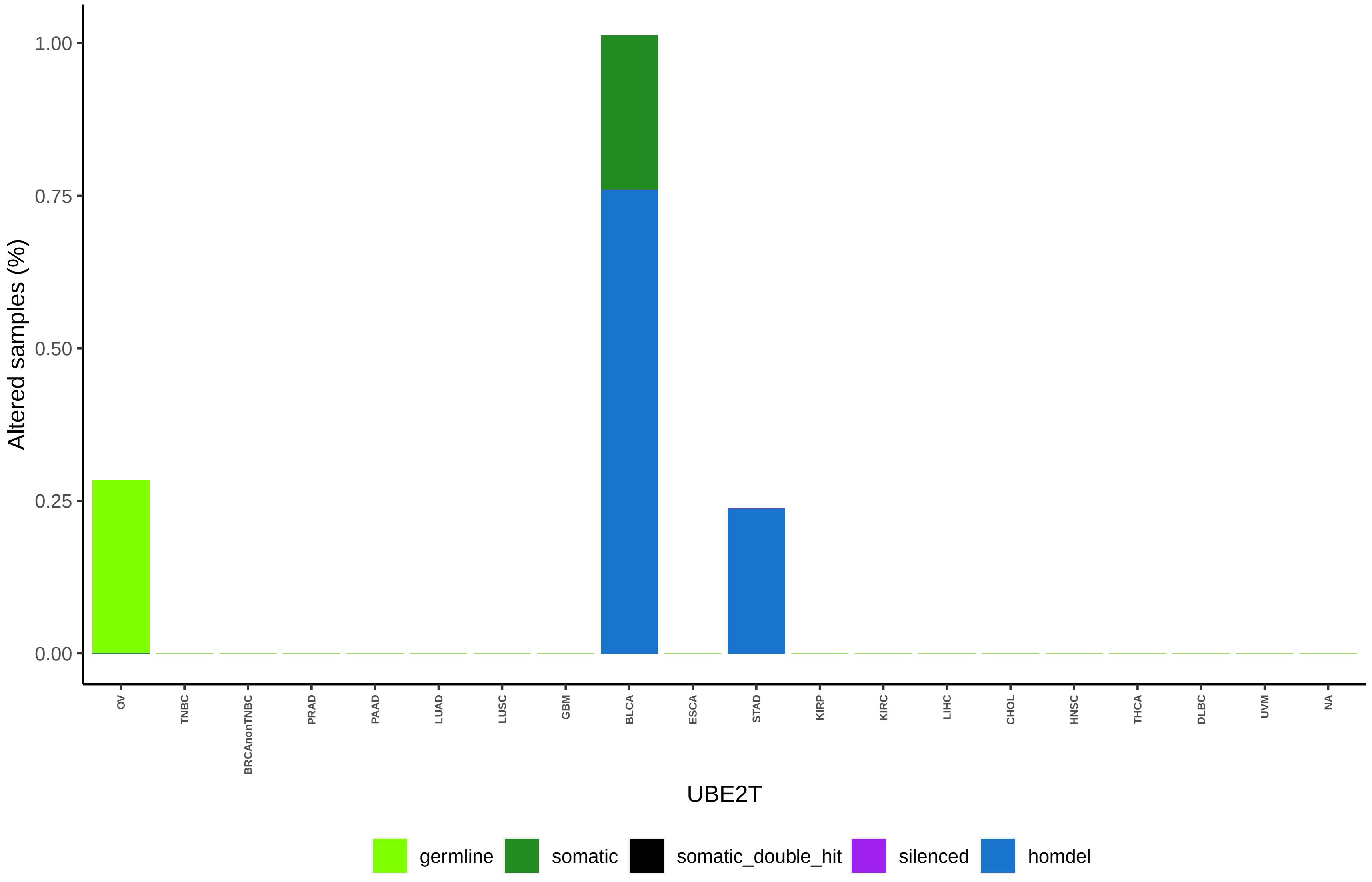
Altered samples (%)
0.00
0.25
0.50
0.75
1.00
OV
TNBC
BRCAnonTNBC
PRAD
PAAD
LUAD
LUSC
GBM
BLCA
ESCA
STAD
KIRP
KIRC
LIHC
CHOL
HNSC
THCA
DLBC
UVM
NA
UBE2T
germline
somatic
somatic_double_hit
silenced
homdel

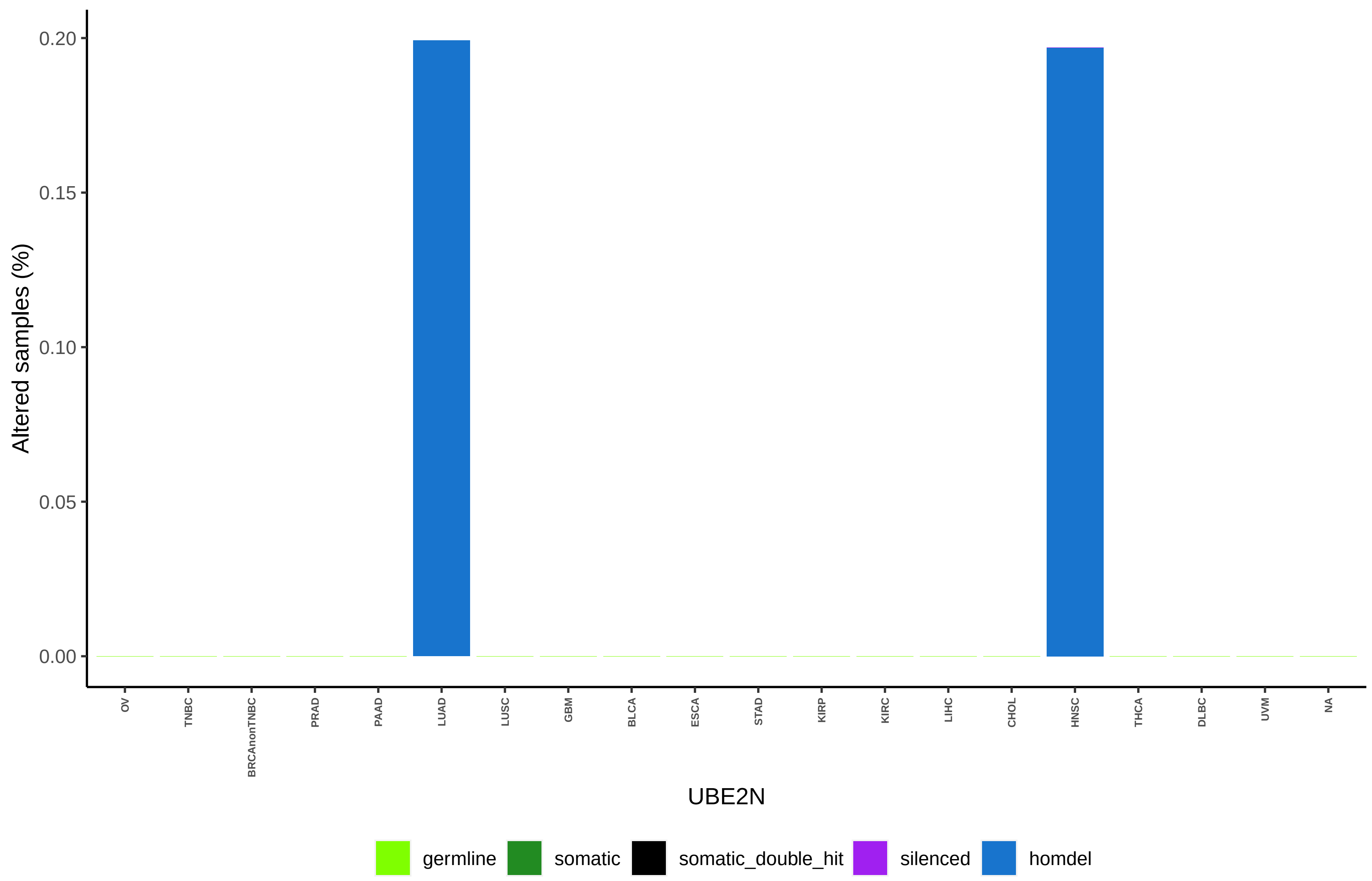
Altered samples (%)
0.00
0.05
0.10
0.15
0.20
OV
TNBC
BRCAnonTNBC
PRAD
PAAD
LUAD
LUSC
GBM
BLCA
ESCA
STAD
KIRP
KIRC
LIHC
CHOL
HNSC
THCA
DLBC
UVM
NA
UBE2N
germline
somatic
somatic_double_hit
silenced
homdel

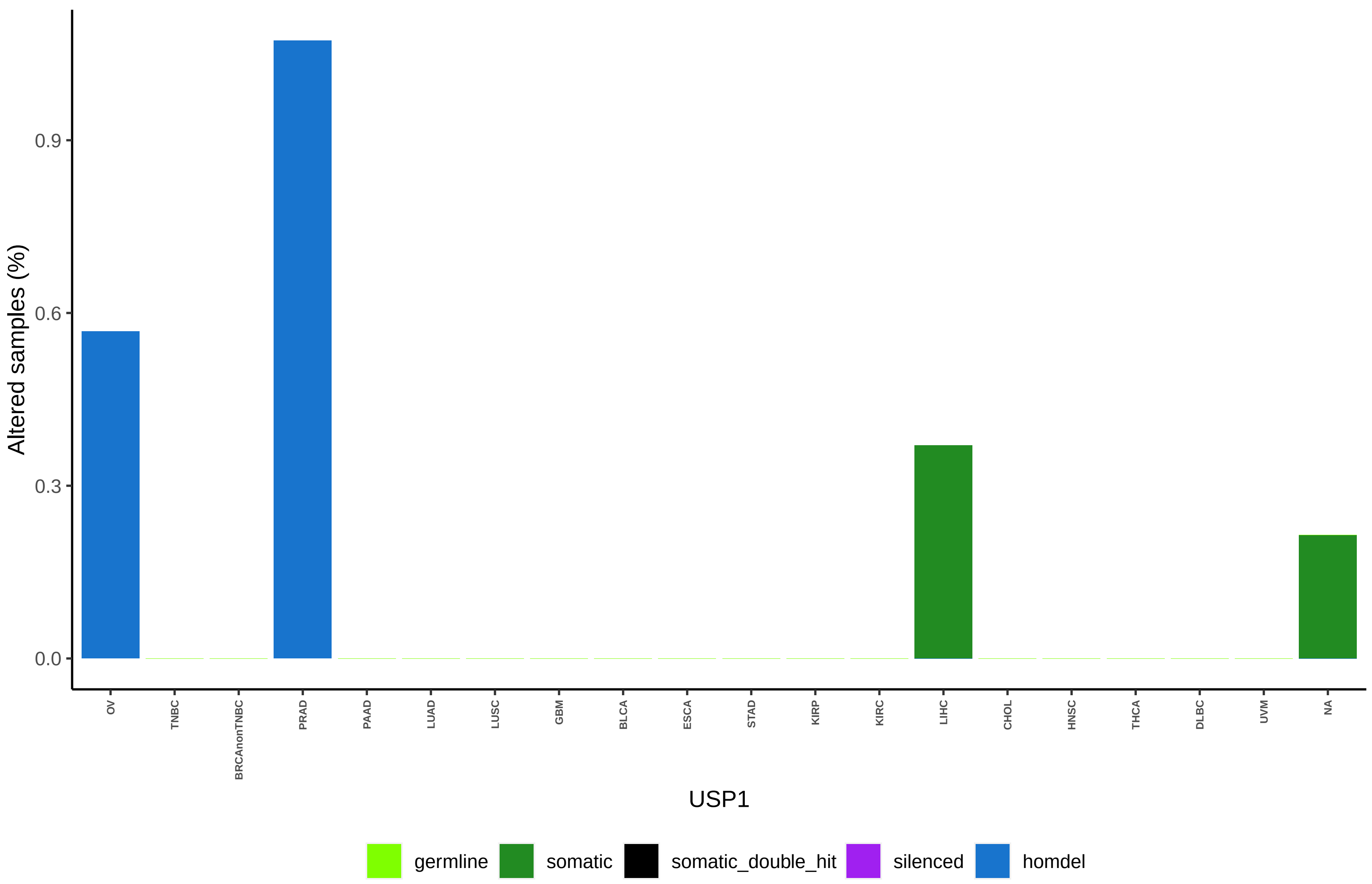
Altered samples (%)
0.0
0.3
0.6
0.9
OV
TNBC
BRCAnonTNBC
PRAD
PAAD
LUAD
LUSC
GBM
BLCA
ESCA
STAD
KIRP
KIRC
LIHC
CHOL
HNSC
THCA
DLBC
UVM
NA
USP1
germline
somatic
somatic_double_hit
silenced
homdel

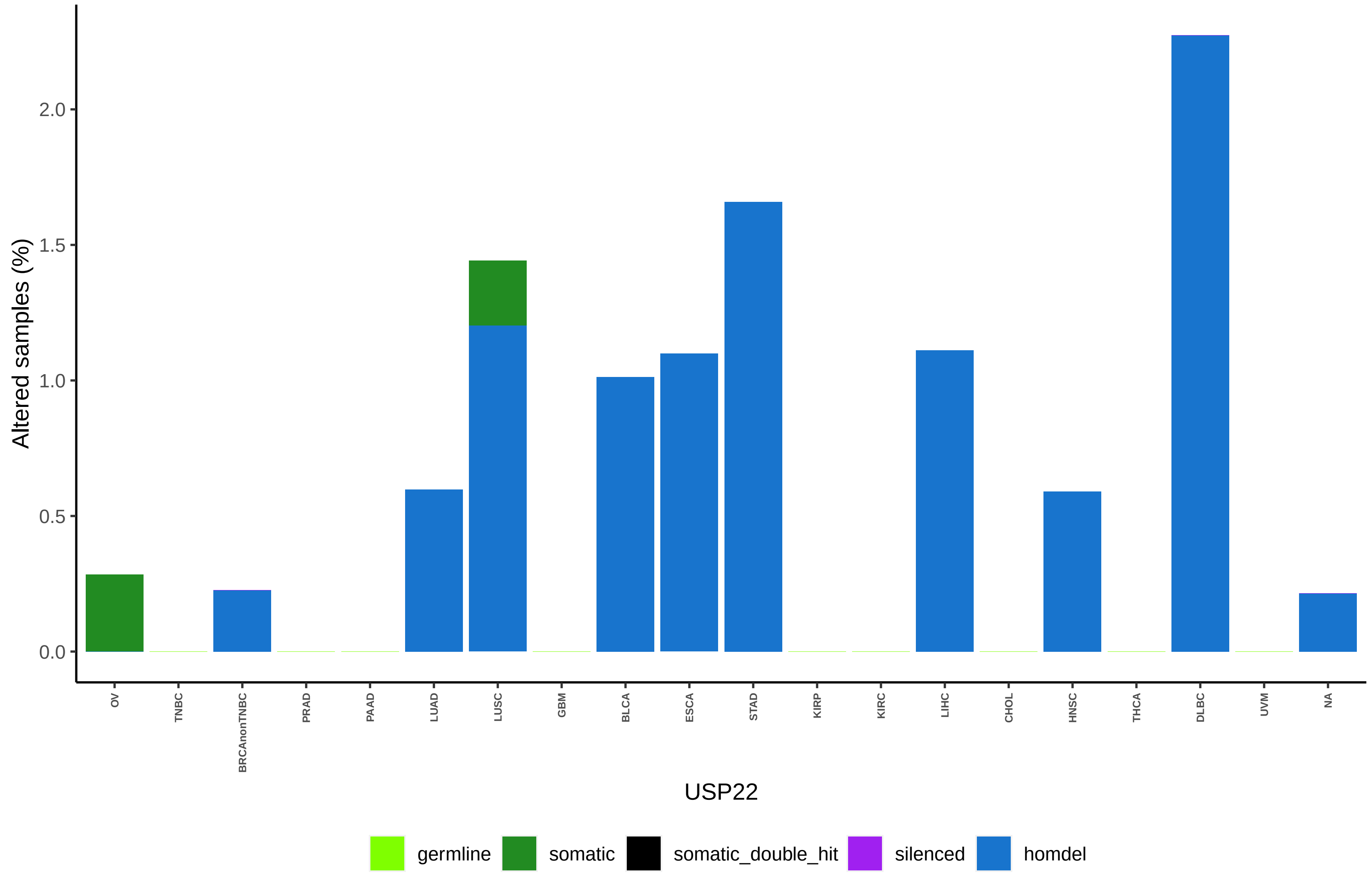
Altered samples (%)
USP22
germline
somatic
somatic_double_hit
silenced
homdel

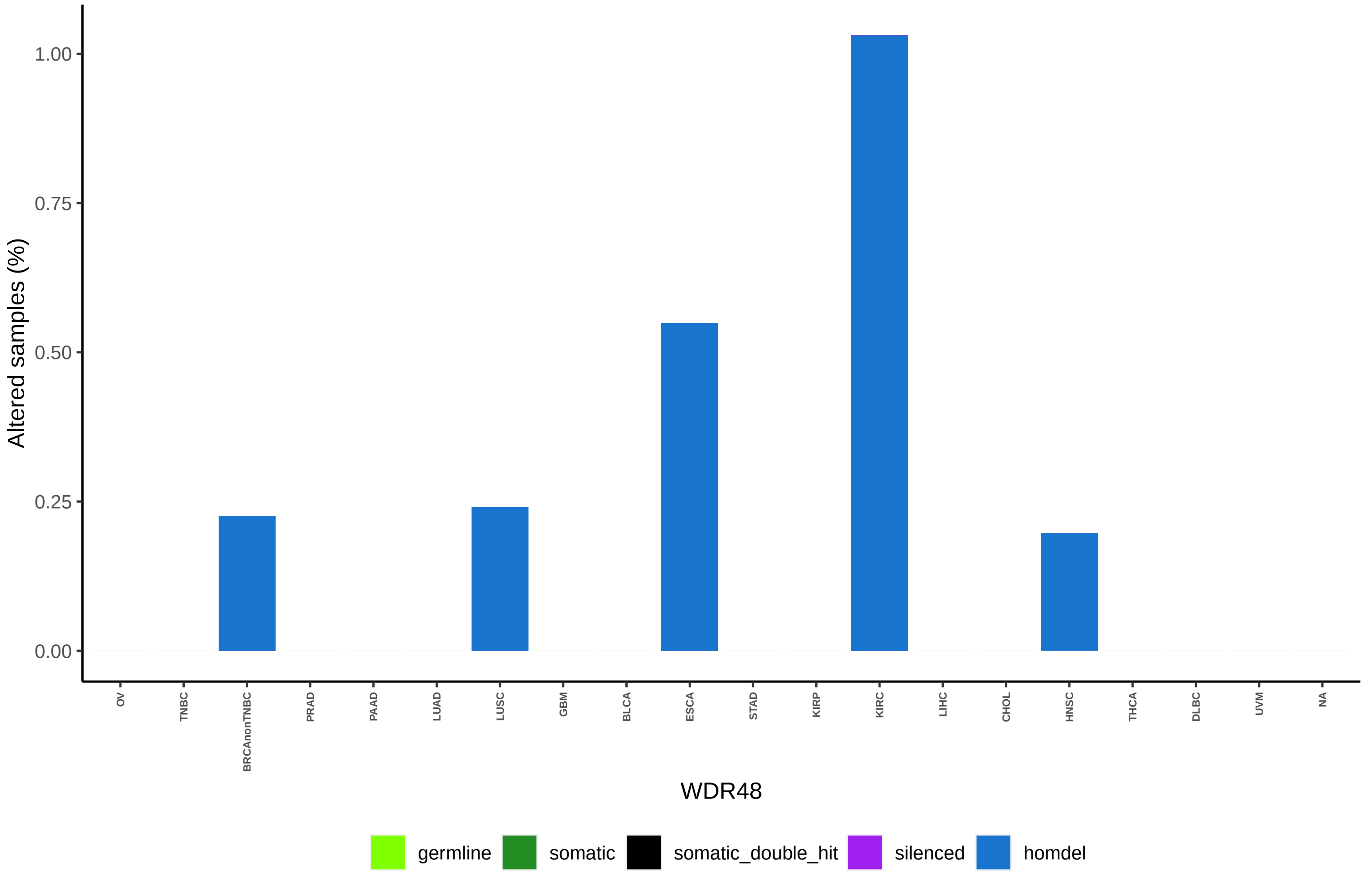
Altered samples (%)
WDR48
germline
somatic
somatic_double_hit
silenced
homdel

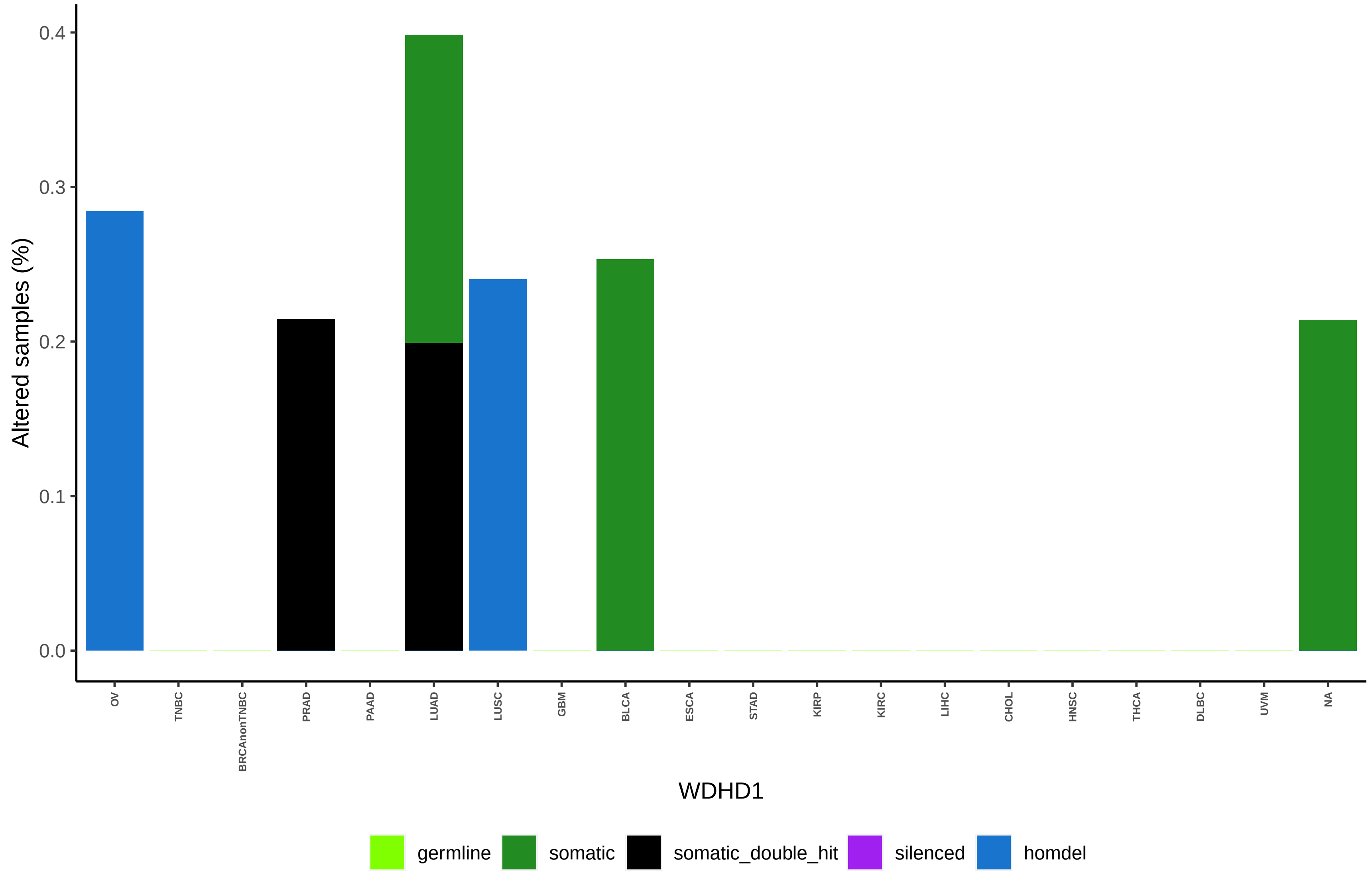
Altered samples (%)
WDHD1
germline
somatic
somatic_double_hit
silenced
homdel

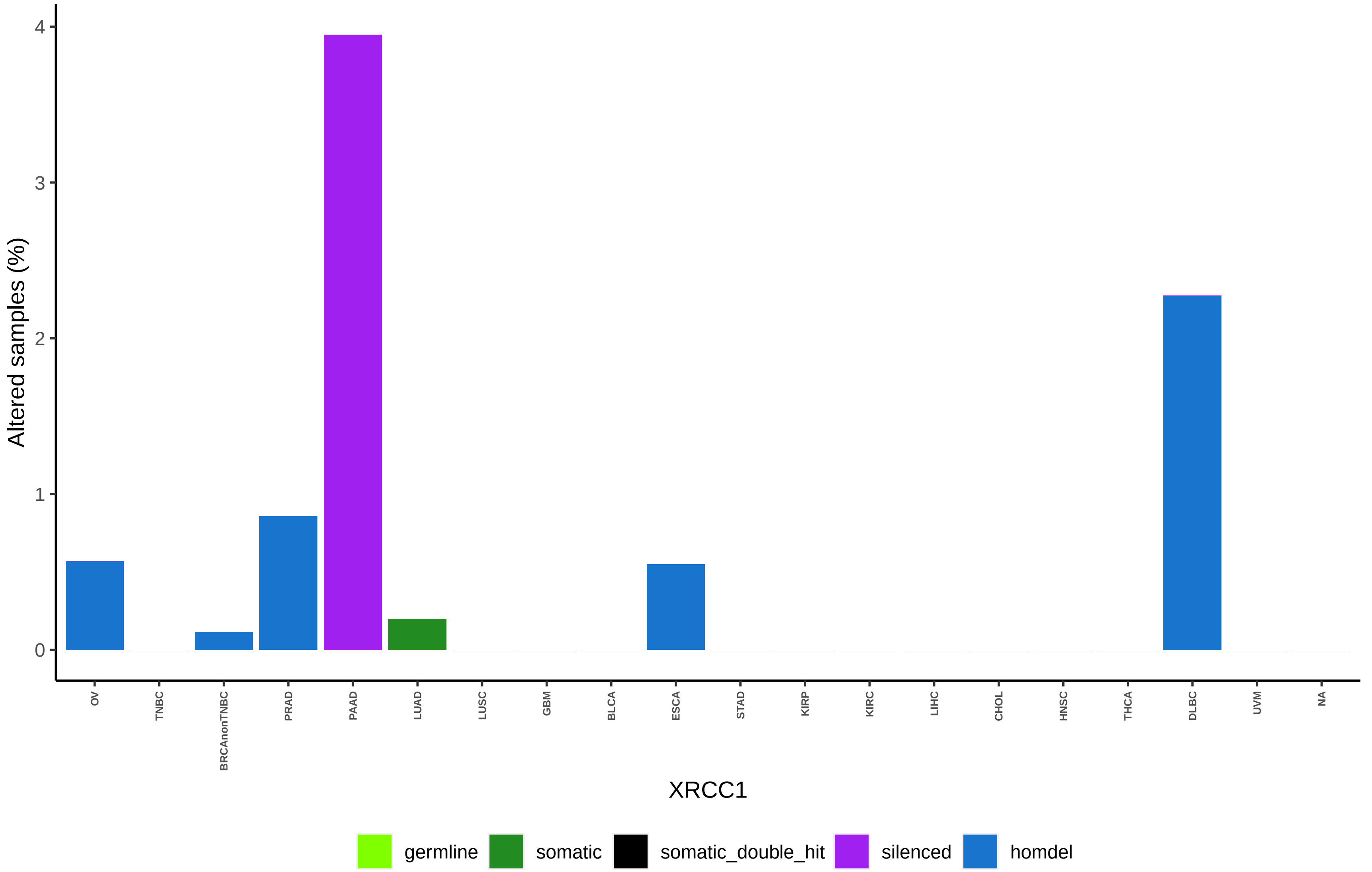
Altered samples (%)
XRCC1
germline
somatic
somatic_double_hit
silenced
homdel

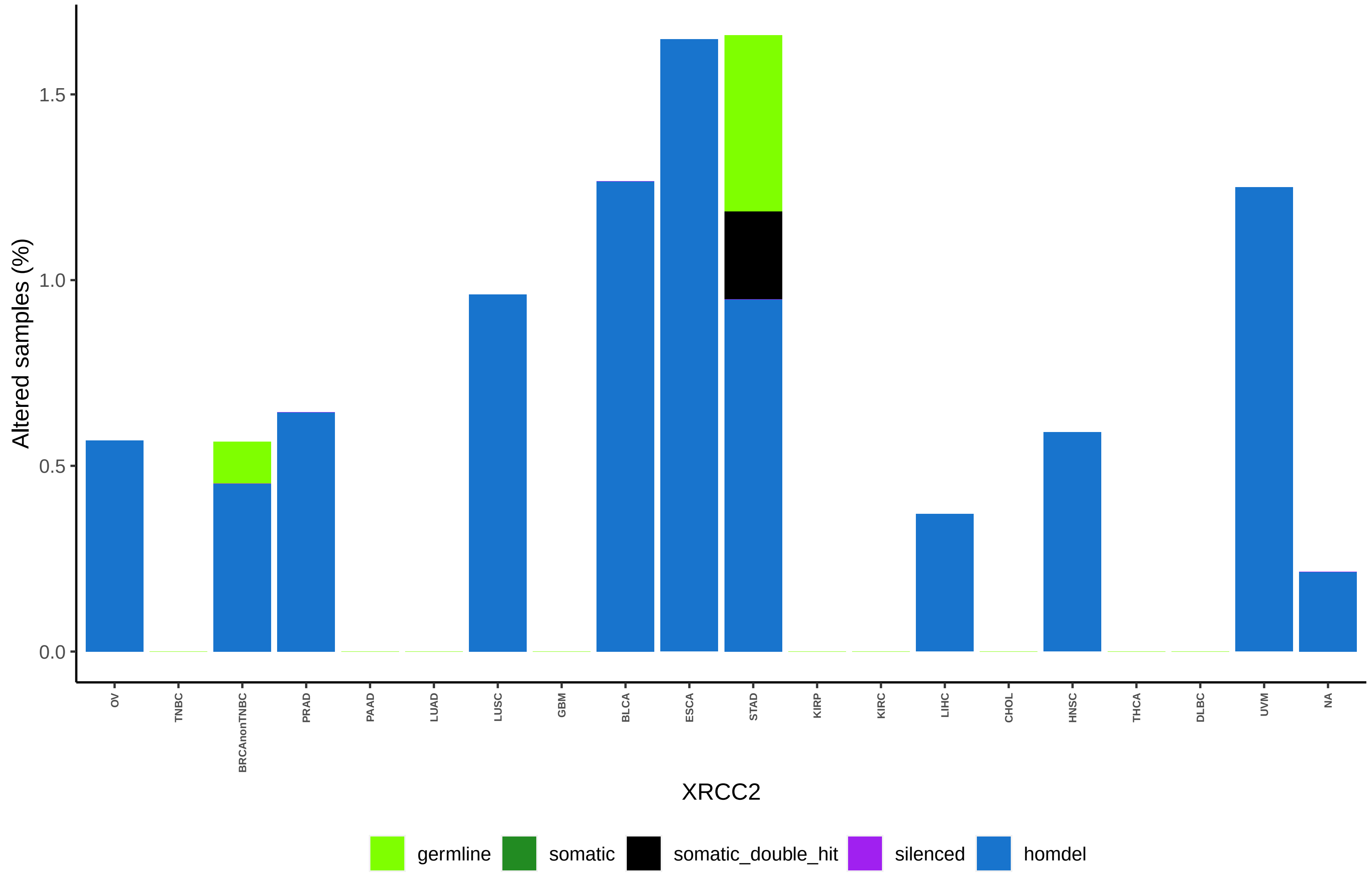
Altered samples (%)
0.0
0.5
1.0
1.5
OV TNBC BRCAnonTNBC PRAD PAAD LUAD LUSC GBM BLCA ESCA STAD KIRP KIRC LIHC CHOL HNSC THCA DLBC UVM NA
XRCC2
germline somatic somatic_double_hit silenced homdel

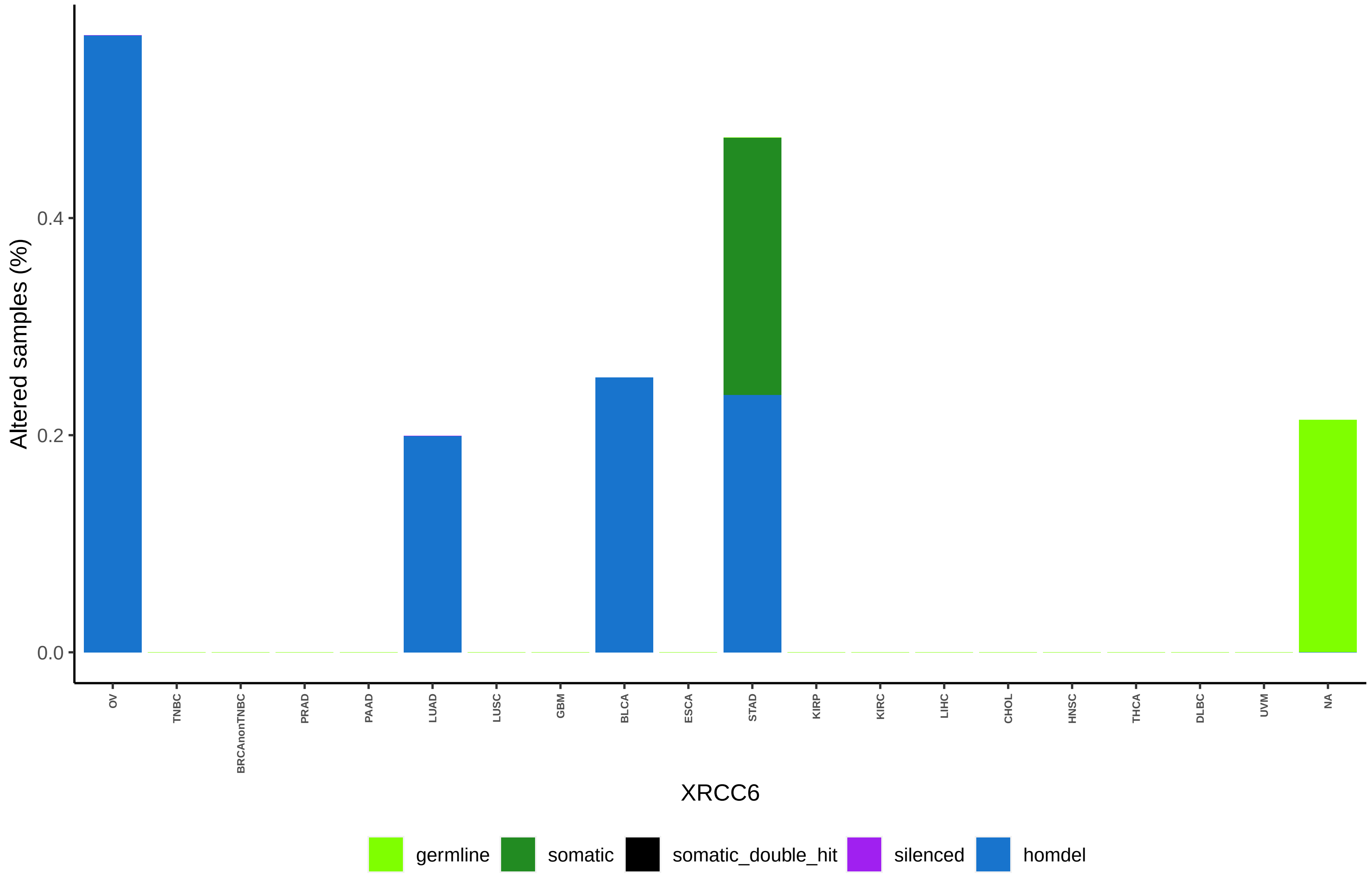
Altered samples (%)
0.0
0.2
0.4
OV TNBC BRCAnonTNBC PRAD PAAD LUAD LUSC GBM BLCA ESCA STAD KIRP KIRC LIHC CHOL HNSC THCA DLBC UVM NA
XRCC6
germline somatic somatic_double_hit silenced homdel

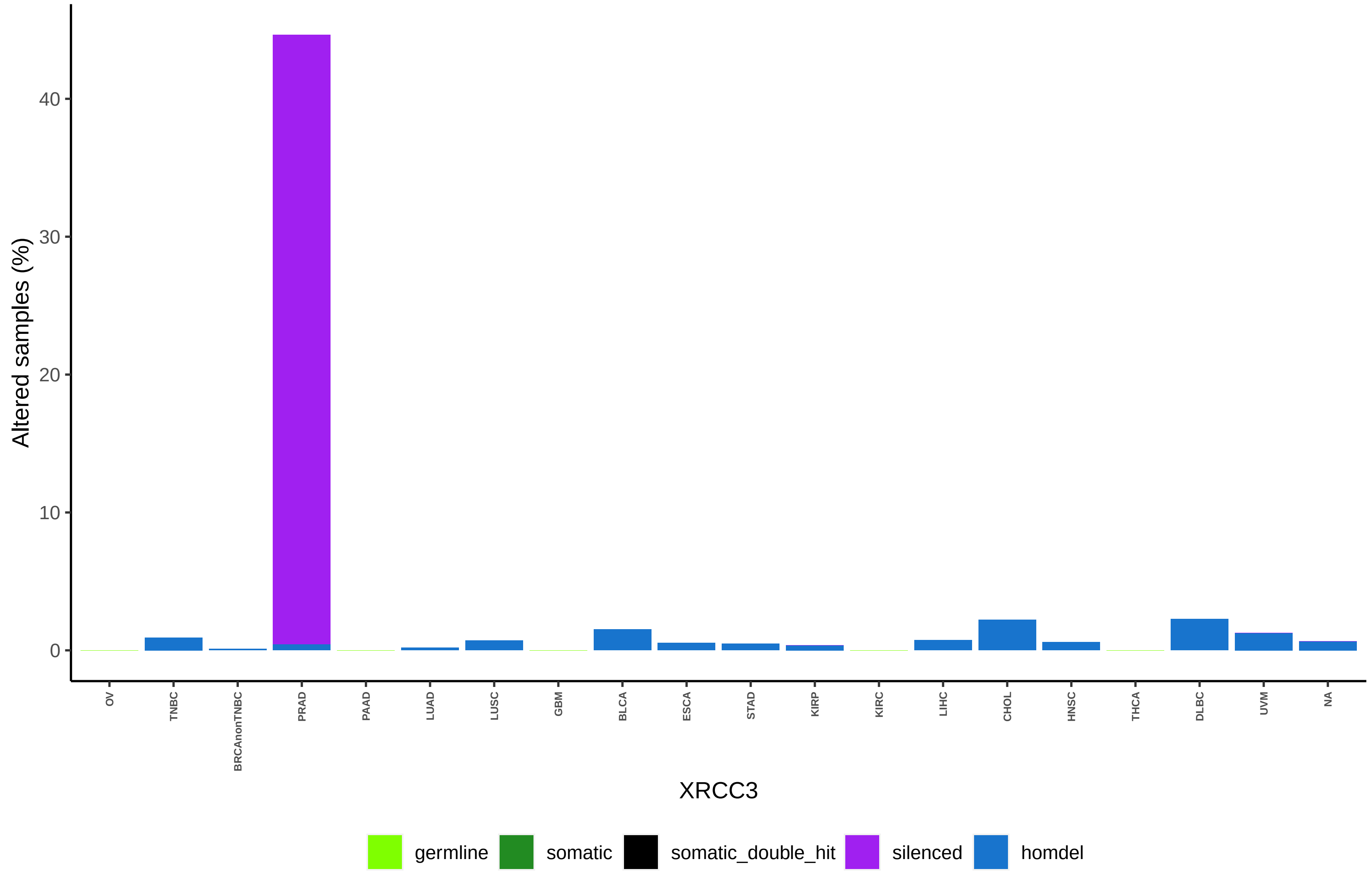
Altered samples (%)
0
10
20
30
40
OV TNBC BRCAnonTNBC PRAD PAAD LUAD LUSC GBM BLCA ESCA STAD KIRP KIRC LIHC CHOL HNSC THCA DLBC UVM NA
XRCC3
germline somatic somatic_double_hit silenced homdel

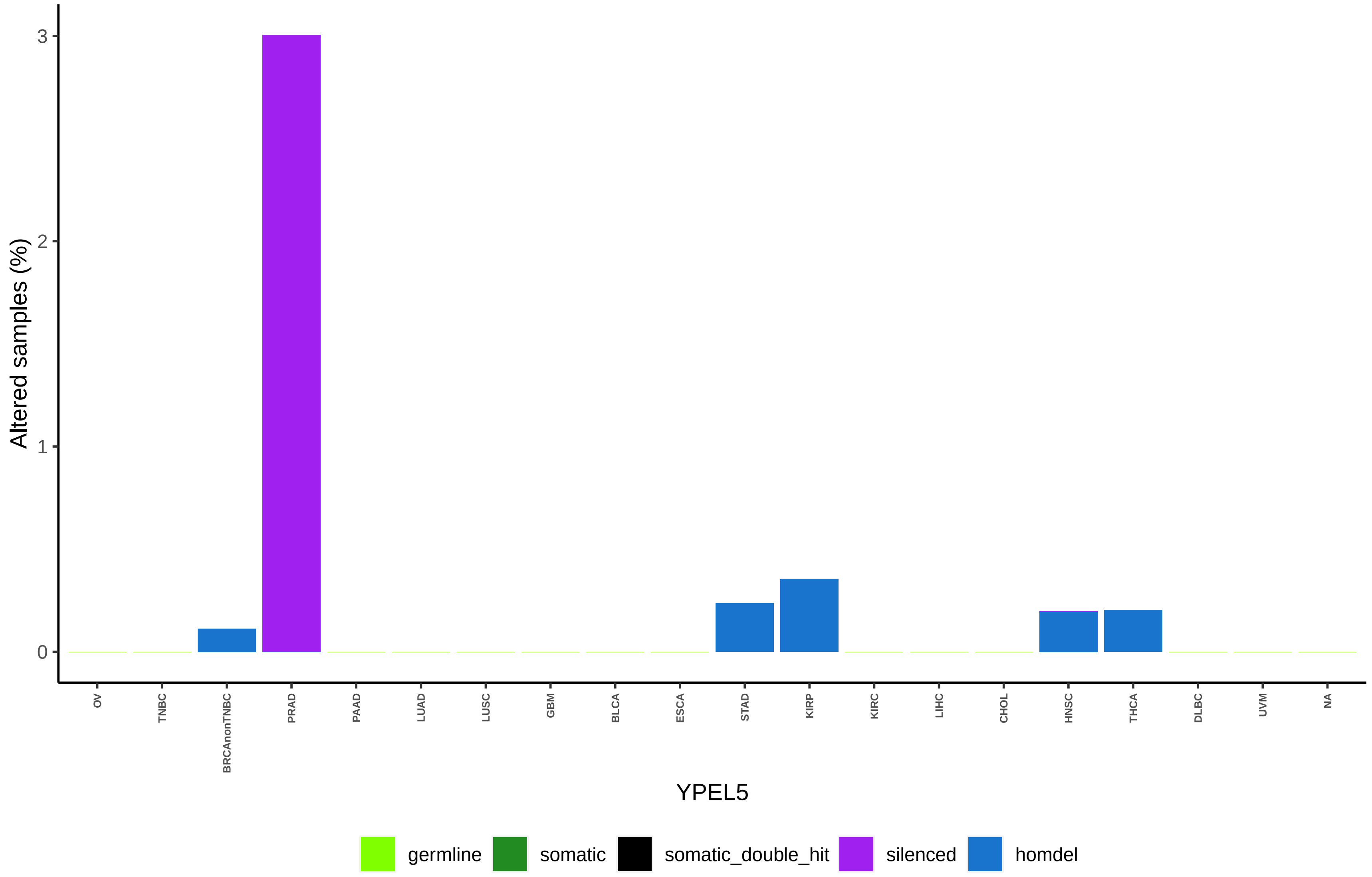
Altered samples (%)
0
1
2
3
OV TNBC BRCAnonTNBC PRAD PAAD LUAD LUSC GBM BLCA ESCA STAD KIRP KIRC LIHC CHOL HNSC THCA DLBC UVM NA
YPEL5
germline somatic somatic_double_hit silenced homdel

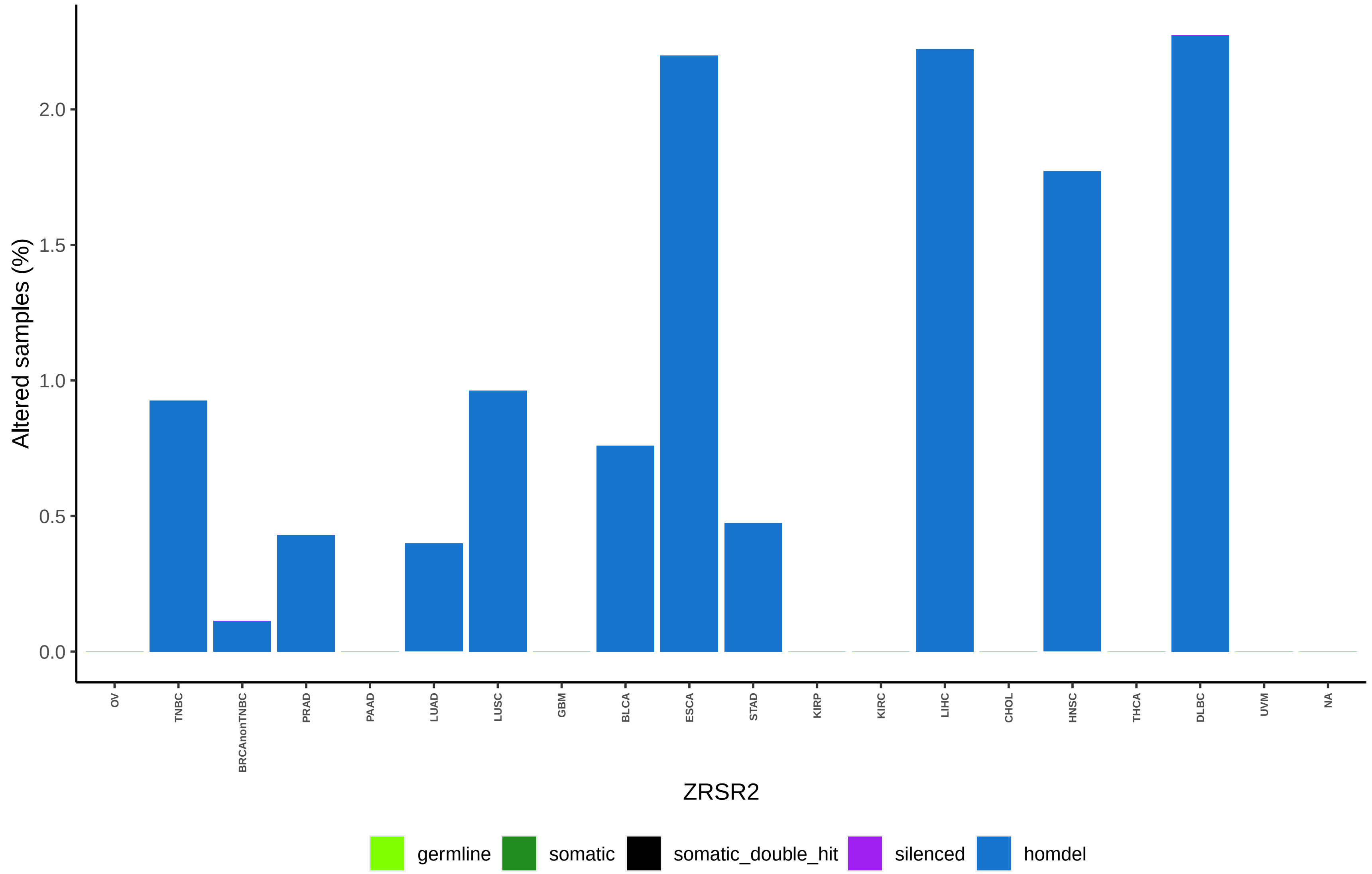
Altered samples (%)
0.0
0.5
1.0
1.5
2.0
OV
TNBC
BRCAnonTNBC
PRAD
PAAD
LUAD
LUSC
GBM
BLCA
ESCA
STAD
KIRP
KIRC
LIHC
CHOL
HNSC
THCA
DLBC
UVM
NA
ZRSR2
germline
somatic
somatic_double_hit
silenced
homdel

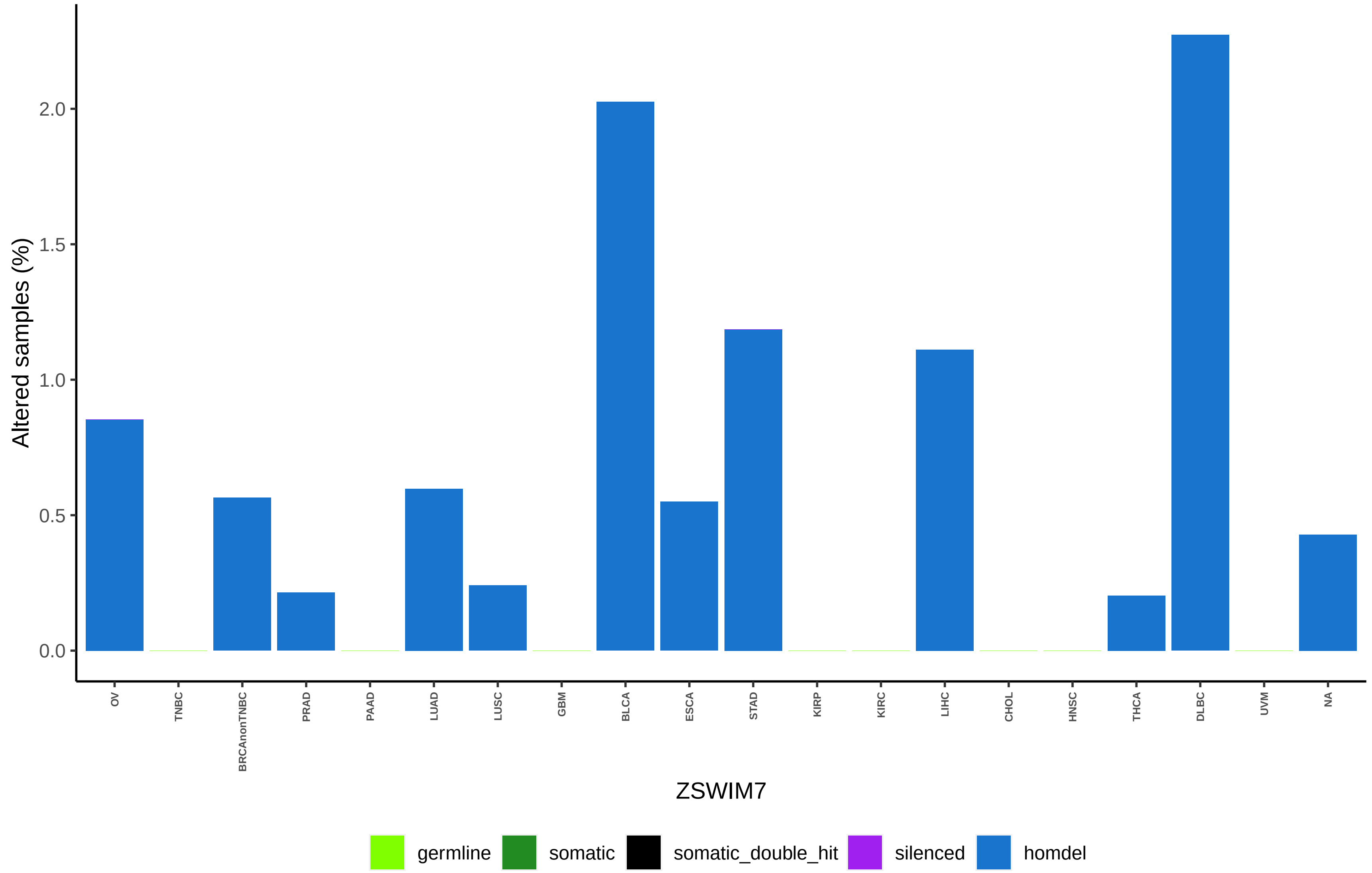
Altered samples (%)
0.0
0.5
1.0
1.5
2.0
OV
TNBC
BRCAnonTNBC
PRAD
PAAD
LUAD
LUSC
GBM
BLCA
ESCA
STAD
KIRP
KIRC
LIHC
CHOL
HNSC
THCA
DLBC
UVM
NA
ZSWIM7
germline
somatic
somatic_double_hit
silenced
homdel
